# Conditional power of antidepressant network meta-analysis

**DOI:** 10.1186/s12888-021-03094-5

**Published:** 2021-03-05

**Authors:** Lisa Holper

**Affiliations:** University Hospital of Psychiatry, University of Zurich, Zurich, Switzerland

**Keywords:** Conditional power, Sample size, Conclusive evidence, Network meta-analysis, Antidepressants

## Abstract

**Background:**

Conditional power of network meta-analysis (NMA) can support the planning of randomized controlled trials (RCTs) assessing medical interventions. Conditional power is the probability that updating existing inconclusive evidence in NMA with additional trial(s) will result in conclusive evidence, given assumptions regarding trial design, anticipated effect sizes, or event probabilities.

**Methods:**

The present work aimed to estimate conditional power for potential future trials on antidepressant treatments. Existing evidence was based on a published network of 502 RCTs conducted between 1979-2018 assessing acute antidepressant treatment in major depressive disorder (MDD). Primary outcomes were efficacy in terms of the symptom change on the Hamilton Depression Scale (HAMD) and tolerability in terms of the dropout rate due to adverse events. The network compares 21 antidepressants consisting of 231 relative treatment comparisons, 164 (efficacy) and 127 (tolerability) of which are currently assumed to have inconclusive evidence.

**Results:**

Required sample sizes to achieve new conclusive evidence with at least 80% conditional power were estimated to range between N = 894 - 4190 (efficacy) and N = 521 - 1246 (tolerability). Otherwise, sample sizes ranging between N = 49 - 485 (efficacy) and N = 40 - 320 (tolerability) may require stopping for futility based on a boundary at 20% conditional power. Optimizing trial designs by considering multiple trials that contribute both direct and indirect evidence, anticipating alternative effect sizes or alternative event probabilities, may increase conditional power but required sample sizes remain high. Antidepressants having the greatest conditional power associated with smallest required sample sizes were identified as those on which current evidence is low, i.e., clomipramine, levomilnacipran, milnacipran, nefazodone, and vilazodone, with respect to both outcomes.

**Conclusions:**

The present results suggest that conditional power to achieve new conclusive evidence in ongoing or future trials on antidepressant treatments is low. Limiting the use of the presented conditional power analysis are primarily due to the estimated large sample sizes which would be required in future trials as well as due to the well-known small effect sizes in antidepressant treatments. These findings may inform researchers and decision-makers regarding the clinical relevance and justification of research in ongoing or future antidepressant RCTs in MDD.

**Supplementary Information:**

The online version contains supplementary material available at (10.1186/s12888-021-03094-5).

## Background

Research suggests that a majority of randomized clinical trials (RCTs) on medical interventions may not be justified based on established evidence, but contain unjustified research. Justified clinical trials may be defined as trials designed around a clear hypothesis around which uncertainty exists and that uncertainty should be as established through systematic reviews or network meta-analyses (NMA) based on existing evidence [1]. This is of relevance because estimated costs of each piece of evidence in a series of RCTs increases across decades [2, 3]. Optimizing the number of clinical trials to scientifically justifiable amounts is therefore recommended to save resources, reduce exposure of patients to less effective treatments, and allow for earlier uptake of treatment recommendations in practice [1].

Conditional power of NMA has been introduced as a concept to optimize trial designs thereby contributing to the reduction of unjustified research [4–6]. Conditional power is the probability that updating existing inconclusive evidence in NMA with additional trial(s) will result in conclusive evidence, given assumptions regarding trial design, anticipated effect sizes, or event probabilities [7, 8]. A key issue when designing a RCT is to determine how large the sample size needs to be in order to achieve a desirable level of power given a predefined significance level *α* [7]. Further, some interventions may not achieve high levels of power when considered within a single trial in isolation. In such situations, two or more RCTs in combination may be appropriate to form a cumulative synthesis of findings from RCTs addressing the same question [5, 6]. This situation may also arise if a direct treatment comparison of interest includes treatments that are known to be poorly tolerated in patients (e.g., due to known adverse events); therefore, adding indirect evidence including only better tolerable treatments in future trials may be more appropriate for the evidence to become conclusive. If conditional power analysis suggests for example at least 80% conditional power, which conventionally implies that trial(s) investigating a true effect will correctly reject the null hypothesis [9], together with a reasonable required sample size, further research may be promising. Otherwise, if such an analysis suggests for example less than 20% conditional power, which conventionally may be regarded as futility boundary with values below indicating that a trial is likely to be futile under the null hypothesis [10], then it may be recommended to refrain from further RCTs on a given intervention to save resources.

The present work aimed to estimate conditional power for NMA on antidepressant treatments. The analysis was based on a published network known as the GRISELDA dataset [11], contributing 502 RCTs for the acute treatment of adult major depressive disorder (MDD) conducted between 1979-2018 [12]. Together the network compares 21 antidepressants, considering outcomes such as efficacy in terms of the symptom change on the Hamilton Depression Scale (HAMD) [13] and tolerability in terms of dropout rate due to adverse events (**Supplement** 1 Fig. S1).

At the time of writing (as of October 2020), four ongoing RCTs can be found on clinicaltrials.gov that cover one or more of the afore-mentioned antidepressants and fit the inclusion criteria of the present data set (NCT04364997, intervention: bupropion (BUP), escitalopram (ESC), mirtazapine (MIR), sertraline (SER), venlafaxine (VEN), planned sample size N = 400, estimated start and completion dates Jun-18 to Dec-22, Beijing Anding Hospital, China [14]; NCT03538691, intervention: citalopram (CIT), duloxetine (DUL), escitalopram (ESC), fluoxetine (FLO), paroxetine (PAR), sertraline (SER), venlafaxine (VEN) versus placebo (PLA), planned sample size N = 1450, estimated start and completion dates Jul-18 to Sep-22, Otsuka Pharmaceutical Development & Commercialization, Inc. [15]; NCT04345471, intervention: desvenlafaxine (DES) versus placebo (PLA), planned sample size N = 594, estimated start and completion dates May-20 to Dec-22, Mochida Investigational sites, Japan [16]; NCT04422652, intervention: desvenlafaxine (DES) versus vortiozetine (VOR), planned sample size N = 600, estimated start and completion dates Aug-20 to Apr-26, H. Lundbeck A/S [17]).

For example, one of the most recent antidepressants is vortioxetine (VOR) approved in 2013 by the US Food and Drug Administration (FDA). The existing evidence on VOR comprises 17 RCTs (16 placebo-controlled RCTs, 1 head-to-head RCT) completed between 2007 - 2017 and published between 2012 - 2018 [18–34]. Based on this current evidence, VOR has been shown to be more effective (standardized mean difference (SMD) -0.29 [95%CI -0.38 - -0.20]), but less tolerable (odds ratio (OR) 1.48 [95%CI 1.15 - 1.89]) compared to placebo, with the evidence becoming conclusive in 2009 (efficacy) and 2011 (tolerability), respectively. An ongoing phase IV, double-bind RCT (NCT04448431 [35]) started in August 2020 with estimated completion date in April 2026. This RCT aims to compare the efficacy of VOR versus desvenlafaxine (DES) in 600 MDD patients that have tried one available treatment without getting the full benefit, with the primary outcome being the change in the Montgomery and Åsberg Depression Rating Scale (MADRS) from baseline to week 8. Based on current evidence, the comparison DES:VOR is inconclusive in terms of efficacy (SMD -0.06 [95%CI -0.19 - 0.08]) and tolerability (OR 0.80 [95%CI 0.54 - 1.18]); suggesting a slight yet inconclusive advantage for VOR compared to DES with respect to both outcomes. To estimate whether the advantage for VOR may turn into conclusive evidence, conditional power analysis may support the decision whether the ongoing research on that comparison is promising or otherwise futile. This example shows how the present work may inform decision-makers and researchers regarding the expected clinical relevance of ongoing and future antidepressant RCTs that aim to challenge antidepressant treatment recommendations.

## Methods

### Data sources

A total of 535 RCTs (445 published trials, 90 unpublished trials) were identified on the acute treatment of MDD conducted between 1979 and 2018. 522 trials constituted the GRISELDA dataset [11] provided by Cipriani et al. [12]. Additional 13 trials [34, 36–47] were identified by own literature search. Together the network compares 21 antidepressants, agomelatine (AGO), amitriptyline (AMI), bupropion (BUP), citalopram (CIT), clomipramine (CLO), desvenlafaxine (DES), duloxetine (DUL), escitalopram (ESC), fluoxetine (FLO), fluvoxamine (FLV), levomilnacipran (LEV), milnacipran (MIL), mirtazapine (MIR), nefazodone (NEF), paroxetine (PAR), reboxetine (REB), sertraline (SER), trazodone (TRA), venlafaxine (VEN), vilazodone (VIL), vortioxetine (VOR), and placebo (PLA). The supplementary appendix provides a PRISMA flow-chart (Preferred Reporting Items for Systematic Reviews and Meta-Analyses) [48] detailing the study selection process (**Supplement** 1, **Fig. S1a, Tab. S1**), a complete list of the included studies (**Supplement** 1, **Tab. S4**).

Two outcomes were considered. The continuous outcome efficacy in terms of the symptom change on the Hamilton Depression Scale (HAMD) [13], estimated on the standardized mean difference (SMD) scale, was available in 438 trials (99 direct comparisons) with a total sample size of N = 109’254 (median sample size N = 249 [range N = 7 - 821]). The binary outcome tolerability in terms of the dropout rate due to adverse events, estimated on the odds ratio (OR) scale, was available in 438 trials (99 direct comparisons) with a total sample size of N = 105’616 (median sample size N = 241 [range N = 3 - 657]). The final dataset, containing information on either one of the outcomes, consisted of 502 trials. Other commonly used outcomes related to the effectiveness of antidepressants, such as response and remission rates, were not considered due to well-known methodological difficulties arising from dichotomization, such as reduced statistical power and inflated effect sizes [49–52].

Study year was defined as study year of completion, study year of publication, or year of drug approval from the FDA, where available in this order; preference was given to study year of completion, because unpublished trials, by definition, have no year of publication [53]. The resulting study year range was 1977-2017.

### Conditional power

Conditional power was estimated using the ConditionalPower package provided by Nikolakopoulou et al. [7, 8, 54] in R [55]. Briefly, conditional power in NMA can be described as [7], for example for a comparison of interest:

1 $$ {\begin{aligned} CP = \phi \left(\frac{-z_{a/2} * \sqrt{C} - H*M}{\sqrt{H^{N}*\nu^{N} * \left(H^{N}\right)^{\prime}}}\right) + \phi \left(\frac{-z_{a/2} * \sqrt{C} + H*M}{\sqrt{H^{N}*\nu^{N} * \left(H^{N}\right)^{\prime}}}\right) \end{aligned}}  $$

where *C* represents the covariance matrix of the NMA (direct and indirect) effect estimates, the vector *M* contains the NMA (direct and indirect) effect estimates of the old pairwise meta-analyses and the alternative effect sizes for the comparison of interest, the matrices *H* and *H*^*N*^ connect the NMA (direct and indirect) effect estimates to the pairwise (direct) effects derived from old or new trials, respectively, and the vector *ν*^*N*^ represents the variances of the pairwise (direct) effect estimates derived from new trials. The reader may be referred to Nikolakopoulou et al. [7] for further details.

Conditional power was estimated across a range of possible N = 1 - 5000 sample sizes assuming 1:1 randomization between treatment arms. Results were reported in terms of two conditional power indices quantifying sample sizes:

- ***N***_***CP***=***20****%*_: Sample size at 80% conditional power, which conventionally implies that a trial investigating a true effect will correctly reject the null hypothesis 80% of the time and will report a false negative (commit a type II error) in the remaining 20% of cases [9].
- ***N***_***CP***=***80****%*_: Sample size at 20% conditional power, which conventionally may be regarded as futility boundary with values below indicating that a trial is likely to be futile under the null hypothesis [10].

Three parameters were considered for each outcome of interest:

- **Trial design:** The main analysis considered a trial design with a ratio of direct/indirect evidence (*r*) of *r* = 1/0. The ratio *r* = 1/0 indicates that conditional power for each treatment comparison was assessed by updating the network with one new trial contributing direct evidence regarding the comparison of interest, but without any new trials contributing indirect evidence. A sensitivity analysis was conducted to estimate conditional power by updating with trial design represented by two additional ratios of *r* = 1/1 and *r* = 1/2. The ratio *r* = 1/1 indicates that conditional power for each treatment comparison was assessed by updating the network with one new trial contributing direct and one new trial contributing indirect evidence regarding the comparison of interest (for this trial design 41 possible combinations for each comparison were computed), whereas the ratio *r* = 1/2 indicates that conditional power for each treatment comparison was assessed by updating the network with one new trial contributing direct and two new trial contributing indirect evidence regarding the comparison of interest (for this trial design 820 possible combinations for each comparison were computed). Results were reported in terms of the optimal trial designs for each comparison, i.e., those with smallest *N*_*CP*=80*%*_.
- **Effect size:** The main analysis considered anticipated treatment effects (*f*_*xy*_) set equal to the relative effect estimates (i.e., the relative effects between competing treatments of interest) observed in the network (*f*_*xyN*_). A sensitivity analysis was conducted to estimate conditional power at alternative effect sizes (*f*_*xy*_ = 0.01, 0.1, 0.2, 0.3, 0.5, 0.8) in terms of Cohen’s d (small effect d = 0.2, moderate effect d = 0.5, large effect d = 0.8) [56].
- **Event probability:** The main analysis considered anticipated event probabilities (*pc*) set equal to the average event probabilities observed in the entire network (*pc*_*N*_). For the outcome efficacy, anticipated average event probability (*pc*_*N*_ = 0.17) was calculated in terms of the proportion of change on the HAMD of at least 4 points (number of trials with change ≥4 points divided by the number of trials with change <4 points) corresponding to Cohen’s d = 0.5 [57]. For the outcome tolerability, anticipated average event probability (*pc*_*N*_ = 0.08) was calculated in terms of the proportion of dropouts (total number of dropouts divided by the total sample size in the network) [7]. A sensitivity analysis was conducted to estimate conditional power at alternative event probabilities in terms of small to large event risks (*pc* = 0.01, 0.1, 0.2, 0.3, 0.5).

Conditional power is typically estimated for direct comparisons observed in the network [7]. The antidepressant network however contains only 99 direct comparisons out of a total of 231 comparisons. It was therefore hypothesized that inclusion of all competing treatment comparisons in the network would be of clinical interest. For this purpose, dummy connections (with sample size = 1) were created to connect treatment comparisons not-directly observed in the network, and subsequently included in the analysis. Dummy connections did not affect relative treatment effects as assessed by the Pearson correlation between original and dummy effect sizes (efficacy *r* = 0.999, tolerability *r* = 0.995) (**Supplement** 1, **Fig. S1d**). Between-trial heterogeneity was assumed to be equal to that observed in the original NMA.

All results reported in the article can be found in the supplementary appendices (**Supplement** 1 & 2). The data set used in the analysis is provided in comma-separated values (CSV) format (**Supplement** 3).

## Results

### Existing evidence

**Fig. 1:**
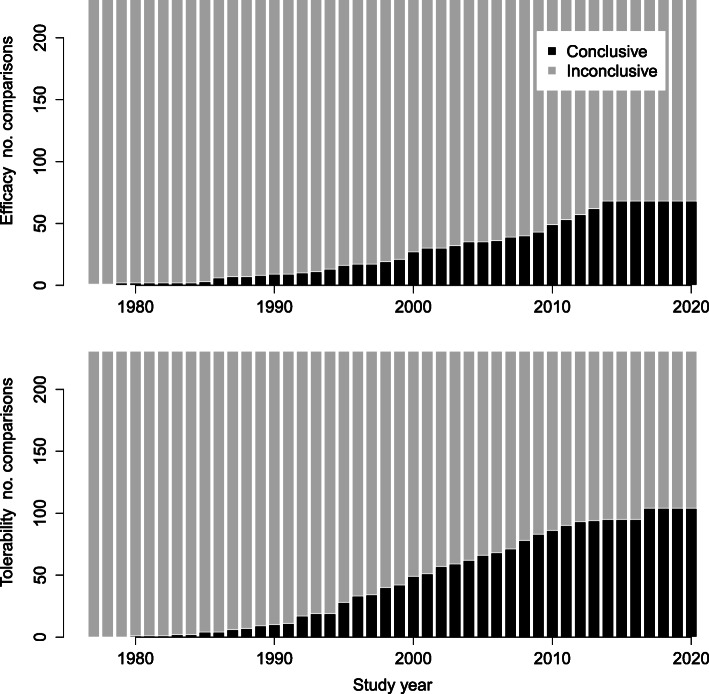
Evidence across study year. Bar plots illustrating the cumulative sum of comparisons with conclusive versus inconclusive evidence across study year with respect to the two outcomes efficacy and tolerability. The total number of treatment comparisons is 231

The cumulative evolution of conclusive evidence in the antidepressant network across decades is illustrated in Fig. 1, for the two outcomes efficacy and tolerability. Since 2017, no new conclusive evidence has been observed. As of 2020, the ratio of the number of comparisons with conclusive evidence versus inconclusive evidence was found to be half the size for the outcome efficacy (ratio = 0.41, conclusive N = 67 versus inconclusive N = 164) compared to tolerability (ratio = 0.82, conclusive N = 104 versus inconclusive N = 127).

### Conditional power main analysis

**Fig. 2:**
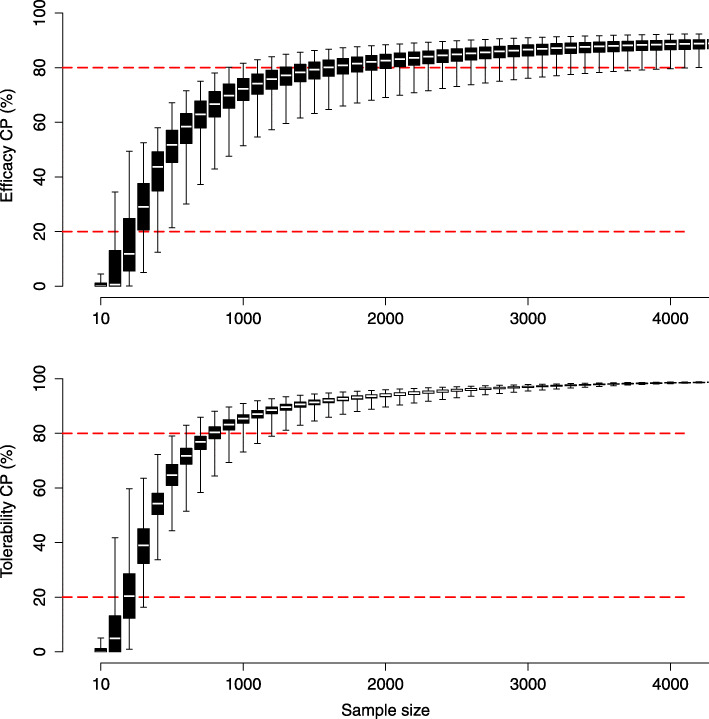
Conditional power. Box plots illustrating conditional power (CP) across all comparisons with inconclusive evidence as a function of sample size with respect to the two outcomes efficacy and tolerability. Whiskers of the box plots extend to the most extreme data values. Horizontal red dashed lines indicate 20% and 80% conditional power at which sample sizes (*N*_*CP*=20*%*_, *N*_*CP*=80*%*_) were estimated. Results are shown based on the main analysis considering a trial design ratio of *r* = 1/0, anticipated alternative effect sizes equal to the network estimates (*f*_*xyN*_), and anticipated event probabilities equal to the average network event probability (*pc*_*N*_)

**Fig. 3:**
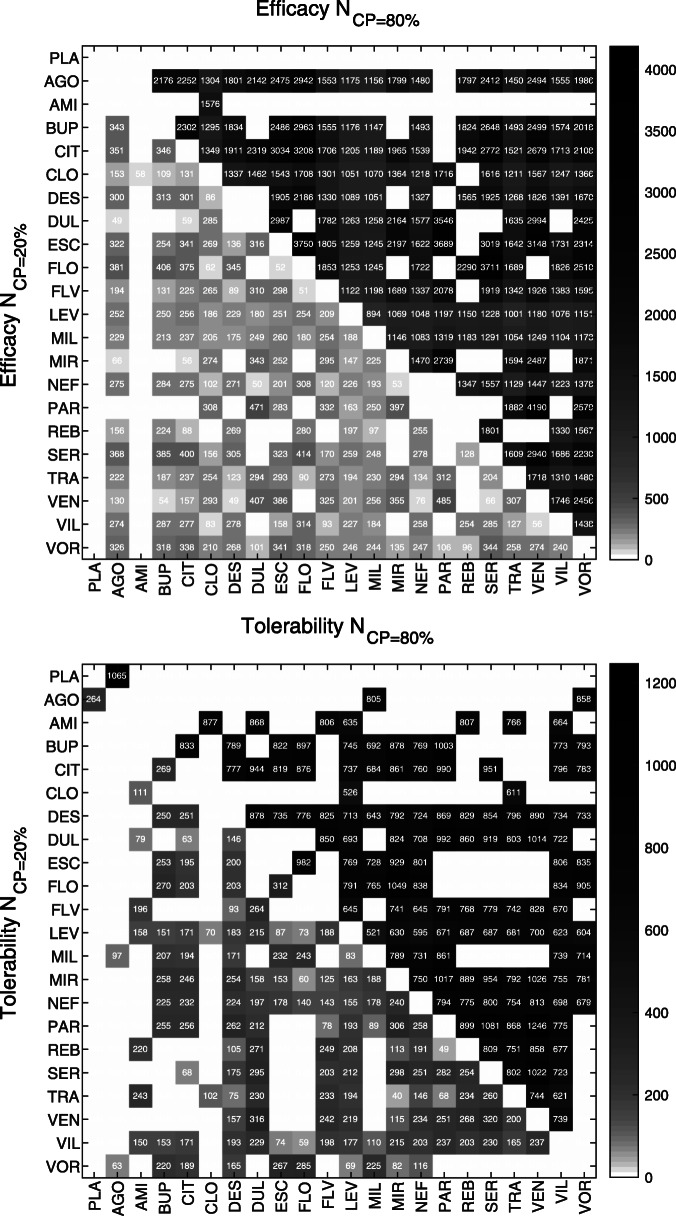
Sample size. Heat map illustrating sample size at 20% (*N*_*CP*=20*%*_) (lower triangles) versus 80% conditional power (*N*_*CP*=80*%*_) (upper triangles) for individual comparisons with respect to the two outcomes efficacy and tolerability. Colormap is log scaled for better visibility. Comparisons with conclusive evidence are marked (white). Results are shown based on the main analysis considering a trial design ratio of *r* = 1/0, anticipated alternative effect sizes equal to the network estimates (*f*_*xyN*_), and anticipated event probabilities equal to the average network event probability (*pc*_*N*_)

**Fig. 4:**
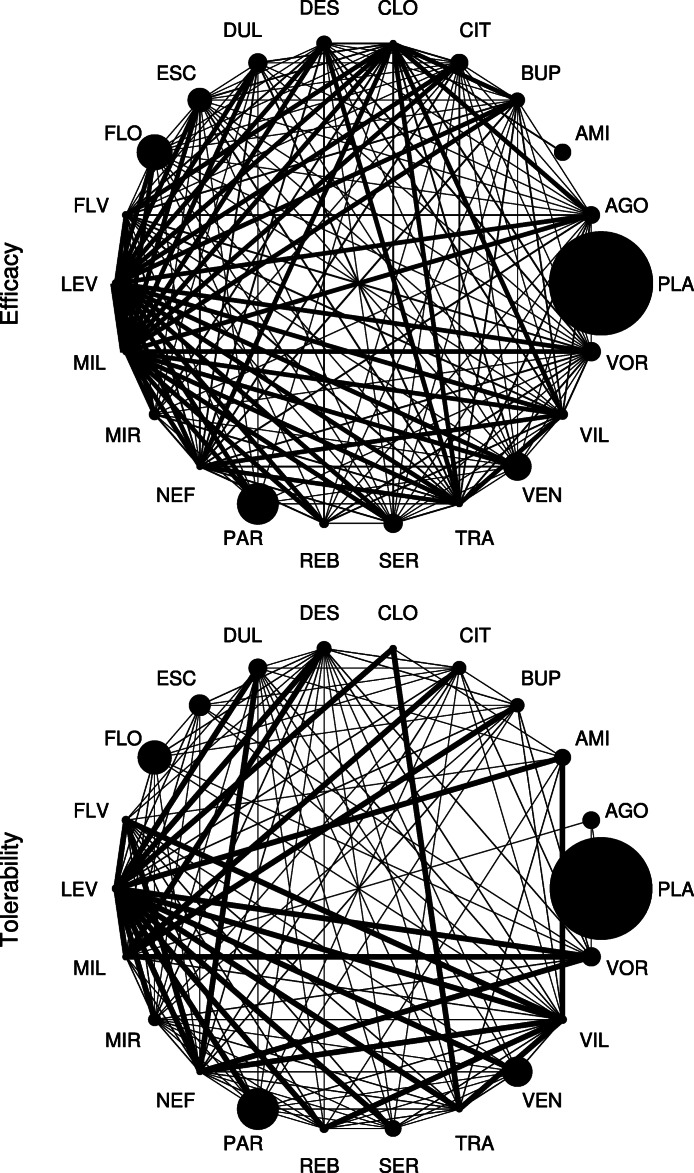
Network graphs. Network graphs illustrating treatment comparisons with inconclusive evidence with respect to the two outcomes efficacy and tolerability. Circle size is proportionate to actual sample size. Line width is inverse proportionate to the sample size at 80% conditional power (*N*_*CP*=80*%*_), such that thicker connections indicate smaller sample sizes and thus greater conditional power. Thickness is log scaled for better visibility. Results are shown based on the main analysis considering a trial design ratio of *r* = 1/0, anticipated alternative effect sizes equal to the network estimates (*f*_*xyN*_), and anticipated event probabilities equal to the average network event probability (*pc*_*N*_). See the supplementary appendix for graphs of the original network (**Supplement** 1, **Fig. S2)**

The estimated strength of conditional power across all comparisons with inconclusive evidence is illustrated in Fig. 2, based on the main analysis considering anticipated effect sizes set equal to *f*_*xyN*_ and anticipated event probabilities set equal to *pc*_*N*_. The figure further demonstrates how the two conditional power indices quantifying sample sizes were derived, i.e., sample sizes at 20% and 80% conditional power (*N*_*CP*=20*%*_, *N*_*CP*=80*%*_). Across all comparisons with inconclusive evidence, required sample sizes at 80% conditional power (*N*_*CP*=80*%*_) were estimated to be approximately double the size for efficacy (median N = 1586, range N = 894 - 4190) than those required for tolerability (median N = 791, range N = 521 - 1246). By contrast, sample sizes at the futility boundary of 20% conditional power (*N*_*CP*=20*%*_) were estimated to be comparable between outcomes (efficacy median N = 250 [range N = 49 - 485], tolerability median N = 198 [range N = 40 - 320]) (Table 1). The relation between the two indices, *N*_*CP*=20*%*_ and *N*_*CP*=80*%*_, for each individual comparison is detailed in Fig. 3. The network graphs depicted in Fig. 4 finally summarize the sample size needed to achieve conditional power. To translate these indices to the individual antidepressant level, the medians of the two indices, *N*_*CP*=20*%*_ and *N*_*CP*=80*%*_, were computed across all inconclusive comparisons including each individual antidepressants. Antidepressants with the smallest median sample sizes were identified as CLO, LEV, MIL, NEF, and VIL with respect to both outcomes (Fig. 4). This is reasonable as these antidepressants (or better the associated comparisons) are the once on which current direct evidence is low. Thus, although estimated conditional power differed in the overall strength between outcomes, with that for efficacy being weaker compared to tolerability, the proportional strength of conditional power in individual treatment comparisons was comparable (Pearson *r* = 0.81). The supplementary appendix provides details on the conditional power for each individual comparison (**Supplement** 1, **Tab. S2 and Supplement** 2).

**Table 1:** Conditional power

				***N***_***CP***=***20****%*_	***N***_***CP***=***80****%*_
				Sample size	Sample size
	Trial design	Effect size	Event probability	median [range]	median [range]
**Efficacy**					
**Main analysis**	*r* = 1/0	*f*_*xyN*_	*pc*_*N*_ = 0.17	250 [49 - 485]	1586 [894 - 4190]
**Sensitivity analysis**	*r* = 1/1	*f*_*xyN*_	*pc*_*N*_ = 0.17	126 [10 - 384]	1198 [720 - 3464]
	*r* = 1/2	*f*_*xyN*_	*pc*_*N*_ = 0.17	117 [10 - 222]	994 [624 - 2586]
	*r* = 1/0	*f*_*xy*_ d = 0.01	*pc*_*N*_ = 0.17	250 [49 - 483]	1576 [916 - 4176]
	*r* = 1/0	*f*_*xy*_ d = 0.1	*pc*_*N*_ = 0.17	245 [49 - 509]	1612 [866 - 4371]
	*r* = 1/0	*f*_*xy*_ d = 0.2	*pc*_*N*_ = 0.17	242 [49 - 538]	1644 [817 - 4682]
	*r* = 1/0	*f*_*xy*_ d = 0.3	*pc*_*N*_ = 0.17	238 [49 - 566]	1628 [773 - 9660]
	*r* = 1/0	*f*_*xy*_ d = 0.5	*pc*_*N*_ = 0.17	236 [47 - 622]	1668 [695 - 9772]
	*r* = 1/0	*f*_*xy*_ d = 0.8	*pc*_*N*_ = 0.17	228 [44 - 699]	1754 [595 - 9880]
	*r* = 1/0	*f*_*xyN*_	*pc* = 0.01	706 [51 - 1407]	3678 [2471 - 10000]
	*r* = 1/0	*f*_*xyN*_	*pc* = 0.1	373 [50 - 741]	2384 [1370 - 9927]
	*r* = 1/0	*f*_*xyN*_	*pc* = 0.2	212 [48 - 416]	1367 [781 - 3793]
	*r* = 1/0	*f*_*xyN*_	*pc* = 0.3	136 [43 - 318]	1044 [599 - 3217]
	*r* = 1/0	*f*_*xyN*_	*pc* = 0.5	96 [36 - 271]	874 [503 - 2877]
**Tolerability**					
**Main analysis**	*r* = 1/0	*f*_*xyN*_	*pc*_*N*_ = 0.57	198 [40 - 320]	791 [521 - 1246]
**Sensitivity analysis**	*r* = 1/1	*f*_*xyN*_	*pc*_*N*_ = 0.57	104 [64 - 278]	738 [414 - 1258]
	*r* = 1/2	*f*_*xyN*_	*pc*_*N*_ = 0.57	102 [81 - 168]	675 [480 - 1161]
	*r* = 1/0	*f*_*xy*_ d = 0.01	*pc*_*N*_ = 0.57	207 [41 - 318]	766 [619 - 1202]
	*r* = 1/0	*f*_*xy*_ d = 0.1	*pc*_*N*_ = 0.57	192 [40 - 321]	782 [569 - 1283]
	*r* = 1/0	*f*_*xy*_ d = 0.2	*pc*_*N*_ = 0.57	181 [40 - 343]	791 [513 - 1373]
	*r* = 1/0	*f*_*xy*_ d = 0.3	*pc*_*N*_ = 0.57	170 [39 - 366]	817 [471 - 1462]
	*r* = 1/0	*f*_*xy*_ d = 0.5	*pc*_*N*_ = 0.57	146 [39 - 420]	908 [409 - 1782]
	*r* = 1/0	*f*_*xy*_ d = 0.8	*pc*_*N*_ = 0.57	121 [40 - 615]	1027 [320 - 2497]
	*r* = 1/0	*f*_*xyN*_	*pc* = 0.01	319 [41 - 523]	1258 [824 - 1991]
	*r* = 1/0	*f*_*xyN*_	*pc* = 0.1	153 [41 - 277]	662 [442 - 1046]
	*r* = 1/0	*f*_*xyN*_	*pc* = 0.2	55 [37 - 117]	371 [257 - 582]
	*r* = 1/0	*f*_*xyN*_	*pc* = 0.3	40 [32 - 72]	277 [207 - 435]
	*r* = 1/0	*f*_*xyN*_	*pc* = 0.5	34 [31 - 55]	227 [192 - 365]

Listed are median [range] of sample sizes at 20% and 80% conditional power (*N*_*CP*=20*%*_, *N*_*CP*=80*%*_) with respect to the three parameters considered in the sensitivity analysis, i.e., trial design (*r*), effect size (*f*_*xy*_), and event probability. The first row for each outcome lists results obtained in the main analysis considering a trial design with a ratio of direct/indirect evidence of *r* = 1/0, with anticipated effect sizes set equal to the network estimates (*f*_*xyN*_), and anticipated event probabilities set equal to the average network event probabilities (*pc*_*N*_). The remaining rows list results obtained in the sensitivity analyses considering trial designs with ratios of direct/indirect evidence of *r* = 1/1 and *r* = 1/2, varying anticipated effect sizes (*f*_*xy*_ in terms of Cohen’s d), and varying anticipated event probabilities (*pc*). The supplementary appendix provides details on sensitivity analyses (**Supplement** 1, **Fig. S3, Tab. S3**)

### Conditional power sensitivity analyses

Sensitivity analysis quantifying the trial design ratio between direct/indirect evidence (*r*) suggested that adding indirect evidence may considerably increase conditional power and consequently reduce required sample sizes. Compared to a trial design ratio of *r* = 1/0, considering trial design ratios of *r* = 1/1 and *r* = 1/2 reduced median sample sizes (*N*_*CP*=80*%*_) by median percentages changes of -24% and -35% for efficacy and -7% and -15% for tolerability (Table 1).

By contrast, sensitivity analysis assessing varying anticipated effect sizes suggested that the impact of *f*_*xy*_ on the strength of conditional power was small. Considering larger effect sizes (e.g., d = 0.8 in terms of Cohen’s, which is indeed unrealistic) than those observed in the network estimates (*f*_*xyN*_) would increase sample sizes by up to 5% (efficacy) and 3% (tolerability), whereas smaller effect sizes (e.g., d = 0.01 in terms of Cohen’s) had basically no impact on sample sizes (0% efficacy, -1% tolerability) (Table 1).

Last, sensitivity analysis assessing varying event probabilities suggested a relatively larger impact of *pc* on the strength of conditional power. However, considering the current evidence in terms of average event probabilities (efficacy *pc*_*N*_ = 0.17, tolerability *pc*_*N*_ = 0.08), larger event probabilities may hardly be considered (Table 1). The supplementary appendix provides details on all sensitivity analyses (**Supplement** 1, **Fig. S3, Tab. S3**).

## Discussion

The recent NMA by Cipriani et al. [12] provided evidence regarding the ongoing debate on the effectiveness of antidepressant treatment. Today, two years after the publication of the NMA, the question aires whether additional RCTs updating the evidence would pay off. Current ongoing RCTs [14–17] may contribute to answer the question, but final results may only be expected after estimated completion of the RCTs (completion dates 2022 - 2026). It may therefore be of clinical interest to estimate the probability whether the current research may lead to updates in treatment recommendations or whether it may be considered unjustified.

Overall, the present findings value the probability of achieving new conclusive evidence in antidepressant treatment recommendations that goes beyond current evidence to be low. Though, sufficient conditional power may be obtained for a majority of evaluated treatment comparisons (Fig. 4), there are substantial limitations in terms of both required sample sizes and expected effect sizes.

Considering median sample sizes in the in the four ongoing RCTs (range N = 400 - 1450) [14–17], required sample sizes obtained by the present analysis to achieve conventionally recommended power of at least 80% [9] were estimated to be more than double (tolerability) or even three times (efficacy) the size and may not even exceed the estimated futility boundaries (Table 1). Though, sample sizes may be reduced using optimized trial designs including additional indirect evidence, the associated research costs when conducting multiple trials may not pay off.

It should be noted that the present work is limited in the evaluation of optimal trial designs evaluating the relation between direct and indirect evidence. Nikolakopoulou et al. [54] demonstrated how decisions in future trials may be supported by conditional power analyses considering not only ’different ratios of the number of trials’ contributing direct versus indirect evidence, as done in the current work, but also by considering ’different ratios of the sample size between trials’ assessing direct versus indirect information. An extensive analysis assessing these ratios is feasible in small networks or may be applied to selected treatment comparisons of interest based on a priori hypotheses. The large treatment space in the present network, however, did not allow for such extensive sensitivity analyses due to practical reasons considering both processing time and exponential result dimension. Future research should therefore consider the present findings as an approximation for a more detailed breakdown of the evidence.

Compared to the impact of trial designs on reducing sample sizes, the impact of varying effect sizes or event probabilities may be assumed of less practical importance; this is because trial designs can be experimentally modified, whereas effect sizes and event probabilities are inherently limited by the existing evidence of the various treatments. In particular, considering the well-known overall small effect sizes for efficacy in antidepressants in the conclusive treatment comparisons (i.e., drug-placebo differences with a median d = 0.3 in terms of Cohen’s d [57]) and the even smaller effect sizes in so far inconclusive relative treatment comparisons (median d <0.1 in terms of Cohen’s d [57]) (**Supplement** 1, **Tab. S2**), the clinical relevance of additional trials aiming to challenge current antidepressant treatment recommendations may be low. In other words, it may be questioned whether any additional RTCs on antidepressant treatment can challenge the current treatment recommendations.

Referring to the example in the introduction, the present results may be applied to judge the conditional power of the ongoing RCT (NCT04448431 [35]) aiming to compare the efficacy of VOR versus DES. Though, current evidence may assume a trend towards the advantage of VOR compared to DES in terms of both efficacy and tolerability **Supplement** 1, **Fig. S1**), the probability of achieving conclusive evidence at reasonable sample sizes is low. The present analysis suggested required sample sizes to achieve at least 80% conditional power (*N*_*CP*=80*%*_) of N = 1670 and N = 733 in terms of efficacy and tolerability, respectively (Fig. 3). These estimated sample sizes are considerably larger than the planned sample size of N = 600 [35]. Indeed, the planned sample size of N = 600 corresponds to approximately 56% (efficacy) and 74% (tolerability) (**Supplement** 2), and may thus be considered too low to reach new conclusive evidence in an updated NMA.

The above-mentioned example demonstrates the importance of a priori conditional power analyses, if it is the aim of a RCT to challenge current treatment recommendations. Based on the information available in the ongoing RCTs, it is unclear whether a priori conditional power analysis has been performed. The results expected after the completion of the ongoing RCTs will show whether a priori conditional power analysis could have contributed to improved trial designs, and thus would have saved resources in terms of clinical trial costs.

It should however be made clear that the ongoing RCTs may focus on primary aims other than challenging current antidepressant treatment recommendations. In other words, and they may have not been indented to be conditionally powered for possible future updating of NMAs, but may indeed be sufficiently powered as stand-alone trials. As discussed by Salanti and Nikolakopoulou [58], when NMA is deemed inconclusive and future trials should be planned, specific recommendations about what sort of trials should be planned are required. Trials can be planned to reduce risk of bias in particular comparisons, to explain heterogeneity, or to inform outcomes for which evidence is imprecise. When the aim is to included the planned trial in an updated NMA later on, trials may not be considered as stand-alone trials but may be seen as sequential additions to the existing evidence. The power and findings of individual trials are thus not of interest; rather, the conditional power of the NMA when the new trial is added and the resulting summary effect are of importance. Consequently, when NMA is deemed inconclusive because of imprecision, sample size calculations should be based on the conditional power of an updated NMA.

With this in mind, the present work should not be misunderstood or lead to possible miss-use of conditional power analyses. Weber et al. [59] raised that fundamental question regarding the use of conditional power analyses by asking whether “it is appropriate to gain power for an updated NMA by in- or decreasing the number of planned future trials while manipulating the power of each of the individual planned future trials?” The authors argued that traditional methods of power analysis are still favorable due to the fact that drug licensing is based on stand-alone RCT. Regardless of planning one or multiple trials, trials planned using conditional power may require different sample sizes (smaller or larger) than those planned using traditional power analysis aimed to achieve stand-alone conclusiveness. In other words, “individual RCTs should always be designed to satisfy their objectives and stand-alone studies (should not be) substituted by a meta-analysis of trials of inadequate size” [60].

## Conclusions

In conclusion, the present analysis may inform decision-makers and researchers in the planning future antidepressant trials in MDD. Results suggests that new conclusive evidence leading to potential updates in antidepressant treatment recommendations may hardly be achieved within reasonable trial scales. Limiting the use of the presented conditional power analysis are primarily due to the estimated large sample sizes which would be required in future trials as well as due to the overall well-known small effect sizes in antidepressant treatments. These findings may be of importance to evaluate the clinical relevance and justification of research in ongoing or future RCTs on antidepressant treatments in MDD.

## Supplementary Information

**Additional file 1** Supplement1 provides a flow chart and checklist according to the PRISMA statement, details on the results for individual treatment comparisons, and details on the sensitivity analyses.

**Additional file 2** Supplement2 provides illustrations of conditional power results for individual treatment comparisons.

**Additional file 3** Supplementary file 3.

## Data Availability

All results reported in the article can be found in the supplementary appendices (**Supplement** 1 & 2). The data set used in the analysis is provided in comma-separated values (CSV) format (**Supplement** 3).

## Abbreviations

AGO: Agomelatine
AMI: Amitriptyline
BUP: Bupropion
CIT: Citalopram
CLO: Clomipramine
DES: Desvenlafaxine
DUL: Duloxetine
ESC: Escitalopram
FLO: Fluoxetine
FLV: Fluvoxamine
LEV: Levomilnacipran
MIL: Milnacipran
MIR: Mirtazapine
NEF: Nefazodone
PAR: Paroxetine
PLA: Placebo
REB: Reboxetine
SER: Sertraline
TRA: Trazodone
VEN: Venlafaxine
VIL: Vilazodone
VOR: Vortioxetine
FDA: US food and drug administration
HAMD: Hamilton depression scale
MDD: Major depressive disorder
NMA: Network meta-analysis
OR: Odds ratio
PRISMA: Preferred reporting items for systematic reviews and meta-Analyses
RCT: Randomized clinical trials
SMD: Standardized mean difference

## References

[1] De Meulemeester J, Fedyk M, Jurkovic L, Reaume M, Dowlatshahi D, Stotts G, Shamy M (2018). Many randomized clinical trials may not be justified: a cross-sectional analysis of the ethics and science of randomized clinical trials. J Clin Epidemiol.

[2] Sertkaya A, Wong H-H, Jessup A, Beleche T (2016). Key cost drivers of pharmaceutical clinical trials in the united states. Clinical Trials.

[3] Moore TJ, Zhang H, Anderson G, Alexander G (2018). Estimated costs of pivotal trials for novel therapeutic agents approved by the us food and drug administration, 2015-2016. JAMA Intern Med.

[4] Langan D, Higgins J, Gregory W, Sutton A (2012). Graphical augmentations to the funnel plot assess the impact of additional evidence on a meta-analysis. J Clin Epidemiol.

[5] Sutton AJ, Cooper N, Jones DR, Lambert P, Thompson J, Abrams KR (2007). Evidence-based sample size calculations based upon updated meta-analysis. Statistics in Medicine.

[6] Roloff V, Higgins J, Sutton AJ (2013). Planning future studies based on the conditional power of a meta-analysis. Stat Med.

[7] Nikolakopoulou A, Mavridis D, Salanti G (2014). Using conditional power of network meta-analysis (nma) to inform the design of future clinical trials. Biom J.

[8] Salanti G, Nikolakopoulou A, Sutton AJ, Reichenbach S, Trelle S, Naci H, Egger M (2018). Planning a future randomized clinical trial based on a network of relevant past trials. Trials.

[9] Cohen J (1988). Statistical Power Analysis for the Behavioral Sciences.

[10] Walter SD, Han H, Guyatt GH, Bassler D, Bhatnagar N, Gloy V., Schandelmaier S, Briel M (2020). A systematic survey of randomised trials that stopped early for reasons of futility. BMC Med Res Methodol.

[11] Cipriani A. Cipriani et al_GRISELDA_Lancet 2018_Open dataset. Mendeley Data, V2. 2018. 10.17632/83rthbp8ys.2.

[12] Cipriani A, Furukawa T, Salanti G, Chaimani A, Atkinson L, Ogawa Y, Leucht S, Ruhe H, Turner EH, Higgins JPT, Egger M, Takeshima N, Hayasaka Y, Imai H., Shinohara K, Tajika A, Ioannidis JPA, Geddes J (2018). Comparative efficacy and acceptability of 21 antidepressant drugs for the acute treatment of adults with major depressive disorder: a systematic review and network meta-analysis. Lancet.

[13] Hamilton M (1960). A rating scale for depression. J Neurol Neurosurg Psychiatry.

[14] CSPC ZhongQi Pharmaceutical Technology Co. Ltd.Study of Desvenlafaxine in Treating Major Depressive Disorder (Clinicaltrials.gov Identifier NCT04364997). 2020. https://clinicaltrials.gov/ct2/show/NCT04364997.

[15] University of Texas Southwestern Medical Center, University of Washington, National Institute of Diabetes and Digestive and Kidney Diseases (NIDDK). Combination of Novel Therapies for CKD Comorbid Depression (Clinicaltrials.gov Identifier NCT04422652). 2020. https://ClinicalTrials.gov/show/NCT04422652.

[16] Mochida Pharmaceutical Company Ltd. |Pfizer. A Study of MD-120 in Patients With Depression (Clinicaltrials.gov Identifier NCT04345471). 2020. https://ClinicalTrials.gov/show/NCT04345471.

[17] Otsuka Pharmaceutical Development & Commercialization, Inc.A Trial to Evaluate the Efficacy, Safety & Tolerability of Brexpiprazole in the Maintenance Treatment of Adults With Major Depressive Disorder (Clinicaltrials.gov Identifier NCT03538691). 2021. https://ClinicalTrials.gov/show/NCT03538691.

[18] Alvarez E, Perez V, Dragheim M, Loft H, Artigas F (2012). A double-blind, randomized, placebo-controlled, active reference study of lu aa21004 in patients with major depressive disorder. Int J Neuropsychopharmacol.

[19] Jain R, Mahableshwarkar A, Jacobsen PL, Chen Y, Thase M (2013). A randomized, double-blind, placebo-controlled 6-wk trial of the efficacy and tolerability of 5 mg vortioxetine in adults with major depressive disorder. Int J Neuropsychopharmacol.

[20] Mahableshwarkar A, Jacobsen P, Chen Y, Serenko M, Trivedi M (2015). A randomized, double-blind, duloxetine-referenced study comparing efficacy and tolerability of 2 fixed doses of vortioxetine in the acute treatment of adults with mdd. Psychopharmacology (Berl).

[21] Mahableshwarkar A, Jacobsen P, Serenko M, Chen Y, Trivedi M (2015). A randomized, double-blind, placebo-controlled study of the efficacy and safety of 2 doses of vortioxetine in adults with major depressive disorder. J Clin Psychiatry.

[22] Baldwin DS, Loft H, Dragheim M (2012). A randomised, double-blind, placebo controlled, duloxetine-referenced, fixed-dose study of three dosages of lu aa21004 in acute treatment of major depressive disorder (mdd). Eur Neuropsychopharmacol.

[23] Henigsberg N, Mahableshwarkar A, Jacobsen P, Chen Y, Thase M (2012). A randomized, double-blind, placebo-controlled 8-week trial of the efficacy and tolerability of multiple doses of lu aa21004 in adults with major depressive disorder. J Clin Psychiatry.

[24] Katona C, Hansen T, Olsen C (2012). A randomized, double-blind, placebo-controlled, duloxetine-referenced, fixed-dose study comparing the efficacy and safety of lu aa21004 in elderly patients with major depressive disorder. Int Clin Psychopharmacol.

[25] Boulenger JP, Loft H, Olsen CK (2014). Efficacy and safety of vortioxetine (lu aa21004), 15 and 20 mg/day: a randomized, double-blind, placebo-controlled, duloxetine-referenced study in the acute treatment of adult patients with major depressive disorder. Int Clin Psychopharmacol.

[26] Jacobsen P, Mahableshwarkar A, Serenko M, Chan S, Trivedi M (2015). A randomized, double-blind, placebo-controlled study of the efficacy and safety of vortioxetine 10 mg and 20 mg in adults with major depressive disorder. J Clin Psychiatry.

[27] NCT, 01255787. Efficacy and safety study of vortioxetine (lu aa21004) for treatment of major depressive disorder. https://doi.org/https://clinicaltrials.gov/ct2/show/NCT01255787. Accessed 2020.

[28] NCT, 01355081. Efficacy study of vortioxetine (lu aa21004) for treatment of major depressive disorder. https://doi.org/https://clinicaltrials.gov/ct2/show/NCT01355081. Accessed 2020.

[29] McIntyre R, Lophaven S, Olsen CK (2014). A randomized, double-blind, placebo-controlled study of vortioxetine on cognitive function in depressed adults. Int J Neuropsychopharmacol.

[30] Wang G, Gislum M, Filippov G, Montgomery S (2015). Comparison of vortioxetine versus venlafaxine xr in adults in asia with major depressive disorder: a randomized, double-blind study. Curr Med Res Opin.

[31] NCT, 02279966. Efficacy of vortioxetine on cognitive dysfunction in working patients with major depressive disorder. https://doi.org/https://clinicaltrials.gov. Accessed 2020.

[32] Mahableshwarkar A, Zajecka J, Jacobson W, Chen Y, Keefe R (2015). A randomized, placebo-controlled, active-reference, double-blind, flexible-dose study of the efficacy of vortioxetine on cognitive function in major depressive disorder. Neuropsychopharmacol.

[33] Mahableshwarkar A, Jacobsen P, Chen Y (2013). A randomized, double-blind trial of 2.5mg and 5mg vortioxetine versus placebo for 8 weeks in adults with major depressive disorder. Curr Med Res Opin.

[34] Nishimura A, Aritomi Y, Sasai K, Kitagawa T, Mahableshwarkar A (2018). Randomized, double-blind, placebo-controlled 8-week trialof the efficacy, safety, and tolerability of 5, 10, and 20 mg day vortioxetine in adults with major depressive disorder. Psychiatry and Clinical Neurosciences.

[35] NCT, 04448431. Comparison of vortioxetine and desvenlafaxine in adult patients suffering from depression. https://doi.org/https://ClinicalTrials.gov/show/NCT04448431. Accessed 2020.

[36] Feiger AD, Tourian K, Rosas GR, Padmanabhan S (2009). A placebo-controlled study evaluating the efficacy and safety of flexible-dose desvenlafaxine treatment in outpatients with major depressive disorder. CNS Spectrums.

[37] Septien-Velez L, Pitrosky B, Padmanabhan SK, Germain J-M, Tourian K. A randomized, double-blind, placebo-controlled trial of desvenlafaxine succinate in the treatment of major depressive disorder. Int Clin Psychopharmacol. 2007; 22(6). 10.1097/YIC.0b013e3281e2c84b 17917552

[38] Liebowitz M, Yeung P, Entsuah R (2007). A randomized, double-blind, placebo-controlled trial of desvenlafaxine succinate in adult outpatients with major depressive disorder. J Clin Psychiatry.

[39] Kornstein SG, Jiang Q, Reddy S, Musgnung J, Guico-Pabia CJ (2010). Short-term efficacy and safety of desvenlafaxine in a randomized, placebo-controlled study of perimenopausal and postmenopausal women with major depressive disorder. J Clin Psychiatry.

[40] Soares CN, Thase M, Clayton A, Guico-Pabia CJ, Focht K, Jiang Q, Kornstein SG, Ninan P, Kane CP, Cohen L (2010). Desvenlafaxine and escitalopram for the treatment of postmenopausal women with major depressive disorder. Menopause.

[41] Wang Z, Xu X, Tan Q, Li K, Ma C, Xie S, Gao C, Wang G, Li H (2015). Treatment of major depressive disorders with generic duloxetine and paroxetine: a multi-centered, double-blind, double-dummy, randomized controlled clinical trial. Shanghai archives of psychiatry.

[42] Khazaie H, Rezaie L, Rezaei Payam N, Najafi F (2015). Antidepressant-induced sexual dysfunction during treatment with fluoxetine, sertraline and trazodone; a randomized controlled trial. Gen Hosp Psychiatry.

[43] Khan A, Bose A, Alexopoulos GS, Gommoll C, Li D, Gandhi C. Clin Drug Investig. 2007; 27(7):481–92. 10.2165/00044011-200727070-00005. 10.2165/00044011-200727070-00005 17563128

[44] H. Lundbeck A/S. Efficacy of Vortioxetine on Cognitive Dysfunction in Working Patients With Major Depressive Disorder (Clinicaltrials.gov Identifier NCT02279966). 2017. https://ClinicalTrials.gov/show/NCT02279966.

[45] Rickels K, Amsterdam J, Clary C, Fox I, Schweizer E, Weise C. J Clin Psychiatry. 1992; 53 Suppl:30–2. 1531820

[46] Claghorn J (1992). The safety and efficacy of paroxetine compared with placebo in a double-blind trial of depressed outpatients. J Clin Psychiatry.

[47] Smith W, Glaudin V (1992). A placebo-controlled trial of paroxetine in the treatment of major depression. J Clin Psychiatry.

[48] Moher D, Liberati A, Tetzlaff J, Altman D (2009). The PG Preferred reporting items for systematic reviews and meta-analyses: The prisma statement. PLOS Med.

[49] Moncrieff J, Kirsch I (2005). Efficacy of antidepressants in adults. BMJ Clin Res ed.

[50] Altman D, Royston P (2006). The cost of dichotomising continuous variables. BMJ Clin Res ed.

[51] Hengartner M (2017). Methodological flaws, conflicts of interest, and scientific fallacies: Implications for the evaluation of antidepressants’ efficacy and harm. Frontiers in psychiatry.

[52] MacCallum R, Zhang S, Preacher KJ, Rucker D. Psychol Methods. 2002; 7(1):19–40. 10.1037/1082-989X.7.1.19. 10.1037/1082-989x.7.1.19 11928888

[53] Furukawa T, Cipriani A, Atkinson LZ, Leucht S, Ogawa Y, Takeshima N, Hayasaka Y, Chaimani A, Salanti G (2016). Placebo response rates in antidepressant trials: a systematic review of published and unpublished double-blind randomised controlled studies. Lancet Psychiatry.

[54] Nikolakopoulou A, Mavridis D, Salanti G (2016). Planning future studies based on the precision of network meta-analysis results. Stat Med.

[55] R Core Team (2017). R: A language and environment for statistical computing.

[56] Borenstein M, Hedges L, Higgins JPT, Rothstein HR. Introduction to Meta-Analysis.Wiley; 2009.

[57] Hengartner M, Plöderl M (2018). Statistically significant antidepressant-placebo differences on subjective symptom-rating scales do not prove that the drugs work: Effect size and method bias matter!. Front Psychiatry.

[58] Salanti G, Nikolakopoulou A. Actively Living Network Meta-Analysis, Working Paper, Institute of Social and Preventive Medicine (ISPM), University of Bern. 2021. https://www.ispm.unibe.ch/e93945/e93947/e451597/e488010/e488012/pane680818/e680822/EBAR_framework_description_paper_eng.pdf.

[59] Weber K, Lasch F, Koch A. Stat Med. 2018; 37(8):1402–4. 10.1002/sim.7595. 10.1002/sim.7595 PMC5873415 29529712

[60] The European Agency for the Evaluation of Medicinal Products. Application with 1. Meta-analyses; 2. One pivotal study, Reference number CPMP/EWP/2330/99. 2001. https://www.ema.europa.eu/en/application-1-metaanalyses-2-one-pivotal-study.

