# Supplementary material for: Conditional power of antidepressant network meta-analysis
Source: BMC Psychiatry. 2021 Mar 5;21:129. doi: 10.1186/s12888-021-03094-5 (PMC7934491; doi:10.1186/s12888-021-03094-5)
Supplement: Supplementary file 1 — Additional file 1 Supplement1 provides a flow chart and checklist according to the PRISMA statement, details on the results for individual treatment comparisons, and details on the sensitivity analyses. [file 12888_2021_3094_MOESM1_ESM.pdf]

Supplementary appendix 1: Conditional power of antidepressant network meta-analysis

Holper L, Department of Psychiatry, Psychotherapy, and Psychosomatics, University Hospital of Psychiatry, University of Zurich, Lenggstrasse 31, 8032 Zurich, Switzerland

Contents

1 Data sources **2**

2 Conditional power main analysis **7**

3 Conditional power sensitivity analysis **20**

4 List of included studies **35**

List of Figures

1a PRISMA flow diagram 2
1b Forest plot efficacy 4
1c Forest plot tolerability 5
1d Correlation between original and dummy effect sizes 6
2 Network graphs 7
3a Sensitivity analysis trial design: r 20
3b Sensitivity analysis effect size: f_{xy} 33
3c Sensitivity analysis event probability: pc 34

List of Tables

1 PRISMA checklist 3
2a Main analysis efficacy 8
2b Main analysis tolerability 14
3a Sensitivity analysis trial design efficacy 21
3b Sensitivity analysis trial design tolerability 27
4 List of included studies 35

1 Data sources

Figure 1a: PRISMA flow diagram. PRISMA (Preferred Reporting Items for Systematic Reviews and Meta-Analyses) flow diagram [1]. The analysis was based on a total of 535 RCTs (445 published studies, 94 unpublished studies) conducted between 1979 and 2018. 522 trials constituted the GRISELDA dataset [2] comparing 21 antidepressants provided by Cipriani et al. [3]. Additional 13 trials [4–16] were identified by own literature search. Efficacy (continuous) outcome was change on the Hamilton Depression Scale (HAMD) [17], for which information was available in 438 trials. Tolerability (binary) outcome was dropout rate due to adverse events, for which information was available in 438 trials. Response and remission rates were not assessed due to well-known methodological problems arising from dichotomization such as reduced statistical power (SP) and inflated effect sizes [18–21].

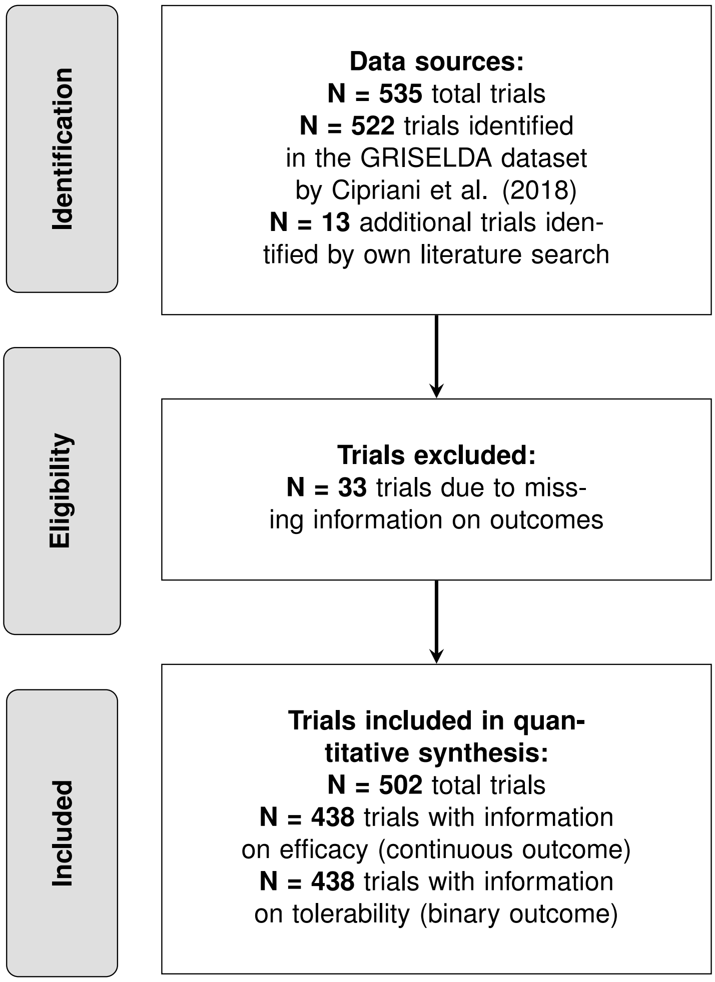

Table 1: PRISMA checklist. PRISMA (Preferred Reporting Items for Systematic Reviews and Meta-Analyses) checklist [1]. Risk of bias (RoB); not applicable (NA).

Section	Item	Checklist	Page
**Title**			
Title	1	Identify report as systematic review or meta-analysis, or both.	1
**Abstract**			
Structured summary	2	Provide structured summary including: background; objectives; data sources; study eligibility criteria; study appraisal and synthesis methods; results; limitations; conclusions; registration number.	1
**Introduction**			
Rationale	3	Describe rationale in the context of what is already known.	3
Objectives	4	Provide explicit statement of questions with reference to participants, interventions, comparisons, outcomes, and study design.	3
**Methods**			
Protocol and registration	5	Indicate if review protocol exists and where it can be accessed, provide registration information including registration number.	NA
Eligibility criteria	6	Specify study characteristics and report characteristics used as criteria for eligibility.	3
Information sources	7	Describe all information sources in the search and date last searched.	NA
Search	8	Present full electronic search strategy for at least one database, including any limits used, such that it could be repeated.	NA
Study selection	9	State the process for selecting studies.	S1
Data collection process	10	Describe method of data extraction from reports and any processes for obtaining and confirming data from investigators.	NA
Data items	11	List and define all variables for which data were sought and any assumptions and simplifications made.	3
Risk of bias individual studies	12	Describe methods for assessing risk of bias of individual studies and how this information is to be used in any data synthesis.	NA
Summary measures	13	State the principal summary measures.	4
Synthesis of results	14	Describe methods of handling data and combining results of studies, if done, including measures of consistency for each meta-analysis.	4
Risk of bias across studies	15	Specify any assessment of risk of bias that may affect the cumulative evidence.	NA
Additional analyses	16	Describe methods of additional analyses, if done, indicating which were pre-specified.	4
**Results**			
Study selection	17	Give numbers of studies screened, assessed for eligibility, and included in the review, with reasons for exclusions at each stage.	S1
Study characteristics	18	For each study, present characteristics for which data were extracted and provide the citations.	NA
Risk of bias within studies	19	Present data on risk of bias of each study and, if available, any outcome level assessment (see item 12).	NA
Results individual studies	20	For all outcomes, present, for each study: (a) simple summary data for each intervention group (b) effect estimates and confidence intervals.	S1
Synthesis of results	21	Present main results of the review. If meta-analyses are done, include for each, confidence intervals and measures of consistency.	5
Risk of bias across studies	22	Present results of any assessment of risk of bias across studies (see Item 15).	NA
Additional analysis	23	Give results of additional analyses (see item 16).	S1
**Discussion**			
Summary of evidence	24	Summarize main findings including the strength of evidence for each outcome; consider their relevance to key groups.	6
Limitations	25	Discuss limitations at study and outcome level and at review-level.	7
Conclusions	26	Provide a general interpretation of the results in the context of other evidence, and implications for future research.	7
**Funding**			
Funding	27	Describe sources of funding and other support.	7

Figure 1b: Forest plot efficacy. Forest plots illustrating drug-placebo effects for efficacy on the standardized mean difference (SMD) scale. Square size is proportionate to actual sample size.

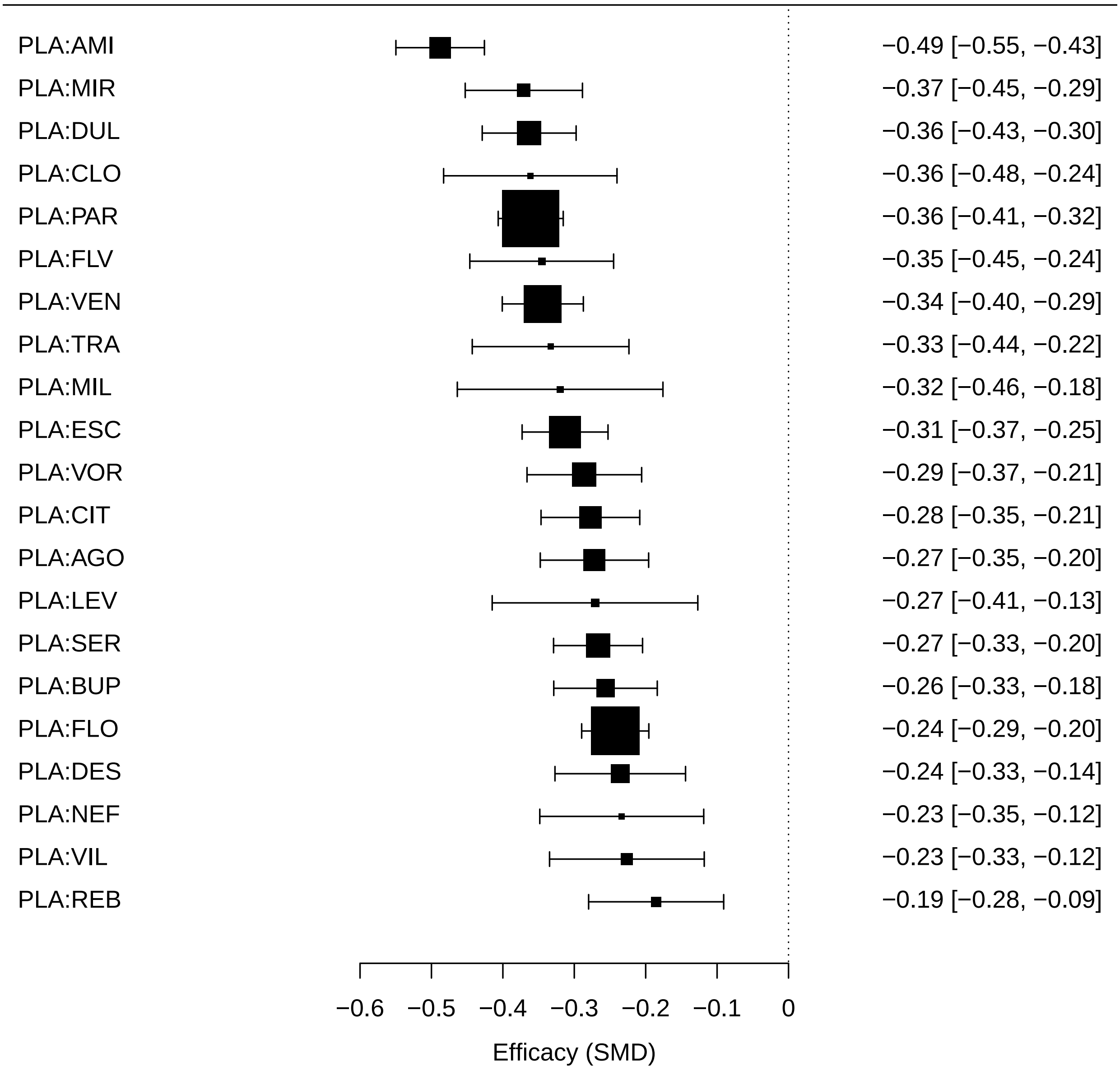

Figure 1c: Forest plot tolerability. Forest plots illustrating drug-placebo effects for tolerability on the odds ratio (OR) scale. Square size is proportionate to actual sample size.

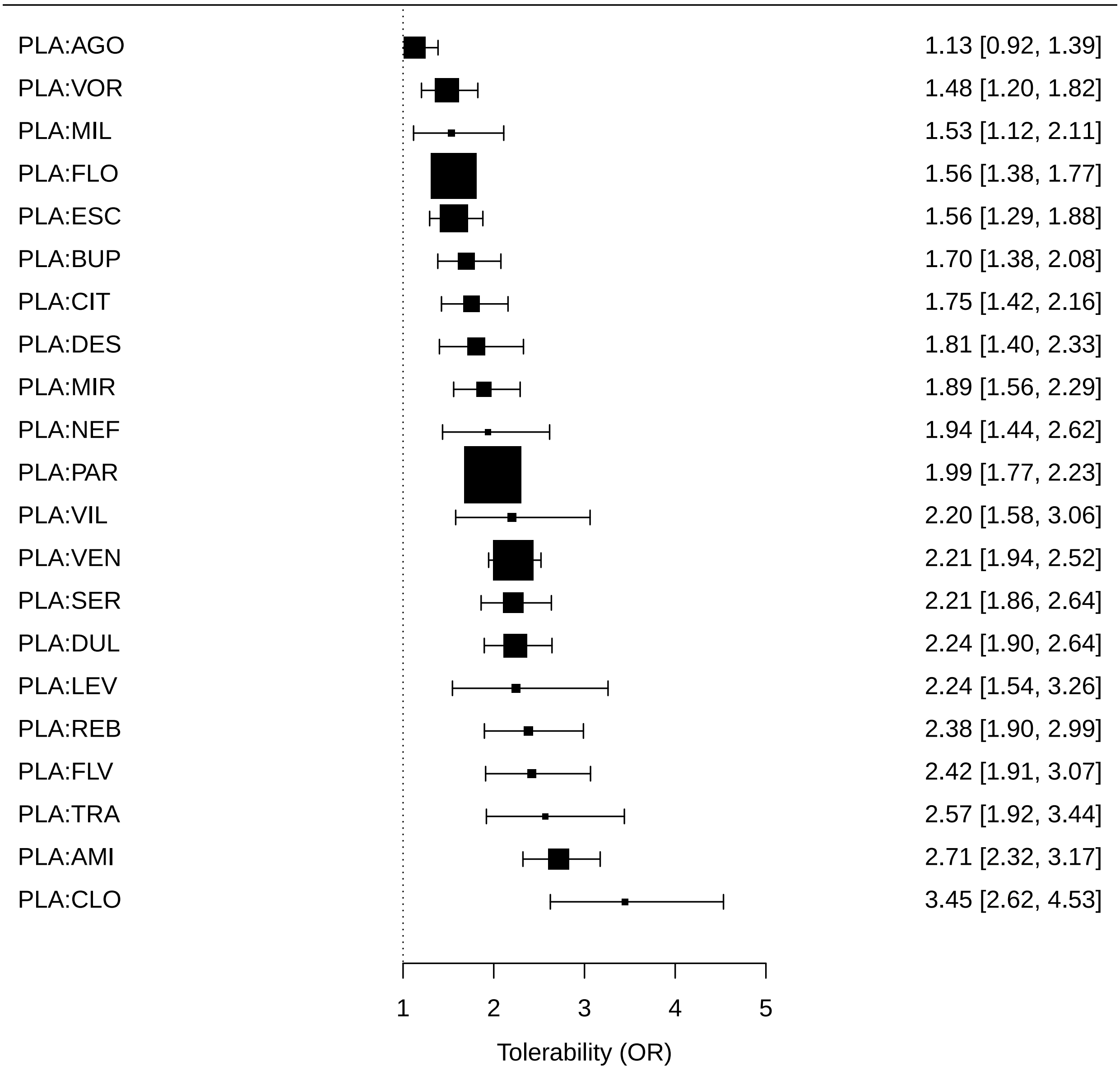

Figure 1d: Correlation between original and dummy effect sizes. Scatter plot illustrating the correlation between original and dummy effect sizes, for efficacy estimated on the standardized mean difference (SMD) scale (Pearson r = 0.999), and for tolerability estimated on the odds ratio (OR) scale (Pearson r = 0.995). Dummy effect sizes were used to estimate conditional power in both direct and not-direct observed comparisons; on the latter conditional power could otherwise not be assessed.

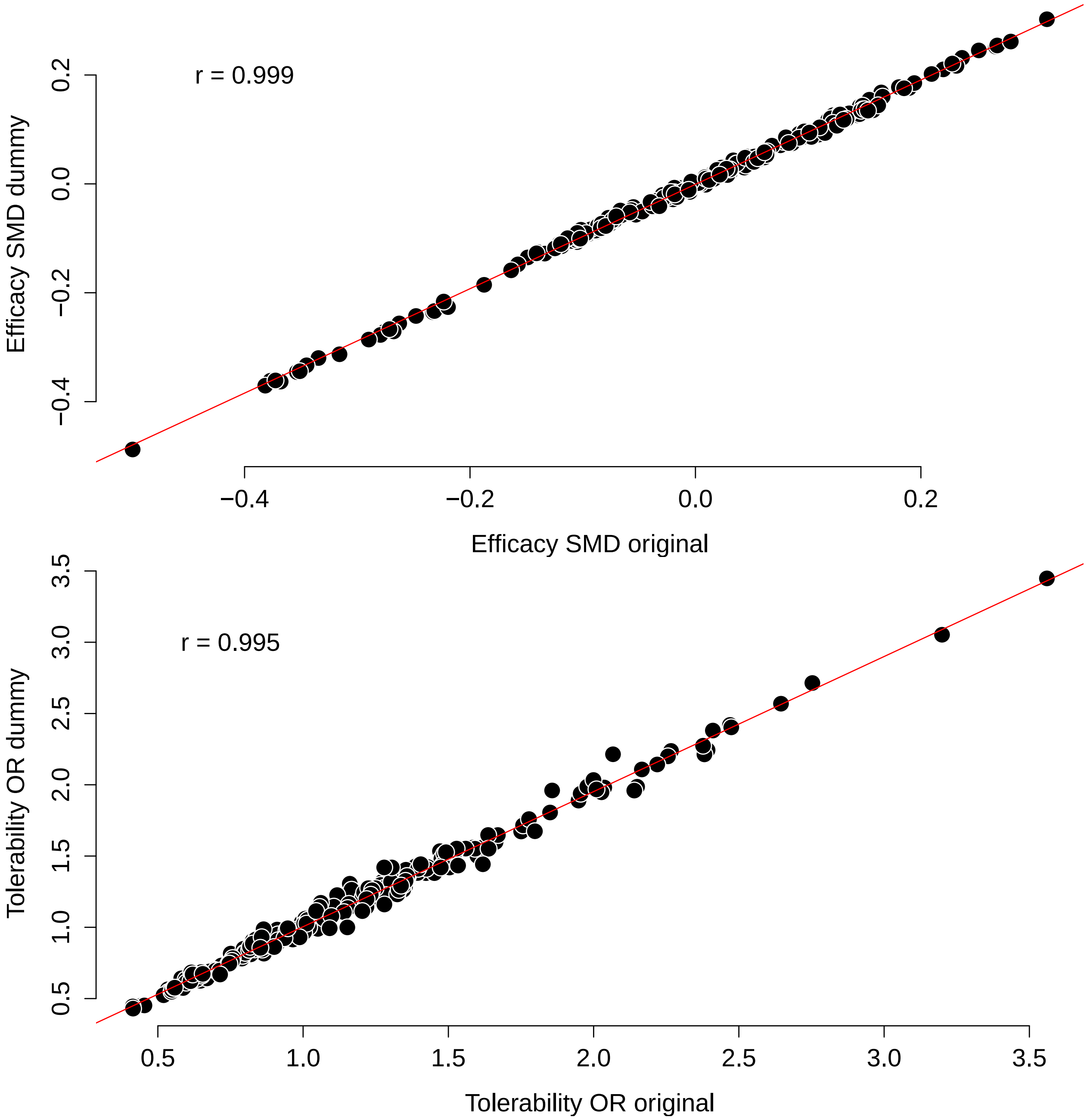

2 Conditional power main analysis

Figure 2: Network graphs. Illustration of treatment eligible comparisons in the original network and separately for comparisons with conclusive versus inconclusive evidence. Circle size is proportionate to actual sample size. For comparisons with conclusive evidence, line width is set to 1. For comparisons with inconclusive evidence, line width is proportionate to the sample size at 80% conditional power ($N_{CP=80\%}$).

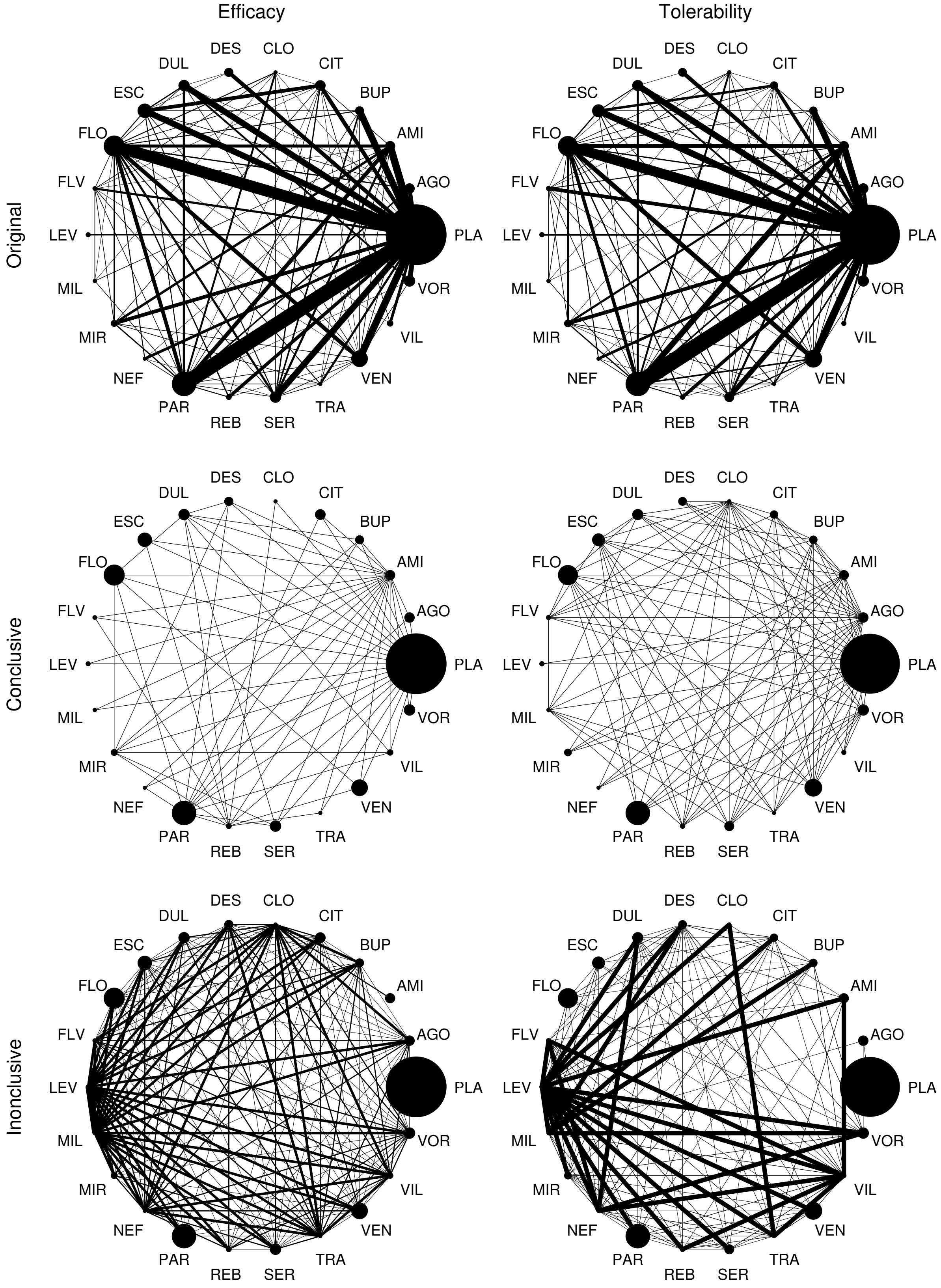

Table 2a: Main analysis efficacy. Listed are network characteristics for each of the 231 comparisons regarding the conclusiveness of evidence (conclusive or inconclusive), the number of trials, sample size, study year range of completion (only for direct comparisons), and network effect estimates with 95% confidence intervals on the standardized mean difference (SMD [95%CI]) scale. Listed are results of the conditional power analysis in terms of the sample size at 20% and 80% conditional power ($N_{CP=20\%}$, $N_{CP=80\%}$). Results are shown based on the main analysis considering a trial design ratio of r = 1/0, anticipated alternative effect sizes equal to the network estimates (f_{xyN}), and anticipated event probabilities equal to the average network event probability (pc_N).

	Network					Conditional power	
	Evidence	Trials	Sample	Year range	SMD [95%CI]	$N_{CP=20\%}$	$N_{CP=80\%}$
PLA:AGO	conclusive	13	4173	1999-2011	-0.27 [-0.35–0.20]	-	-
PLA:AMI	conclusive	24	3055	1977-1995	-0.49 [-0.55–0.43]	-	-
PLA:BUP	conclusive	22	5268	1980-2012	-0.26 [-0.33–0.18]	-	-
PLA:CIT	conclusive	13	3786	1992-2013	-0.28 [-0.35–0.21]	-	-
PLA:CLO	conclusive	1	38	1987-1987	-0.36 [-0.48–0.24]	-	-
PLA:DES	conclusive	13	5604	2004-2012	-0.24 [-0.33–0.14]	-	-
PLA:DUL	conclusive	20	5587	2000-2014	-0.36 [-0.43–0.30]	-	-
PLA:ESC	conclusive	20	6223	2000-2010	-0.31 [-0.37–0.25]	-	-
PLA:FLO	conclusive	38	8460	1983-2014	-0.24 [-0.29–0.20]	-	-
PLA:FLV	conclusive	7	991	1982-1994	-0.35 [-0.45–0.24]	-	-
PLA:LEV	conclusive	5	2654	2007-2012	-0.27 [-0.41–0.13]	-	-
PLA:MIL	conclusive	-	-	-	-0.32 [-0.46–0.18]	-	-
PLA:MIR	conclusive	12	1465	1985-2007	-0.37 [-0.45–0.29]	-	-
PLA:NEF	conclusive	9	1362	1990-1996	-0.23 [-0.35–0.12]	-	-
PLA:PAR	conclusive	48	9726	1984-2014	-0.36 [-0.41–0.32]	-	-
PLA:REB	conclusive	11	2612	1991-2001	-0.19 [-0.28–0.09]	-	-
PLA:SER	conclusive	20	4403	1989-2013	-0.27 [-0.33–0.20]	-	-
PLA:TRA	conclusive	7	1230	1978-2013	-0.33 [-0.44–0.22]	-	-
PLA:VEN	conclusive	23	5724	1991-2014	-0.34 [-0.40–0.29]	-	-
PLA:VIL	conclusive	9	3978	2007-2013	-0.23 [-0.33–0.12]	-	-
PLA:VOR	conclusive	16	6908	2007-2017	-0.29 [-0.37–0.21]	-	-
AGO:AMI	conclusive	-	-	-	-0.22 [-0.31–0.12]	-	-
AGO:BUP	inconclusive	-	-	-	0.02 [-0.09- 0.12]	343	2176
AGO:CIT	inconclusive	-	-	-	-0.01 [-0.11- 0.09]	351	2252
AGO:CLO	inconclusive	-	-	-	-0.09 [-0.23- 0.05]	153	1304
AGO:DES	inconclusive	-	-	-	0.04 [-0.08- 0.15]	300	1801
AGO:DUL	inconclusive	1	418	2008-2008	-0.09 [-0.19- 0.00]	49	2142
AGO:ESC	inconclusive	2	462	2006-2008	-0.04 [-0.13- 0.05]	322	2475
AGO:FLO	inconclusive	4	1862	1999-2012	0.03 [-0.05- 0.11]	381	2942
AGO:FLV	inconclusive	-	-	-	-0.07 [-0.20- 0.05]	194	1553
AGO:LEV	inconclusive	-	-	-	0.00 [-0.16- 0.16]	252	1175
AGO:MIL	inconclusive	-	-	-	-0.05 [-0.21- 0.11]	229	1156

AGO:MIR	inconclusive	-	-	-	-0.10 [-0.21- 0.01]	66	1799
AGO:NEF	inconclusive	-	-	-	0.04 [-0.10- 0.17]	275	1480
AGO:PAR	conclusive	5	1648	1999-2008	-0.09 [-0.17–0.01]	-	-
AGO:REB	inconclusive	-	-	-	0.09 [-0.03- 0.21]	156	1797
AGO:SER	inconclusive	-	-	-	0.01 [-0.09- 0.10]	368	2412
AGO:TRA	inconclusive	-	-	-	-0.06 [-0.19- 0.07]	222	1450
AGO:VEN	inconclusive	2	609	2002-2002	-0.07 [-0.16- 0.02]	130	2494
AGO:VIL	inconclusive	-	-	-	0.05 [-0.09- 0.18]	274	1555
AGO:VOR	inconclusive	-	-	-	-0.01 [-0.12- 0.09]	326	1986
AMI:BUP	conclusive	-	-	-	0.23 [0.14- 0.32]	-	-
AMI:CIT	conclusive	2	409	1984-1996	0.21 [0.12- 0.30]	-	-
AMI:CLO	inconclusive	-	-	-	0.13 [-0.01- 0.26]	58	1576
AMI:DES	conclusive	-	-	-	0.25 [0.14- 0.36]	-	-
AMI:DUL	conclusive	-	-	-	0.12 [0.04- 0.21]	-	-
AMI:ESC	conclusive	-	-	-	0.17 [0.09- 0.26]	-	-
AMI:FLO	conclusive	10	796	1983-1997	0.25 [0.18- 0.32]	-	-
AMI:FLV	conclusive	1	235	1996-1996	0.14 [0.03- 0.25]	-	-
AMI:LEV	conclusive	-	-	-	0.22 [0.06- 0.37]	-	-
AMI:MIL	conclusive	2	231	1987-1987	0.17 [0.02- 0.32]	-	-
AMI:MIR	conclusive	8	895	1988-1995	0.12 [0.02- 0.21]	-	-
AMI:NEF	conclusive	1	106	1992-1992	0.25 [0.13- 0.38]	-	-
AMI:PAR	conclusive	13	1813	1985-2000	0.13 [0.06- 0.19]	-	-
AMI:REB	conclusive	-	-	-	0.30 [0.19- 0.41]	-	-
AMI:SER	conclusive	7	1432	1988-1998	0.22 [0.14- 0.30]	-	-
AMI:TRA	conclusive	2	207	1980-1987	0.15 [0.03- 0.28]	-	-
AMI:VEN	conclusive	2	272	1998-2001	0.14 [0.06- 0.22]	-	-
AMI:VIL	conclusive	-	-	-	0.26 [0.14- 0.39]	-	-
AMI:VOR	conclusive	-	-	-	0.20 [0.10- 0.30]	-	-
BUP:CIT	inconclusive	-	-	-	-0.02 [-0.12- 0.08]	346	2302
BUP:CLO	inconclusive	-	-	-	-0.11 [-0.24- 0.03]	109	1295
BUP:DES	inconclusive	-	-	-	0.02 [-0.10- 0.14]	313	1834
BUP:DUL	conclusive	-	-	-	-0.11 [-0.20–0.01]	-	-
BUP:ESC	inconclusive	2	566	2004-2004	-0.06 [-0.15- 0.03]	254	2486
BUP:FLO	inconclusive	3	740	1989-2000	0.01 [-0.07- 0.10]	406	2963
BUP:FLV	inconclusive	-	-	-	-0.09 [-0.21- 0.03]	131	1555
BUP:LEV	inconclusive	-	-	-	-0.01 [-0.18- 0.15]	250	1176
BUP:MIL	inconclusive	-	-	-	-0.06 [-0.22- 0.10]	213	1147
BUP:MIR	conclusive	-	-	-	-0.11 [-0.22–0.01]	-	-
BUP:NEF	inconclusive	-	-	-	0.02 [-0.11- 0.16]	284	1493
BUP:PAR	conclusive	2	218	2001-2009	-0.10 [-0.19–0.02]	-	-
BUP:REB	inconclusive	-	-	-	0.07 [-0.05- 0.19]	224	1824
BUP:SER	inconclusive	5	991	1995-2001	-0.01 [-0.10- 0.08]	385	2648

BUP:TRA	inconclusive	1	124	1992-1992	-0.08 [-0.20- 0.05]	187	1493
BUP:VEN	inconclusive	2	725	2004-2005	-0.09 [-0.18- 0.00]	54	2499
BUP:VIL	inconclusive	-	-	-	0.03 [-0.10- 0.16]	287	1574
BUP:VOR	inconclusive	-	-	-	-0.03 [-0.14- 0.08]	318	2018
CIT:CLO	inconclusive	1	114	1984-1984	-0.08 [-0.22- 0.05]	131	1349
CIT:DES	inconclusive	-	-	-	0.04 [-0.07- 0.16]	301	1911
CIT:DUL	inconclusive	-	-	-	-0.09 [-0.18- 0.01]	59	2319
CIT:ESC	inconclusive	12	2400	2000-2011	-0.04 [-0.12- 0.04]	341	3034
CIT:FLO	inconclusive	2	673	1994-1995	0.03 [-0.04- 0.11]	375	3208
CIT:FLV	inconclusive	1	217	1991-1991	-0.07 [-0.19- 0.05]	225	1706
CIT:LEV	inconclusive	-	-	-	0.01 [-0.15- 0.17]	256	1205
CIT:MIL	inconclusive	-	-	-	-0.04 [-0.20- 0.11]	237	1189
CIT:MIR	inconclusive	2	322	1997-2013	-0.09 [-0.20- 0.01]	56	1965
CIT:NEF	inconclusive	-	-	-	0.04 [-0.09- 0.18]	275	1539
CIT:PAR	conclusive	1	310	2000-2000	-0.08 [-0.16–0.01]	-	-
CIT:REB	inconclusive	2	460	2004-2004	0.09 [-0.02- 0.20]	88	1942
CIT:SER	inconclusive	4	811	1995-2009	0.01 [-0.08- 0.10]	400	2772
CIT:TRA	inconclusive	-	-	-	-0.06 [-0.18- 0.07]	237	1521
CIT:VEN	inconclusive	1	151	2002-2002	-0.07 [-0.15- 0.02]	157	2679
CIT:VIL	inconclusive	2	1232	2010-2013	0.05 [-0.07- 0.17]	277	1713
CIT:VOR	inconclusive	-	-	-	-0.01 [-0.11- 0.10]	338	2108
CLO:DES	inconclusive	-	-	-	0.13 [-0.03- 0.28]	86	1337
CLO:DUL	inconclusive	-	-	-	-0.00 [-0.14- 0.13]	285	1462
CLO:ESC	inconclusive	-	-	-	0.05 [-0.08- 0.18]	269	1543
CLO:FLO	inconclusive	3	204	1987-1989	0.12 [-0.01- 0.24]	62	1708
CLO:FLV	inconclusive	2	83	1981-1993	0.02 [-0.13- 0.17]	265	1301
CLO:LEV	inconclusive	-	-	-	0.09 [-0.10- 0.28]	186	1051
CLO:MIL	inconclusive	1	107	1995-1995	0.04 [-0.13- 0.22]	205	1070
CLO:MIR	inconclusive	-	-	-	-0.01 [-0.15- 0.13]	274	1364
CLO:NEF	inconclusive	-	-	-	0.13 [-0.04- 0.29]	102	1218
CLO:PAR	inconclusive	6	1455	1987-1995	0.00 [-0.12- 0.12]	308	1716
CLO:REB	conclusive	-	-	-	0.18 [0.03- 0.33]	-	-
CLO:SER	inconclusive	2	272	1992-1998	0.09 [-0.03- 0.22]	156	1616
CLO:TRA	inconclusive	-	-	-	0.03 [-0.13- 0.19]	254	1211
CLO:VEN	inconclusive	1	102	1996-1996	0.02 [-0.11- 0.15]	293	1567
CLO:VIL	inconclusive	-	-	-	0.14 [-0.03- 0.30]	83	1247
CLO:VOR	inconclusive	-	-	-	0.08 [-0.07- 0.22]	210	1366
DES:DUL	conclusive	1	474	2007-2007	-0.13 [-0.24–0.02]	-	-
DES:ESC	inconclusive	1	388	2008-2008	-0.08 [-0.18- 0.03]	136	1905
DES:FLO	inconclusive	-	-	-	-0.01 [-0.11- 0.10]	345	2186
DES:FLV	inconclusive	-	-	-	-0.11 [-0.25- 0.03]	89	1330
DES:LEV	inconclusive	-	-	-	-0.04 [-0.21- 0.13]	229	1089

DES:MIL	inconclusive	-	-	-	-0.08 [-0.25- 0.09]	175	1051
DES:MIR	conclusive	-	-	-	-0.14 [-0.26–0.01]	-	-
DES:NEF	inconclusive	-	-	-	0.00 [-0.14- 0.15]	271	1327
DES:PAR	conclusive	-	-	-	-0.13 [-0.23–0.02]	-	-
DES:REB	inconclusive	-	-	-	0.05 [-0.08- 0.18]	269	1565
DES:SER	inconclusive	-	-	-	-0.03 [-0.14- 0.08]	305	1925
DES:TRA	inconclusive	-	-	-	-0.10 [-0.24- 0.04]	123	1268
DES:VEN	inconclusive	-	-	-	-0.11 [-0.22–0.00]	49	1826
DES:VIL	inconclusive	-	-	-	0.01 [-0.13- 0.15]	278	1391
DES:VOR	inconclusive	-	-	-	-0.05 [-0.17- 0.07]	268	1670
DUL:ESC	inconclusive	4	1382	2005-2006	0.05 [-0.03- 0.13]	316	2987
DUL:FLO	conclusive	2	222	2000-2001	0.12 [0.04- 0.20]	-	-
DUL:FLV	inconclusive	-	-	-	0.02 [-0.10- 0.14]	310	1782
DUL:LEV	inconclusive	-	-	-	0.09 [-0.07- 0.25]	180	1263
DUL:MIL	inconclusive	-	-	-	0.04 [-0.11- 0.20]	249	1258
DUL:MIR	inconclusive	-	-	-	-0.01 [-0.11- 0.10]	343	2164
DUL:NEF	inconclusive	-	-	-	0.13 [-0.00- 0.26]	50	1577
DUL:PAR	inconclusive	8	2448	2001-2012	0.00 [-0.07- 0.08]	471	3546
DUL:REB	conclusive	-	-	-	0.18 [0.06- 0.29]	-	-
DUL:SER	conclusive	-	-	-	0.10 [0.01- 0.18]	-	-
DUL:TRA	inconclusive	-	-	-	0.03 [-0.10- 0.16]	294	1635
DUL:VEN	inconclusive	2	836	2006-2006	0.02 [-0.06- 0.10]	407	2994
DUL:VIL	conclusive	-	-	-	0.14 [0.01- 0.26]	-	-
DUL:VOR	inconclusive	6	2392	2008-2014	0.08 [-0.02- 0.17]	101	2425
ESC:FLO	inconclusive	3	783	2003-2006	0.07 [-0.00- 0.14]	52	3750
ESC:FLV	inconclusive	-	-	-	-0.03 [-0.15- 0.08]	298	1805
ESC:LEV	inconclusive	-	-	-	0.04 [-0.11- 0.20]	251	1259
ESC:MIL	inconclusive	-	-	-	-0.01 [-0.16- 0.15]	260	1245
ESC:MIR	inconclusive	-	-	-	-0.06 [-0.16- 0.04]	252	2197
ESC:NEF	inconclusive	-	-	-	0.08 [-0.05- 0.21]	201	1622
ESC:PAR	inconclusive	3	1145	2004-2009	-0.05 [-0.12- 0.02]	283	3689
ESC:REB	conclusive	-	-	-	0.13 [0.02- 0.24]	-	-
ESC:SER	inconclusive	3	629	2002-2005	0.05 [-0.04- 0.13]	323	3019
ESC:TRA	inconclusive	-	-	-	-0.02 [-0.14- 0.10]	293	1642
ESC:VEN	inconclusive	2	495	2002-2002	-0.03 [-0.11- 0.05]	386	3148
ESC:VIL	inconclusive	-	-	-	0.09 [-0.04- 0.21]	158	1731
ESC:VOR	inconclusive	-	-	-	0.03 [-0.07- 0.13]	341	2314
FLO:FLV	inconclusive	2	284	1994-2001	-0.10 [-0.21- 0.00]	51	1853
FLO:LEV	inconclusive	-	-	-	-0.03 [-0.18- 0.12]	254	1253
FLO:MIL	inconclusive	1	300	1996-1996	-0.08 [-0.22- 0.07]	180	1245
FLO:MIR	conclusive	4	579	1996-2003	-0.13 [-0.22–0.04]	-	-
FLO:NEF	inconclusive	3	161	1995-1995	0.01 [-0.11- 0.13]	308	1722

FLO:PAR	conclusive	12	2754	1991-2000	-0.12 [-0.18–0.06]	-	-
FLO:REB	inconclusive	4	806	1995-2001	0.06 [-0.04- 0.16]	280	2290
FLO:SER	inconclusive	7	1328	1990-2010	-0.02 [-0.09- 0.05]	414	3711
FLO:TRA	inconclusive	3	284	1987-2010	-0.09 [-0.21- 0.02]	90	1689
FLO:VEN	conclusive	13	3253	1992-2013	-0.10 [-0.17–0.04]	-	-
FLO:VIL	inconclusive	1	196	2010-2010	0.02 [-0.10- 0.13]	314	1826
FLO:VOR	inconclusive	-	-	-	-0.04 [-0.13- 0.05]	318	2510
FLV:LEV	inconclusive	-	-	-	0.07 [-0.10- 0.25]	209	1122
FLV:MIL	inconclusive	2	239	1989-1999	0.03 [-0.14- 0.19]	254	1198
FLV:MIR	inconclusive	2	412	2000-2000	-0.03 [-0.15- 0.10]	295	1689
FLV:NEF	inconclusive	-	-	-	0.11 [-0.04- 0.26]	120	1337
FLV:PAR	inconclusive	2	180	1992-1995	-0.02 [-0.12- 0.09]	332	2078
FLV:REB	conclusive	-	-	-	0.16 [0.02- 0.30]	-	-
FLV:SER	inconclusive	2	185	1993-2003	0.08 [-0.03- 0.19]	170	1919
FLV:TRA	inconclusive	-	-	-	0.01 [-0.13- 0.16]	273	1342
FLV:VEN	inconclusive	1	111	1996-1996	0.00 [-0.11- 0.11]	325	1926
FLV:VIL	inconclusive	-	-	-	0.12 [-0.03- 0.27]	93	1383
FLV:VOR	inconclusive	-	-	-	0.06 [-0.07- 0.19]	250	1595
LEV:MIL	inconclusive	-	-	-	-0.05 [-0.25- 0.15]	188	894
LEV:MIR	inconclusive	-	-	-	-0.10 [-0.26- 0.07]	147	1069
LEV:NEF	inconclusive	-	-	-	0.04 [-0.15- 0.22]	226	1048
LEV:PAR	inconclusive	-	-	-	-0.09 [-0.24- 0.06]	163	1197
LEV:REB	inconclusive	-	-	-	0.09 [-0.09- 0.26]	197	1150
LEV:SER	inconclusive	-	-	-	0.00 [-0.15- 0.16]	259	1228
LEV:TRA	inconclusive	-	-	-	-0.06 [-0.24- 0.12]	194	1001
LEV:VEN	inconclusive	-	-	-	-0.07 [-0.23- 0.08]	201	1180
LEV:VIL	inconclusive	-	-	-	0.04 [-0.14- 0.22]	227	1076
LEV:VOR	inconclusive	-	-	-	-0.01 [-0.18- 0.15]	246	1151
MIL:MIR	inconclusive	-	-	-	-0.05 [-0.21- 0.11]	225	1146
MIL:NEF	inconclusive	-	-	-	0.09 [-0.10- 0.27]	193	1083
MIL:PAR	inconclusive	2	1207	2002-2011	-0.04 [-0.18- 0.10]	250	1319
MIL:REB	inconclusive	-	-	-	0.13 [-0.04- 0.30]	97	1183
MIL:SER	inconclusive	-	-	-	0.05 [-0.10- 0.21]	248	1291
MIL:TRA	inconclusive	-	-	-	-0.01 [-0.19- 0.16]	230	1054
MIL:VEN	inconclusive	-	-	-	-0.02 [-0.18- 0.13]	256	1249
MIL:VIL	inconclusive	-	-	-	0.09 [-0.09- 0.27]	184	1104
MIL:VOR	inconclusive	-	-	-	0.03 [-0.13- 0.20]	244	1173
MIR:NEF	inconclusive	-	-	-	0.14 [-0.00- 0.28]	53	1470
MIR:PAR	inconclusive	4	719	1996-2007	0.01 [-0.08- 0.10]	397	2739
MIR:REB	conclusive	-	-	-	0.19 [0.06- 0.31]	-	-
MIR:SER	conclusive	-	-	-	0.10 [0.01- 0.20]	-	-
MIR:TRA	inconclusive	2	300	1993-1995	0.04 [-0.09- 0.17]	294	1594

MIR:VEN	inconclusive	2	533	2004-2004	0.03 [-0.07- 0.12]	355	2487
MIR:VIL	conclusive	-	-	-	0.14 [0.01- 0.28]	-	-
MIR:VOR	inconclusive	-	-	-	0.08 [-0.03- 0.20]	135	1871
NEF:PAR	conclusive	2	246	1994-2000	-0.13 [-0.25–0.01]	-	-
NEF:REB	inconclusive	-	-	-	0.05 [-0.10- 0.20]	255	1347
NEF:SER	inconclusive	1	160	1994-1994	-0.03 [-0.16- 0.09]	278	1557
NEF:TRA	inconclusive	-	-	-	-0.10 [-0.26- 0.06]	134	1129
NEF:VEN	inconclusive	-	-	-	-0.11 [-0.24- 0.02]	76	1447
NEF:VIL	inconclusive	-	-	-	0.01 [-0.15- 0.16]	258	1223
NEF:VOR	inconclusive	-	-	-	-0.05 [-0.19- 0.09]	247	1378
PAR:REB	conclusive	3	1375	2000-2004	0.18 [0.08- 0.28]	-	-
PAR:SER	conclusive	2	545	1998-2000	0.09 [0.02- 0.17]	-	-
PAR:TRA	inconclusive	2	333	1998-2003	0.03 [-0.09- 0.14]	312	1882
PAR:VEN	inconclusive	2	607	1995-1996	0.02 [-0.05- 0.08]	485	4190
PAR:VIL	conclusive	-	-	-	0.13 [0.02- 0.25]	-	-
PAR:VOR	inconclusive	1	88	2014-2014	0.08 [-0.02- 0.17]	106	2579
REB:SER	inconclusive	-	-	-	-0.08 [-0.19- 0.03]	128	1801
REB:TRA	conclusive	-	-	-	-0.15 [-0.29–0.00]	-	-
REB:VEN	conclusive	1	167	2000-2000	-0.16 [-0.27–0.05]	-	-
REB:VIL	inconclusive	-	-	-	-0.04 [-0.18- 0.10]	254	1330
REB:VOR	inconclusive	-	-	-	-0.10 [-0.22- 0.02]	96	1567
SER:TRA	inconclusive	2	250	2004-2010	-0.07 [-0.19- 0.05]	204	1609
SER:VEN	inconclusive	2	323	2003-2004	-0.08 [-0.16- 0.00]	66	2940
SER:VIL	inconclusive	-	-	-	0.04 [-0.08- 0.16]	285	1686
SER:VOR	inconclusive	-	-	-	-0.02 [-0.12- 0.08]	344	2230
TRA:VEN	inconclusive	1	153	1994-1994	-0.01 [-0.13- 0.11]	307	1718
TRA:VIL	inconclusive	-	-	-	0.11 [-0.05- 0.26]	127	1310
TRA:VOR	inconclusive	-	-	-	0.05 [-0.09- 0.18]	258	1480
VEN:VIL	inconclusive	-	-	-	0.12 [-0.00- 0.24]	56	1746
VEN:VOR	inconclusive	2	767	2007-2013	0.06 [-0.04- 0.15]	274	2456
VIL:VOR	inconclusive	-	-	-	-0.06 [-0.19- 0.07]	240	1438

Table 2b: Main analysis tolerability. Listed are network characteristics for each of the 231 comparisons regarding the conclusiveness of evidence (conclusive or inconclusive), the number of trials, sample size, study year range of completion (only for direct comparisons), and network effect estimates with 95% confidence intervals on the odds ratio (OR [95%CI]) scale. Listed are results of the conditional power analysis in terms of the sample size at 20% and 80% conditional power ($N_{CP=20\%}$, $N_{CP=80\%}$). Results are shown based on the main analysis considering a trial design ratio of r = 1/0, anticipated alternative effect sizes equal to the network estimates (f_{xyN}), and anticipated event probabilities equal to the average network event probability (pc_N).

	Network					Conditional power	
	Evidence	Trials	Sample	Year range	OR [95%CI]	$N_{CP=20\%}$	$N_{CP=80\%}$
PLA:AGO	inconclusive	13	4173	1999-2011	1.13 [0.92-1.39]	264	1065
PLA:AMI	conclusive	22	2473	1977-1995	2.71 [2.32-3.17]	-	-
PLA:BUP	conclusive	21	5220	1980-2012	1.70 [1.38-2.08]	-	-
PLA:CIT	conclusive	11	3266	1992-2013	1.75 [1.42-2.16]	-	-
PLA:CLO	conclusive	1	38	1987-1987	3.45 [2.62-4.53]	-	-
PLA:DES	conclusive	13	5604	2004-2012	1.81 [1.40-2.33]	-	-
PLA:DUL	conclusive	20	5800	2000-2014	2.24 [1.90-2.64]	-	-
PLA:ESC	conclusive	21	6432	2000-2010	1.56 [1.29-1.88]	-	-
PLA:FLO	conclusive	33	7724	1983-2014	1.56 [1.38-1.77]	-	-
PLA:FLV	conclusive	12	1408	1981-1994	2.42 [1.91-3.07]	-	-
PLA:LEV	conclusive	6	2837	2007-2012	2.24 [1.54-3.26]	-	-
PLA:MIL	conclusive	-	-	-	1.53 [1.12-2.11]	-	-
PLA:MIR	conclusive	12	1717	1985-2007	1.89 [1.56-2.29]	-	-
PLA:NEF	conclusive	9	1362	1990-1996	1.94 [1.44-2.62]	-	-
PLA:PAR	conclusive	52	10405	1984-2010	1.99 [1.77-2.23]	-	-
PLA:REB	conclusive	11	2612	1991-2001	2.38 [1.90-2.99]	-	-
PLA:SER	conclusive	16	3834	1989-2013	2.21 [1.86-2.64]	-	-
PLA:TRA	conclusive	5	1065	1977-2013	2.57 [1.92-3.44]	-	-
PLA:VEN	conclusive	24	6083	1991-2014	2.21 [1.94-2.52]	-	-
PLA:VIL	conclusive	6	2895	2007-2013	2.20 [1.58-3.06]	-	-
PLA:VOR	conclusive	15	6820	2007-2017	1.48 [1.20-1.82]	-	-
AGO:AMI	conclusive	-	-	-	2.40 [1.88-3.07]	-	-
AGO:BUP	conclusive	-	-	-	1.50 [1.13-1.99]	-	-
AGO:CIT	conclusive	-	-	-	1.55 [1.17-2.06]	-	-
AGO:CLO	conclusive	-	-	-	3.05 [2.20-4.24]	-	-
AGO:DES	conclusive	-	-	-	1.60 [1.16-2.21]	-	-
AGO:DUL	conclusive	1	418	2008-2008	1.98 [1.55-2.54]	-	-
AGO:ESC	conclusive	2	462	2006-2008	1.38 [1.06-1.80]	-	-
AGO:FLO	conclusive	4	1862	1999-2012	1.38 [1.11-1.71]	-	-
AGO:FLV	conclusive	-	-	-	2.14 [1.58-2.91]	-	-
AGO:LEV	conclusive	-	-	-	1.99 [1.30-3.04]	-	-
AGO:MIL	inconclusive	-	-	-	1.36 [0.94-1.96]	97	805

AGO:MIR	conclusive	-	-	-	1.67 [1.28-2.19]	-	-
AGO:NEF	conclusive	-	-	-	1.72 [1.20-2.45]	-	-
AGO:PAR	conclusive	5	1648	1999-2008	1.76 [1.42-2.18]	-	-
AGO:REB	conclusive	-	-	-	2.11 [1.57-2.83]	-	-
AGO:SER	conclusive	-	-	-	1.96 [1.51-2.54]	-	-
AGO:TRA	conclusive	-	-	-	2.27 [1.61-3.22]	-	-
AGO:VEN	conclusive	2	609	2002-2002	1.96 [1.56-2.45]	-	-
AGO:VIL	conclusive	-	-	-	1.95 [1.32-2.86]	-	-
AGO:VOR	inconclusive	-	-	-	1.31 [0.99-1.74]	63	858
AMI:BUP	conclusive	1	118	1981-1981	0.62 [0.49-0.80]	-	-
AMI:CIT	conclusive	2	409	1984-1996	0.65 [0.51-0.82]	-	-
AMI:CLO	inconclusive	-	-	-	1.27 [0.94-1.71]	111	877
AMI:DES	conclusive	-	-	-	0.67 [0.49-0.89]	-	-
AMI:DUL	inconclusive	-	-	-	0.82 [0.66-1.03]	79	868
AMI:ESC	conclusive	-	-	-	0.57 [0.46-0.72]	-	-
AMI:FLO	conclusive	13	859	1983-1997	0.57 [0.48-0.68]	-	-
AMI:FLV	inconclusive	3	337	1989-1996	0.89 [0.69-1.16]	196	806
AMI:LEV	inconclusive	-	-	-	0.83 [0.55-1.24]	158	635
AMI:MIL	conclusive	1	87	1987-1987	0.57 [0.40-0.79]	-	-
AMI:MIR	conclusive	7	865	1988-1995	0.70 [0.56-0.87]	-	-
AMI:NEF	conclusive	1	106	1992-1992	0.71 [0.51-0.99]	-	-
AMI:PAR	conclusive	19	2001	1983-2000	0.73 [0.62-0.86]	-	-
AMI:REB	inconclusive	-	-	-	0.88 [0.67-1.14]	220	807
AMI:SER	conclusive	7	1432	1988-1998	0.82 [0.67-0.99]	-	-
AMI:TRA	inconclusive	2	229	1986-1987	0.95 [0.69-1.29]	243	766
AMI:VEN	conclusive	2	272	1998-2001	0.82 [0.68-0.98]	-	-
AMI:VIL	inconclusive	-	-	-	0.81 [0.57-1.16]	150	664
AMI:VOR	conclusive	-	-	-	0.55 [0.42-0.70]	-	-
BUP:CIT	inconclusive	-	-	-	1.03 [0.78-1.38]	269	833
BUP:CLO	conclusive	-	-	-	2.03 [1.46-2.84]	-	-
BUP:DES	inconclusive	-	-	-	1.07 [0.77-1.47]	250	789
BUP:DUL	conclusive	-	-	-	1.32 [1.02-1.71]	-	-
BUP:ESC	inconclusive	2	566	2004-2004	0.92 [0.70-1.20]	253	822
BUP:FLO	inconclusive	2	617	1995-2000	0.92 [0.73-1.16]	270	897
BUP:FLV	conclusive	-	-	-	1.43 [1.05-1.94]	-	-
BUP:LEV	inconclusive	-	-	-	1.32 [0.87-2.02]	151	745
BUP:MIL	inconclusive	-	-	-	0.91 [0.62-1.31]	207	692
BUP:MIR	inconclusive	-	-	-	1.11 [0.85-1.46]	258	878
BUP:NEF	inconclusive	-	-	-	1.14 [0.80-1.64]	225	769
BUP:PAR	inconclusive	2	218	2001-2009	1.17 [0.94-1.47]	255	1003
BUP:REB	conclusive	-	-	-	1.40 [1.04-1.90]	-	-
BUP:SER	conclusive	2	479	1998-1998	1.31 [1.01-1.69]	-	-

BUP:TRA	conclusive	1	124	1992-1992	1.52 [1.08-2.12]	-	-
BUP:VEN	conclusive	2	725	2004-2005	1.31 [1.04-1.64]	-	-
BUP:VIL	inconclusive	-	-	-	1.30 [0.88-1.91]	153	773
BUP:VOR	inconclusive	-	-	-	0.87 [0.66-1.17]	220	793
CIT:CLO	conclusive	1	114	1984-1984	1.97 [1.41-2.74]	-	-
CIT:DES	inconclusive	-	-	-	1.03 [0.74-1.43]	251	777
CIT:DUL	inconclusive	-	-	-	1.28 [0.99-1.65]	63	944
CIT:ESC	inconclusive	8	1750	2000-2008	0.89 [0.69-1.15]	195	819
CIT:FLO	inconclusive	2	673	1994-1995	0.89 [0.71-1.11]	203	876
CIT:FLV	conclusive	1	217	1991-1991	1.38 [1.03-1.85]	-	-
CIT:LEV	inconclusive	-	-	-	1.28 [0.84-1.96]	171	737
CIT:MIL	inconclusive	-	-	-	0.88 [0.61-1.27]	194	684
CIT:MIR	inconclusive	1	270	1997-1997	1.08 [0.82-1.41]	246	861
CIT:NEF	inconclusive	-	-	-	1.11 [0.77-1.58]	232	760
CIT:PAR	inconclusive	1	310	2000-2000	1.13 [0.91-1.42]	256	990
CIT:REB	conclusive	1	101	2004-2004	1.36 [1.01-1.83]	-	-
CIT:SER	inconclusive	2	360	1998-2002	1.26 [0.98-1.63]	68	951
CIT:TRA	conclusive	-	-	-	1.47 [1.03-2.08]	-	-
CIT:VEN	conclusive	1	53	2013-2013	1.26 [1.00-1.59]	-	-
CIT:VIL	inconclusive	1	872	2013-2013	1.26 [0.88-1.79]	171	796
CIT:VOR	inconclusive	-	-	-	0.85 [0.63-1.13]	189	783
CLO:DES	conclusive	-	-	-	0.52 [0.36-0.76]	-	-
CLO:DUL	conclusive	-	-	-	0.65 [0.48-0.89]	-	-
CLO:ESC	conclusive	-	-	-	0.45 [0.33-0.62]	-	-
CLO:FLO	conclusive	2	263	1987-1989	0.45 [0.34-0.60]	-	-
CLO:FLV	conclusive	2	83	1981-1993	0.70 [0.50-0.99]	-	-
CLO:LEV	inconclusive	-	-	-	0.65 [0.41-1.03]	70	526
CLO:MIL	conclusive	1	107	1995-1995	0.45 [0.30-0.66]	-	-
CLO:MIR	conclusive	-	-	-	0.55 [0.40-0.75]	-	-
CLO:NEF	conclusive	-	-	-	0.56 [0.38-0.84]	-	-
CLO:PAR	conclusive	6	1455	1987-1995	0.58 [0.44-0.75]	-	-
CLO:REB	conclusive	-	-	-	0.69 [0.49-0.97]	-	-
CLO:SER	conclusive	2	272	1992-1998	0.64 [0.47-0.87]	-	-
CLO:TRA	inconclusive	-	-	-	0.75 [0.51-1.10]	102	611
CLO:VEN	conclusive	2	223	1991-1996	0.64 [0.48-0.85]	-	-
CLO:VIL	conclusive	-	-	-	0.64 [0.42-0.98]	-	-
CLO:VOR	conclusive	-	-	-	0.43 [0.31-0.60]	-	-
DES:DUL	inconclusive	1	474	2007-2007	1.24 [0.93-1.66]	146	878
DES:ESC	inconclusive	-	-	-	0.86 [0.63-1.18]	200	735
DES:FLO	inconclusive	-	-	-	0.86 [0.65-1.14]	203	776
DES:FLV	inconclusive	-	-	-	1.34 [0.95-1.89]	93	825
DES:LEV	inconclusive	-	-	-	1.24 [0.79-1.95]	183	713

DES:MIL	inconclusive	-	-	-	0.85 [0.57-1.27]	171	643
DES:MIR	inconclusive	-	-	-	1.05 [0.76-1.44]	254	792
DES:NEF	inconclusive	-	-	-	1.07 [0.73-1.59]	224	724
DES:PAR	inconclusive	-	-	-	1.10 [0.84-1.45]	262	869
DES:REB	inconclusive	-	-	-	1.32 [0.94-1.85]	105	829
DES:SER	inconclusive	-	-	-	1.23 [0.90-1.67]	175	854
DES:TRA	inconclusive	-	-	-	1.42 [0.97-2.09]	75	796
DES:VEN	inconclusive	-	-	-	1.23 [0.92-1.63]	157	890
DES:VIL	inconclusive	-	-	-	1.22 [0.80-1.85]	193	734
DES:VOR	inconclusive	-	-	-	0.82 [0.59-1.13]	165	733
DUL:ESC	conclusive	3	1120	2005-2005	0.70 [0.55-0.88]	-	-
DUL:FLO	conclusive	1	103	2000-2000	0.70 [0.57-0.85]	-	-
DUL:FLV	inconclusive	-	-	-	1.08 [0.81-1.44]	264	850
DUL:LEV	inconclusive	-	-	-	1.00 [0.67-1.51]	215	693
DUL:MIL	conclusive	-	-	-	0.69 [0.48-0.97]	-	-
DUL:MIR	inconclusive	-	-	-	0.84 [0.66-1.08]	158	824
DUL:NEF	inconclusive	-	-	-	0.87 [0.62-1.22]	197	708
DUL:PAR	inconclusive	7	2167	2001-2010	0.89 [0.74-1.07]	212	992
DUL:REB	inconclusive	-	-	-	1.06 [0.81-1.40]	271	860
DUL:SER	inconclusive	-	-	-	0.99 [0.78-1.25]	295	919
DUL:TRA	inconclusive	-	-	-	1.15 [0.83-1.60]	230	803
DUL:VEN	inconclusive	2	836	2006-2006	0.99 [0.82-1.20]	316	1014
DUL:VIL	inconclusive	-	-	-	0.98 [0.68-1.42]	229	722
DUL:VOR	conclusive	6	2392	2008-2014	0.66 [0.53-0.83]	-	-
ESC:FLO	inconclusive	4	813	2003-2006	1.00 [0.81-1.23]	312	982
ESC:FLV	conclusive	-	-	-	1.55 [1.16-2.08]	-	-
ESC:LEV	inconclusive	-	-	-	1.44 [0.95-2.18]	87	769
ESC:MIL	inconclusive	-	-	-	0.98 [0.69-1.41]	232	728
ESC:MIR	inconclusive	-	-	-	1.21 [0.94-1.57]	153	929
ESC:NEF	inconclusive	-	-	-	1.24 [0.88-1.76]	178	801
ESC:PAR	conclusive	2	686	2004-2009	1.27 [1.04-1.57]	-	-
ESC:REB	conclusive	-	-	-	1.53 [1.15-2.04]	-	-
ESC:SER	conclusive	3	629	2002-2005	1.42 [1.11-1.81]	-	-
ESC:TRA	conclusive	-	-	-	1.65 [1.17-2.31]	-	-
ESC:VEN	conclusive	2	495	2002-2002	1.42 [1.15-1.76]	-	-
ESC:VIL	inconclusive	-	-	-	1.41 [0.97-2.05]	74	806
ESC:VOR	inconclusive	-	-	-	0.95 [0.72-1.25]	267	835
FLO:FLV	conclusive	1	100	1994-1994	1.55 [1.20-2.01]	-	-
FLO:LEV	inconclusive	-	-	-	1.44 [0.97-2.13]	73	791
FLO:MIL	inconclusive	2	490	1992-1996	0.98 [0.71-1.36]	243	765
FLO:MIR	inconclusive	6	1035	1996-2003	1.21 [0.99-1.49]	60	1049
FLO:NEF	inconclusive	3	161	1995-1995	1.24 [0.91-1.71]	140	838

FLO:PAR	conclusive	11	2512	1991-2000	1.27 [1.11-1.46]	-	-
FLO:REB	conclusive	4	806	1995-2001	1.53 [1.20-1.94]	-	-
FLO:SER	conclusive	3	532	1991-2000	1.42 [1.17-1.72]	-	-
FLO:TRA	conclusive	3	196	1986-1989	1.65 [1.22-2.23]	-	-
FLO:VEN	conclusive	15	3138	1992-2007	1.42 [1.23-1.64]	-	-
FLO:VIL	inconclusive	-	-	-	1.41 [0.99-2.00]	59	834
FLO:VOR	inconclusive	-	-	-	0.95 [0.75-1.20]	285	905
FLV:LEV	inconclusive	-	-	-	0.93 [0.60-1.44]	188	645
FLV:MIL	conclusive	2	239	1989-1999	0.63 [0.43-0.92]	-	-
FLV:MIR	inconclusive	2	412	2000-2000	0.78 [0.59-1.03]	125	741
FLV:NEF	inconclusive	-	-	-	0.80 [0.55-1.17]	143	645
FLV:PAR	inconclusive	2	180	1992-1995	0.82 [0.64-1.05]	78	791
FLV:REB	inconclusive	-	-	-	0.98 [0.71-1.36]	249	768
FLV:SER	inconclusive	2	185	1993-2003	0.91 [0.69-1.21]	203	779
FLV:TRA	inconclusive	-	-	-	1.06 [0.73-1.53]	233	742
FLV:VEN	inconclusive	1	92	2001-2001	0.91 [0.71-1.19]	242	828
FLV:VIL	inconclusive	-	-	-	0.91 [0.61-1.36]	198	670
FLV:VOR	conclusive	-	-	-	0.61 [0.45-0.84]	-	-
LEV:MIL	inconclusive	-	-	-	0.68 [0.42-1.12]	83	521
LEV:MIR	inconclusive	-	-	-	0.84 [0.55-1.28]	163	630
LEV:NEF	inconclusive	-	-	-	0.86 [0.54-1.39]	155	595
LEV:PAR	inconclusive	-	-	-	0.89 [0.60-1.31]	193	671
LEV:REB	inconclusive	-	-	-	1.06 [0.69-1.64]	208	687
LEV:SER	inconclusive	-	-	-	0.99 [0.65-1.49]	212	687
LEV:TRA	inconclusive	-	-	-	1.14 [0.71-1.84]	194	681
LEV:VEN	inconclusive	-	-	-	0.99 [0.66-1.46]	219	700
LEV:VIL	inconclusive	-	-	-	0.98 [0.60-1.61]	177	623
LEV:VOR	inconclusive	-	-	-	0.66 [0.43-1.01]	69	604
MIL:MIR	inconclusive	-	-	-	1.23 [0.86-1.76]	188	789
MIL:NEF	inconclusive	-	-	-	1.26 [0.82-1.94]	178	731
MIL:PAR	inconclusive	2	1207	2002-2011	1.30 [0.95-1.76]	89	861
MIL:REB	conclusive	-	-	-	1.55 [1.06-2.27]	-	-
MIL:SER	conclusive	-	-	-	1.44 [1.02-2.05]	-	-
MIL:TRA	conclusive	-	-	-	1.67 [1.10-2.55]	-	-
MIL:VEN	conclusive	-	-	-	1.44 [1.04-2.01]	-	-
MIL:VIL	inconclusive	-	-	-	1.43 [0.91-2.26]	110	739
MIL:VOR	inconclusive	-	-	-	0.97 [0.66-1.41]	225	714
MIR:NEF	inconclusive	-	-	-	1.03 [0.72-1.46]	240	750
MIR:PAR	inconclusive	4	719	1996-2007	1.05 [0.86-1.29]	306	1017
MIR:REB	inconclusive	-	-	-	1.26 [0.94-1.68]	113	889
MIR:SER	inconclusive	1	346	2001-2001	1.17 [0.92-1.49]	298	954
MIR:TRA	inconclusive	2	300	1993-1995	1.36 [0.98-1.90]	40	792

MIR:VEN	inconclusive	2	533	2004-2004	1.17 [0.95-1.44]	115	1026
MIR:VIL	inconclusive	-	-	-	1.16 [0.80-1.70]	215	755
MIR:VOR	inconclusive	-	-	-	0.78 [0.59-1.04]	82	781
NEF:PAR	inconclusive	2	246	1994-2000	1.03 [0.75-1.40]	258	794
NEF:REB	inconclusive	-	-	-	1.23 [0.85-1.78]	191	775
NEF:SER	inconclusive	1	160	1994-1994	1.14 [0.82-1.59]	251	800
NEF:TRA	inconclusive	-	-	-	1.33 [0.88-2.00]	146	754
NEF:VEN	inconclusive	-	-	-	1.14 [0.83-1.57]	234	813
NEF:VIL	inconclusive	-	-	-	1.14 [0.73-1.77]	203	698
NEF:VOR	inconclusive	-	-	-	0.76 [0.53-1.10]	116	679
PAR:REB	inconclusive	3	1375	2000-2004	1.20 [0.95-1.51]	49	899
PAR:SER	inconclusive	1	192	2000-2000	1.11 [0.92-1.35]	282	1081
PAR:TRA	inconclusive	2	333	1998-2003	1.29 [0.96-1.74]	68	868
PAR:VEN	inconclusive	5	1134	1995-2001	1.11 [0.96-1.29]	251	1246
PAR:VIL	inconclusive	-	-	-	1.11 [0.78-1.56]	237	775
PAR:VOR	conclusive	-	-	-	0.75 [0.59-0.94]	-	-
REB:SER	inconclusive	-	-	-	0.93 [0.70-1.23]	254	809
REB:TRA	inconclusive	-	-	-	1.08 [0.75-1.55]	234	751
REB:VEN	inconclusive	1	167	2000-2000	0.93 [0.72-1.19]	268	858
REB:VIL	inconclusive	-	-	-	0.92 [0.62-1.38]	203	677
REB:VOR	conclusive	-	-	-	0.62 [0.46-0.84]	-	-
SER:TRA	inconclusive	1	122	2004-2004	1.16 [0.84-1.61]	260	802
SER:VEN	inconclusive	4	1058	1998-2004	1.00 [0.83-1.21]	320	1022
SER:VIL	inconclusive	-	-	-	0.99 [0.69-1.44]	230	723
SER:VOR	conclusive	-	-	-	0.67 [0.51-0.87]	-	-
TRA:VEN	inconclusive	1	153	1994-1994	0.86 [0.64-1.17]	200	744
TRA:VIL	inconclusive	-	-	-	0.86 [0.55-1.33]	165	621
TRA:VOR	conclusive	-	-	-	0.58 [0.40-0.82]	-	-
VEN:VIL	inconclusive	-	-	-	0.99 [0.70-1.41]	237	739
VEN:VOR	conclusive	2	767	2007-2013	0.67 [0.53-0.84]	-	-
VIL:VOR	conclusive	-	-	-	0.67 [0.46-0.99]	-	-

3 Conditional power sensitivity analysis

Figure 3a: Sensitivity analysis trial design: r. Lines represent median conditional power across all comparisons with inconclusive evidence, estimated in dependence on the trial design ratio contributing direct/indirect evidence (r). Horizontal red dashed lines indicate 20% and 80% conditional power conventionally at which sample sizes ($N_{CP=20\%}$, $N_{CP=80\%}$) were estimated.

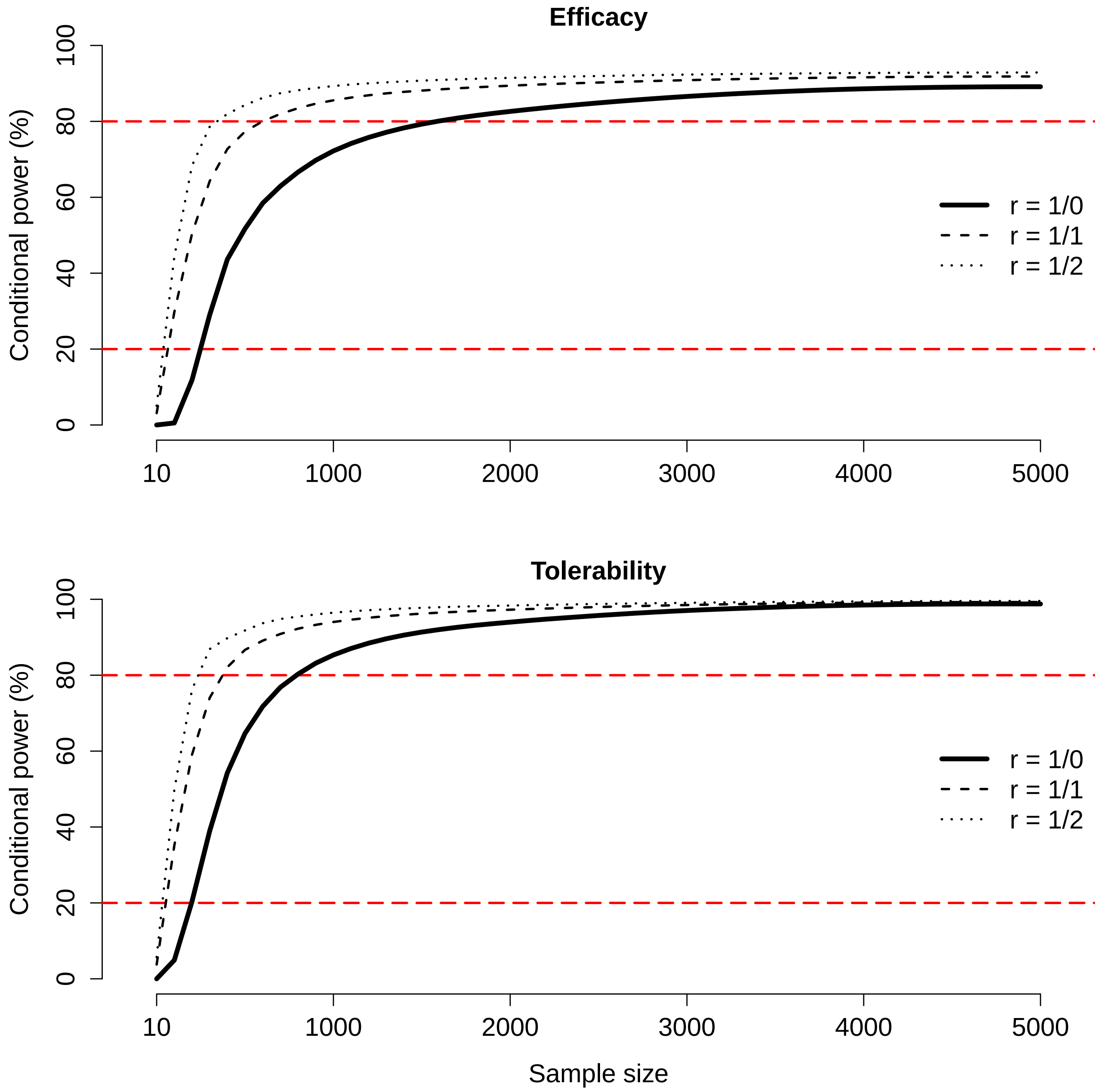

Table 3a: Sensitivity analysis trial design efficacy. Listed are sample size at 20% and 80% conditional power ($N_{CP=20\%}$, $N_{CP=80\%}$) for optimal trial designs considering trial design ratios of r = 1/0, r = 1/1, and r = 1/2. For brevity, non-optimal trial designs are not listed due to space limitations (861 combinations for each treatment comparison).

	r = 1/0 (one trial)		*r* = 1/1 (two trials)			*r* = 1/2 (three trials)		
	$N_{CP=20\%}$	$N_{CP=80\%}$		$N_{CP=20\%}$	$N_{CP=80\%}$		$N_{CP=20\%}$	$N_{CP=80\%}$
AAA:AGO								
AAA:AMI								
AAA:BUP								
AAA:CIT								
AAA:CLO								
AAA:DES								
AAA:DUL								
AAA:ESC								
AAA:FLO								
AAA:FLV								
AAA:LEV								
AAA:MIL								
AAA:MIR								
AAA:NEF								
AAA:PAR								
AAA:REB								
AAA:SER								
AAA:TRA								
AAA:VEN								
AAA:VIL								
AAA:VOR								
AGO:AMI								
AGO:BUP	343	2176	AGO:BUP / PLA:AGO	168	1518	AGO:BUP / AGO:SER / BUP:PAR	132	1338
AGO:CIT	351	2252	AGO:CIT / AGO:ESC	138	1362	AGO:CIT / AGO:ESC / AGO:SER	120	1158
AGO:CLO	153	1304	AGO:CLO / PLA:CLO	92	906	AGO:CLO / PLA:BUP / BUP:ESC	102	720
AGO:DES	300	1801	AGO:DES / PLA:DES	126	1196	AGO:DES / BUP:FLO / BUP:PAR	114	984
AGO:DUL	49	2142	AGO:DUL / PLA:AGO	100	1430	AGO:DUL / PLA:AGO / AGO:VOR	117	1257
AGO:ESC	322	2475	AGO:ESC / AGO:CIT	168	1642	AGO:ESC / PLA:AGO / AGO:CIT	132	1398
AGO:FLO	381	2942	AGO:FLO / PLA:AGO	164	1668	AGO:FLO / PLA:AGO / AGO:AMI	126	1335
AGO:FLV	194	1553	AGO:FLV / PLA:FLV	104	1054	AGO:FLV / PLA:BUP / BUP:PAR	108	837
AGO:LEV	252	1175	AGO:LEV / PLA:LEV	88	772	AGO:LEV / PLA:BUP / BUP:PAR	96	645
AGO:MIL	229	1156	AGO:MIL / PLA:MIL	84	720	AGO:MIL / PLA:BUP / BUP:FLV	93	624

AGO:MIR	66	1799	AGO:MIR / PLA:MIR	108	1330	AGO:MIR / PLA:BUP / AGO:AMI	117	1170
AGO:NEF	275	1480	AGO:NEF / PLA:NEF	100	922	AGO:NEF / PLA:BUP / BUP:PAR	102	738
AGO:PAR								
AGO:REB	156	1797	AGO:REB / PLA:REB	102	1084	AGO:REB / PLA:BUP / BUP:REB	108	855
AGO:SER	368	2412	AGO:SER / AGO:AMI	190	1630	AGO:SER / AGO:AMI / AGO:BUP	135	1386
AGO:TRA	222	1450	AGO:TRA / PLA:TRA	96	940	AGO:TRA / PLA:BUP / BUP:FLO	102	744
AGO:VEN	130	2494	AGO:VEN / PLA:AGO	136	1762	AGO:VEN / PLA:AGO / AGO:FLO	132	1485
AGO:VIL	274	1555	AGO:VIL / PLA:VIL	108	1000	AGO:VIL / PLA:BUP / BUP:REB	105	798
AGO:VOR	326	1986	AGO:VOR / FLO:VOR	178	1500	AGO:VOR / AGO:DUL / BUP:FLV	132	1299
AMI:BUP								
AMI:CIT								
AMI:CLO	58	1576	AMI:CLO / PLA:CLO	86	874	AMI:CLO / PLA:BUP / BUP:FLO	96	675
AMI:DES								
AMI:DUL								
AMI:ESC								
AMI:FLO								
AMI:FLV								
AMI:LEV								
AMI:MIL								
AMI:MIR								
AMI:NEF								
AMI:PAR								
AMI:REB								
AMI:SER								
AMI:TRA								
AMI:VEN								
AMI:VIL								
AMI:VOR								
BUP:CIT	346	2302	BUP:CIT / BUP:ESC	188	1632	BUP:CIT / AGO:FLV / AGO:TRA	138	1431
BUP:CLO	109	1295	BUP:CLO / PLA:CLO	88	884	BUP:CLO / PLA:BUP / BUP:ESC	99	702
BUP:DES	313	1834	BUP:DES / DES:SER	140	1264	BUP:DES / PLA:BUP / BUP:SER	117	1044
BUP:DUL								
BUP:ESC	254	2486	BUP:ESC / BUP:CIT	186	1964	BUP:ESC / AGO:DES / AGO:FLO	150	1659
BUP:FLO	406	2963	BUP:FLO / AMI:BUP	282	2164	BUP:FLO / AGO:CIT / AGO:SER	162	1707
BUP:FLV	131	1555	BUP:FLV / PLA:FLV	116	1220	BUP:FLV / PLA:BUP / BUP:SER	114	996
BUP:LEV	250	1176	BUP:LEV / LEV:SER	108	924	BUP:LEV / PLA:BUP / BUP:SER	105	753
BUP:MIL	213	1147	BUP:MIL / PLA:MIL	98	864	BUP:MIL / PLA:BUP / BUP:SER	99	702
BUP:MIR								
BUP:NEF	284	1493	BUP:NEF / PLA:NEF	130	1108	BUP:NEF / PLA:BUP / BUP:SER	114	906
BUP:PAR								
BUP:REB	224	1824	BUP:REB / REB:SER	148	1420	BUP:REB / PLA:BUP / BUP:SER	129	1239
BUP:SER	385	2648	BUP:SER / AMI:BUP	270	2026	BUP:SER / AGO:CIT / AGO:DES	162	1695

BUP:TRA	187	1493	BUP:TRA / PLA:TRA	126	1220	BUP:TRA / PLA:BUP / BUP:TRA	117	999
BUP:VEN	54	2499	BUP:VEN / AGO:BUP	112	1970	BUP:VEN / AGO:AMI / AGO:LEV	135	1653
BUP:VIL	287	1574	BUP:VIL / PLA:VIL	130	1128	BUP:VIL / PLA:BUP / BUP:TRA	114	915
BUP:VOR	318	2018	BUP:VOR / SER:VOR	216	1662	BUP:VOR / BUP:FLV / BUP:TRA	147	1458
CIT:CLO	131	1349	CIT:CLO / PLA:CLO	100	1012	CIT:CLO / PLA:BUP / BUP:ESC	105	780
CIT:DES	301	1911	CIT:DES / DES:ESC	186	1514	CIT:DES / BUP:ESC / BUP:SER	135	1302
CIT:DUL	59	2319	CIT:DUL / CIT:VOR	118	1940	CIT:DUL / AGO:TRA / BUP:CLO	138	1701
CIT:ESC	341	3034	CIT:ESC / CIT:DUL	250	2468	CIT:ESC / PLA:AGO / AGO:FLV	174	2049
CIT:FLO	375	3208	CIT:FLO / CIT:VEN	240	2286	CIT:FLO / AGO:TRA / AMI:BUP	159	1800
CIT:FLV	225	1706	CIT:FLV / ESC:FLV	150	1380	CIT:FLV / PLA:BUP / BUP:FLO	126	1179
CIT:LEV	256	1205	CIT:LEV / ESC:LEV	104	894	CIT:LEV / BUP:FLO / BUP:FLV	102	723
CIT:MIL	237	1189	CIT:MIL / MIL:VOR	126	1034	CIT:MIL / BUP:FLO / BUP:VOR	111	837
CIT:MIR	56	1965	CIT:MIR / ESC:MIR	114	1820	CIT:MIR / BUP:FLO / BUP:SER	138	1605
CIT:NEF	275	1539	CIT:NEF / ESC:NEF	148	1226	CIT:NEF / AGO:CLO / BUP:FLO	120	1011
CIT:PAR								
CIT:REB	88	1942	CIT:REB / ESC:REB	130	1616	CIT:REB / BUP:FLO / BUP:SER	135	1410
CIT:SER	400	2772	CIT:SER / BUP:CIT	362	2464	CIT:SER / AGO:CIT / AGO:DES	201	2079
CIT:TRA	237	1521	CIT:TRA / ESC:TRA	154	1330	CIT:TRA / BUP:FLO / BUP:FLV	126	1131
CIT:VEN	157	2679	CIT:VEN / CIT:VOR	172	2520	CIT:VEN / AGO:MIL / BUP:CLO	171	2085
CIT:VIL	277	1713	CIT:VIL / ESC:VIL	164	1358	CIT:VIL / BUP:FLO / BUP:TRA	126	1140
CIT:VOR	338	2108	CIT:VOR / ESC:VOR	254	1746	CIT:VOR / BUP:FLO / BUP:TRA	156	1506
CLO:DES	86	1337	CLO:DES / CLO:VOR	112	1140	CLO:DES / PLA:AGO / AGO:MIL	114	957
CLO:DUL	285	1462	CLO:DUL / CLO:VOR	142	1154	CLO:DUL / AGO:MIL / BUP:DES	117	945
CLO:ESC	269	1543	CLO:ESC / CLO:VOR	156	1280	CLO:ESC / AGO:ESC / AGO:LEV	123	1047
CLO:FLO	62	1708	CLO:FLO / CLO:VOR	108	1196	CLO:FLO / PLA:AGO / AGO:CIT	111	900
CLO:FLV	265	1301	CLO:FLV / CLO:VOR	172	1298	CLO:FLV / AGO:SER / BUP:DES	129	1143
CLO:LEV	186	1051	CLO:LEV / LEV:PAR	112	964	CLO:LEV / BUP:DES / BUP:VOR	111	840
CLO:MIL	205	1070	CLO:MIL / MIL:VOR	112	976	CLO:MIL / BUP:DES / BUP:VOR	111	852
CLO:MIR	274	1364	CLO:MIR / AMI:CLO	150	1190	CLO:MIR / AGO:CIT / BUP:CIT	120	1005
CLO:NEF	102	1218	CLO:NEF / CLO:VOR	118	1152	CLO:NEF / BUP:DES / BUP:VOR	117	981
CLO:PAR	308	1716	CLO:PAR / CLO:VOR	168	1306	CLO:PAR / AGO:CIT / AGO:LEV	123	1047
CLO:REB								
CLO:SER	156	1616	CLO:SER / PLA:CLO	110	1092	CLO:SER / PLA:AGO / AGO:CIT	108	840
CLO:TRA	254	1211	CLO:TRA / PLA:CLO	108	952	CLO:TRA / PLA:AGO / BUP:REB	108	813
CLO:VEN	293	1567	CLO:VEN / PLA:CLO	136	1130	CLO:VEN / PLA:AGO / AGO:MIR	114	903
CLO:VIL	83	1247	CLO:VIL / PLA:CLO	92	932	CLO:VIL / PLA:AGO / BUP:REB	105	768
CLO:VOR	210	1366	CLO:VOR / PLA:CLO	110	1028	CLO:VOR / PLA:AGO / AGO:LEV	108	822
DES:DUL								
DES:ESC	136	1905	DES:ESC / CIT:DES	132	1454	DES:ESC / AGO:ESC / AGO:NEF	126	1236
DES:FLO	345	2186	DES:FLO / DES:VEN	174	1438	DES:FLO / AGO:VIL / BUP:CLO	126	1149
DES:FLV	89	1330	DES:FLV / PLA:FLV	112	1202	DES:FLV / PLA:BUP / AGO:TRA	117	1035
DES:LEV	229	1089	DES:LEV / PLA:LEV	98	864	DES:LEV / PLA:BUP / BUP:FLO	102	714

DES:MIL	175	1051	DES:MIL / PLA:MIL	94	836	DES:MIL / PLA:BUP / BUP:FLO	99	693
DES:MIR								
DES:NEF	271	1327	DES:NEF / PLA:NEF	136	1116	DES:NEF / PLA:BUP / BUP:FLO	117	957
DES:PAR								
DES:REB	269	1565	DES:REB / PLA:REB	136	1230	DES:REB / PLA:BUP / AGO:NEF	120	1059
DES:SER	305	1925	DES:SER / AMI:DES	198	1532	DES:SER / AGO:CIT / AGO:NEF	135	1293
DES:TRA	123	1268	DES:TRA / PLA:TRA	112	1122	DES:TRA / PLA:BUP / BUP:FLO	114	936
DES:VEN	49	1826	DES:VEN / DES:FLO	10	1348	DES:VEN / PLA:AGO / AGO:NEF	10	1140
DES:VIL	278	1391	DES:VIL / PLA:VIL	128	1088	DES:VIL / PLA:BUP / BUP:FLO	114	930
DES:VOR	268	1670	DES:VOR / DES:DUL	164	1418	DES:VOR / AGO:MIL / BUP:CLO	132	1254
DUL:ESC	316	2987	DUL:ESC / CIT:DUL	220	2312	DUL:ESC / AGO:ESC / BUP:CIT	168	1968
DUL:FLO								
DUL:FLV	310	1782	DUL:FLV / FLV:VOR	150	1278	DUL:FLV / PLA:BUP / BUP:VOR	120	1041
DUL:LEV	180	1263	DUL:LEV / LEV:VOR	100	920	DUL:LEV / BUP:PAR / BUP:VOR	102	732
DUL:MIL	249	1258	DUL:MIL / PLA:MIL	98	866	DUL:MIL / PLA:BUP / BUP:FLO	99	696
DUL:MIR	343	2164	DUL:MIR / MIR:VOR	214	1632	DUL:MIR / BUP:FLO / BUP:VOR	141	1404
DUL:NEF	50	1577	DUL:NEF / NEF:VOR	100	1126	DUL:NEF / BUP:PAR / BUP:VOR	105	870
DUL:PAR	471	3546	DUL:PAR / CLO:DUL	330	2568	DUL:PAR / AGO:CIT / AGO:FLV	177	1953
DUL:REB								
DUL:SER								
DUL:TRA	294	1635	DUL:TRA / TRA:VOR	142	1210	DUL:TRA / BUP:FLO / BUP:VOR	117	981
DUL:VEN	407	2994	DUL:VEN / DUL:FLO	308	2384	DUL:VEN / AGO:PAR / AGO:VEN	180	1986
DUL:VIL								
DUL:VOR	101	2425	DUL:VOR / ESC:VOR	132	1798	DUL:VOR / BUP:FLO / BUP:REB	135	1533
ESC:FLO	52	3750	ESC:FLO / AMI:ESC	104	2898	ESC:FLO / AGO:CIT / BUP:DUL	144	2250
ESC:FLV	298	1805	ESC:FLV / PLA:FLV	146	1296	ESC:FLV / PLA:BUP / AGO:FLO	120	1041
ESC:LEV	251	1259	ESC:LEV / CIT:LEV	112	948	ESC:LEV / AGO:FLO / BUP:PAR	105	762
ESC:MIL	260	1245	ESC:MIL / CIT:MIL	108	922	ESC:MIL / PLA:BUP / AGO:FLO	102	738
ESC:MIR	252	2197	ESC:MIR / CIT:MIR	160	1638	ESC:MIR / AGO:FLO / AGO:PAR	135	1389
ESC:NEF	201	1622	ESC:NEF / CIT:NEF	114	1080	ESC:NEF / PLA:BUP / AGO:FLO	108	843
ESC:PAR	283	3689	ESC:PAR / CLO:ESC	214	3028	ESC:PAR / AGO:CIT / AGO:FLV	183	2316
ESC:REB								
ESC:SER	323	3019	ESC:SER / CIT:SER	226	2506	ESC:SER / AGO:CIT / AGO:FLO	171	2094
ESC:TRA	293	1642	ESC:TRA / CIT:TRA	168	1320	ESC:TRA / AGO:FLO / AGO:PAR	123	1083
ESC:VEN	386	3148	ESC:VEN / ESC:FLO	324	2788	ESC:VEN / AGO:FLO / AGO:REB	201	2283
ESC:VIL	158	1731	ESC:VIL / DUL:VIL	126	1258	ESC:VIL / AGO:FLO / AGO:PAR	117	1008
ESC:VOR	341	2314	ESC:VOR / CIT:VOR	230	1752	ESC:VOR / AGO:FLO / BUP:FLV	150	1488
FLO:FLV	51	1853	FLO:FLV / PLA:FLV	102	1348	FLO:FLV / PLA:BUP / BUP:VEN	117	1068
FLO:LEV	254	1253	FLO:LEV / LEV:PAR	114	952	FLO:LEV / BUP:PAR / BUP:VEN	105	753
FLO:MIL	180	1245	FLO:MIL / MIL:VEN	104	948	FLO:MIL / PLA:BUP / BUP:VEN	102	747
FLO:MIR								
FLO:NEF	308	1722	FLO:NEF / NEF:VEN	142	1172	FLO:NEF / BUP:PAR / BUP:VEN	114	915

FLO:PAR								
FLO:REB	280	2290	FLO:REB / REB:VEN	178	1632	FLO:REB / BUP:SER / BUP:VEN	135	1329
FLO:SER	414	3711	FLO:SER / AGO:SER	334	3032	FLO:SER / AGO:BUP / BUP:VEN	198	2331
FLO:TRA	90	1689	FLO:TRA / TRA:VOR	124	1366	FLO:TRA / BUP:REB / BUP:VEN	120	1107
FLO:VEN								
FLO:VIL	314	1826	FLO:VIL / VEN:VIL	170	1334	FLO:VIL / BUP:REB / BUP:VIL	123	1059
FLO:VOR	318	2510	FLO:VOR / AMI:VOR	196	1788	FLO:VOR / AGO:CLO / BUP:REB	141	1452
FLV:LEV	209	1122	FLV:LEV / LEV:MIR	102	894	FLV:LEV / AGO:VEN / BUP:MIR	102	741
FLV:MIL	254	1198	FLV:MIL / MIL:VOR	124	1022	FLV:MIL / BUP:MIR / BUP:SER	111	864
FLV:MIR	295	1689	FLV:MIR / AMI:FLV	188	1462	FLV:MIR / AGO:CIT / BUP:FLO	135	1275
FLV:NEF	120	1337	FLV:NEF / AMI:NEF	96	984	FLV:NEF / PLA:BUP / AGO:CLO	105	795
FLV:PAR	332	2078	FLV:PAR / PLA:FLV	162	1356	FLV:PAR / PLA:AGO / AGO:NEF	120	1059
FLV:REB								
FLV:SER	170	1919	FLV:SER / PLA:FLV	126	1352	FLV:SER / PLA:AGO / AGO:DES	120	1113
FLV:TRA	273	1342	FLV:TRA / MIL:TRA	120	1050	FLV:TRA / PLA:AGO / PLA:BUP	114	912
FLV:VEN	325	1926	FLV:VEN / PLA:FLV	168	1374	FLV:VEN / PLA:AGO / AGO:TRA	123	1119
FLV:VIL	93	1383	FLV:VIL / MIL:VIL	102	1048	FLV:VIL / PLA:AGO / BUP:NEF	111	873
FLV:VOR	250	1595	FLV:VOR / DUL:FLV	128	1200	FLV:VOR / PLA:AGO / AGO:NEF	117	987
LEV:MIL	188	894	LEV:MIL / FLV:LEV	96	828	LEV:MIL / PLA:BUP / BUP:DES	102	711
LEV:MIR	147	1069	LEV:MIR / PLA:LEV	90	828	LEV:MIR / PLA:AGO / AGO:CIT	99	678
LEV:NEF	226	1048	LEV:NEF / LEV:PAR	104	898	LEV:NEF / AGO:TRA / BUP:DES	105	762
LEV:PAR	163	1197	LEV:PAR / FLO:LEV	98	888	LEV:PAR / PLA:AGO / AGO:TRA	99	696
LEV:REB	197	1150	LEV:REB / PLA:LEV	94	838	LEV:REB / PLA:AGO / BUP:DES	99	693
LEV:SER	259	1228	LEV:SER / PLA:LEV	98	852	LEV:SER / PLA:AGO / AGO:CIT	99	684
LEV:TRA	194	1001	LEV:TRA / FLO:LEV	100	882	LEV:TRA / AGO:TRA / BUP:DES	102	741
LEV:VEN	201	1180	LEV:VEN / FLO:LEV	98	880	LEV:VEN / PLA:AGO / AGO:TRA	99	699
LEV:VIL	227	1076	LEV:VIL / CIT:LEV	106	904	LEV:VIL / PLA:AGO / AGO:ESC	105	765
LEV:VOR	246	1151	LEV:VOR / DUL:LEV	112	938	LEV:VOR / AGO:NEF / BUP:FLV	105	777
MIL:MIR	225	1146	MIL:MIR / PLA:MIL	106	928	MIL:MIR / PLA:AGO / BUP:LEV	105	756
MIL:NEF	193	1083	MIL:NEF / MIL:VOR	106	930	MIL:NEF / BUP:MIR / BUP:VOR	108	807
MIL:PAR	250	1319	MIL:PAR / PLA:MIL	110	960	MIL:PAR / PLA:AGO / AGO:NEF	105	753
MIL:REB	97	1183	MIL:REB / FLO:MIL	94	896	MIL:REB / PLA:AGO / AGO:TRA	102	732
MIL:SER	248	1291	MIL:SER / MIL:VOR	116	992	MIL:SER / AGO:DES / AGO:TRA	108	798
MIL:TRA	230	1054	MIL:TRA / PLA:MIL	104	894	MIL:TRA / PLA:AGO / BUP:FLO	105	753
MIL:VEN	256	1249	MIL:VEN / MIL:VOR	122	1004	MIL:VEN / AGO:TRA / BUP:MIR	108	810
MIL:VIL	184	1104	MIL:VIL / CIT:MIL	106	932	MIL:VIL / PLA:AGO / AGO:ESC	105	789
MIL:VOR	244	1173	MIL:VOR / DUL:MIL	106	916	MIL:VOR / PLA:AGO / AGO:NEF	105	750
MIR:NEF	53	1470	MIR:NEF / AMI:NEF	92	1088	MIR:NEF / AGO:CLO / BUP:PAR	108	843
MIR:PAR	397	2739	MIR:PAR / DUL:MIR	308	2058	MIR:PAR / AGO:AMI / AGO:NEF	165	1623
MIR:REB								
MIR:SER								
MIR:TRA	294	1594	MIR:TRA / FLV:TRA	164	1318	MIR:TRA / AGO:CLO / AGO:VOR	126	1122

MIR:VEN	355	2487	MIR:VEN / MIR:VOR	254	1918	MIR:VEN / AGO:TRA / BUP:NEF	156	1578
MIR:VIL								
MIR:VOR	135	1871	MIR:VOR / DUL:MIR	134	1496	MIR:VOR / AGO:CLO / AGO:NEF	129	1326
NEF:PAR								
NEF:REB	255	1347	NEF:REB / CIT:NEF	130	1120	NEF:REB / AGO:ESC / AGO:TRA	117	957
NEF:SER	278	1557	NEF:SER / AMI:NEF	132	1160	NEF:SER / AGO:CIT / AGO:DES	114	921
NEF:TRA	134	1129	NEF:TRA / AMI:NEF	102	1004	NEF:TRA / AGO:CIT / AGO:CLO	108	831
NEF:VEN	76	1447	NEF:VEN / AMI:NEF	102	1180	NEF:VEN / AGO:CIT / AGO:TRA	111	906
NEF:VIL	258	1223	NEF:VIL / CIT:NEF	120	1016	NEF:VIL / PLA:AGO / PLA:BUP	111	879
NEF:VOR	247	1378	NEF:VOR / DUL:NEF	138	1170	NEF:VOR / AGO:NEF / BUP:MIR	117	993
PAR:REB								
PAR:SER								
PAR:TRA	312	1882	PAR:TRA / DUL:TRA	158	1310	PAR:TRA / AGO:PAR / AGO:VEN	120	1029
PAR:VEN	485	4190	PAR:VEN / MIL:VEN	384	3464	PAR:VEN / AGO:CLO / AGO:LEV	222	2586
PAR:VIL								
PAR:VOR	106	2579	PAR:VOR / CLO:VOR	132	1746	PAR:VOR / AGO:CLO / AGO:VEN	132	1416
REB:SER	128	1801	REB:SER / BUP:REB	138	1498	REB:SER / AGO:CIT / AGO:DES	129	1275
REB:TRA								
REB:VEN								
REB:VIL	254	1330	REB:VIL / PAR:VIL	138	1168	REB:VIL / AGO:VEN / BUP:MIL	117	1011
REB:VOR	96	1567	REB:VOR / DUL:REB	118	1272	REB:VOR / PLA:AGO / AGO:NEF	117	1080
SER:TRA	204	1609	SER:TRA / PLA:TRA	124	1214	SER:TRA / PLA:BUP / AGO:CLO	114	957
SER:VEN	66	2940	SER:VEN / AGO:SER	120	2462	SER:VEN / AGO:AMI / AGO:NEF	147	2040
SER:VIL	285	1686	SER:VIL / AMI:VIL	126	1122	SER:VIL / PLA:BUP / AGO:CLO	111	885
SER:VOR	344	2230	SER:VOR / AMI:VOR	226	1702	SER:VOR / AGO:CLO / AGO:VEN	144	1443
TRA:VEN	307	1718	TRA:VEN / PLA:TRA	132	1144	TRA:VEN / PLA:AGO / AGO:TRA	111	903
TRA:VIL	127	1310	TRA:VIL / CIT:TRA	114	1120	TRA:VIL / BUP:DUL / BUP:ESC	114	942
TRA:VOR	258	1480	TRA:VOR / DUL:TRA	130	1150	TRA:VOR / PLA:AGO / AGO:NEF	114	954
VEN:VIL	56	1746	VEN:VIL / FLO:VIL	100	1126	VEN:VIL / PLA:BUP / AGO:VEN	108	849
VEN:VOR	274	2456	VEN:VOR / FLO:VOR	170	1724	VEN:VOR / AGO:VEN / BUP:REB	138	1452
VIL:VOR	240	1438	VIL:VOR / DUL:VIL	134	1178	VIL:VOR / PLA:AGO / AGO:NEF	117	996

Table 3b: Sensitivity analysis trial design tolerability. Listed are sample size at 20% and 80% conditional power ($N_{CP=20\%}$, $N_{CP=80\%}$) for optimal trial designs considering trial design ratios of r = 1/0, r = 1/1, and r = 1/2. For brevity, non-optimal trial designs are not listed due to space limitations (861 combinations for each treatment comparison).

r = 1/0 (one trial)			*r* = 1/1 (two trials)			*r* = 1/2 (three trials)		
	$N_{CP=20\%}$	$N_{CP=80\%}$		$N_{CP=20\%}$	$N_{CP=80\%}$		$N_{CP=20\%}$	$N_{CP=80\%}$
AAA:AGO	264	1065	AAA:AGO / AGO:AMI	122	878	AAA:AGO / AGO:PAR / AGO:VEN	117	819
AAA:AMI								
AAA:BUP								
AAA:CIT								
AAA:CLO								
AAA:DES								
AAA:DUL								
AAA:ESC								
AAA:FLO								
AAA:FLV								
AAA:LEV								
AAA:MIL								
AAA:MIR								
AAA:NEF								
AAA:PAR								
AAA:REB								
AAA:SER								
AAA:TRA								
AAA:VEN								
AAA:VIL								
AAA:VOR								
AGO:AMI								
AGO:BUP								
AGO:CIT								
AGO:CLO								
AGO:DES								
AGO:DUL								
AGO:ESC								
AGO:FLO								
AGO:FLV								
AGO:LEV								
AGO:MIL	97	805	AGO:MIL / MIL:VOR	120	892	AGO:MIL / AGO:AMI / AGO:VOR	117	828

AGO:MIR								
AGO:NEF								
AGO:PAR								
AGO:REB								
AGO:SER								
AGO:TRA								
AGO:VEN								
AGO:VIL								
AGO:VOR	63	858	AGO:VOR / FLO:VOR	112	846	AGO:VOR / PLA:FLO / AGO:FLO	111	771
AMI:BUP								
AMI:CIT								
AMI:CLO	111	877	AMI:CLO / CLO:VOR	142	876	AMI:CLO / AGO:BUP / AGO:VOR	120	840
AMI:DES								
AMI:DUL	79	868	AMI:DUL / AMI:VOR	190	1258	AMI:DUL / AGO:BUP / AGO:VOR	168	1161
AMI:ESC								
AMI:FLO								
AMI:FLV	196	806	AMI:FLV / FLV:VOR	138	924	AMI:FLV / AGO:BUP / AGO:VOR	126	894
AMI:LEV	158	635	AMI:LEV / LEV:VOR	108	788	AMI:LEV / AGO:BUP / AGO:VOR	108	747
AMI:MIL								
AMI:MIR								
AMI:NEF								
AMI:PAR								
AMI:REB	220	807	AMI:REB / REB:SER	160	896	AMI:REB / AGO:BUP / AGO:VOR	126	873
AMI:SER								
AMI:TRA	243	766	AMI:TRA / SER:TRA	120	808	AMI:TRA / AGO:BUP / AGO:VOR	114	786
AMI:VEN								
AMI:VIL	150	664	AMI:VIL / VIL:VOR	118	844	AMI:VIL / AGO:BUP / AGO:VOR	114	804
AMI:VOR								
BUP:CIT	269	833	BUP:CIT / CIT:VOR	252	994	BUP:CIT / AGO:CIT / AGO:VOR	138	951
BUP:CLO								
BUP:DES	250	789	BUP:DES / DES:FLO	150	838	BUP:DES / AGO:CIT / AGO:VOR	114	786
BUP:DUL								
BUP:ESC	253	822	BUP:ESC / BUP:VOR	224	974	BUP:ESC / AGO:CIT / AGO:VOR	135	942
BUP:FLO	270	897	BUP:FLO / BUP:PAR	208	1008	BUP:FLO / AGO:CIT / AGO:VOR	150	1023
BUP:FLV								
BUP:LEV	151	745	BUP:LEV / LEV:VOR	110	780	BUP:LEV / AGO:CIT / AGO:VOR	108	723
BUP:MIL	207	692	BUP:MIL / MIL:VOR	146	864	BUP:MIL / AGO:CIT / AGO:VOR	117	819
BUP:MIR	258	878	BUP:MIR / AMI:BUP	260	1012	BUP:MIR / AGO:CIT / AGO:VOR	138	966
BUP:NEF	225	769	BUP:NEF / NEF:VOR	162	892	BUP:NEF / AGO:CIT / AGO:VOR	120	852
BUP:PAR	255	1003	BUP:PAR / BUP:VOR	206	1068	BUP:PAR / AGO:CIT / AGO:VOR	153	1053
BUP:REB								
BUP:SER								

BUP:TRA								
BUP:VEN								
BUP:VIL	153	773	BUP:VIL / VIL:VOR	124	836	BUP:VIL / AGO:CIT / AGO:VOR	111	777
BUP:VOR	220	793	BUP:VOR / PAR:VOR	146	834	BUP:VOR / PLA:TRA / AGO:REB	114	789
CIT:CLO								
CIT:DES	251	777	CIT:DES / DES:VOR	200	930	CIT:DES / AGO:CLO / AGO:VOR	129	894
CIT:DUL	63	944	CIT:DUL / CIT:VOR	110	894	CIT:DUL / AGO:CLO / AGO:VOR	120	840
CIT:ESC	195	819	CIT:ESC / CIT:DUL	154	936	CIT:ESC / AGO:CLO / AGO:VOR	126	891
CIT:FLO	203	876	CIT:FLO / CIT:VOR	222	1152	CIT:FLO / AGO:CLO / AGO:VOR	162	1110
CIT:FLV								
CIT:LEV	171	737	CIT:LEV / FLO:LEV	104	744	CIT:LEV / AGO:CLO / AGO:VOR	102	684
CIT:MIL	194	684	CIT:MIL / MIL:VOR	132	834	CIT:MIL / AGO:CLO / AGO:VOR	114	780
CIT:MIR	246	861	CIT:MIR / CIT:VEN	122	850	CIT:MIR / AGO:CLO / AGO:VOR	120	831
CIT:NEF	232	760	CIT:NEF / AMI:NEF	102	746	CIT:NEF / AGO:CLO / AGO:VOR	105	714
CIT:PAR	256	990	CIT:PAR / CIT:VEN	132	1038	CIT:PAR / AGO:CLO / AGO:VOR	150	1041
CIT:REB								
CIT:SER	68	951	CIT:SER / CIT:VEN	90	1004	CIT:SER / AGO:CLO / AGO:VOR	132	945
CIT:TRA								
CIT:VEN								
CIT:VIL	171	796	CIT:VIL / FLO:VIL	110	760	CIT:VIL / AGO:CLO / AGO:VOR	105	705
CIT:VOR	189	783	CIT:VOR / AMI:VOR	148	844	CIT:VOR / PLA:MIL / AGO:FLO	117	801
CLO:DES								
CLO:DUL								
CLO:ESC								
CLO:FLO								
CLO:FLV								
CLO:LEV	70	526	CLO:LEV / LEV:PAR	92	722	CLO:LEV / AGO:DES / AGO:VOR	102	666
CLO:MIL								
CLO:MIR								
CLO:NEF								
CLO:PAR								
CLO:REB								
CLO:SER								
CLO:TRA	102	611	CLO:TRA / PAR:TRA	116	814	CLO:TRA / AGO:DES / AGO:VOR	108	732
CLO:VEN								
CLO:VIL								
CLO:VOR								
DES:DUL	146	878	DES:DUL / DES:VOR	126	902	DES:DUL / AGO:DUL / AGO:VOR	123	870
DES:ESC	200	735	DES:ESC / DES:VOR	162	896	DES:ESC / AGO:DUL / AGO:VOR	123	858
DES:FLO	203	776	DES:FLO / DES:VOR	172	936	DES:FLO / AGO:DUL / AGO:VOR	132	930
DES:FLV	93	825	DES:FLV / DES:VOR	118	880	DES:FLV / AGO:DUL / AGO:VOR	114	795
DES:LEV	183	713	DES:LEV / LEV:VOR	112	776	DES:LEV / AGO:DUL / AGO:VOR	105	708

DES:MIL	171	643	DES:MIL / MIL:VEN	108	738	DES:MIL / AGO:DUL / AGO:VOR	102	666
DES:MIR	254	792	DES:MIR / PLA:DES	82	574	DES:MIR / PLA:AGO / PLA:AMI	90	564
DES:NEF	224	724	DES:NEF / PLA:NEF	76	528	DES:NEF / PLA:AGO / PLA:AMI	87	540
DES:PAR	262	869	DES:PAR / DES:FLO	128	800	DES:PAR / AGO:DUL / AGO:VOR	114	786
DES:REB	105	829	DES:REB / PLA:DES	80	570	DES:REB / PLA:AGO / PLA:AMI	90	564
DES:SER	175	854	DES:SER / PLA:DES	94	664	DES:SER / PLA:AGO / PLA:AMI	99	630
DES:TRA	75	796	DES:TRA / PLA:TRA	70	480	DES:TRA / PLA:AGO / PLA:AMI	87	519
DES:VEN	157	890	DES:VEN / PLA:DES	78	540	DES:VEN / PLA:AGO / PLA:AMI	90	558
DES:VIL	193	734	DES:VIL / PLA:VIL	84	604	DES:VIL / PLA:AGO / PLA:AMI	93	588
DES:VOR	165	733	DES:VOR / PLA:DES	94	678	DES:VOR / PLA:AGO / PLA:AMI	99	633
DUL:ESC								
DUL:FLO								
DUL:FLV	264	850	DUL:FLV / PLA:FLV	82	568	DUL:FLV / PLA:AGO / PLA:AMI	90	564
DUL:LEV	215	693	DUL:LEV / PLA:LEV	74	502	DUL:LEV / PLA:AGO / PLA:AMI	87	540
DUL:MIL								
DUL:MIR	158	824	DUL:MIR / PLA:MIR	100	708	DUL:MIR / PLA:AGO / PLA:AMI	99	654
DUL:NEF	197	708	DUL:NEF / PLA:NEF	80	570	DUL:NEF / PLA:AGO / PLA:AMI	93	576
DUL:PAR	212	992	DUL:PAR / DUL:FLO	148	866	DUL:PAR / PLA:AGO / PLA:AMI	123	849
DUL:REB	271	860	DUL:REB / PLA:REB	106	714	DUL:REB / PLA:AGO / PLA:AMI	102	666
DUL:SER	295	919	DUL:SER / SER:VOR	154	830	DUL:SER / PLA:AGO / PLA:AMI	117	801
DUL:TRA	230	803	DUL:TRA / PLA:TRA	86	620	DUL:TRA / PLA:AGO / PLA:AMI	96	606
DUL:VEN	316	1014	DUL:VEN / DUL:FLO	158	832	DUL:VEN / PLA:AGO / PLA:AMI	120	813
DUL:VIL	229	722	DUL:VIL / PLA:VIL	70	466	DUL:VIL / PLA:AGO / PLA:AMI	87	516
DUL:VOR								
ESC:FLO	312	982	ESC:FLO / ESC:VEN	176	864	ESC:FLO / AGO:DUL / AGO:VEN	126	876
ESC:FLV								
ESC:LEV	87	769	ESC:LEV / LEV:VOR	104	776	ESC:LEV / AGO:FLO / AGO:VOR	105	723
ESC:MIL	232	728	ESC:MIL / PLA:MIL	100	694	ESC:MIL / AGO:FLO / AGO:VOR	99	657
ESC:MIR	153	929	ESC:MIR / AMI:ESC	154	898	ESC:MIR / AGO:FLO / AGO:VOR	120	837
ESC:NEF	178	801	ESC:NEF / PLA:NEF	92	654	ESC:NEF / PLA:AGO / PLA:AMI	96	627
ESC:PAR								
ESC:REB								
ESC:SER								
ESC:TRA								
ESC:VEN								
ESC:VIL	74	806	ESC:VIL / PLA:VIL	82	604	ESC:VIL / PLA:AGO / PLA:AMI	96	603
ESC:VOR	267	835	ESC:VOR / FLO:VOR	158	842	ESC:VOR / PLA:BUP / PLA:CIT	117	810
FLO:FLV								
FLO:LEV	73	791	FLO:LEV / LEV:PAR	88	666	FLO:LEV / AGO:FLV / AGO:VOR	99	657
FLO:MIL	243	765	FLO:MIL / PLA:MIL	90	636	FLO:MIL / PLA:AGO / PLA:AMI	96	621
FLO:MIR	60	1049	FLO:MIR / MIR:SER	82	878	FLO:MIR / AGO:DES / AGO:SER	111	795
FLO:NEF	140	838	FLO:NEF / AMI:NEF	80	622	FLO:NEF / PLA:CLO / PLA:DES	96	630

FLO:PAR								
FLO:REB								
FLO:SER								
FLO:TRA								
FLO:VEN								
FLO:VIL	59	834	FLO:VIL / PAR:VIL	88	668	FLO:VIL / AGO:FLV / AGO:VOR	102	666
FLO:VOR	285	905	FLO:VOR / PAR:VOR	166	852	FLO:VOR / AGO:CIT / AGO:REB	126	864
FLV:LEV	188	645	FLV:LEV / LEV:PAR	96	692	FLV:LEV / AGO:LEV / AGO:VOR	99	642
FLV:MIL								
FLV:MIR	125	741	FLV:MIR / FLO:FLV	154	888	FLV:MIR / AGO:LEV / AGO:VOR	120	843
FLV:NEF	143	645	FLV:NEF / AMI:NEF	114	752	FLV:NEF / AGO:LEV / AGO:VOR	102	681
FLV:PAR	78	791	FLV:PAR / FLO:FLV	122	840	FLV:PAR / AGO:LEV / AGO:VOR	117	816
FLV:REB	249	768	FLV:REB / PLA:FLV	104	704	FLV:REB / PLA:AGO / PLA:AMI	99	642
FLV:SER	203	779	FLV:SER / FLV:VEN	158	928	FLV:SER / AGO:LEV / AGO:VOR	126	897
FLV:TRA	233	742	FLV:TRA / PLA:TRA	100	698	FLV:TRA / AGO:DUL / AGO:TRA	99	642
FLV:VEN	242	828	FLV:VEN / FLO:FLV	148	834	FLV:VEN / AGO:LEV / AGO:VOR	120	825
FLV:VIL	198	670	FLV:VIL / PLA:VIL	86	612	FLV:VIL / PLA:AGO / PLA:AMI	93	591
FLV:VOR								
LEV:MIL	83	521	LEV:MIL / PLA:LEV	78	550	LEV:MIL / PLA:AGO / PLA:AMI	90	558
LEV:MIR	163	630	LEV:MIR / LEV:PAR	98	706	LEV:MIR / AGO:MIL / AGO:VOR	102	663
LEV:NEF	155	595	LEV:NEF / LEV:PAR	94	676	LEV:NEF / AGO:MIL / AGO:VOR	96	624
LEV:PAR	193	671	LEV:PAR / PLA:LEV	80	566	LEV:PAR / PLA:AGO / PLA:AMI	93	579
LEV:REB	208	687	LEV:REB / LEV:PAR	88	636	LEV:REB / AGO:MIL / AGO:VOR	96	615
LEV:SER	212	687	LEV:SER / FLO:LEV	90	648	LEV:SER / AGO:MIL / AGO:VOR	96	627
LEV:TRA	194	681	LEV:TRA / LEV:PAR	88	642	LEV:TRA / AGO:MIL / AGO:VOR	96	609
LEV:VEN	219	700	LEV:VEN / FLO:LEV	100	702	LEV:VEN / AGO:MIL / AGO:VOR	102	666
LEV:VIL	177	623	LEV:VIL / LEV:PAR	94	670	LEV:VIL / AGO:MIL / AGO:VOR	96	615
LEV:VOR	69	604	LEV:VOR / DUL:LEV	86	652	LEV:VOR / AGO:LEV / AGO:VOR	99	645
MIL:MIR	188	789	MIL:MIR / MIL:VEN	106	740	MIL:MIR / AGO:MIR / AGO:VOR	105	687
MIL:NEF	178	731	MIL:NEF / MIL:PAR	100	710	MIL:NEF / AGO:MIR / AGO:VOR	99	642
MIL:PAR	89	861	MIL:PAR / PLA:MIL	80	574	MIL:PAR / PLA:AGO / PLA:AMI	93	597
MIL:REB								
MIL:SER								
MIL:TRA								
MIL:VEN								
MIL:VIL	110	739	MIL:VIL / PLA:VIL	68	444	MIL:VIL / PLA:AGO / PLA:AMI	84	495
MIL:VOR	225	714	MIL:VOR / PLA:MIL	80	562	MIL:VOR / PLA:AGO / PLA:AMI	90	564
MIR:NEF	240	750	MIR:NEF / PLA:NEF	68	446	MIR:NEF / PLA:AGO / PLA:AMI	84	501
MIR:PAR	306	1017	MIR:PAR / PLA:MIR	84	584	MIR:PAR / PLA:AGO / PLA:AMI	93	579
MIR:REB	113	889	MIR:REB / PLA:REB	90	640	MIR:REB / PLA:AGO / PLA:AMI	96	606
MIR:SER	298	954	MIR:SER / PLA:MIR	108	704	MIR:SER / PLA:AGO / PLA:AMI	99	642
MIR:TRA	40	792	MIR:TRA / PLA:TRA	64	414	MIR:TRA / PLA:AGO / PLA:AMI	84	492

MIR:VEN	115	1026	MIR:VEN / PLA:MIR	86	626	MIR:VEN / PLA:AGO / PLA:AMI	96	612
MIR:VIL	215	755	MIR:VIL / PLA:VIL	70	474	MIR:VIL / PLA:AGO / PLA:AMI	87	519
MIR:VOR	82	781	MIR:VOR / PLA:MIR	94	676	MIR:VOR / PLA:AGO / PLA:AMI	96	615
NEF:PAR	258	794	NEF:PAR / PLA:NEF	80	562	NEF:PAR / PLA:AGO / PLA:AMI	93	573
NEF:REB	191	775	NEF:REB / PLA:NEF	76	522	NEF:REB / PLA:AGO / PLA:AMI	87	543
NEF:SER	251	800	NEF:SER / AMI:NEF	82	586	NEF:SER / PLA:AGO / PLA:AMI	93	588
NEF:TRA	146	754	NEF:TRA / PLA:NEF	74	508	NEF:TRA / PLA:AGO / PLA:AMI	87	528
NEF:VEN	234	813	NEF:VEN / PLA:NEF	76	526	NEF:VEN / PLA:AGO / PLA:AMI	90	552
NEF:VIL	203	698	NEF:VIL / PLA:VIL	64	414	NEF:VIL / PLA:AGO / PLA:AMI	81	480
NEF:VOR	116	679	NEF:VOR / DUL:NEF	94	698	NEF:VOR / PLA:CLO / PLA:DES	99	645
PAR:REB	49	899	PAR:REB / PLA:REB	84	668	PAR:REB / PLA:AGO / PLA:AMI	99	663
PAR:SER	282	1081	PAR:SER / PLA:SER	144	846	PAR:SER / PLA:AGO / PLA:AMI	120	837
PAR:TRA	68	868	PAR:TRA / FLO:TRA	106	774	PAR:TRA / AGO:REB / AGO:VOR	108	741
PAR:VEN	251	1246	PAR:VEN / AMI:VEN	188	1188	PAR:VEN / AGO:REB / AGO:VOR	162	1128
PAR:VIL	237	775	PAR:VIL / PLA:VIL	84	598	PAR:VIL / PLA:AGO / PLA:AMI	93	597
PAR:VOR								
REB:SER	254	809	REB:SER / AMI:REB	126	782	REB:SER / AGO:LEV / AGO:MIL	108	735
REB:TRA	234	751	REB:TRA / PAR:TRA	104	712	REB:TRA / AGO:SER / AGO:VOR	102	660
REB:VEN	268	858	REB:VEN / FLO:REB	144	816	REB:VEN / AGO:LEV / AGO:MIL	114	783
REB:VIL	203	677	REB:VIL / PAR:VIL	106	728	REB:VIL / AGO:SER / AGO:VOR	102	672
REB:VOR								
SER:TRA	260	802	SER:TRA / FLO:TRA	116	754	SER:TRA / AGO:TRA / AGO:VOR	105	711
SER:VEN	320	1022	SER:VEN / SER:VOR	278	1028	SER:VEN / AGO:TRA / AGO:VOR	159	1044
SER:VIL	230	723	SER:VIL / AMI:VIL	104	716	SER:VIL / AGO:TRA / AGO:VOR	102	675
SER:VOR								
TRA:VEN	200	744	TRA:VEN / FLO:TRA	116	762	TRA:VEN / AGO:VEN / AGO:VOR	108	732
TRA:VIL	165	621	TRA:VIL / PLA:VIL	80	570	TRA:VIL / PLA:AGO / PLA:AMI	90	561
TRA:VOR								
VEN:VIL	237	739	VEN:VIL / PLA:VIL	76	532	VEN:VIL / PLA:AGO / PLA:AMI	90	555
VEN:VOR								
VIL:VOR								

Figure 3b: Sensitivity analysis effect size: f_{xy}. Line represent median conditional power across all comparisons with inconclusive evidence, estimated in dependence on different anticipated alternative effect sizes(f_{xy} = 0.01, 0.1, 0.2, 0.3, 0.5, 0.8) in terms of Cohen's d (small effect d = 0.2, moderate effect d = 0.5, large effect d = 0.8) [22]. Thick lines indicate conditional power estimated at the network estimates (f_{xyN}). Horizontal red dashed lines indicate 20% and 80% conditional power conventionally at which sample sizes ($N_{CP=20\%}$, $N_{CP=80\%}$) were estimated.

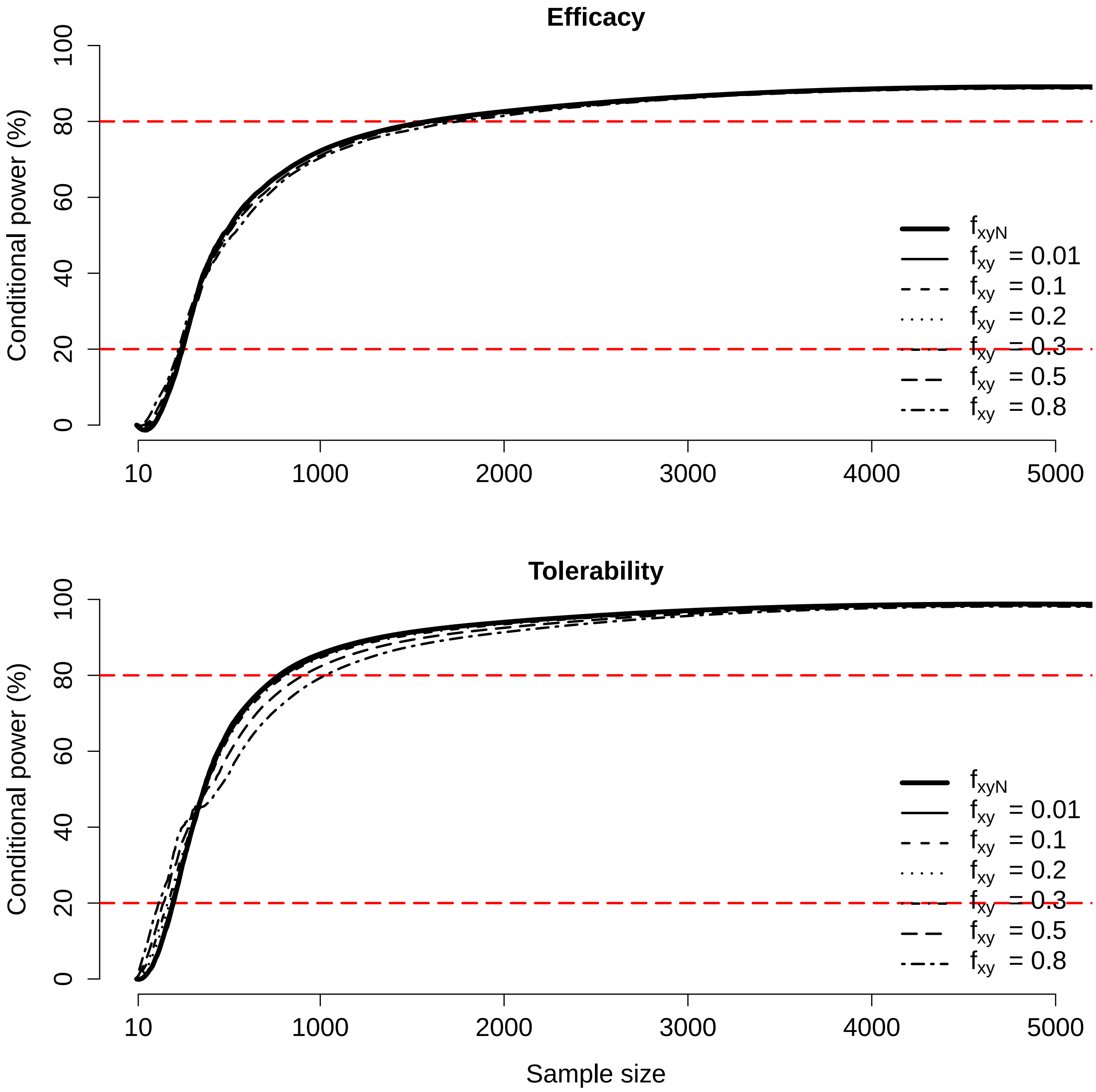

Figure 3c: Sensitivity analysis event probability: pc. Lines represent median conditional power across all comparisons with inconclusive evidence, estimated in dependence on different anticipated alternative event probabilities (pc = 0.01, 0.1, 0.2, 0.3, 0.5). Thick lines indicate conditional power estimated at the average network event probability (pc_N). Horizontal red dashed lines indicate 20% and 80% conditional power conventionally at which sample sizes ($N_{CP=20\%}$, $N_{CP=80\%}$) were estimated.

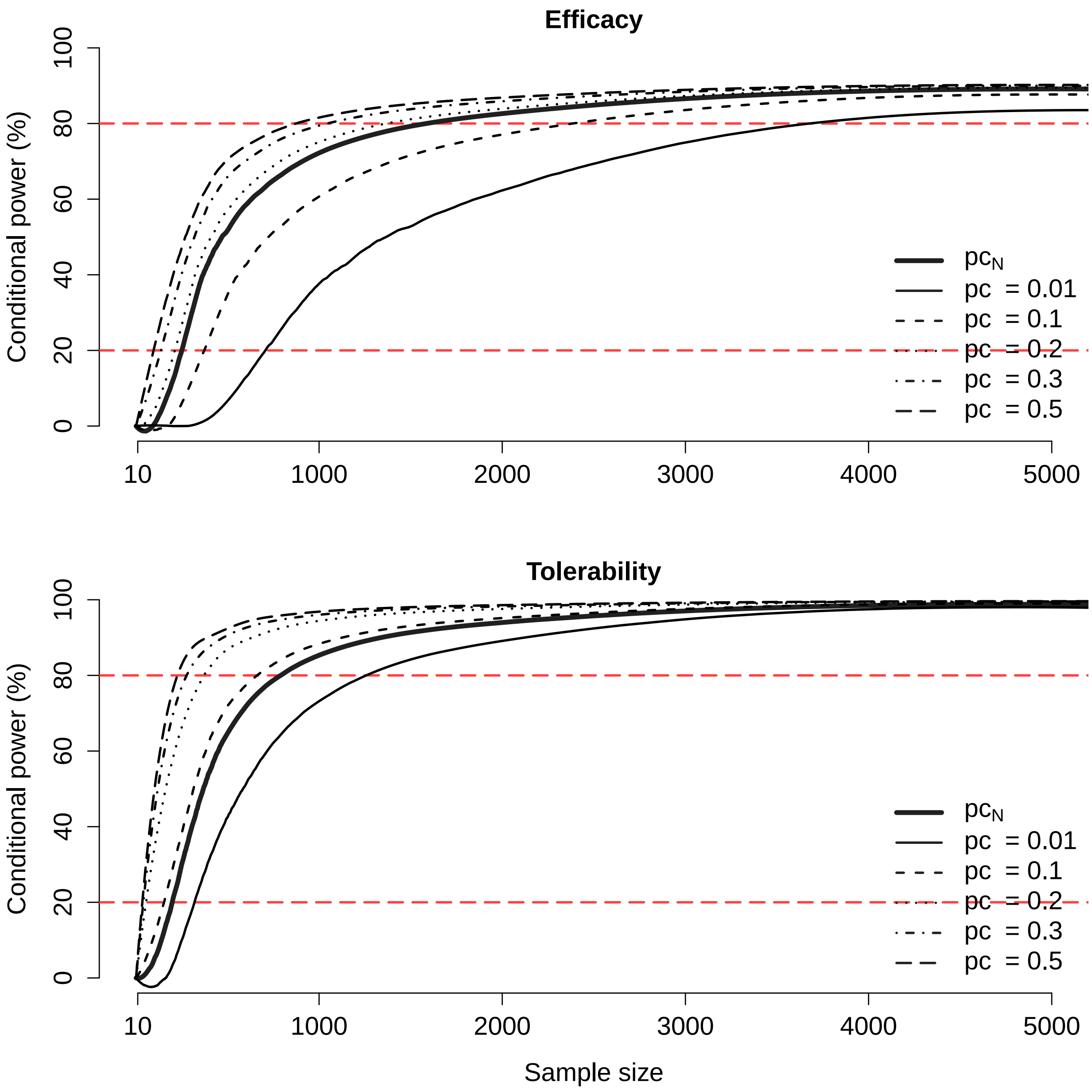

4 List of included studies

Table 4: List of included studies. List of 502 studies of the antidepressant network included in the present analysis, for which information was available on at least one of the primary outcomes, efficacy (438 trials) and/or tolerability (438 trials). Studies are sorted by study year of completion from 1977 - 2017.

ID	Year	Study	Comparison	Efficacy	Tolerability
1	1977	Feighner1979	PLA:AMI	x	x
2	1977	Kellams1979	PLA:TRA	-	x
3	1978	Feighner1980	PLA:TRA	x	-
4	1979	Georgotas1982	PLA:AMI	x	-
5	1979	Mann1981	PLA:TRA	x	-
6	1979	Raft1981	PLA:AMI	-	x
7	1979	Shipley1981	PLA:AMI	x	-
8	1980	GSK14	PLA:BUP	x	x
9	1980	Rickels1982	PLA:AMI:TRA	x	-
10	1980	Roffman1982	PLA:AMI	x	x
11	1980	Thomson1982	PLA:AMI	-	x
12	1981	Chouinard1983a	AMI:BUP	-	x
13	1981	Claghorn1983	PLA:AMI	x	x
14	1981	DeWilde1983a	CLO:FLV	x	x
15	1981	Itil1983	PLA:FLV	-	x
16	1981	Kane1983	PLA:BUP	-	x
17	1982	Amin1984	PLA:FLV	x	-
18	1982	Feighner1984	PLA:BUP	x	x
19	1982	Norton1984	PLA:FLV	x	x
20	1983	Battegay1985	AMI:PAR	-	x
21	1983	Chouinard1985	AMI:FLO	x	x
22	1983	Cohn1985a	PLA:FLO	x	x
23	1983	Dominguez1985	PLA:FLV	-	x
24	1983	Hormazabal1985	PLA:AMI	x	x
25	1983	Masco1985	AMI:FLO	x	x
26	1983	PAR29060.308(HP/81/74A)	AMI:PAR	-	x
27	1983	PAR29060.316(HP/82/47A)	AMI:PAR	-	x
28	1983	PAR29060.318(HP/82/64A)	AMI:PAR	-	x
29	1983	Rickels1985	PLA:AMI	x	x
30	1984	Andersen1986	CIT:CLO	x	x
31	1984	Brown1986	PLA:FLV	-	x
32	1984	Cassano1986	PLA:FLV	x	x
33	1984	Edwards1989	PLA:PAR	x	x
34	1984	MDUK/26090/III/83/007	PLA:PAR	-	x
35	1984	PAR29060.310(HP81/85A)	AMI:PAR	-	x

36	1984	PAR29060.314(HP82/134)	AMI:PAR	-	x
37	1984	Shaw1986	AMI:CIT	x	x
38	1985	Amsterdam1986	PLA:AMI	x	x
39	1985	Lapierre1987	PLA:FLV	-	x
40	1985	MIR003-003	PLA:MIR	x	x
41	1985	PAR01001	PLA:PAR	x	x
42	1985	PAR29060/281	AMI:PAR	x	x
43	1985	Stark1985	PLA:FLO	x	x
44	1985	Study19	PLA:FLO	x	x
45	1985	Study25	PLA:FLO	x	x
46	1985	Study62a	PLA:FLO	x	x
47	1985	Study62b	PLA:FLO	x	x
48	1985	Young1987	AMI:FLO	-	x
49	1986	Blacker1988	AMI:TRA	-	x
50	1986	Byerley1988	PLA:FLO	-	x
51	1986	DeRonchi1998	AMI:FLO	x	x
52	1986	Debus1988	FLO:TRA	-	x
53	1986	Dunbar1993a	PLA:PAR	x	x
54	1986	Dunbar1993b	PLA:PAR	x	x
55	1986	Dunbar1993c	PLA:PAR	x	x
56	1986	Feighner1993a	PLA:PAR	x	x
57	1986	Feighner1993b	PLA:PAR	x	x
58	1986	Feighner1993c	PLA:PAR	x	x
59	1986	Feighner1993d	PLA:PAR	x	x
60	1986	Feighner1993e	PLA:PAR	x	x
61	1986	Feighner1993f	PLA:PAR	x	x
62	1986	Hicks1988	PLA:AMI	-	x
63	1986	Laakman1988	AMI:FLO	x	-
64	1986	PARMDUK032	AMI:PAR	x	x
65	1986	Paykel1988	PLA:AMI	x	-
66	1986	Upward1988	AMI:FLO	-	x
67	1987	2906007001	PLA:AMI:PAR	x	x
68	1987	Altamura1989b	AMI:TRA	x	x
69	1987	Ansseau1989a	AMI:MIL	x	-
70	1987	Ansseau1989b	AMI:MIL	x	x
71	1987	Dunbar1993d	PLA:PAR	x	x
72	1987	Falk1989	FLO:TRA	x	x
73	1987	Fawcett1989	AMI:FLO	x	x
74	1987	Feighner1989a	PLA:FLO	x	x
75	1987	Feighner1989b	PLA:FLV	x	x
76	1987	Ginestet1989	CLO:FLO	x	-
77	1987	Guillibert1989	CLO:PAR	x	x
78	1987	Kuhs1989	AMI:PAR	x	-

79	1987	Larsen1989	PLA:CLO	x	x
80	1987	Lydiard1989	PLA:FLV	-	x
81	1987	Manna1989	CLO:FLO	x	-
82	1987	Ropert1989	CLO:FLO	-	x
83	1988	Cohn1990	AMI:SER	x	x
84	1988	DUAG1990	CLO:PAR	x	x
85	1988	Gelenberg1990	PLA:AMI	-	x
86	1988	Lineberry1990	PLA:BUP	x	x
87	1988	MIR84062	PLA:AMI:MIR	x	-
88	1988	March1990	PLA:FLV	-	x
89	1988	Miller1989	PLA:PAR	x	-
90	1988	Roth1990	PLA:FLV	x	-
91	1988	Smith1990	PLA:AMI:MIR	x	x
92	1989	Ansseau1991	FLV:MIL	x	x
93	1989	Beasley1991	FLO:TRA	x	x
94	1989	Bhatia1991	PLA:AMI	-	x
95	1989	Carman1991	PLA:AMI	x	-
96	1989	Feighner1991	BUP:FLO	x	-
97	1989	Harris1991	AMI:FLV	-	x
98	1989	Keegan1991	AMI:FLO	-	x
99	1989	MDF/29060/III/070/88/MC	CLO:PAR	x	x
100	1989	Noguera1991	CLO:FLO	x	x
101	1989	PAR279MDUK	PLA:PAR	x	x
102	1989	PZ/109	PLA:SER	x	-
103	1989	Preskorn1991	AMI:FLO	x	x
104	1989	Rouillon1991	CLO:PAR	x	x
105	1989	SER315	PLA:AMI:SER	x	x
106	1990	003-008	PLA:MIR	x	x
107	1990	03A0A-004A	PLA:NEF	x	x
108	1990	Bakish1992	PLA:AMI	-	x
109	1990	Bignamini1992	AMI:PAR	x	x
110	1990	CN104-002	PLA:NEF	x	x
111	1990	Claghorn/1992	PLA:PAR	x	x
112	1990	D'Amico1990	PLA:NEF	x	x
113	1990	Hutchinson1992	AMI:PAR	-	x
114	1990	MIR003-020	PLA:AMI:MIR	x	x
115	1990	MIR003-021	PLA:AMI:MIR	x	x
116	1990	PZ/111	PLA:FLO:SER	x	-
117	1990	Reimherr1990	PLA:AMI:SER	x	x
118	1990	Rickels/1992	PLA:PAR	x	x
119	1990	Smith/1992	PLA:PAR	x	x
120	1991	0600A-326	CLO:VEN	-	x
121	1991	Aguglia1993	FLO:SER	x	x

122	1991	Ban1998	PLA:REB	x	x
123	1991	DeWilde1993	FLO:PAR	x	x
124	1991	Gagiano1993	FLO:PAR	x	x
125	1991	Judd1993	AMI:FLO	-	x
126	1991	Katz1993a	PLA:AMI	x	-
127	1991	Katz1993b	PLA:AMI	x	-
128	1991	MY-1008/CPMS-076	PLA:PAR	-	x
129	1991	MY-1043/BRL-029060/115	PLA:FLO:PAR	x	x
130	1991	MY-1045/BRL-029060/1	PLA:FLO:PAR	x	x
131	1991	Moller1993	AMI:PAR	x	x
132	1991	Pelicier1993	CLO:PAR	x	x
133	1991	Reimherr1998	PLA:BUP	x	x
134	1991	Schoene1993	FLO:PAR	x	x
135	1991	Tignol1993	FLO:PAR	x	x
136	1991	Timmerman1993	CIT:FLV	x	x
137	1991	VEN600A-303	PLA:VEN	x	x
138	1991	VEN600A-313	PLA:VEN	x	x
139	1992	0600A-332	FLO:VEN	-	x
140	1992	Ansseau1994a	FLV:PAR	x	x
141	1992	Ansseau1994b	FLO:MIL	-	x
142	1992	Ansseau1994c	AMI:NEF	x	x
143	1992	Bersani1994	AMI:SER	x	x
144	1992	Clerc1994	FLO:VEN	x	x
145	1992	Doogan1994	PLA:SER	x	-
146	1992	Dunner1992	PLA:PAR	x	x
147	1992	Heiligenstein1994	PLA:FLO	x	x
148	1992	Montgomery1992	PLA:CIT	x	x
149	1992	Moon1994	CLO:SER	x	x
150	1992	Ontiveros1994	FLO:PAR	x	x
151	1992	Remick1994	AMI:FLV	-	x
152	1992	Vartiainen1994	PLA:MIR	x	x
153	1992	Weisler1994	BUP:TRA	x	x
154	1992	Wilcox1994	PLA:AMI	x	x
155	1993	Bennie1995	FLO:SER	x	-
156	1993	Faber1995	PLA:SER	x	x
157	1993	Geretsegger1995	AMI:PAR	x	x
158	1993	Lam1995	PLA:FLO	x	x
159	1993	MY-1042/CPMS-251	PLA:PAR	x	x
160	1993	Mynors-Wallis1995	PLA:AMI	x	x
161	1993	Nemeroff1995	FLV:SER	x	x
162	1993	Ottevanger1995	CLO:FLV	x	x
163	1993	Ravindran1995	PLA:SER	-	x
164	1993	Settle1999	PLA:BUP	x	x

165	1993	Sramek1995	PLA:FLO	x	x
166	1993	Staner1995	AMI:PAR	x	x
167	1993	Study205	PLA:BUP	x	x
168	1993	Tollefson1995	PLA:FLO	x	x
169	1993	vanMoffaert1995a	FLO:SER	x	-
170	1993	vanMoffaert1995b	MIR:TRA	x	x
171	1994	29060/356	FLO:PAR	x	x
172	1994	Baldwin1996	NEF:PAR	x	x
173	1994	Christiansen1996	AMI:PAR	x	x
174	1994	Claghorn1996	PLA:FLV	x	x
175	1994	Cunningham1994	PLA:TRA:VEN	x	x
176	1994	Dierick1996	FLO:VEN	x	x
177	1994	Fabre1996	PLA:FLV	x	x
178	1994	Fava1998	PLA:FLO:PAR	x	x
179	1994	Feiger1996	NEF:SER	x	x
180	1994	Fontaine1994	PLA:NEF	x	x
181	1994	Hoyberg1996	AMI:MIR	x	x
182	1994	Mullin1996	AMI:MIR	x	x
183	1994	Patris1996	CIT:FLO	x	x
184	1994	Rapaport1996	FLO:FLV	x	x
185	1994	Rickels1994	PLA:NEF	x	x
186	1994	SER-CHN-1	AMI:PAR	x	x
187	1994	Schweizer1994	PLA:VEN	x	x
188	1994	Walczak1996	PLA:FLV	-	x
189	1995	29060/299	AMI:PAR	x	x
190	1995	845	PLA:BUP	x	x
191	1995	Armitage1997	FLO:NEF	x	x
192	1995	Berlanga1997	FLO:NEF	x	x
193	1995	Bosc1997a	PLA:FLO:REB	x	x
194	1995	Bosc1997b	FLO:REB	x	x
195	1995	Bougerol1997	CIT:FLO	x	x
196	1995	Bremner1995	PLA:AMI:MIR	x	x
197	1995	Buchsbaum1997	PLA:SER	x	-
198	1995	Claghorn1995	PLA:MIR	x	x
199	1995	Ekselius1997	CIT:SER	x	-
200	1995	Gillin1997	FLO:NEF	x	x
201	1995	Guelfi1995	PLA:VEN	x	x
202	1995	Halikas1995	PLA:MIR:TRA	x	x
203	1995	Kavoussi1997	BUP:SER	x	-
204	1995	Kiev1997	FLV:PAR	x	x
205	1995	Lecrubier1997	PLA:VEN	x	x
206	1995	Leinonen1997	CLO:MIL	x	x
207	1995	Lydiard1997	PLA:AMI:SER	x	x

208	1995	Mendels1995	PLA:NEF	x	x
209	1995	Olie1997	PLA:SER	x	x
210	1995	Ravindran1997	CLO:PAR	x	x
211	1995	Rickels1995	PLA:NEF	x	x
212	1995	Study015	PLA:REB	x	x
213	1995	Study032a	PLA:REB	x	x
214	1995	Tylee1997	FLO:VEN	-	x
215	1995	VENXR367	PLA:PAR:VEN	x	x
216	1995	WELL029	PLA:BUP	x	-
217	1995	WELLAK1A4006	PLA:BUP:FLO	x	x
218	1995	Wellbutrin06	PLA:BUP	x	x
219	1995	Wellbutrin25	PLA:BUP	x	x
220	1995	Zivkov1995	AMI:MIR	x	x
221	1996	003-042	PLA:MIR	x	x
222	1996	003-048	PLA:FLO:MIR	x	x
223	1996	0600B1-367	PAR:VEN	-	x
224	1996	Cohn1996	PLA:NEF	x	x
225	1996	Costaesilva1998	FLO:VEN	x	x
226	1996	Demyttenaere1998	AMI:FLO	x	x
227	1996	E-1569	MIR:PAR	x	x
228	1996	Feighner1998	PLA:NEF	x	x
229	1996	Guelfi1998	FLO:MIL	x	x
230	1996	Hackett1998	FLV:VEN	x	-
231	1996	Khan1998	PLA:VEN	x	x
232	1996	Kramer1998	PLA:PAR	x	x
233	1996	Kyle1998	AMI:CIT	x	x
234	1996	Marchesi1998	AMI:FLO	x	x
235	1996	McPartlin1998	PAR:VEN	x	x
236	1996	Moller1998	AMI:SER	x	x
237	1996	Murasaki1998	AMI:FLV	x	x
238	1996	OntiverosSanchez1998	AMI:FLO	x	x
239	1996	Samuelian1998	CLO:VEN	x	x
240	1996	Studie009	PLA:REB	x	x
241	1996	Study89306	PLA:CIT	x	-
242	1996	Wheatley1998	FLO:MIR	x	x
243	1997	0600A1-372	FLO:VEN	-	x
244	1997	Alves1999	FLO:VEN	x	x
245	1997	Chouinard1999	FLO:PAR	x	x
246	1997	Cunningham1997	PLA:VEN	x	x
247	1997	Golden2002a	PLA:PAR	x	x
248	1997	Golden2002b	PLA:PAR	x	x
249	1997	Leinonen1999	CIT:MIR	x	x
250	1997	Rapaport2003	PLA:PAR	x	x

251	1997	Rudolph1999	PLA:FLO:VEN	x	x
252	1997	Silverstone1999	PLA:FLO:VEN	x	x
253	1997	Thase1997	PLA:VEN	x	x
254	1997	Versiani1999	AMI:FLO	x	x
255	1998	Aberg-Wisted2000	PAR:SER	x	-
256	1998	Benkert2000	MIR:PAR	x	x
257	1998	Coleman1999	PLA:BUP:SER	x	x
258	1998	Corrigan2000	PLA:FLO	-	x
259	1998	Croft1999	PLA:BUP:SER	x	x
260	1998	Gentil2000	AMI:VEN	x	x
261	1998	Lepine2000	CLO:SER	x	x
262	1998	McGrath2000	PLA:FLO	x	-
263	1998	Mehtonen2000	SER:VEN	-	x
264	1998	Miura2000	AMI:PAR	x	x
265	1998	Moller2000	AMI:SER	x	x
266	1998	Newhouse2000	FLO:SER	x	x
267	1998	Rudolph1998	PLA:VEN	x	x
268	1998	Schneider2003	PLA:SER	x	x
269	1998	Stahl2000	PLA:CIT:SER	x	x
270	1998	Tsutsui2000	PAR:TRA	x	x
271	1998	Tzanakaki2000	FLO:VEN	x	x
272	1998	Versiani2000	PLA:REB	x	x
273	1999	Bjerkenstedt2005	PLA:FLO	x	x
274	1999	CL3-20098-022	PLA:AGO:FLO	x	x
275	1999	CL3-20098-023	PLA:AGO:PAR	x	x
276	1999	CL3-20098-026	PLA:AGO	x	x
277	1999	Clerc2001	FLV:MIL	x	x
278	1999	Feighner1999	PLA:CIT	x	x
279	1999	Mendels1999	PLA:CIT	x	x
280	1999	Montgomery2004b	AGO:PAR	x	x
281	1999	Rush2001	BUP:SER	x	-
282	1999	Sheehan2009a	PLA:FLO:VEN	x	x
283	1999	Study045	PLA:REB	x	x
284	1999	Study049	PLA:REB	x	x
285	2000	Burke2002	PLA:CIT:ESC	x	x
286	2000	CL3-20098-024	PLA:AGO:FLO	x	x
287	2000	Casabona2002	PAR:VEN	-	x
288	2000	Cassano2002	FLO:PAR	x	-
289	2000	Coleman2001	PLA:BUP:FLO	x	x
290	2000	Davidson2002	PLA:SER	x	x
291	2000	Fava2002	FLO:PAR:SER	x	x
292	2000	Goldstein2002	PLA:DUL:FLO	x	x
293	2000	Gorman2002	PLA:CIT:ESC	x	x

294	2000	Hicks2002	NEF:PAR	x	x
295	2000	Jefferson2000	PLA:CIT:PAR	x	x
296	2000	Lepola2003	PLA:CIT:ESC	x	x
297	2000	Loo2002	PLA:AGO:PAR	x	x
298	2000	M/2020/0046	PLA:PAR:REB	x	x
299	2000	M/2020/0047	PLA:PAR:REB	x	x
300	2000	Murasaki2010a	FLV:MIR	x	x
301	2000	Murasaki2010b	FLV:MIR	x	x
302	2000	Sacchetti2002	AMI:PAR	x	x
303	2000	Schatzberg2002	MIR:PAR	x	x
304	2000	Schwartz2002	REB:VEN	x	x
305	2001	0600A-347	FLV:VEN	-	x
306	2001	0600A-349	PAR:VEN	-	x
307	2001	Behnke2003	MIR:SER	-	x
308	2001	Caligiuri2003	BUP:SER	x	-
309	2001	Clayton2003	PLA:FLO:REB	x	x
310	2001	Dalery2003	FLO:FLV	x	-
311	2001	Goldstein2004a	PLA:DUL:PAR	x	x
312	2001	Goldstein2004b	PLA:DUL:PAR	x	x
313	2001	Hong2003	FLO:MIR	x	x
314	2001	Sauer2003	AMI:VEN	x	x
315	2001	Studie032	FLO:REB	x	x
316	2001	StudyHMAQ-GroupB	PLA:DUL:FLO	-	-
317	2001	WELLAK140016	BUP:PAR	x	x
318	2001	Winokur2003	FLO:MIR	x	x
319	2002	0600B1-384	PLA:VEN	-	x
320	2002	Allard2004	CIT:VEN	x	-
321	2002	Bielski2004	ESC:VEN	x	x
322	2002	CL3-20098-036	AGO:VEN	x	x
323	2002	Detke2002a	PLA:DUL	x	x
324	2002	Detke2002b	PLA:DUL	x	x
325	2002	Detke2004	PLA:DUL:PAR	x	x
326	2002	Katz2004	PLA:PAR	-	x
327	2002	Lalit2004	CIT:ESC:SER	x	x
328	2002	Lemoine2007	AGO:VEN	x	x
329	2002	LopezRodriguez2004	PLA:FLO	x	-
330	2002	Montgomery2004a	ESC:VEN	x	x
331	2002	Moscovitch2004	PLA:SER	x	x
332	2002	Olie2007	PLA:AGO	x	x
333	2002	Roose2004	PLA:CIT	x	x
334	2002	Sechter2004	MIL:PAR	x	x
335	2002	Tomarken2004	PLA:BUP	x	-
336	2002	Trivedi2004	PLA:PAR	x	x

337	2002	Wade2002	PLA:ESC	x	x
338	2003	0600B1-402	SER:VEN	-	x
339	2003	Alexopoulos2004	PLA:ESC:SER	x	x
340	2003	Amini2005	FLO:MIR	-	x
341	2003	Colonna2005	CIT:ESC	x	-
342	2003	Fava2005	PLA:FLO	x	x
343	2003	Kasper2005a	PLA:ESC:FLO	x	x
344	2003	Kasper2005b	PAR:TRA	x	x
345	2003	Keller2006b	PLA:PAR	-	x
346	2003	Keller2006c	PLA:PAR	x	x
347	2003	Kennedy2006	PLA:AGO	x	x
348	2003	Moore2005	CIT:ESC	x	x
349	2003	Moreno2005	PLA:FLO	x	-
350	2003	NKD20006	PLA:PAR	x	x
351	2003	Ninan2003	PLA:ESC	x	x
352	2003	Rossini2005	FLV:SER	x	x
353	2003	Sir2005	SER:VEN	x	x
354	2003	Versiani2005	FLO:MIR	x	x
355	2004	Baldwin2006a	PAR:REB	x	x
356	2004	Baldwin2006b	ESC:PAR	x	x
357	2004	Benkert2006	MIR:VEN	x	x
358	2004	Berlanga2006	CIT:REB	x	x
359	2004	Bose2008	PLA:ESC	x	x
360	2004	Boulenger2006	ESC:PAR	x	-
361	2004	Clayton2006a	PLA:BUP:ESC	x	x
362	2004	Clayton2006b	PLA:BUP:ESC	x	x
363	2004	DeMartinis2007	PLA:DES	x	x
364	2004	Dimidjian2006	PLA:PAR	-	x
365	2004	Feiger2009	PLA:DES	x	x
366	2004	Gastpar2006	PLA:CIT	x	x
367	2004	Jefferson2006	PLA:BUP	x	x
368	2004	Keller2006a	PLA:PAR	-	x
369	2004	Kennedy2005	ESC:FLO	x	x
370	2004	Li2006	CIT:ESC	x	x
371	2004	Lieberman2008a	PLA:VEN	x	x
372	2004	Lieberman2008b	PLA:VEN	x	x
373	2004	Liebowitz2007	PLA:DES	x	x
374	2004	Munizza2006	SER:TRA	x	x
375	2004	Perahia2006	PLA:DUL:PAR	x	x
376	2004	Raskin2007	PLA:DUL	x	x
377	2004	SCT-MD-09	ESC:FLO	-	x
378	2004	Schatzberg2006a	PLA:FLO:VEN	x	x
379	2004	Septien-Velez2007	PLA:DES	x	x

380	2004	Shelton2006	SER:VEN	x	x
381	2004	Study043	CIT:REB	x	-
382	2004	Szegedi2006	MIR:VEN	x	x
383	2004	Thase2006	BUP:VEN	x	x
384	2005	Hewett2009	PLA:BUP:VEN	x	x
385	2005	Hewett2010a	PLA:BUP	x	x
386	2005	Hewett2010b	PLA:VEN	x	x
387	2005	Keller2007	FLO:VEN	x	-
388	2005	Khan2007	DUL:ESC	x	x
389	2005	Lee2007	DUL:PAR	x	x
390	2005	Nemeroff2007	PLA:FLO:VEN	x	x
391	2005	Nierenberg2007	PLA:DUL:ESC	x	x
392	2005	Rapaport2009	PLA:PAR	x	x
393	2005	Ventura2007	ESC:SER	x	x
394	2005	Wade2007	DUL:ESC	x	x
395	2005	Yevtushenko2007	CIT:ESC	x	-
396	2006	Binnemann2008	PLA:SER	x	x
397	2006	CL3-20098-048	AGO:PAR	x	x
398	2006	Corruble2013	AGO:ESC	x	x
399	2006	Hale2010	AGO:FLO	x	x
400	2006	Khan/2007	DUL:ESC	x	-
401	2006	Mao2008	ESC:FLO	x	x
402	2006	Perahia2008a	DUL:VEN	x	x
403	2006	Perahia2008b	DUL:VEN	x	x
404	2007	0600A-626	FLO:VEN	-	x
405	2007	0600A-654	FLO:VEN	-	x
406	2007	Alvarez2012	PLA:VEN:VOR	x	x
407	2007	Barber2011	PLA:SER	x	-
408	2007	Boyer2008	PLA:DES	x	x
409	2007	Cutler2009	PLA:DUL	x	x
410	2007	Debonnel2000	MIR:PAR	x	x
411	2007	Heller2009	FLO:VEN	x	-
412	2007	Higuchi2009	PLA:DUL:PAR	x	x
413	2007	Hu2009	CIT:ESC	x	-
414	2007	Jiang2009	CIT:ESC	-	x
415	2007	Kinoshita2009	PLA:MIR	x	x
416	2007	Montgomery2013	PLA:LEV	x	x
417	2007	Mundt2012	PLA:SER	-	x
418	2007	Rickels2009	PLA:VIL	x	x
419	2007	Tourian2009	PLA:DES:DUL	x	x
420	2007	Wang2014	PLA:ESC	x	x
421	2008	AK1102365	PLA:BUP	x	x
422	2008	CAGO178A2303	PLA:AGO:PAR	x	x

423	2008	CL3-20098-062	AGO:DUL	x	x
424	2008	Dube2010	PLA:ESC	x	x
425	2008	Griebel2012	PLA:ESC	x	x
426	2008	Griebel2012b	PLA:PAR	x	x
427	2008	Jain2013	PLA:VOR	x	x
428	2008	Kornstein2010	PLA:DES	x	x
429	2008	Learned2012aStudy1	PLA:VEN	x	x
430	2008	Learned2012bStudy2	PLA:PAR	x	x
431	2008	Li2010	CIT:ESC	x	-
432	2008	Liebowitz2008	PLA:DES	x	x
433	2008	Mahableshwarkar2013	PLA:DUL:VOR	x	x
434	2008	Ou2010	CIT:ESC	x	x
435	2008	Quera-Salva2010	AGO:ESC	x	x
436	2008	Soares/2010	DES:ESC	x	-
437	2008	Stahl2010	PLA:AGO	x	x
438	2008	Zajecka2010	PLA:AGO	x	x
439	2009	Baldwin2012	PLA:DUL:VOR	x	x
440	2009	CL3-20098-070	PLA:AGO	x	x
441	2009	Dunlop2011	PLA:DES	x	x
442	2009	Grunebaum2011	BUP:PAR	x	x
443	2009	Henigsberg2012	PLA:VOR	x	x
444	2009	Hirayasu2011a	PLA:ESC	x	x
445	2009	Hirayasu2011b	PLA:ESC:PAR	x	x
446	2009	Hsu2011	CIT:SER	x	-
447	2009	Khan2011	PLA:VIL	x	x
448	2009	Robinson2014	PLA:DUL	-	x
449	2009	SCT-MD-49	PLA:ESC	x	x
450	2009	Sheehan2009b	PLA:TRA	x	x
451	2010	244(EMD68843-009)	PLA:FLO	x	-
452	2010	245(EMD68843-010)	PLA:FLO:VIL	x	-
453	2010	246(SB659746-003)	PLA:CIT:VIL	x	-
454	2010	247(SB659746-014)	PLA:VIL	x	x
455	2010	248(SB659746-002)	PLA:VIL	x	x
456	2010	Gommoll2014	PLA:LEV	x	x
457	2010	Higuchi2011	PLA:PAR	x	x
458	2010	Iwata2013	PLA:DES	x	x
459	2010	Kasper2012	PLA:ESC	x	x
460	2010	Katona2012	PLA:DUL:VOR	x	x
461	2010	Khazaie/2015	FLO:SER:TRA	x	-
462	2010	Liebowitz2013	PLA:DES	x	x
463	2010	Mischoulon2014	PLA:ESC	x	x
464	2010	NCT00822744	PLA:ESC	-	x
465	2010	NCT01020799	PLA:ESC	x	x

466	2010	NCT01145755	PLA:DUL	x	x
467	2010	Wang/2015	DUL:PAR	x	x
468	2011	Asnis2013	PLA:LEV	x	x
469	2011	Boulenger2014	PLA:DUL:VOR	x	x
470	2011	Brunoni2012	PLA:SER	x	-
471	2011	Clayton2013	PLA:DES	x	x
472	2011	Heun2013	PLA:AGO	x	x
473	2011	Kamijima2013	MIL:PAR	x	x
474	2011	Kennedy2014	PLA:AGO	x	x
475	2011	Lv2013	CIT:ESC	x	-
476	2011	Sambunaris2014	PLA:LEV	x	x
477	2012	Bakish2014	PLA:LEV	x	x
478	2012	Clayton2015	PLA:DES	x	x
479	2012	Hao2014	DUL:PAR	x	-
480	2012	Jacobsen2015	PLA:VOR	x	x
481	2012	Koshino2013	PLA:BUP	x	x
482	2012	Mahableshwarkar2015a	PLA:DUL:VOR	x	x
483	2012	Mahableshwarkar2015b	PLA:VOR	x	x
484	2012	NCT01254305	PLA:LEV	-	x
485	2012	NCT01255787	PLA:VOR	x	x
486	2012	NCT01355081	PLA:VOR	x	x
487	2012	Shu2014	AGO:FLO	x	x
488	2013	Chang2015	FLO:VEN	x	-
489	2013	Croft2014	PLA:VIL	x	x
490	2013	Goodarzi2015	CIT:MIR	x	-
491	2013	Hosseini2015	CIT:VEN	-	x
492	2013	Mao2015	PLA:SER	x	x
493	2013	Mathews2015	PLA:CIT:VIL	x	x
494	2013	McIntyre2014	PLA:VOR	x	x
495	2013	Pomara2013	PLA:VIL	x	-
496	2013	Wang2015	VEN:VOR	x	x
497	2013	Zhang2014	PLA:TRA	x	x
498	2014	Higuchi2014	PLA:VEN	x	x
499	2014	Lundbeck/2016	PLA:PAR:VOR	x	-
500	2014	Mahableshwarkar2015c	PLA:DUL:VOR	x	x
501	2014	NCT01808612	PLA:FLO	x	x
502	2017	Nishimura/2018	PLA:VOR	x	x

References

1. Moher D, Liberati A, Tetzlaff J, Altman DG, The PG. Preferred Reporting Items for Systematic Reviews and Meta-Analyses: The PRISMA Statement [Journal Article]. PLOS Medicine. 2009;6(7):e1000097. Available from: https://doi.org/10.1371/journal.pmed.1000097.

2. Cipriani A. GRISELDA Open dataset, Group of Researchers Investigating Specific Efficacy of individual Drugs for Acute depression [Journal Article]. The Lancet. 2018;.

3. Cipriani A, Furukawa TA, Salanti G, Chaimani A, Atkinson LZ, Ogawa Y, et al. Comparative efficacy and acceptability of 21 antidepressant drugs for the acute treatment of adults with major depressive disorder: a systematic review and network meta-analysis [Journal Article]. The Lancet. 2018;391(10128):1357–1366. Available from: http://dx.doi.org/10.1016/S0140-6736(17)32802-7.

4. Feiger AD, Tourian KA, Rosas GR, Padmanabhan SK. A Placebo-Controlled Study Evaluating the Efficacy and Safety of Flexible-Dose Desvenlafaxine Treatment in Outpatients with Major Depressive Disorder [Journal Article]. CNS Spectrums. 2009;14(1):41–50. Available from: https://www.cambridge.org/core/article/placebocontrolled-study-evaluating-the-efficacy-and-safety-of-flexibledose-desvenlafaxine-treatment 76A9BF557365AA29766F8990CC14F19E.

5. Septien-Velez L, Pitrosky B, Padmanabhan SK, Germain JM, Tourian KA. A randomized, double-blind, placebo-controlled trial of desvenlafaxine succinate in the treatment of major depressive disorder [Journal Article]. International Clinical Psychopharmacology. 2007;22(6). Available from: https://journals.lww.com/intclinpsychopharm/Fulltext/2007/11000/A_randomized,_double_blind,_placebo_controlled.4.aspx.

6. Liebowitz MR, Yeung PP, Entsuah R. A Randomized, Double-Blind, Placebo-Controlled Trial of Desvenlafaxine Succinate in Adult Outpatients With Major Depressive Disorder [Journal Article]. The Journal of Clinical Psychiatry. 2007;68(11):1663–1672.

7. Kornstein SG, Jiang Q, Reddy S, Musgnung JJ, Guico-Pabia CJ. Short-Term Efficacy and Safety of Desvenlafaxine in a Randomized, Placebo-Controlled Study of Perimenopausal and Postmenopausal Women With Major Depressive Disorder [Journal Article]. The Journal of Clinical Psychiatry. 2010;71(8):1088–1096.

8. Soares CN, Thase ME, Clayton A, Guico-Pabia CJ, Focht K, Jiang Q, et al. Desvenlafaxine and escitalopram for the treatment of postmenopausal women with major depressive disorder [Journal Article]. Menopause. 2010;17(4):700–11.

9. Nishimura A, Aritomi Y, Sasai K, Kitagawa T, Mahableshwarkar A. Randomized, double-blind, placebo-controlled 8-week trialof the efficacy, safety, and tolerability of 5, 10, and 20 mg day vortioxetine in adults with major depressive disorder [Journal Article]. Psychiatry and Clinical Neurosciences. 2018;72(2):64–72.

10. Wang Z, Xu X, Tan Q, Li K, Ma C, Xie S, et al. Treatment of major depressive disorders with generic duloxetine and paroxetine: a multi-centered, double-blind, double-dummy, randomized controlled clinical trial [Journal Article]. Shanghai archives of psychiatry. 2015;27(4):228–236.

11. Khazaie H, Rezaie L, Rezaei Payam N, Najafi F. Antidepressant-induced sexual dysfunction during treatment with fluoxetine, sertraline and trazodone; a randomized controlled trial [Journal Article]. Gen Hosp Psychiatry. 2015;37(1):40–5.

12. Khan A, Bose A, Alexopoulos GS, Gommoll C, Li D, Gandhi C. Double-blind comparison of escitalopram and duloxetine in the acute treatment of major depressive disorder [Journal Article]. Clin Drug Investig. 2007;27(7):481–92.

13. Lundbeck H. Efficacy of Vortioxetine on Cognitive Dysfunction in Working Patients With Major Depressive Disorder [Journal Article]. Clinical Trials. 2016;.

14. Rickels K, Amsterdam J, Clary C, Fox I, Schweizer E, Weise C. The efficacy and safety of paroxetine compared with placebo in outpatients with major depression [Journal Article]. J Clin Psychiatry. 1992;53 Suppl:30–2.

15. Claghorn JL. The safety and efficacy of paroxetine compared with placebo in a double-blind trial of depressed outpatients [Journal Article]. J Clin Psychiatry. 1992;53 Suppl:33–5.

16. Smith WT, Glaudin V. A placebo-controlled trial of paroxetine in the treatment of major depression [Journal Article]. The Journal of Clinical Psychiatry. 1992;53(Suppl):36–39.

17. Hamilton M. A rating scale for depression [Journal Article]. Journal of Neurology, Neurosurgery, Psychiatry. 1960;23(1):56–62.

18. Moncrieff J, Kirsch I. Efficacy of antidepressants in adults [Journal Article]. BMJ (Clinical research ed). 2005;331(7509):155–157. Available from: https://www.ncbi.nlm.nih.gov/pubmed/16020858https://www.ncbi.nlm.nih.gov/pmc/PMC558707/.

19. Altman DG, Royston P. The cost of dichotomising continuous variables [Journal Article]. BMJ (Clinical research ed). 2006;332(7549):1080–1080. Available from: https://www.ncbi.nlm.nih.gov/pubmed/16675816https://www.ncbi.nlm.nih.gov/pmc/articles/PMC1458573/.

20. Hengartner MP. Methodological Flaws, Conflicts of Interest, and Scientific Fallacies: Implications for the Evaluation of Antidepressants' Efficacy and Harm [Journal Article]. Frontiers in psychiatry. 2017;8:275–275. Available from: https://www.ncbi.nlm.nih.gov/pubmed/29270136https://www.ncbi.nlm.nih.gov/pmc/PMC5725408/.

21. MacCallum RC, Zhang S, Preacher KJ, Rucker DD. On the practice of dichotomization of quantitative variables [Journal Article]. Psychological Methods. 2002;7(1):19–40.

22. Borenstein M, Hedges L, Higgins JPT, Rothstein H. Introduction to Meta-Analysis. John Wiley and Sons; 2009.
