# Supplementary material for: Conditional power of antidepressant network meta-analysis
Source: BMC Psychiatry. 2021 Mar 5;21:129. doi: 10.1186/s12888-021-03094-5 (PMC7934491; doi:10.1186/s12888-021-03094-5)
Supplement: Supplementary file 2 — Additional file 2 Supplement2 provides illustrations of conditional power results for individual treatment comparisons. [file 12888_2021_3094_MOESM2_ESM.pdf]

Supplementary appendix 2: Conditional power of antidepressant network meta-analysis

Holper L, Department of Psychiatry, Psychotherapy, and Psychosomatics, University Hospital of Psychiatry, University of Zurich, Lenggstrasse 31, 8032 Zurich, Switzerland

This supplementary appendix illustrates conditional power of an updated NMA based on the approach by Nikolakopoulou et al. [1] for each of the N = 231 comparisons of the antidepressant network (N = 504 trials) provided by Cipriani et al. [2]. In each plot, vertical axes indicate conditional power, horizontal axes indicate the number of randomized patients (1:1 randomization) in future trials. Results are shown based on the main analysis considering a trial design ratio of r = 1/0, anticipated alternative effect sizes equal to the network estimates (f_{xyN}), and anticipated event probabilities equal to the average network event probability (pc_N). Comparisons with conclusive evidence are represented by black lines, comparisons with inconclusive evidence are highlighted by red lines. Results are shown for the outcomes efficacy in terms of the symptom change on the Hamilton Depression Scale (HAMD) [3] (dashed lines) and tolerability in terms of the dropout rate due to adverse events (solid lines).

Figure 1:

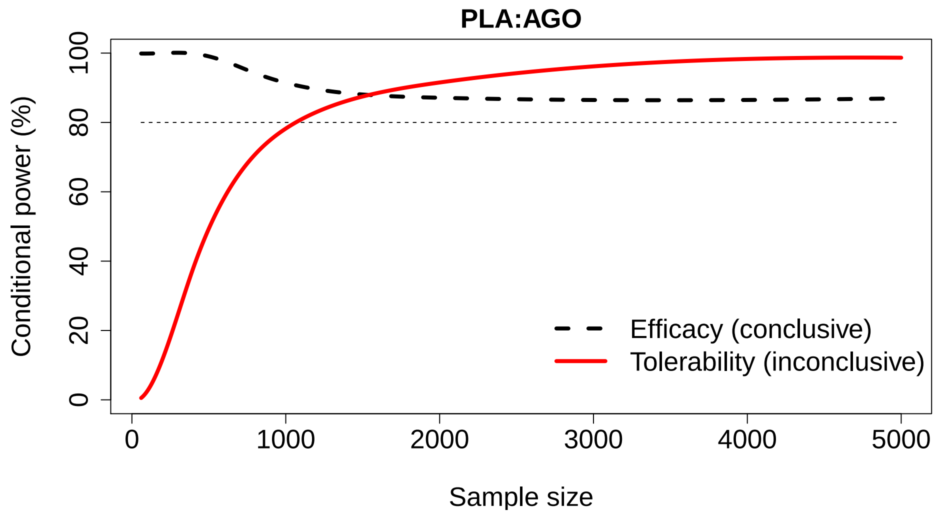

Figure 2:

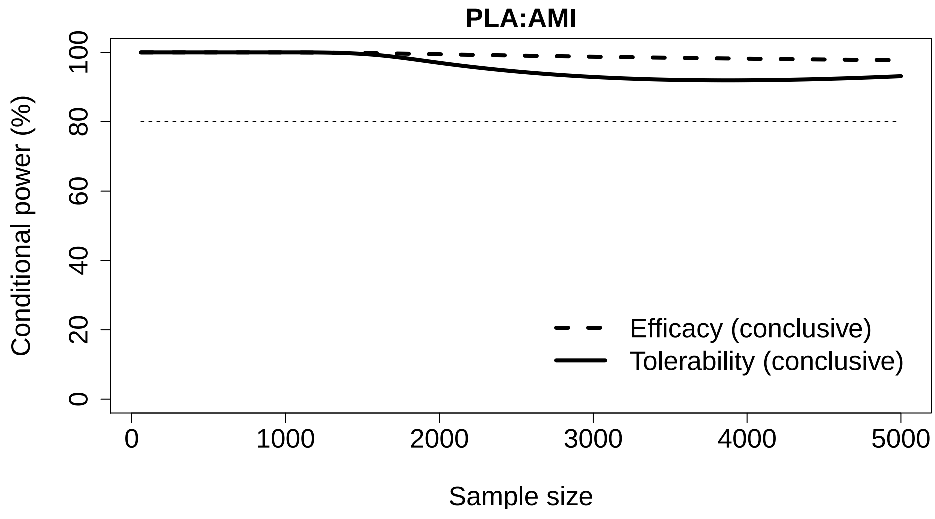

Figure 3:

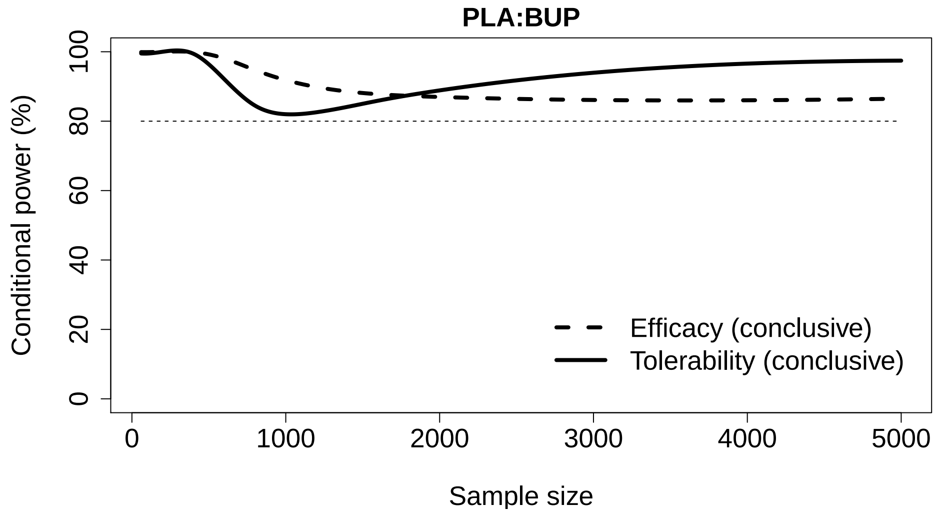

Figure 4:

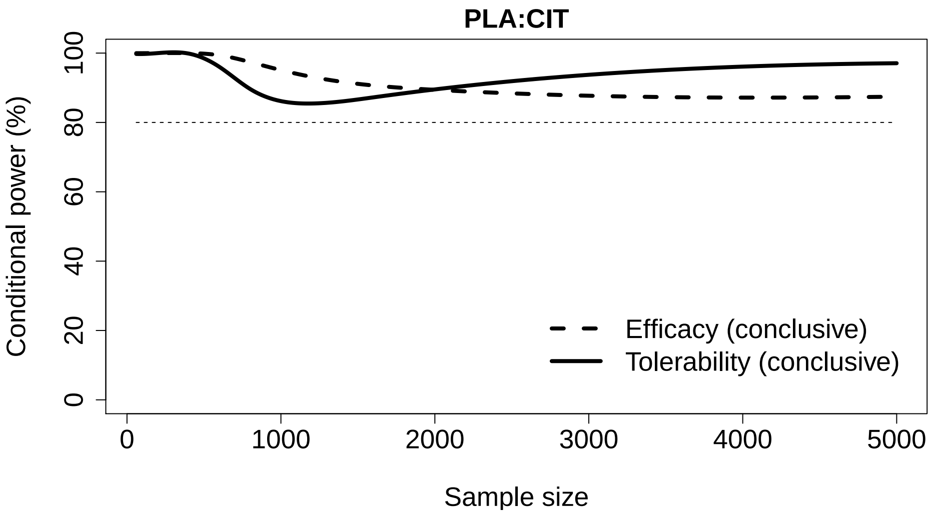

Figure 5:

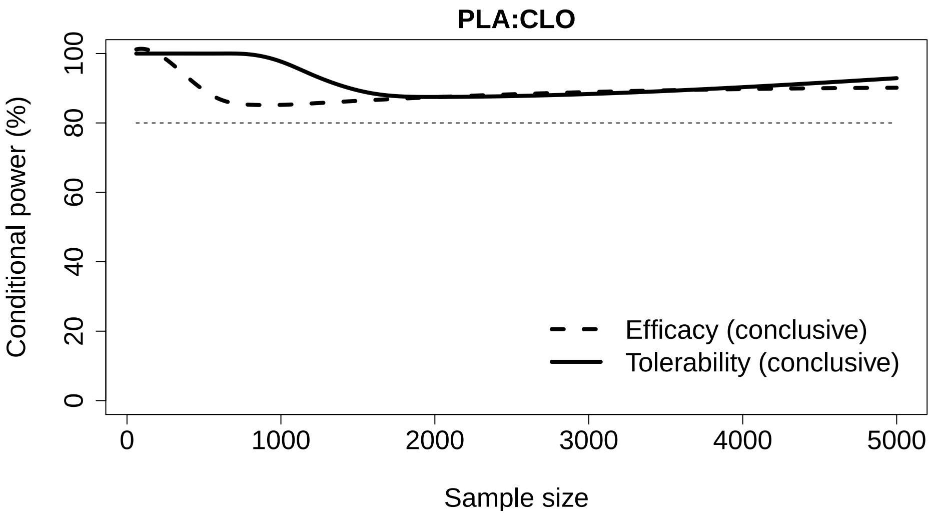

Figure 6:

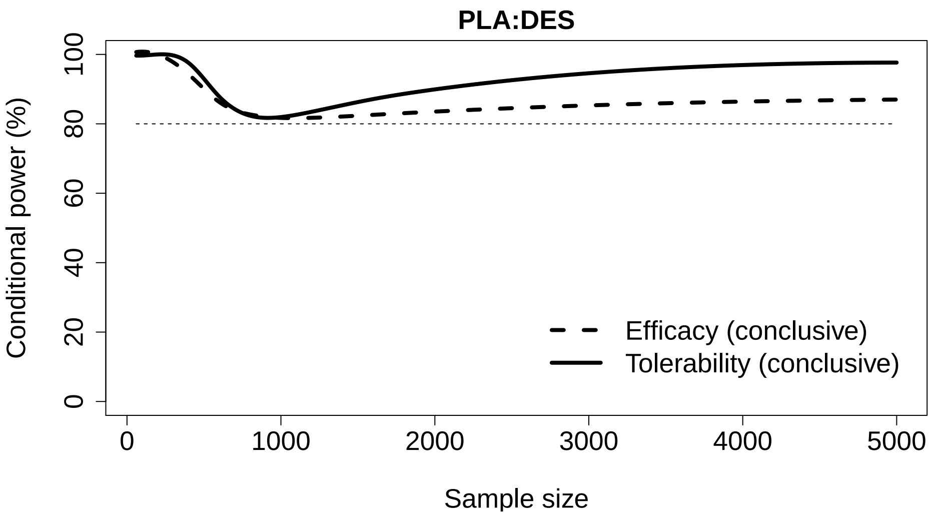

Figure 7:

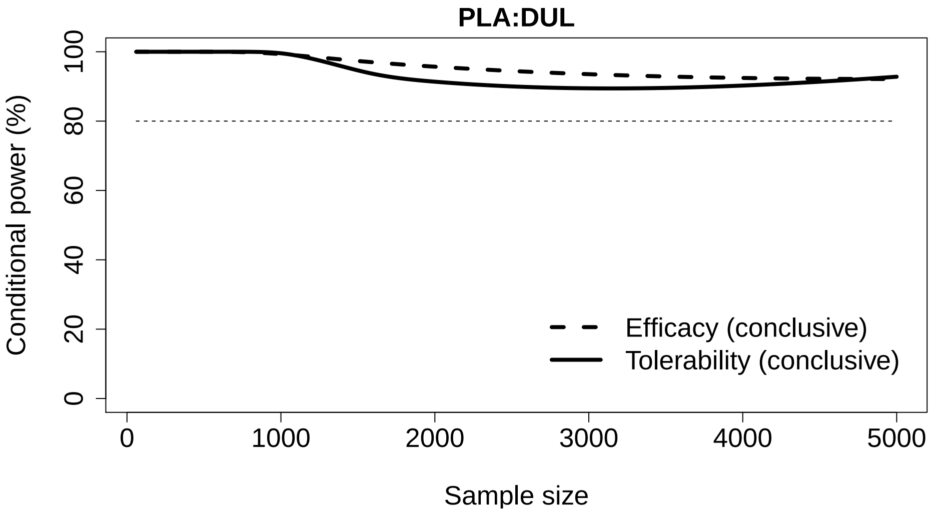

Figure 8:

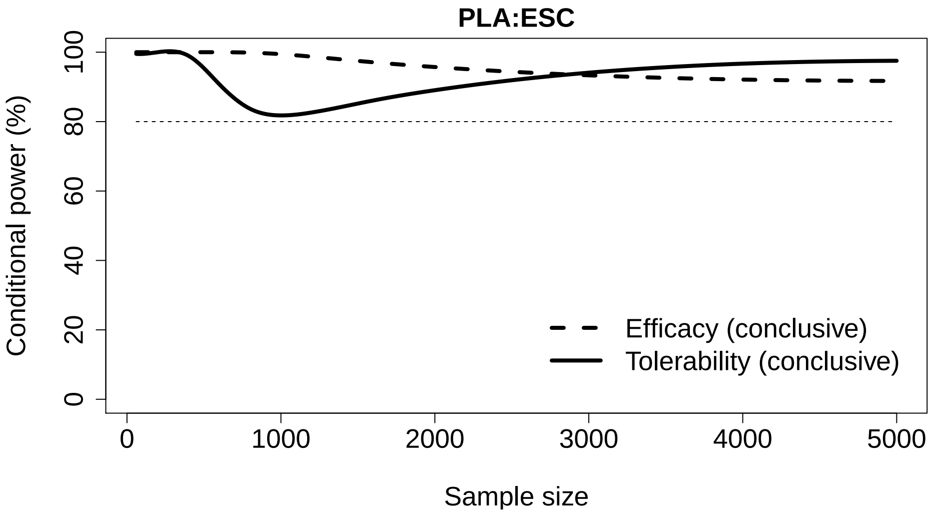

Figure 9:

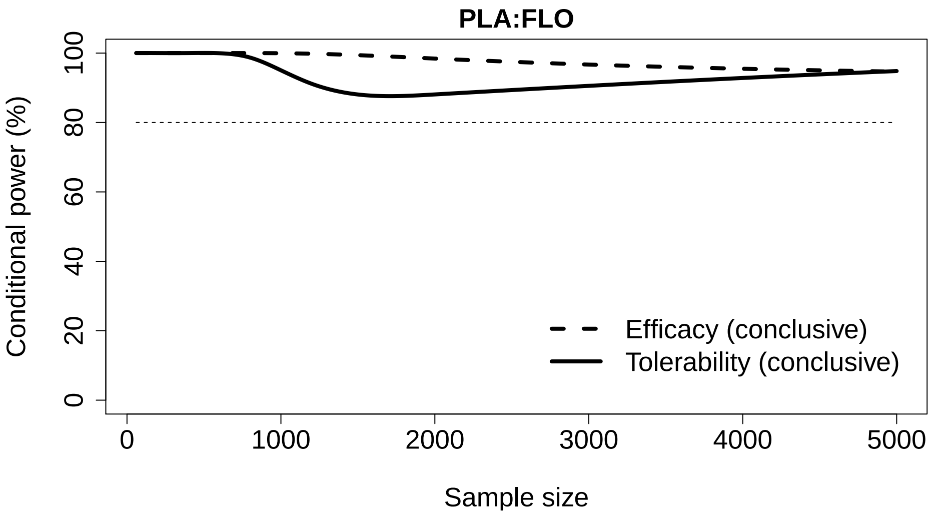

Figure 10:

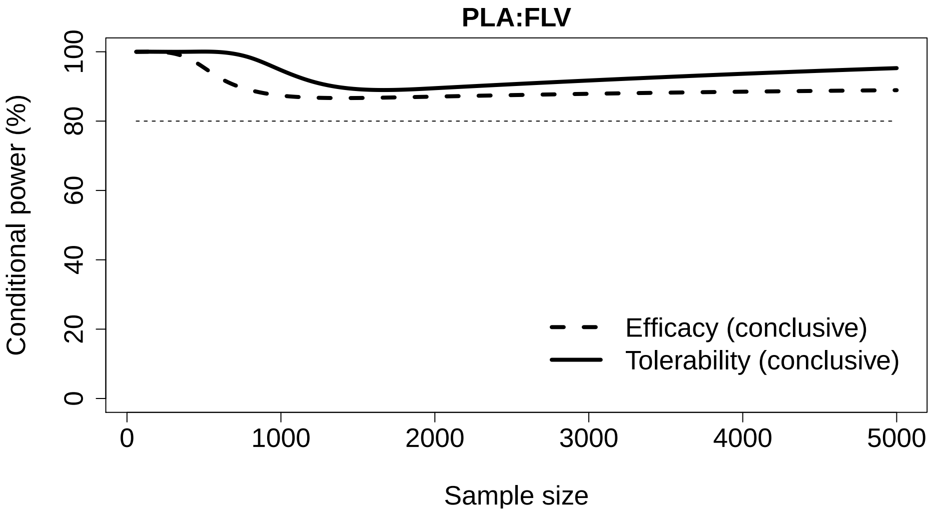

Figure 11:

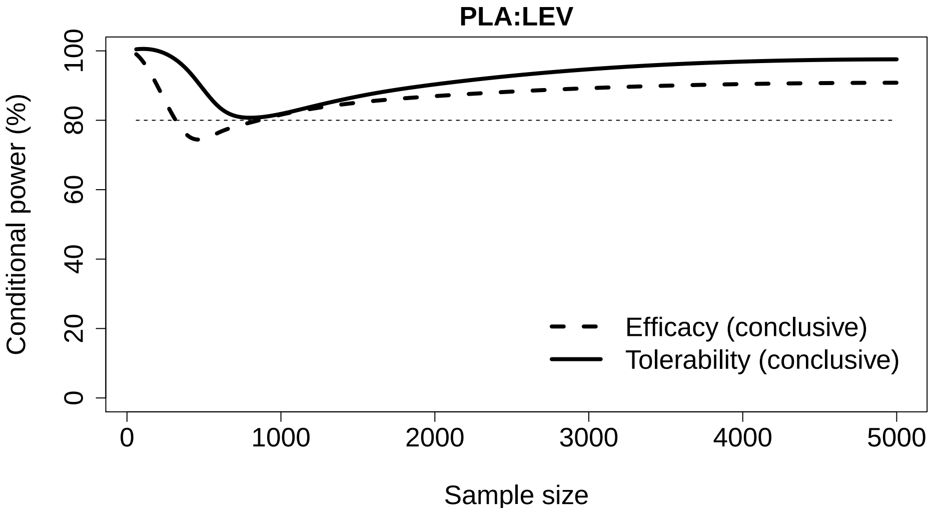

Figure 12:

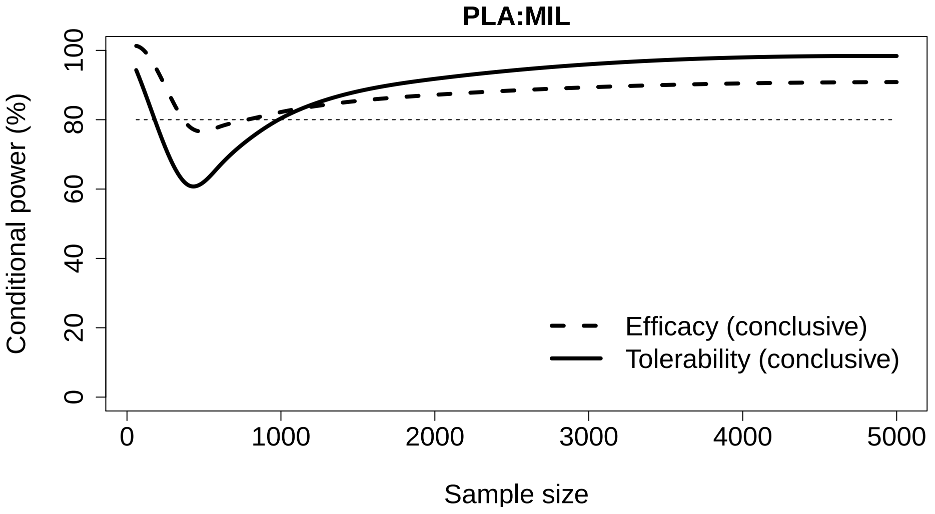

Figure 13:

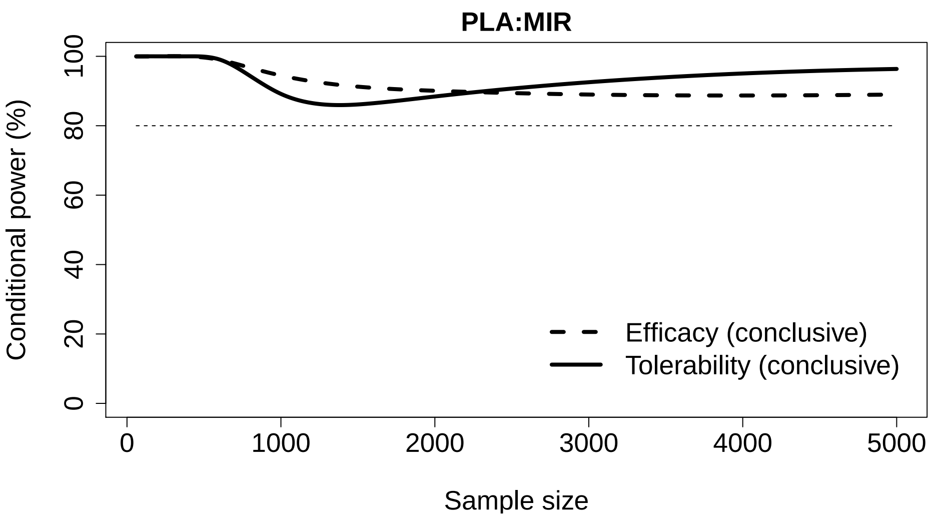

Figure 14:

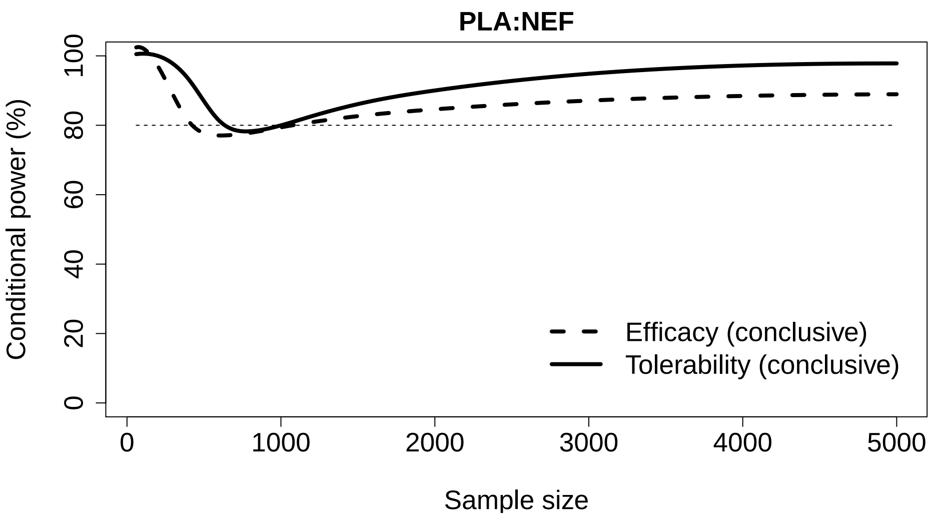

Figure 15:

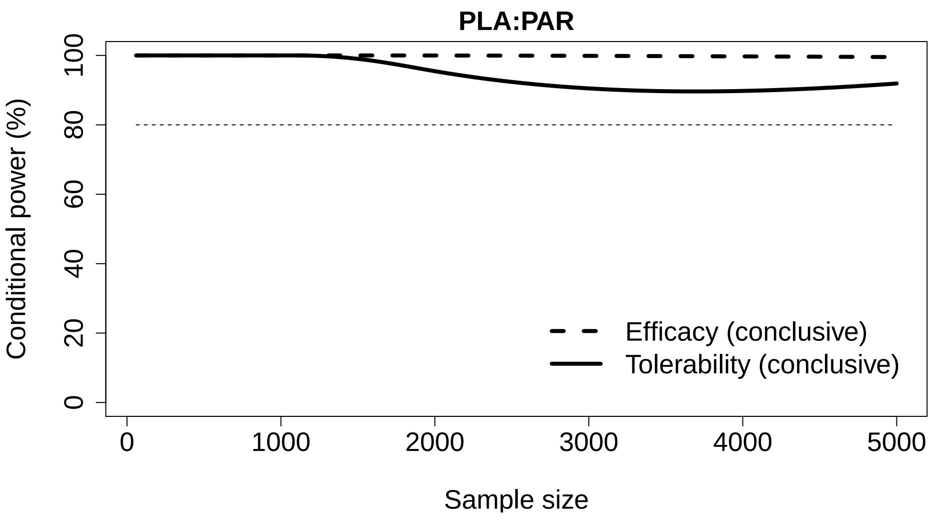

Figure 16:

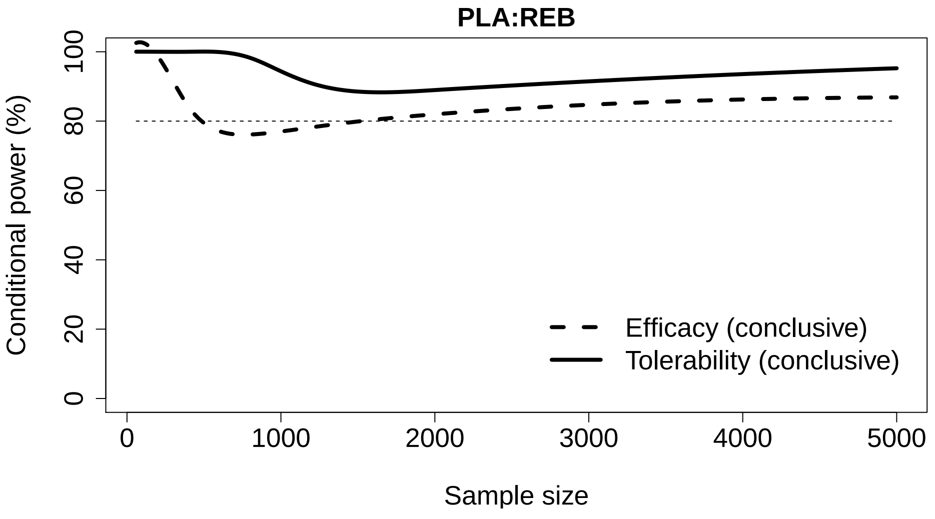

Figure 17:

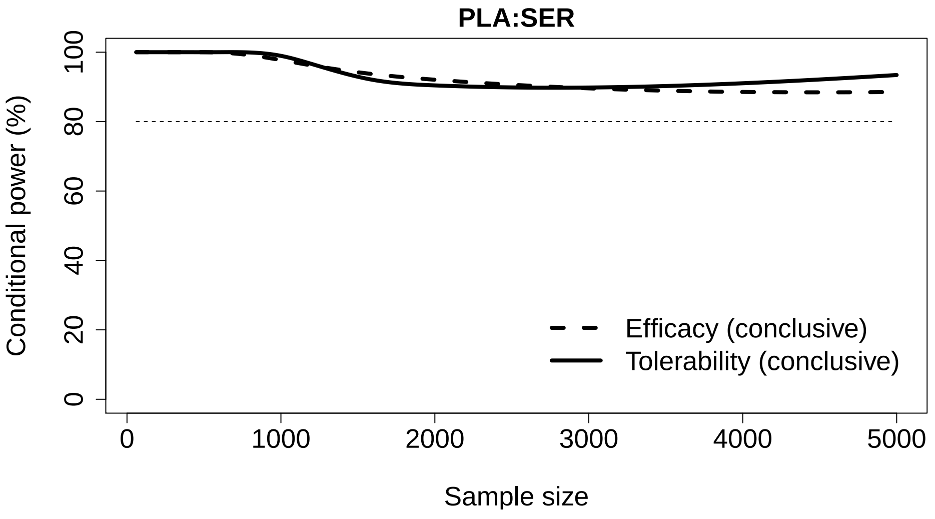

Figure 18:

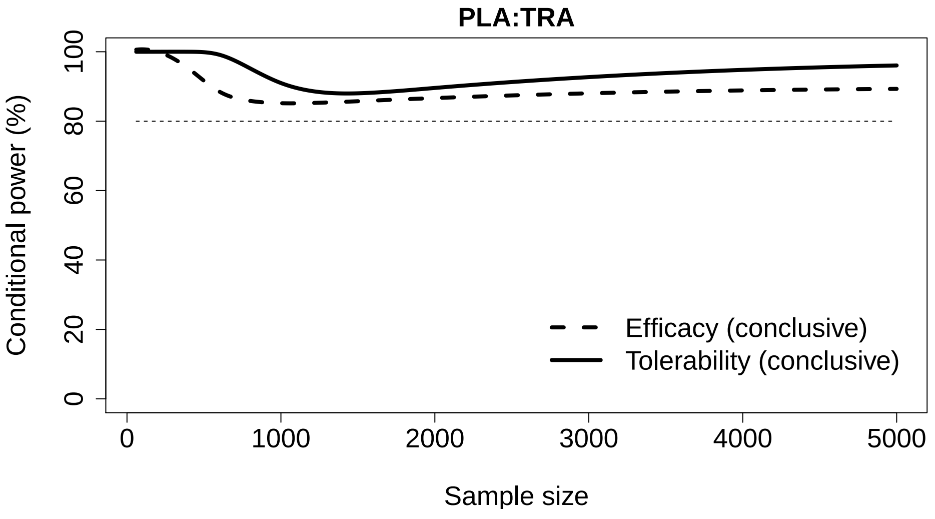

Figure 19:

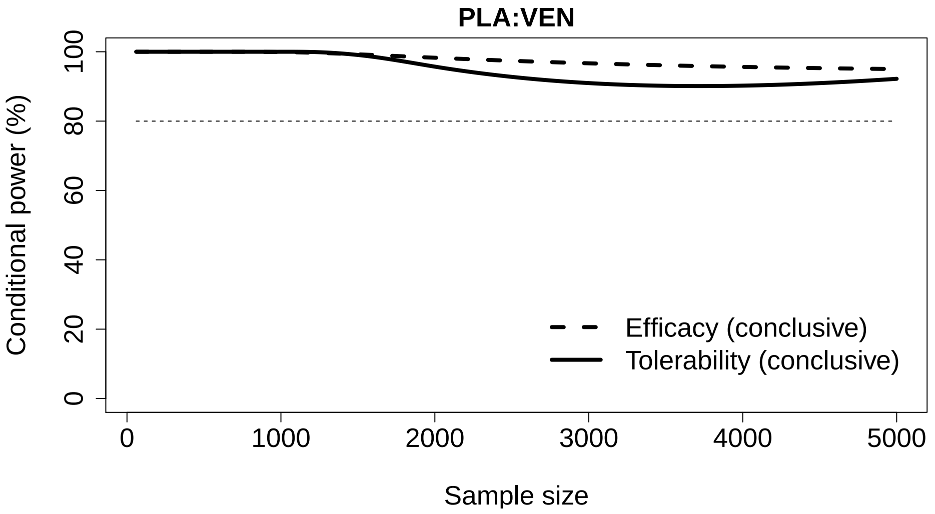

Figure 20:

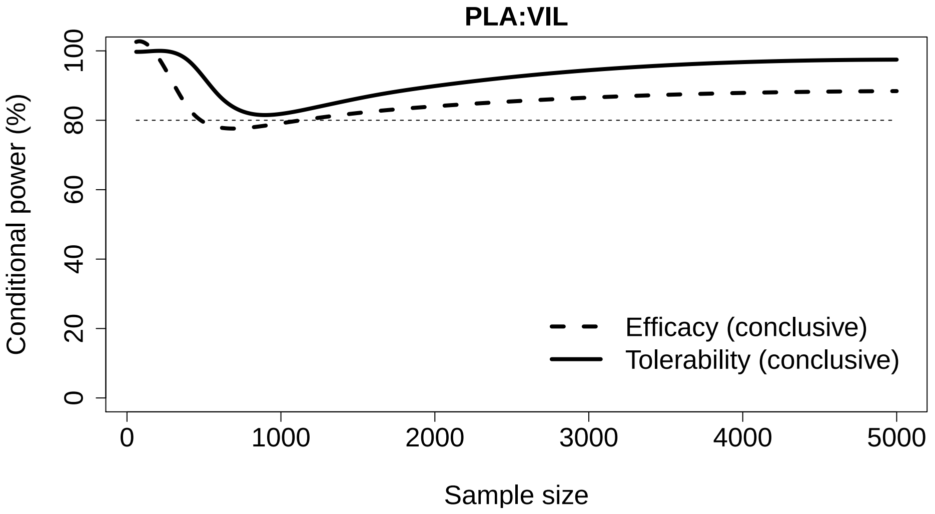

Figure 21:

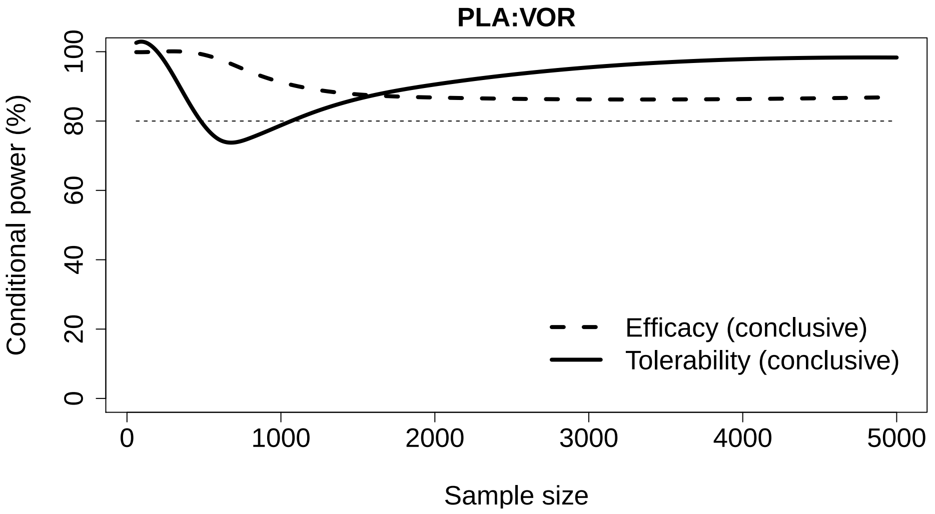

Figure 22:

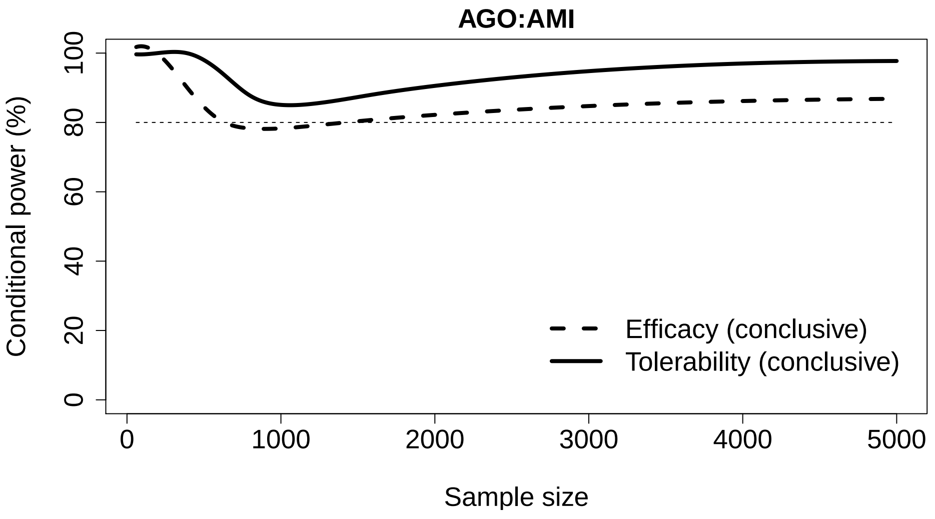

Figure 23:

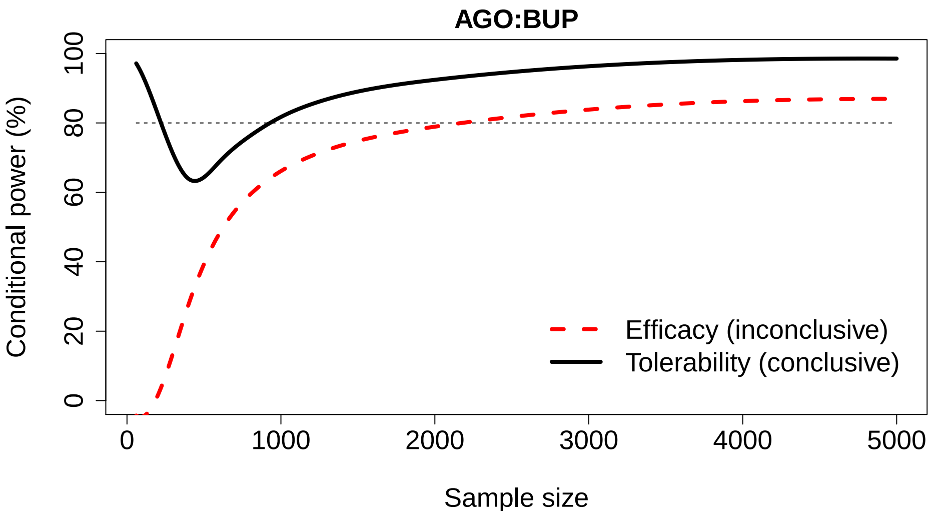

Figure 24:

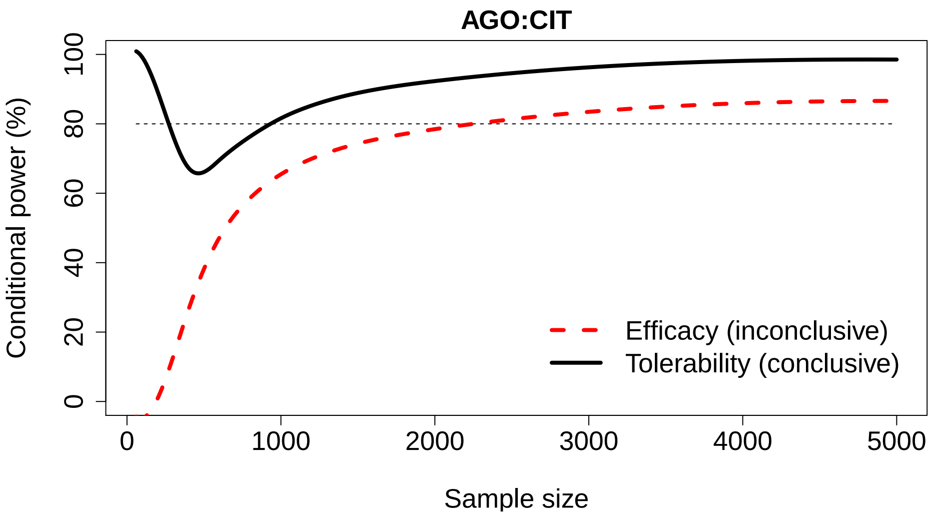

Figure 25:

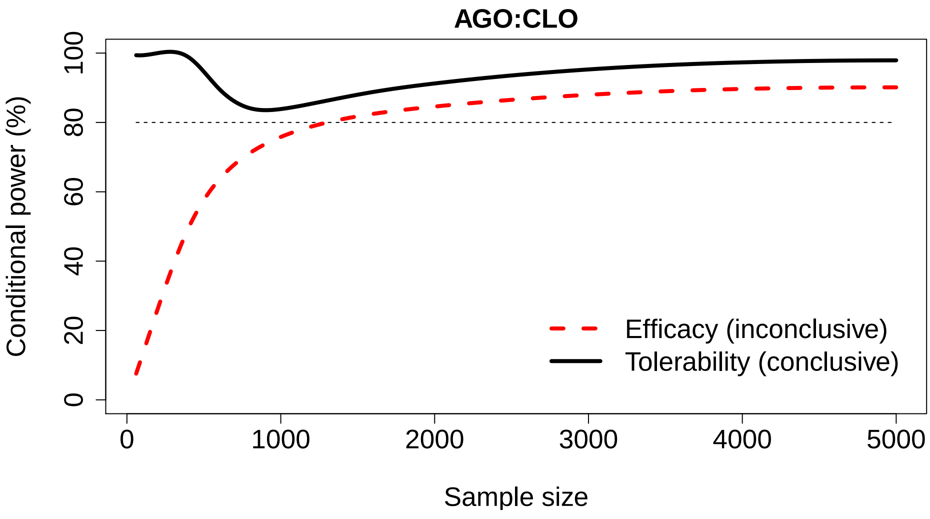

Figure 26:

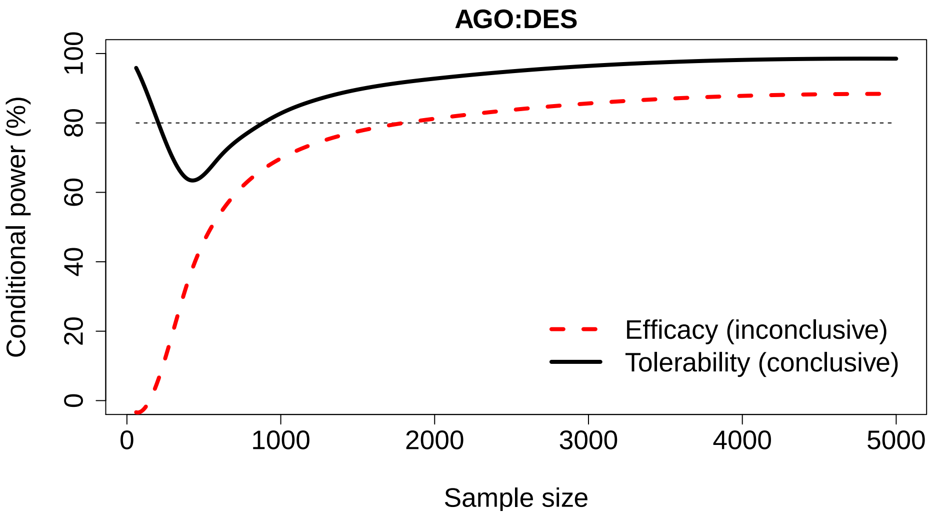

Figure 27:

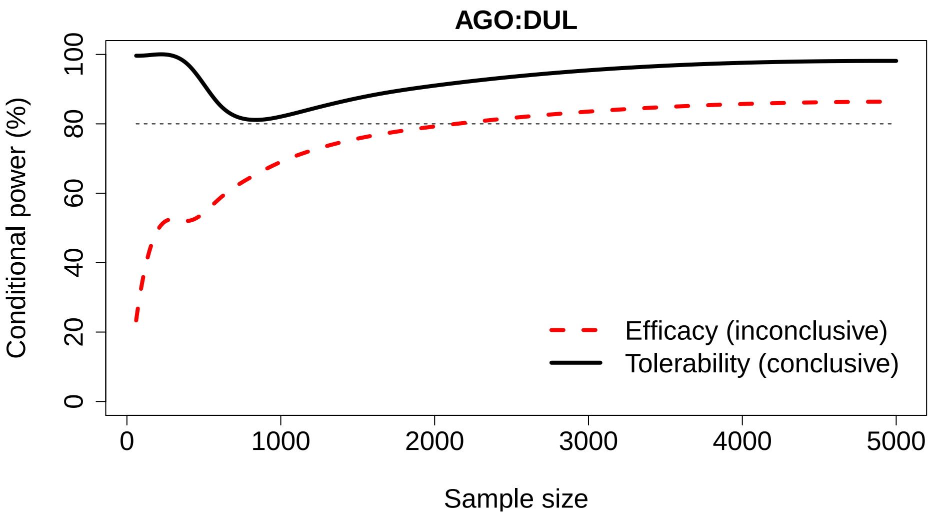

Figure 28:

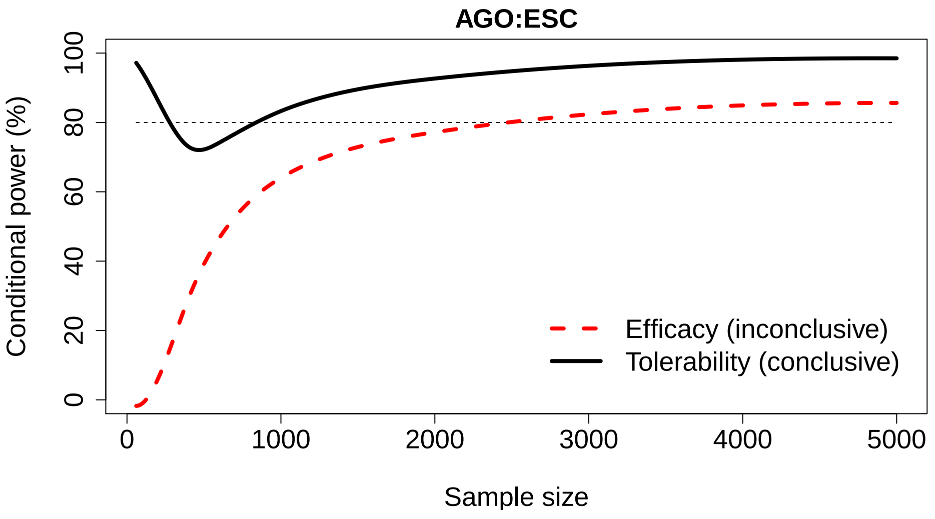

Figure 29:

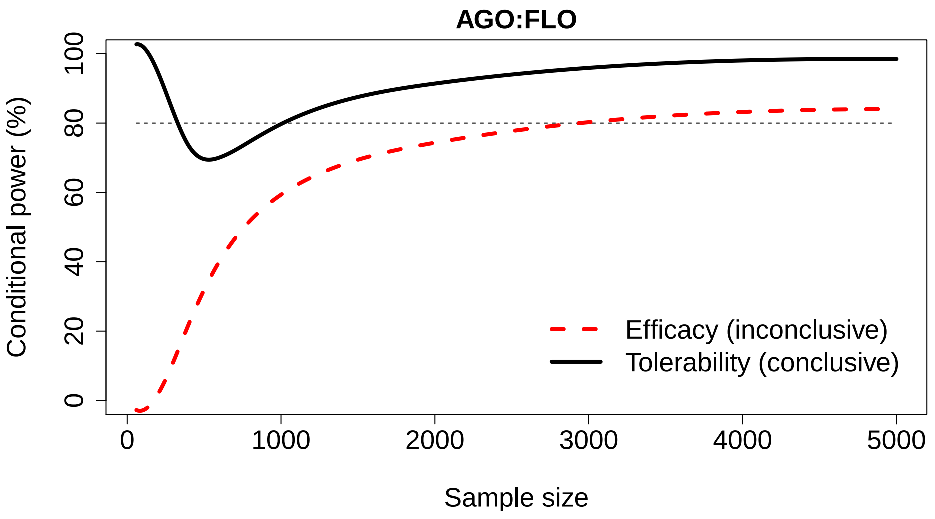

Figure 30:

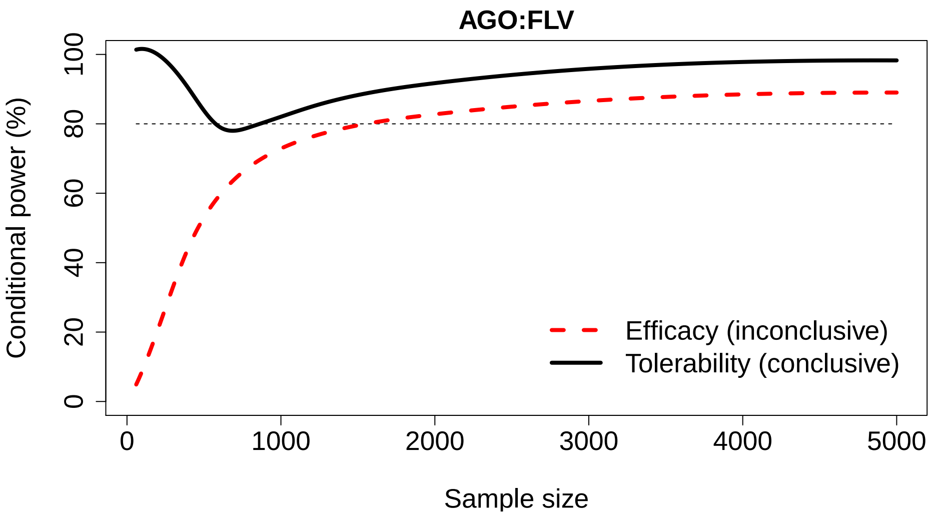

Figure 31:

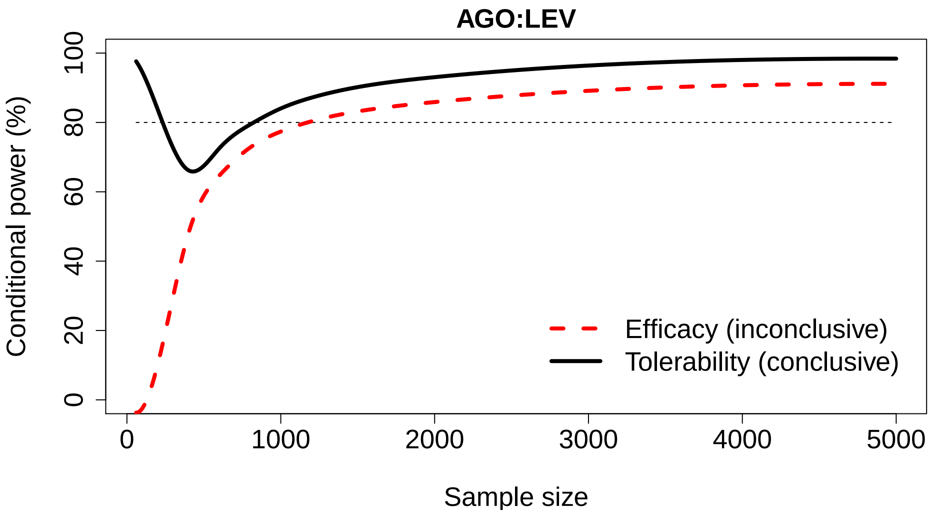

Figure 32:

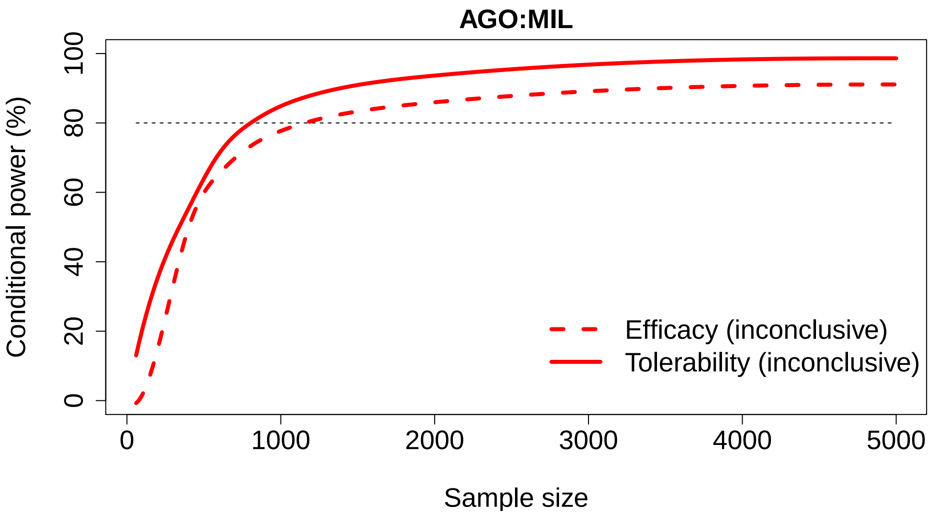

Figure 33:

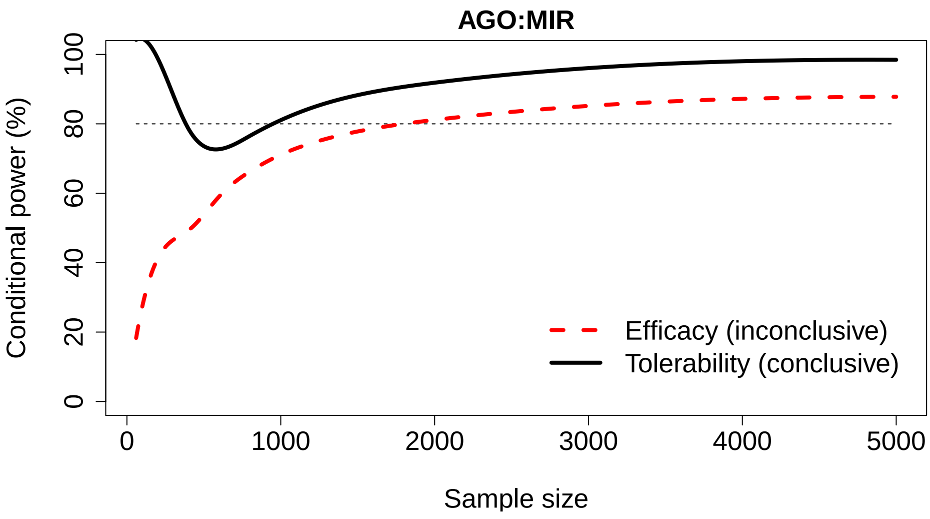

Figure 34:

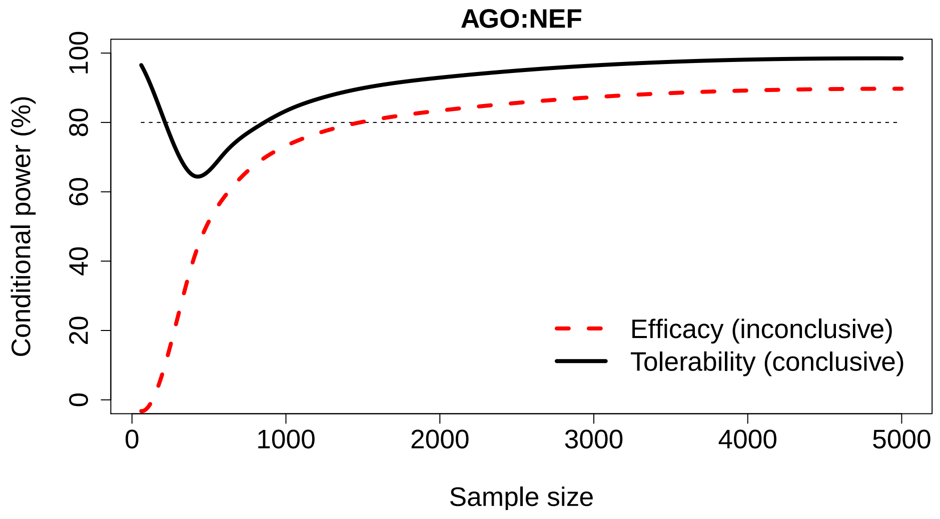

Figure 35:

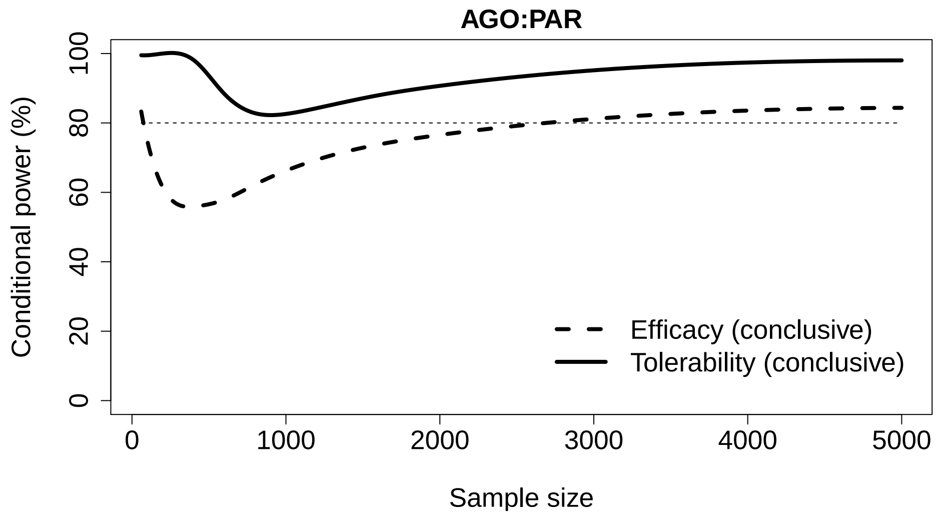

Figure 36:

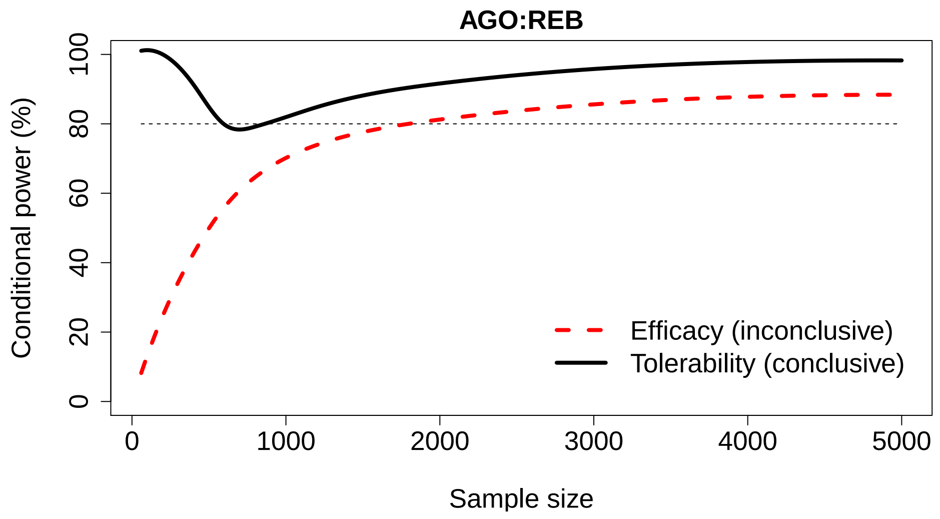

Figure 37:

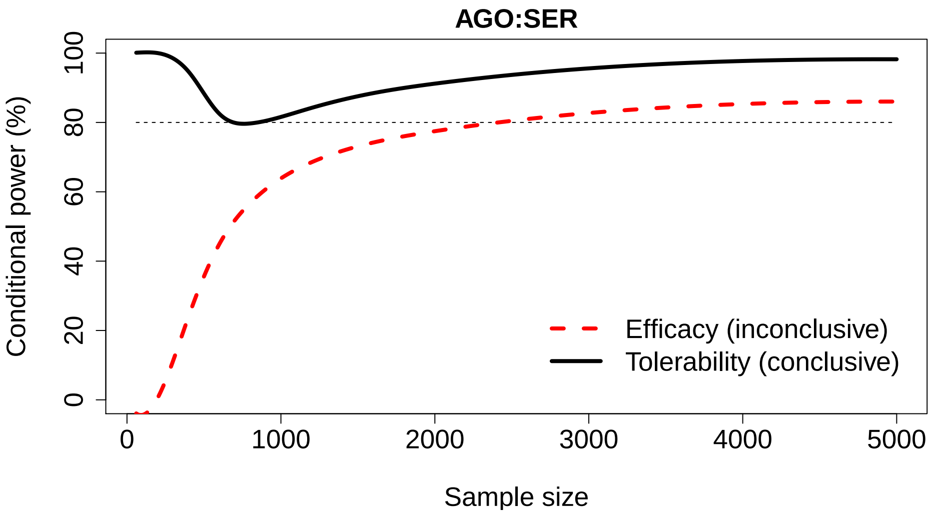

Figure 38:

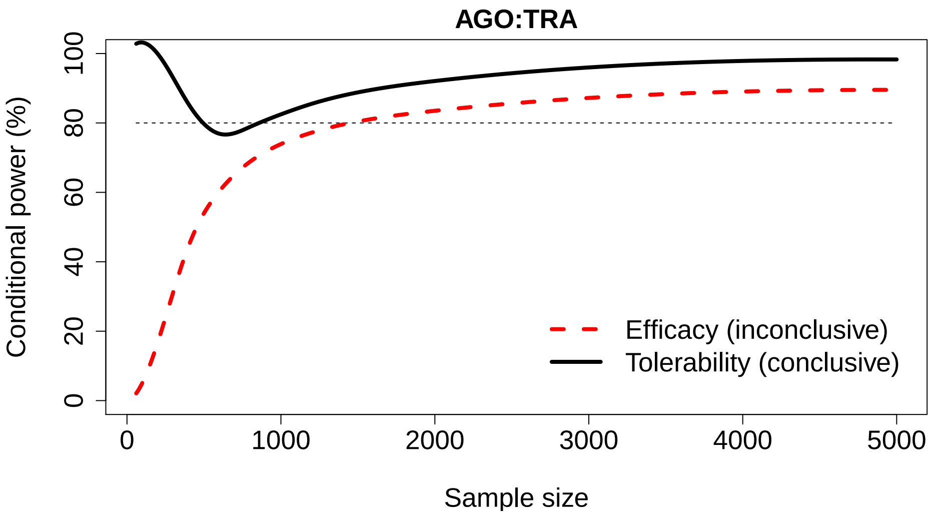

Figure 39:

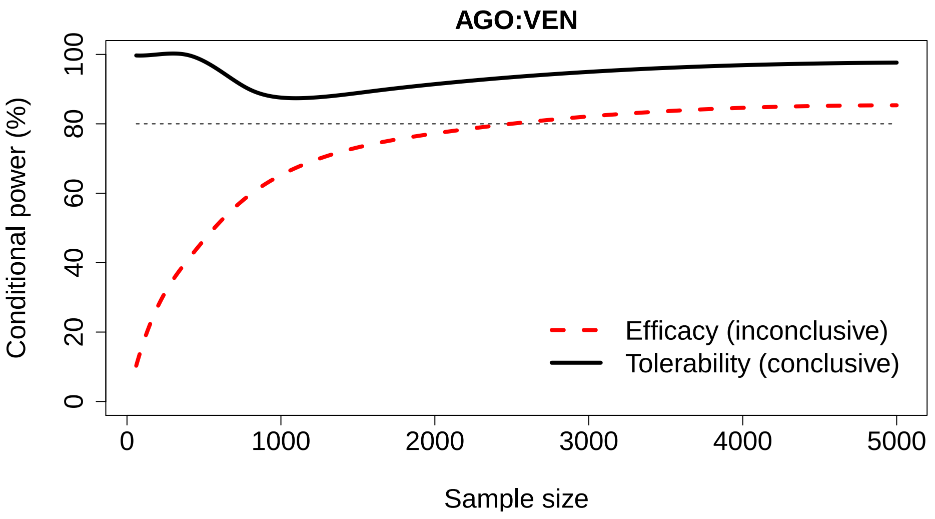

Figure 40:

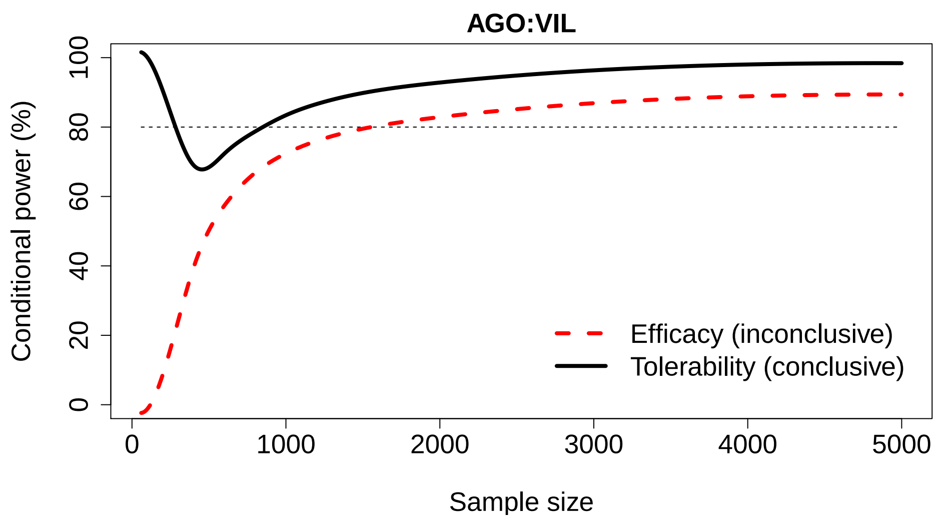

Figure 41:

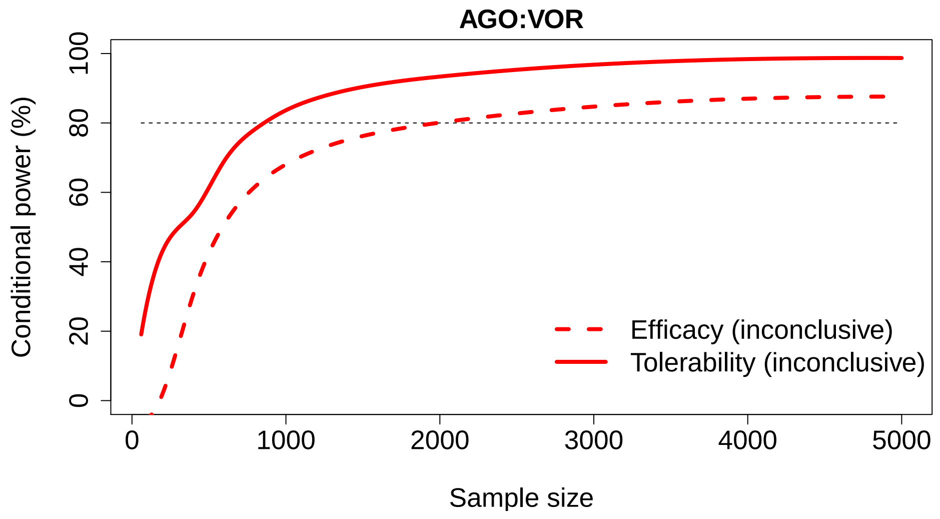

Figure 42:

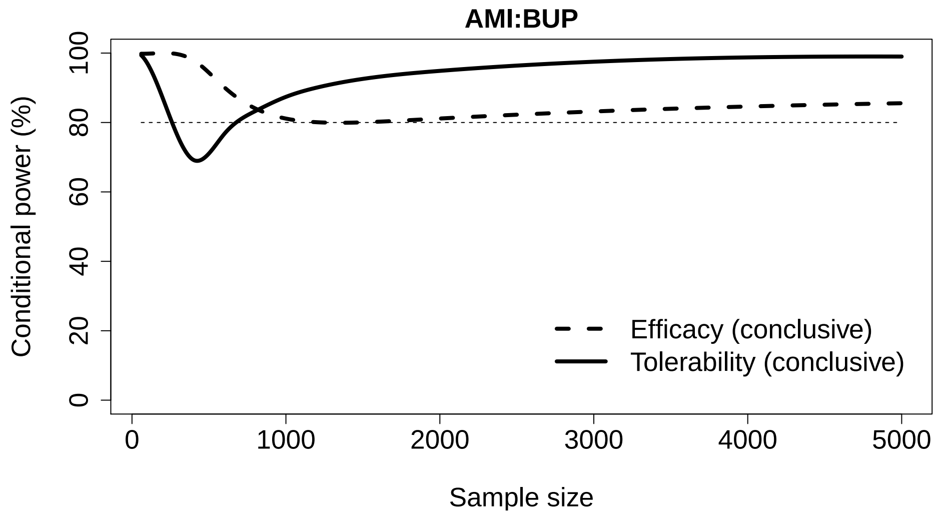

Figure 43:

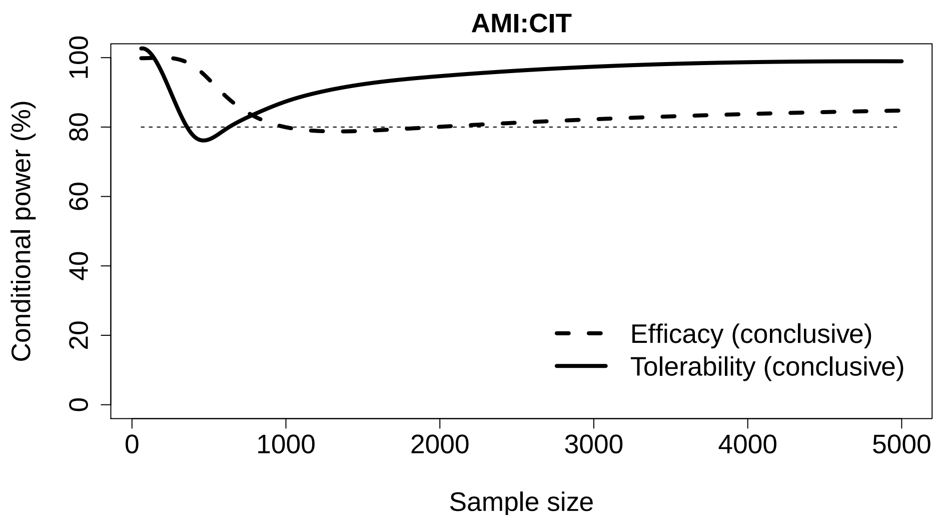

Figure 44:

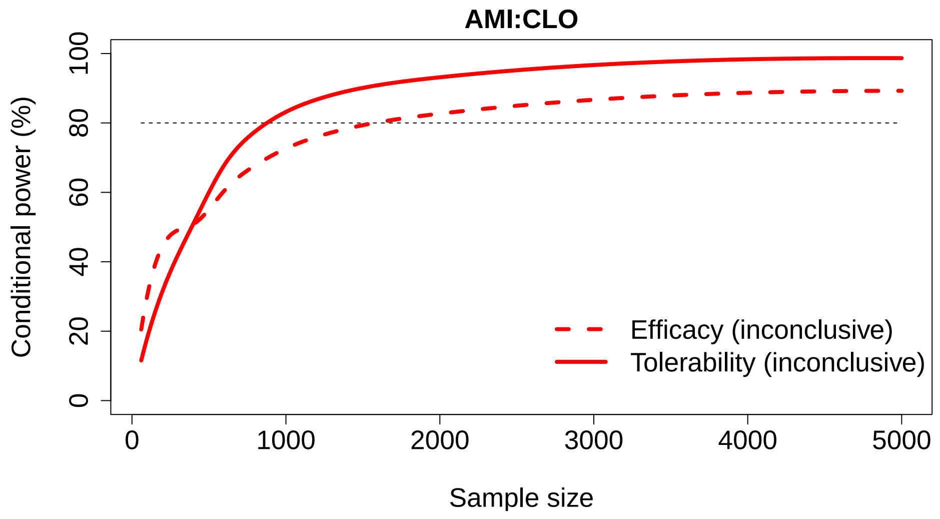

Figure 45:

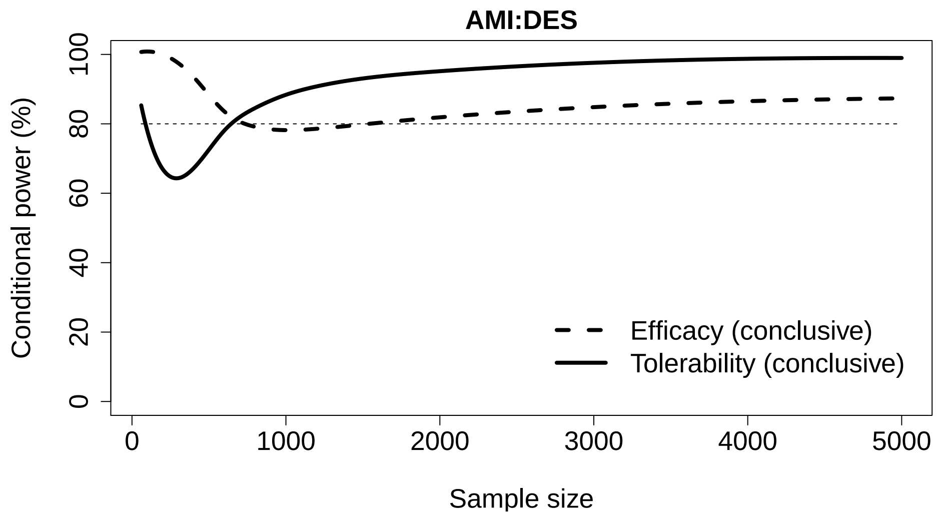

Figure 46:

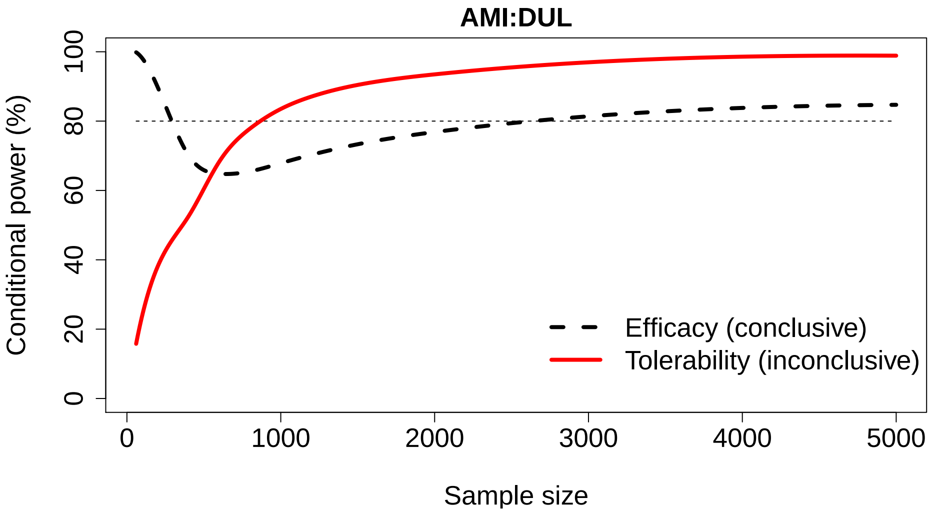

Figure 47:

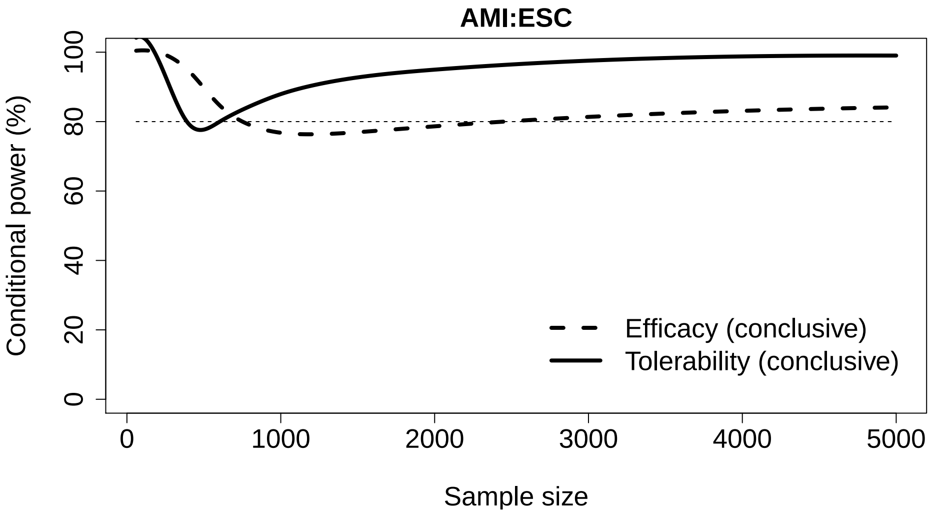

Figure 48:

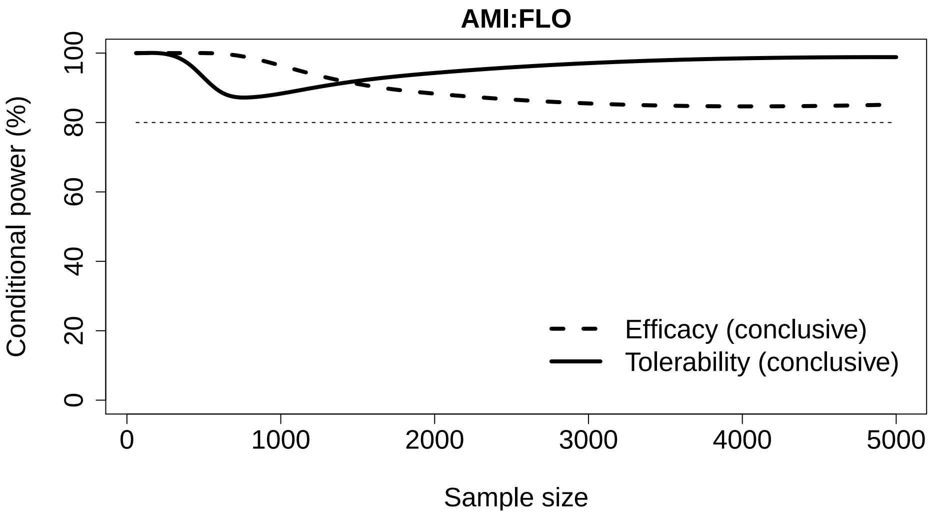

Figure 49:

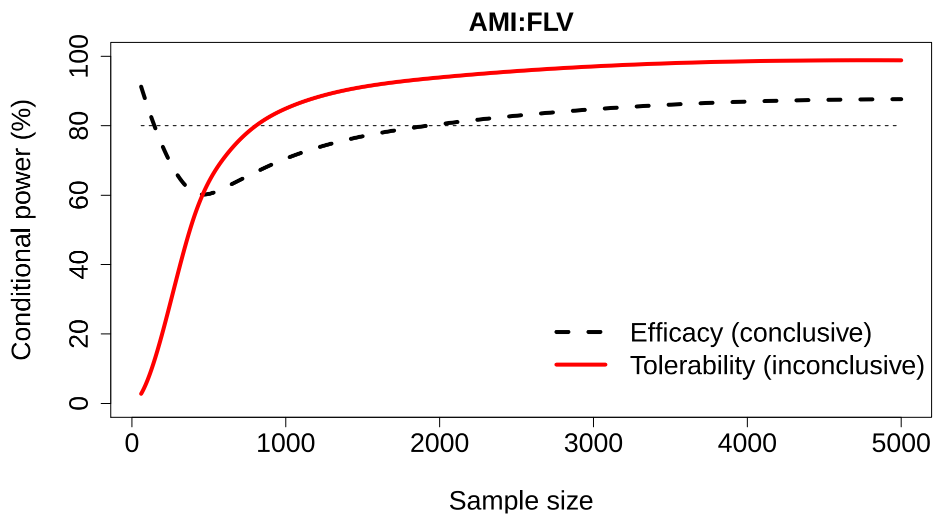

Figure 50:

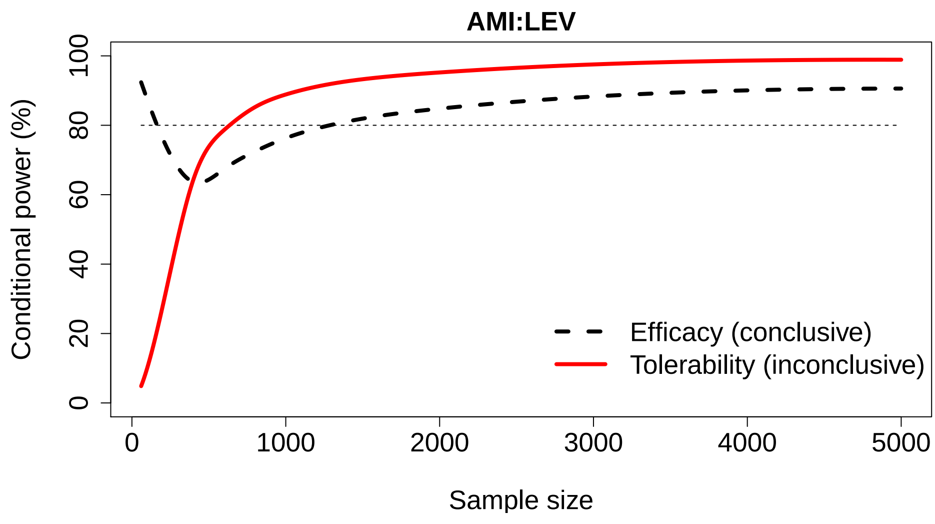

Figure 51:

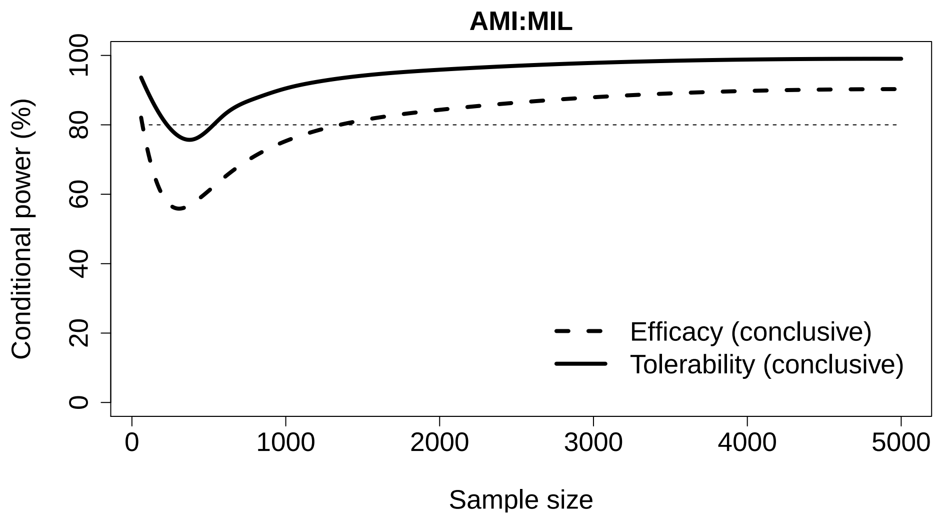

Figure 52:

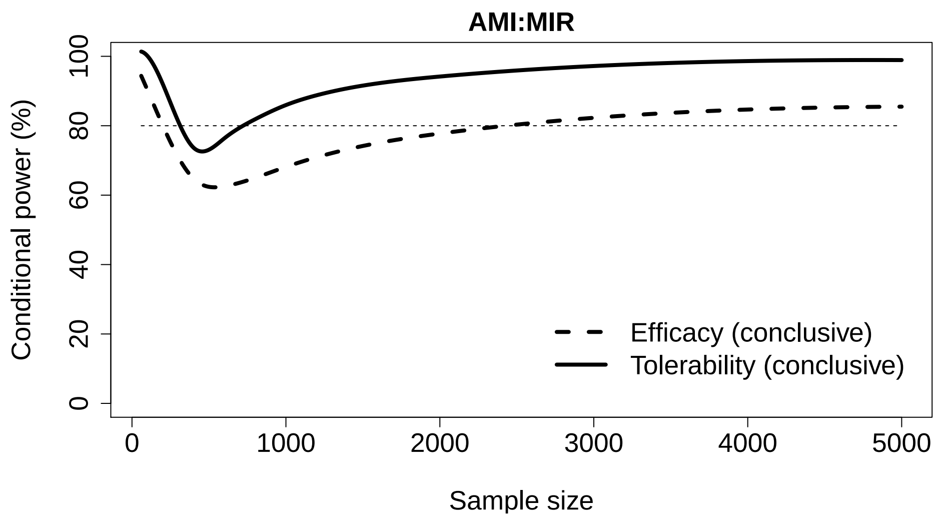

Figure 53:

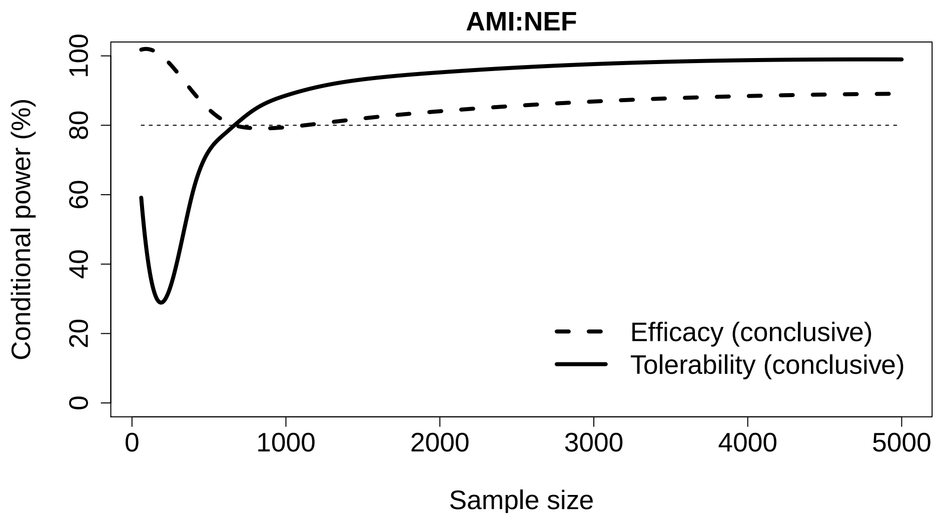

Figure 54:

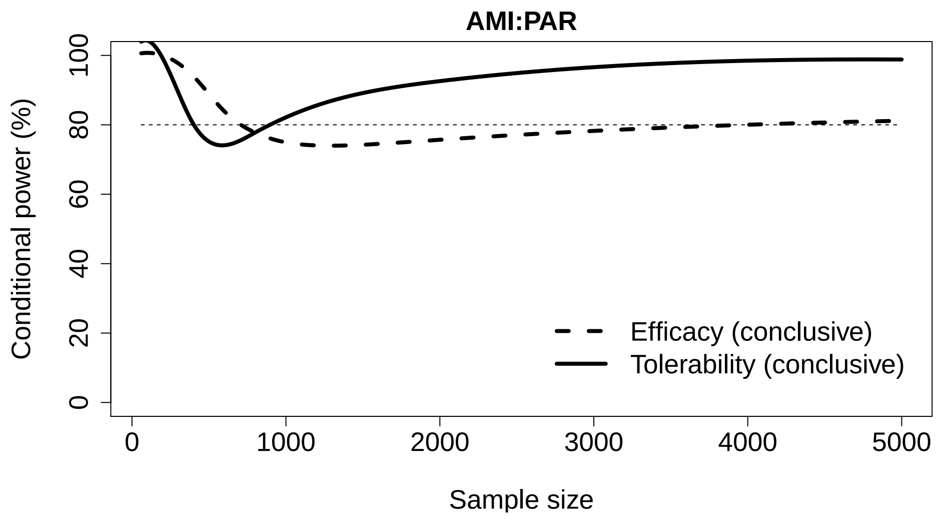

Figure 55:

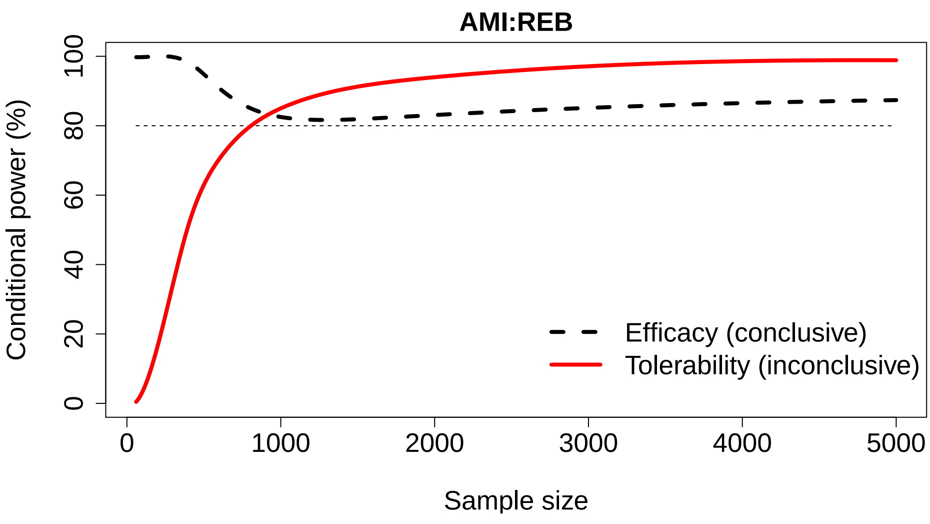

Figure 56:

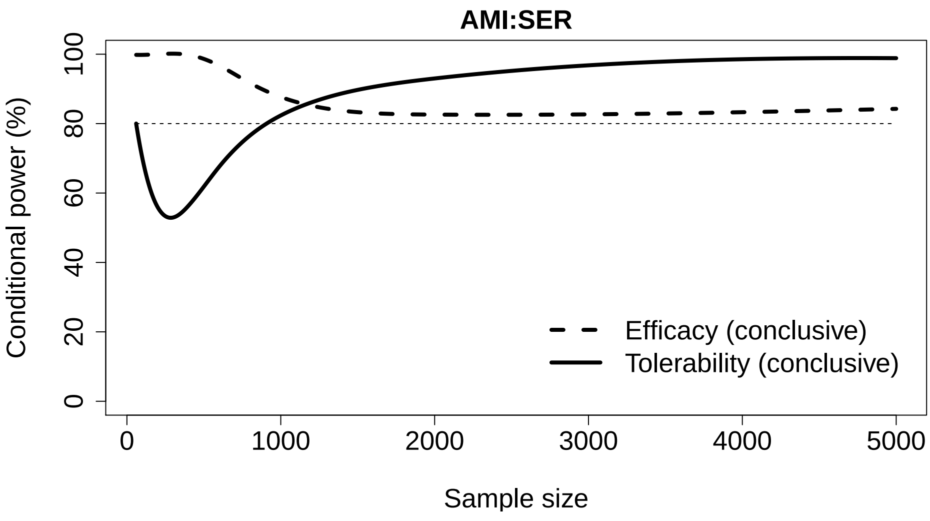

Figure 57:

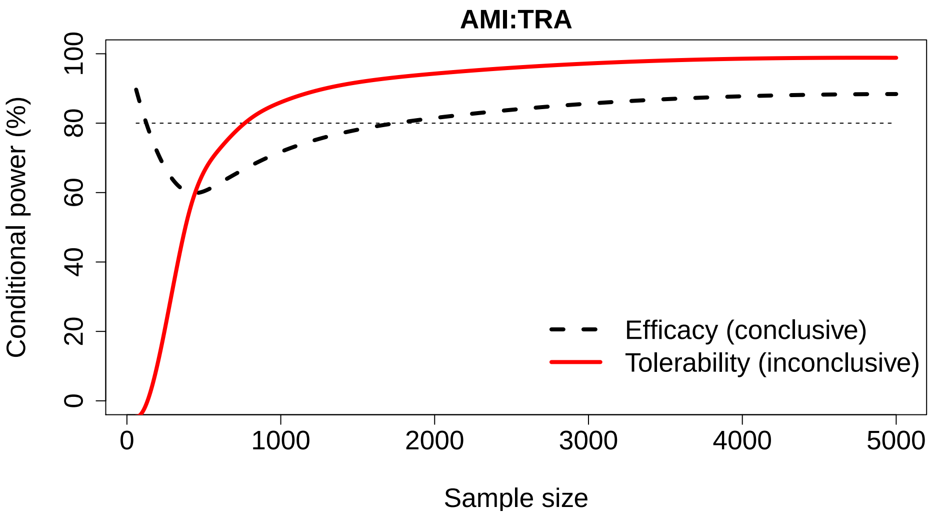

Figure 58:

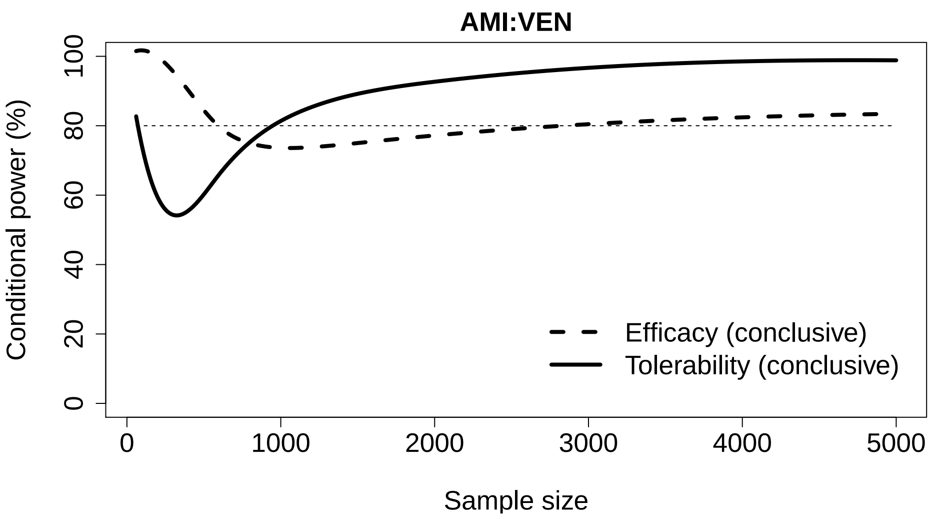

Figure 59:

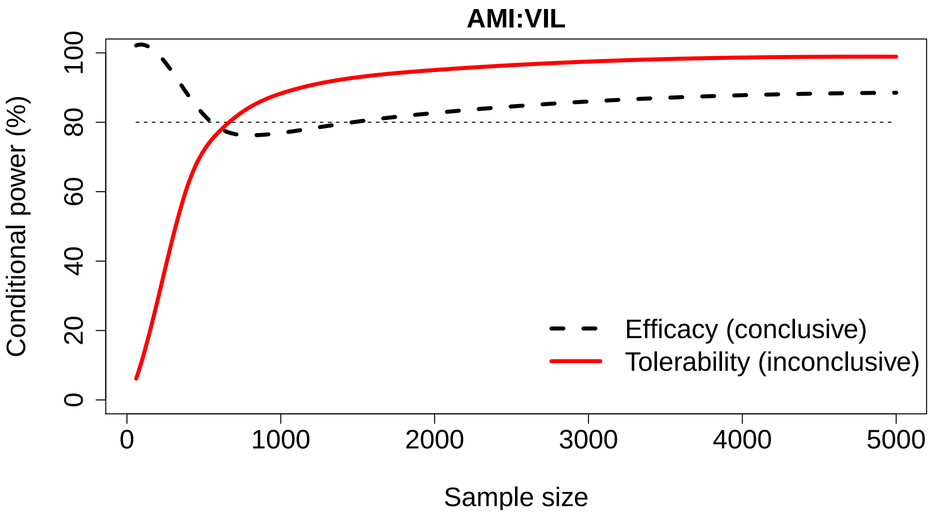

Figure 60:

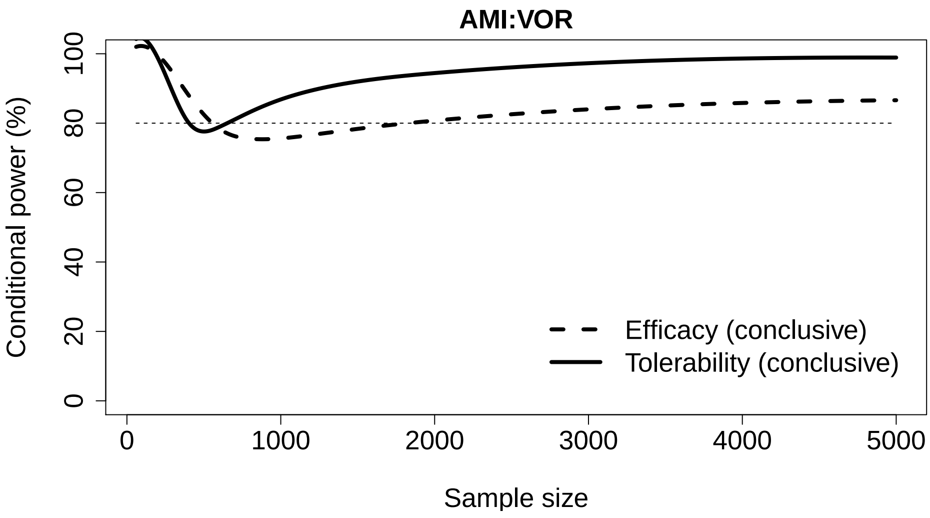

Figure 61:

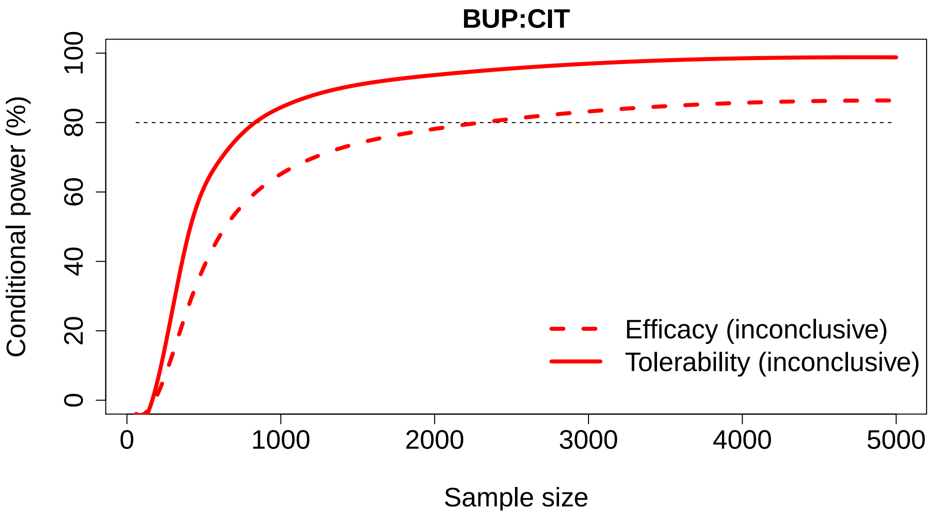

Figure 62:

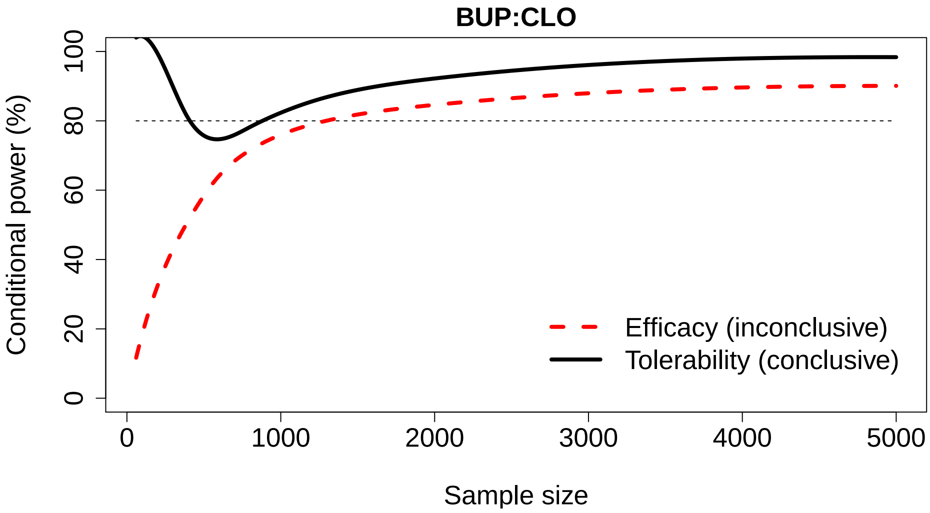

Figure 63:

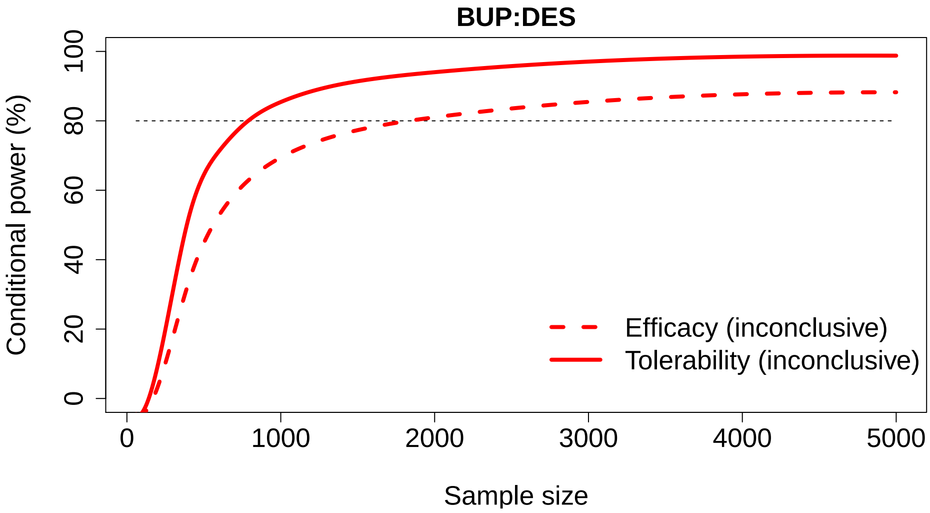

Figure 64:

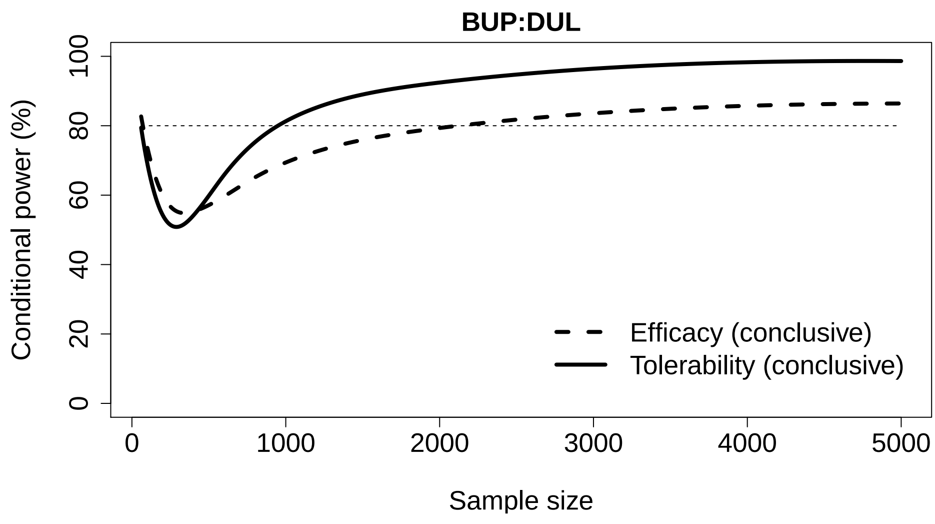

Figure 65:

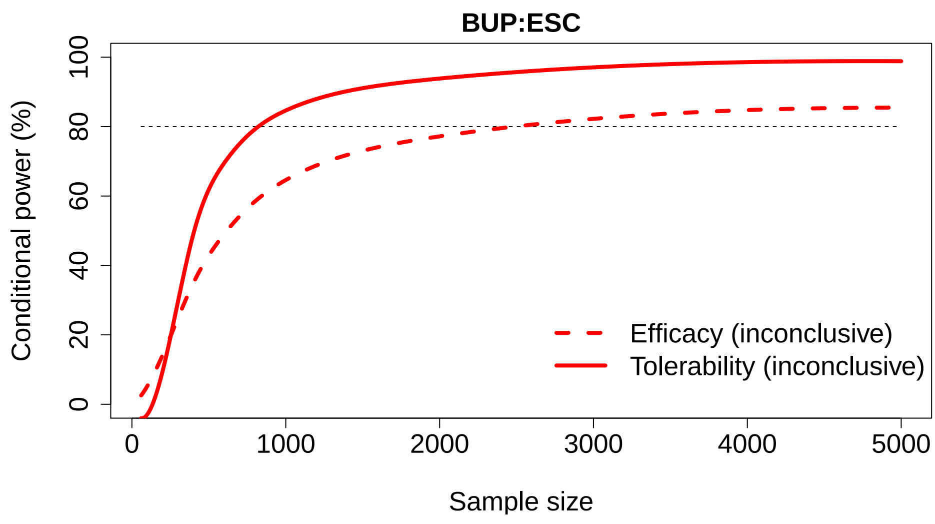

Figure 66:

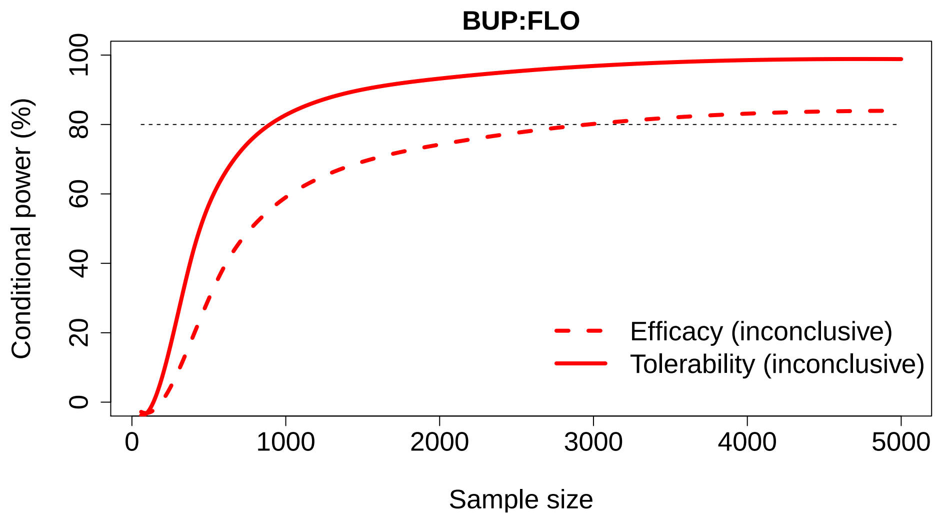

Figure 67:

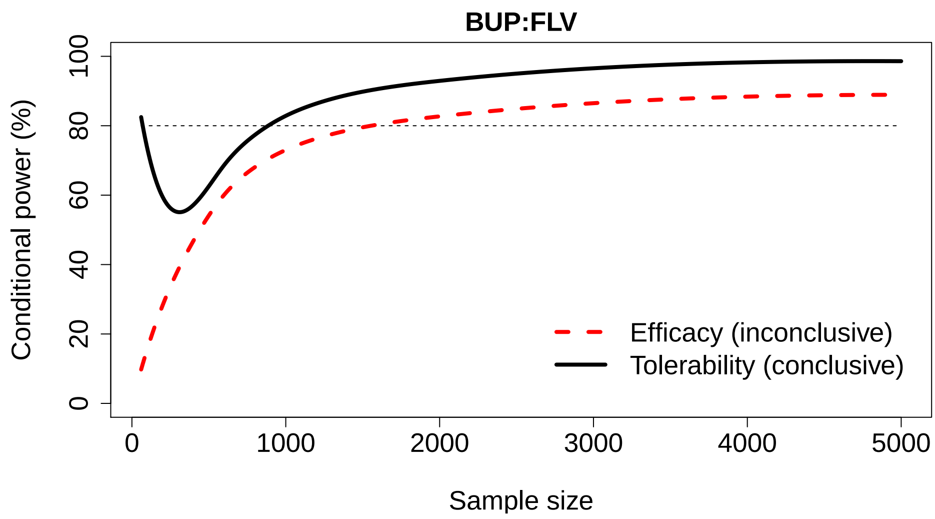

Figure 68:

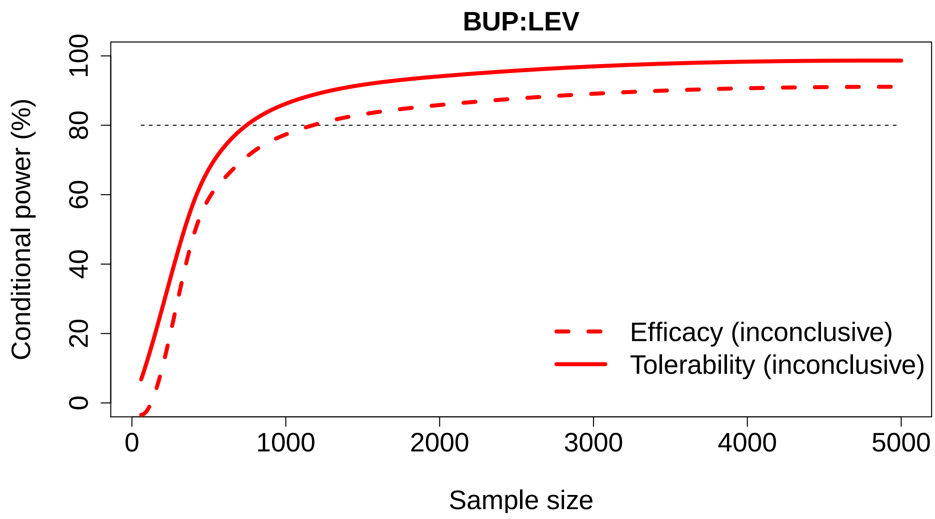

Figure 69:

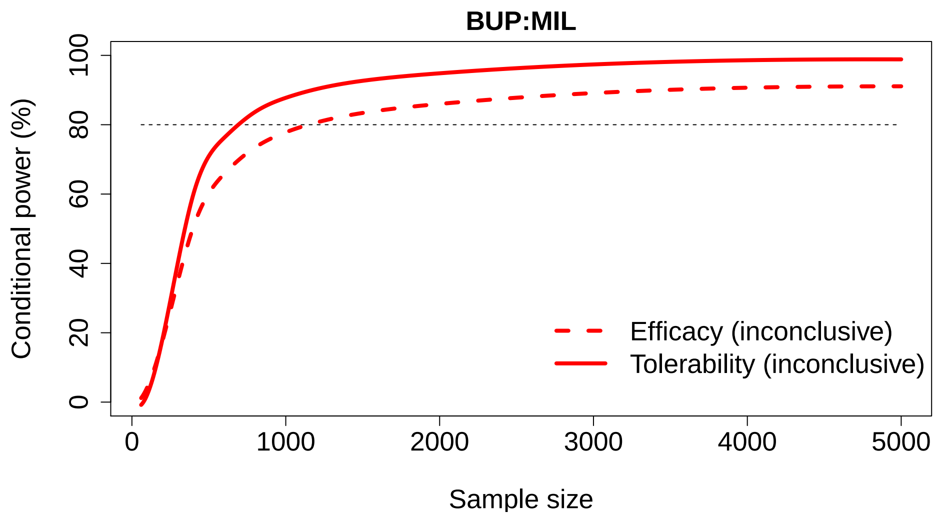

Figure 70:

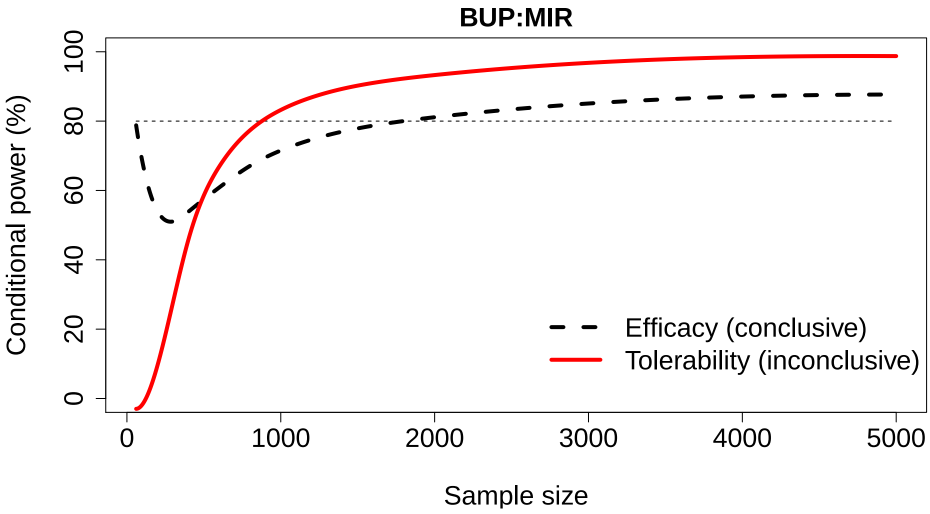

Figure 71:

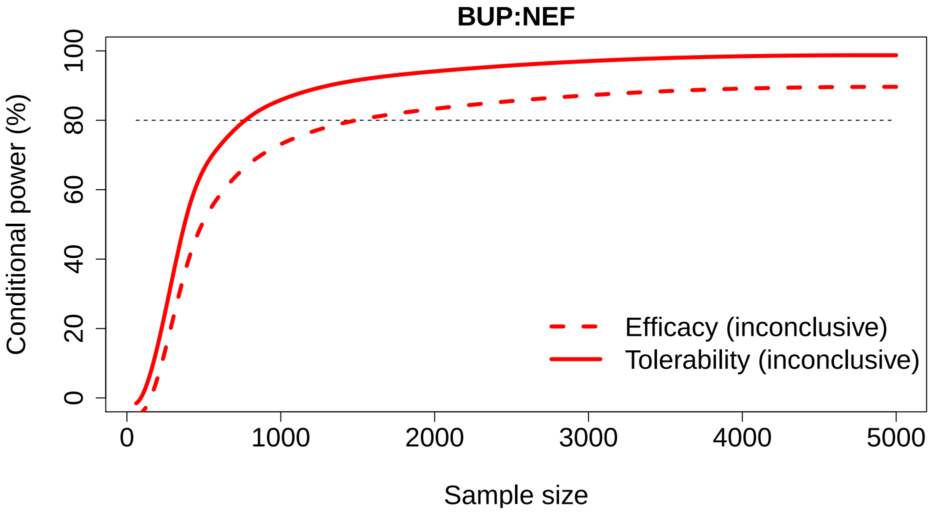

Figure 72:

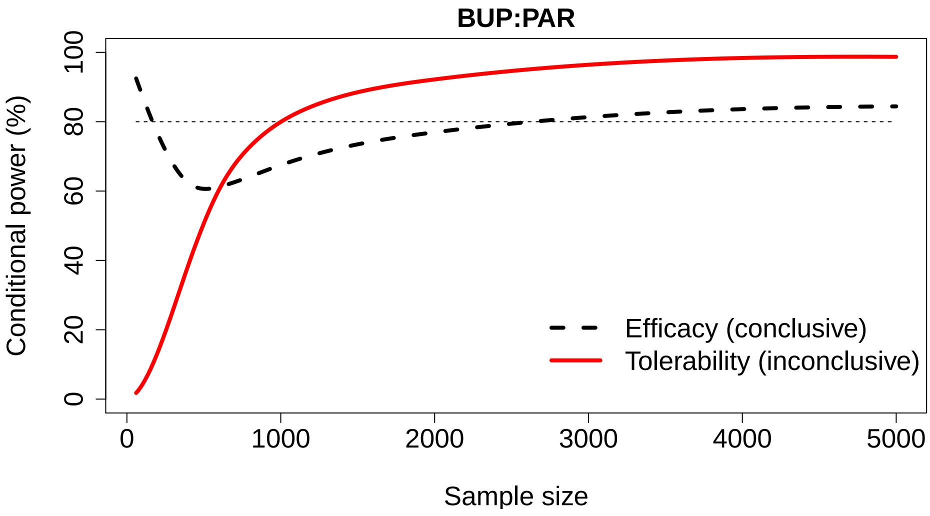

Figure 73:

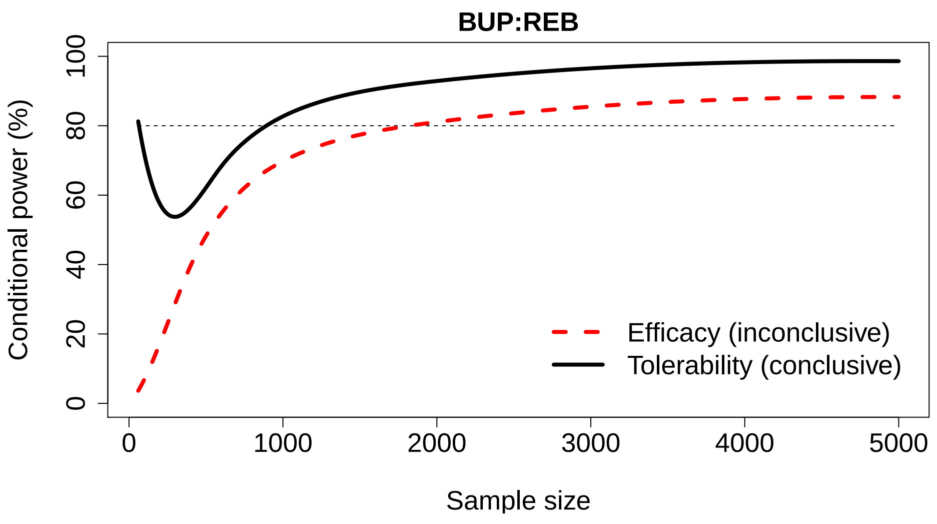

Figure 74:

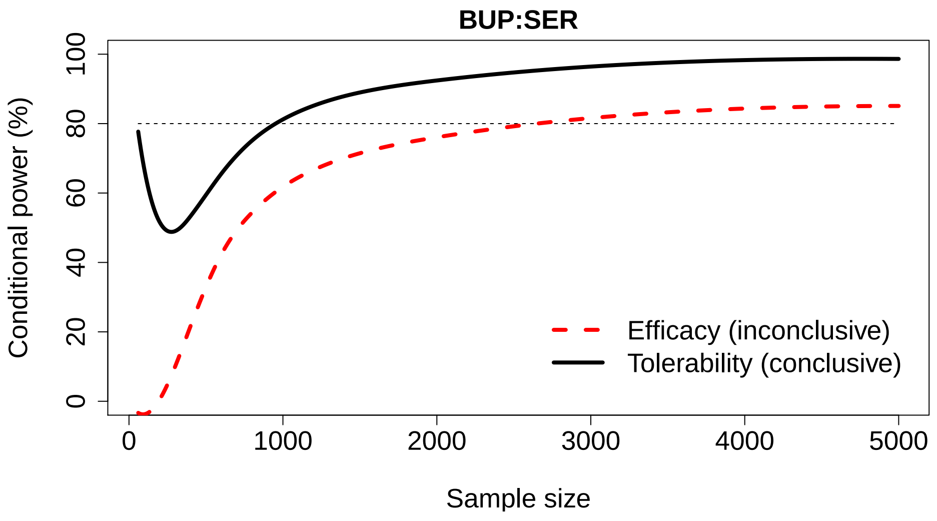

Figure 75:

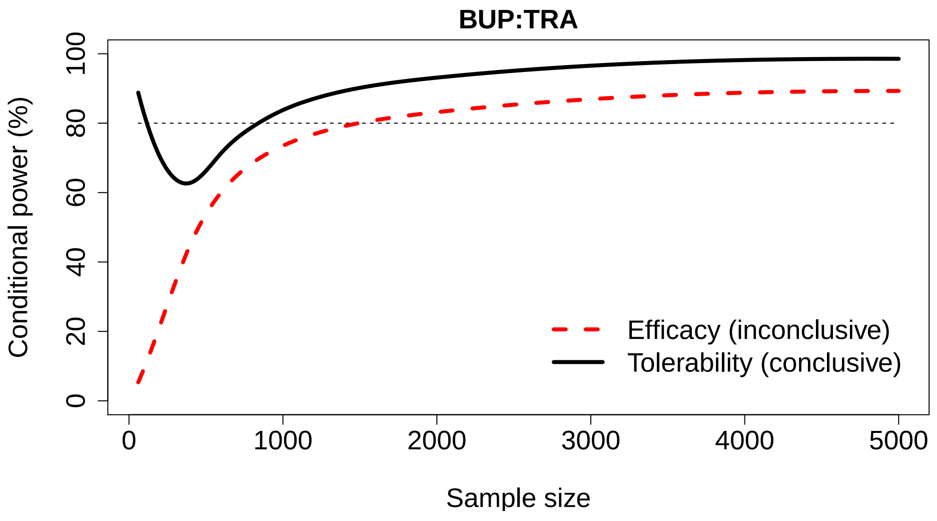

Figure 76:

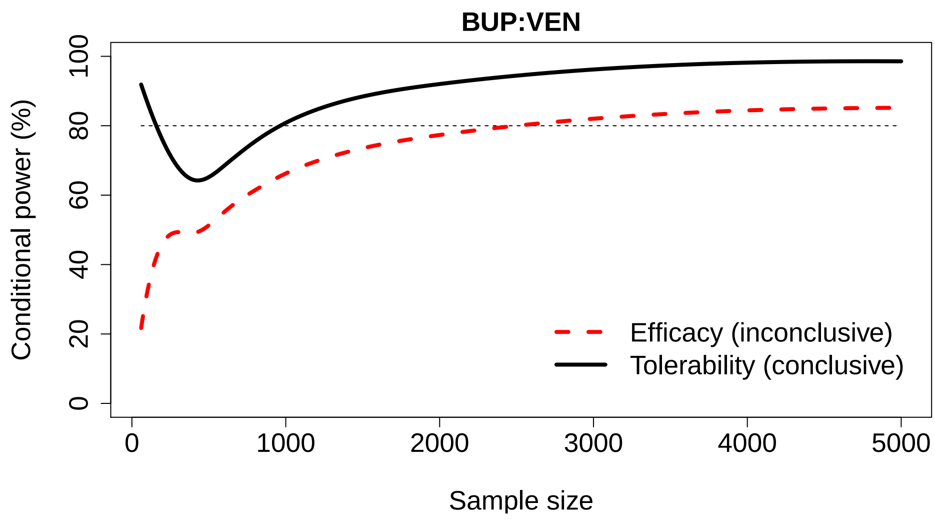

Figure 77:

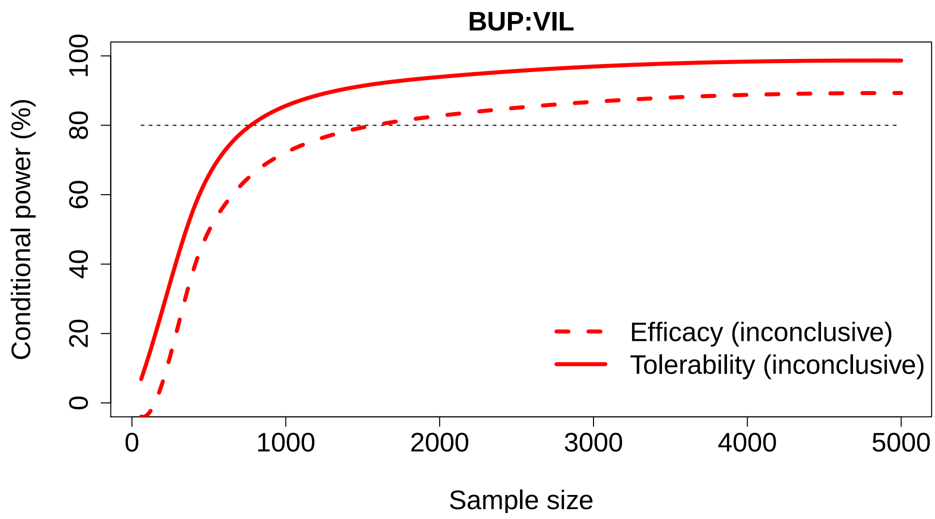

Figure 78:

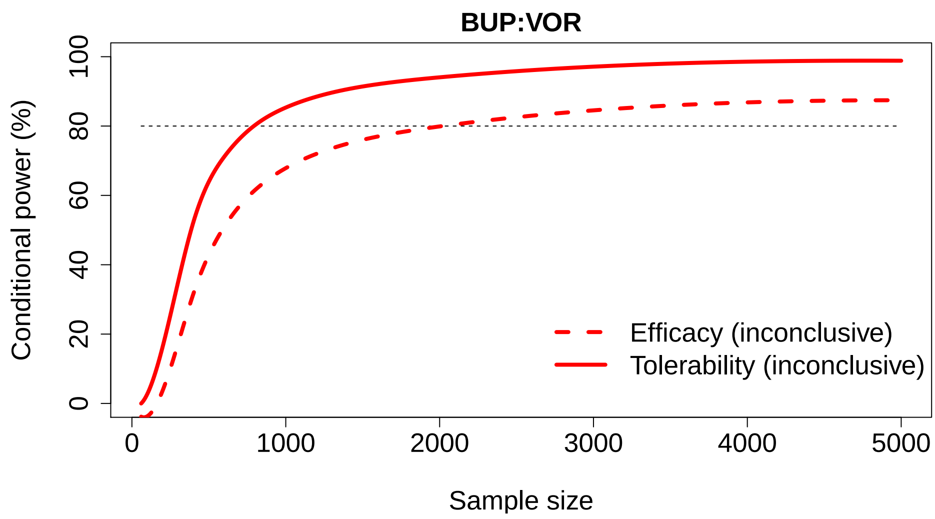

Figure 79:

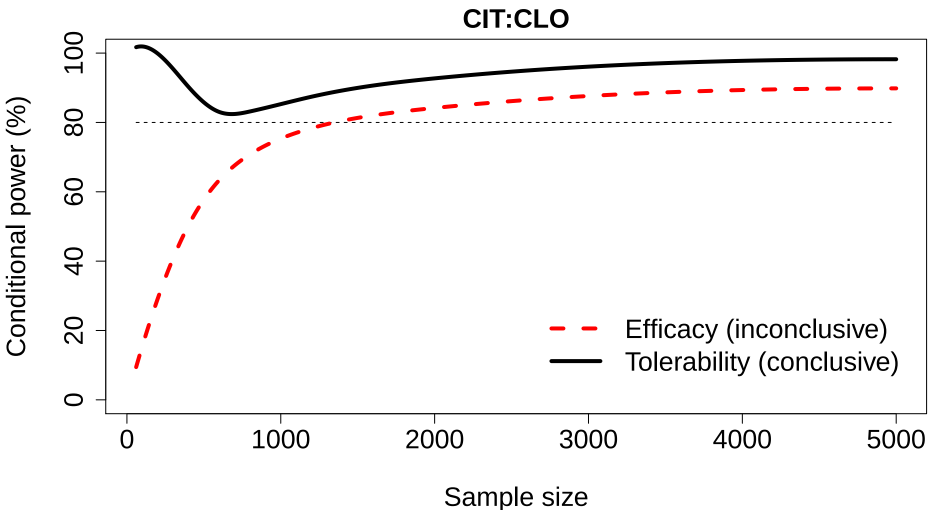

Figure 80:

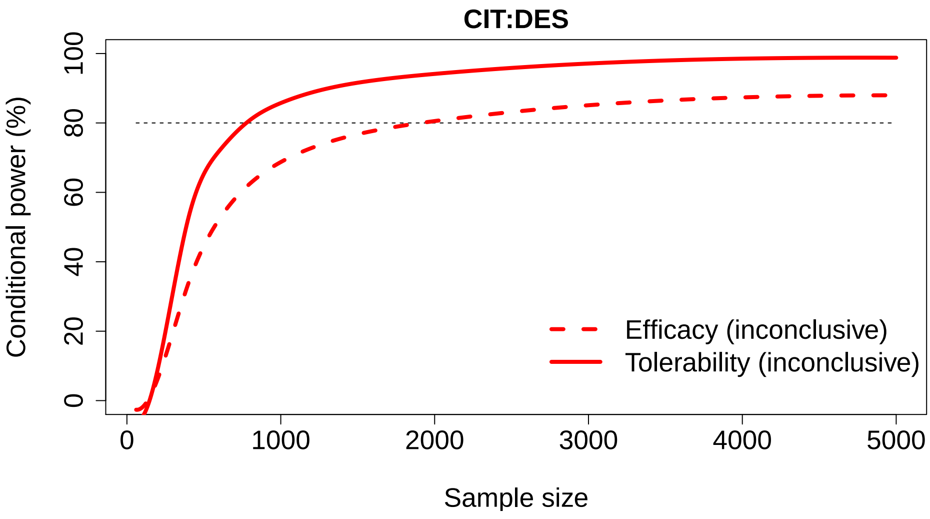

Figure 81:

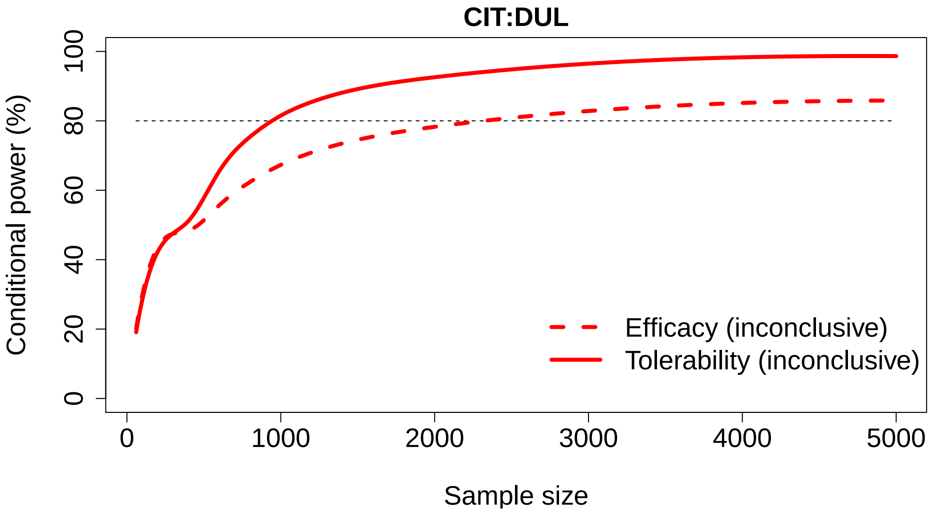

Figure 82:

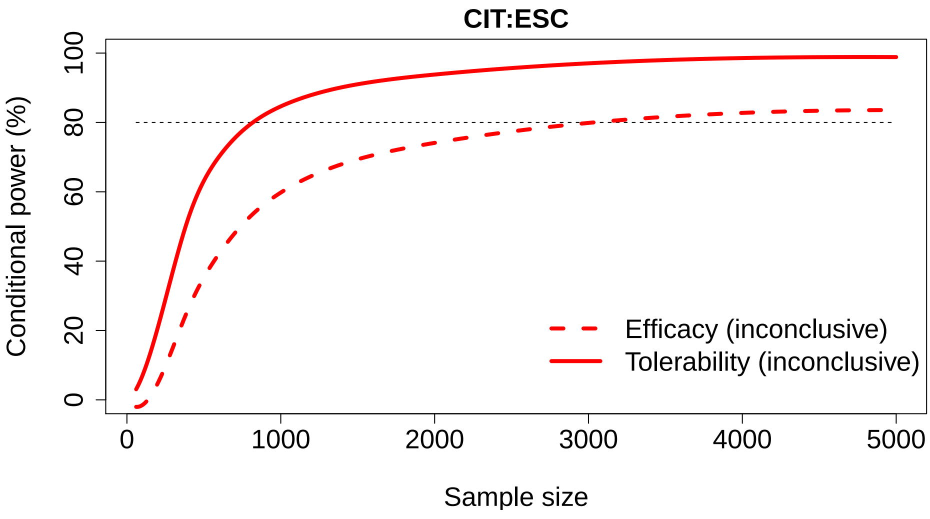

Figure 83:

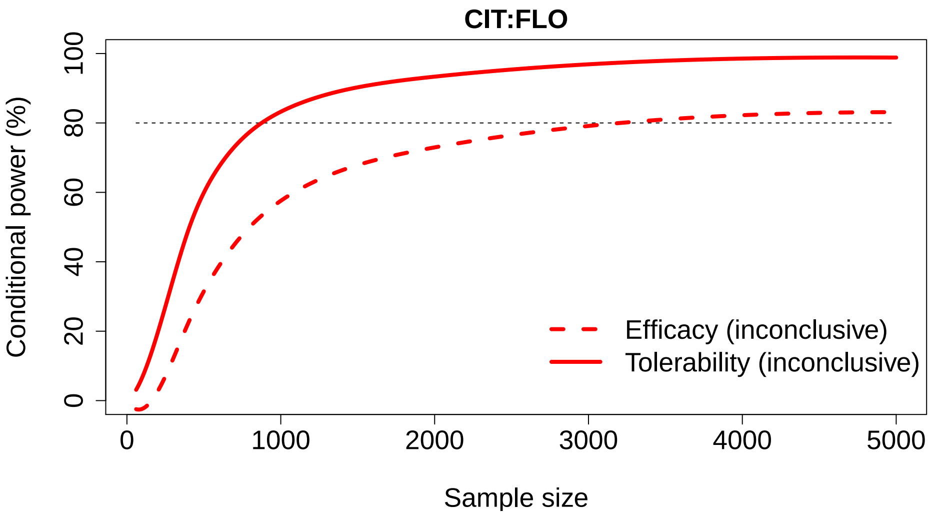

Figure 84:

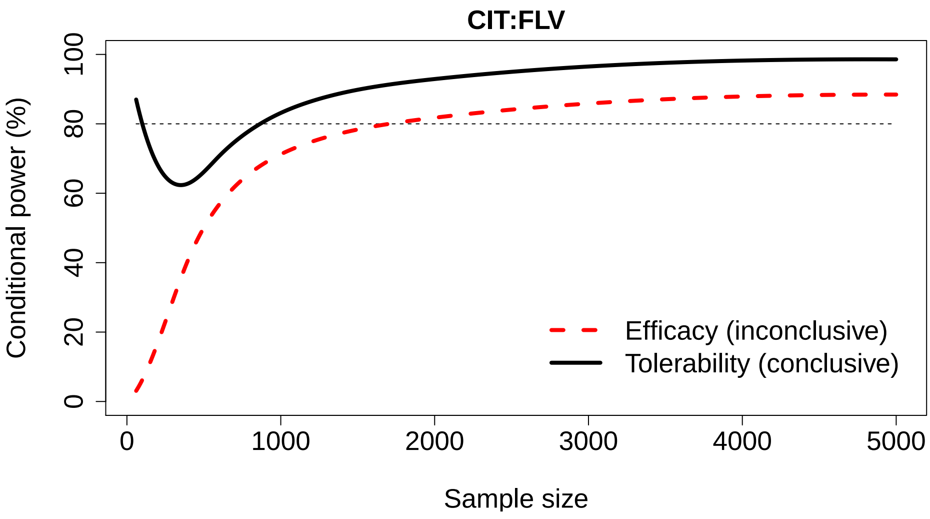

Figure 85:

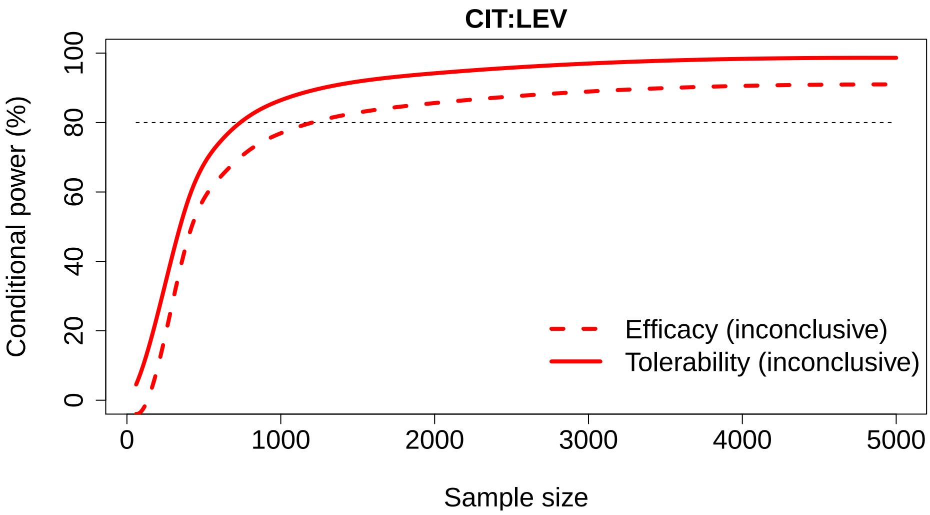

Figure 86:

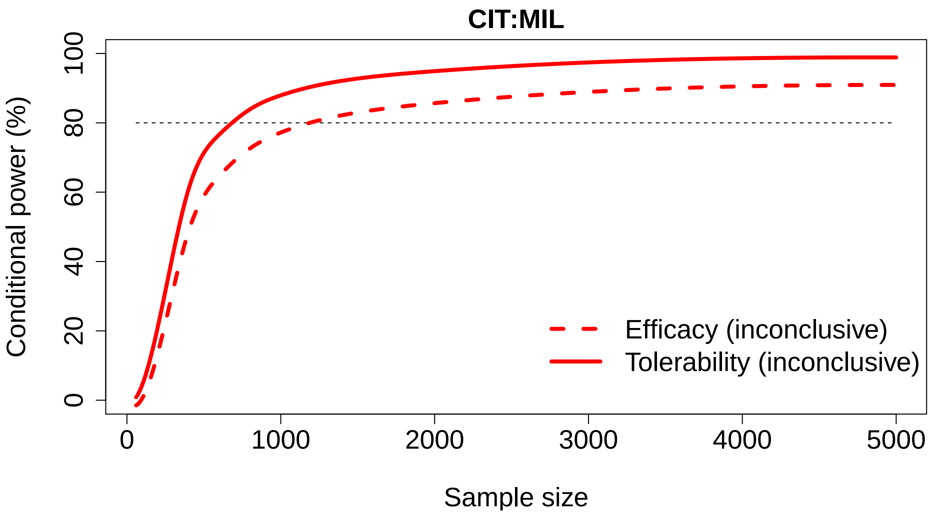

Figure 87:

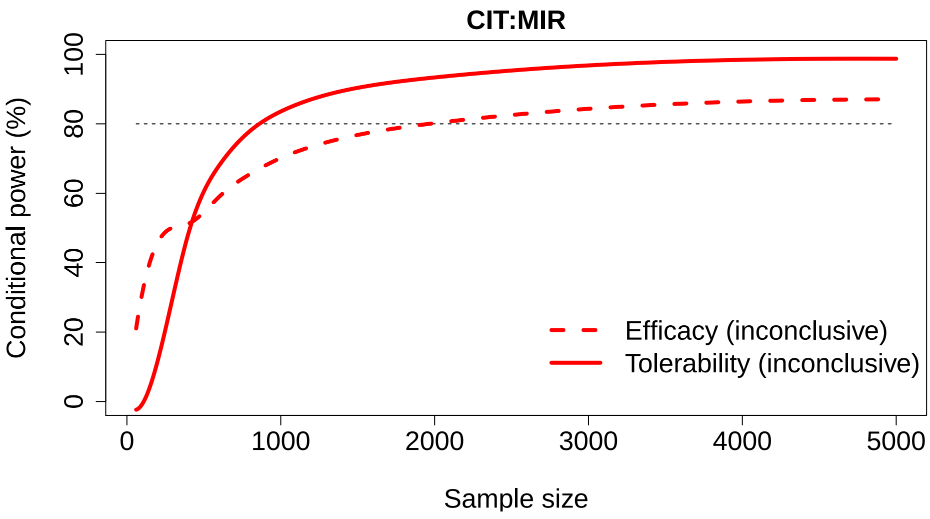

Figure 88:

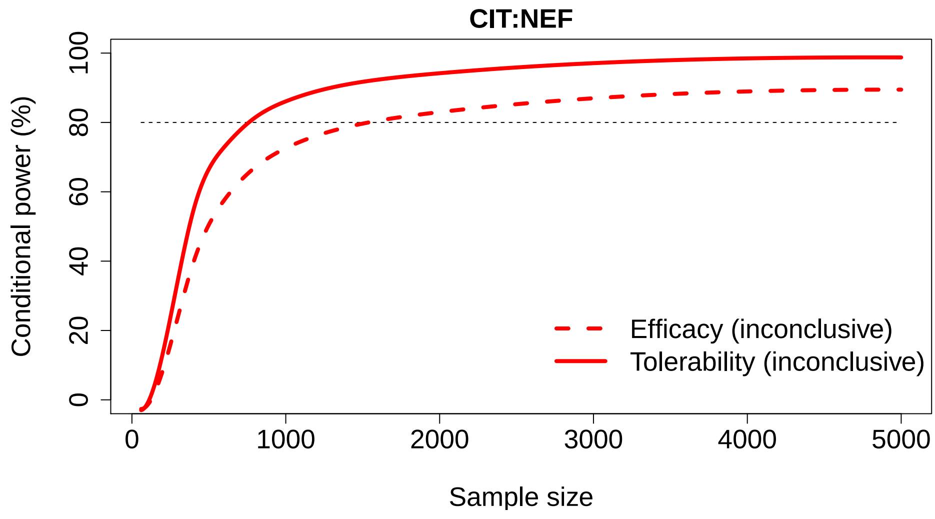

Figure 89:

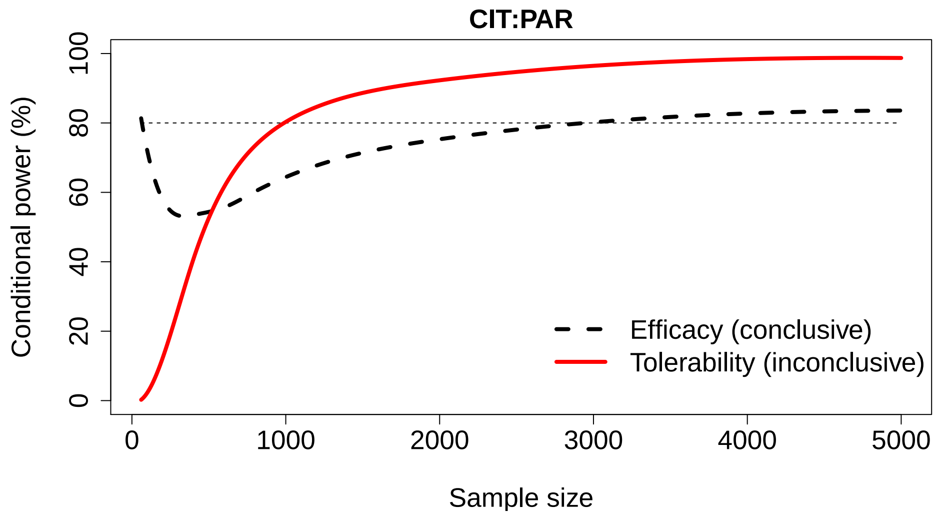

Figure 90:

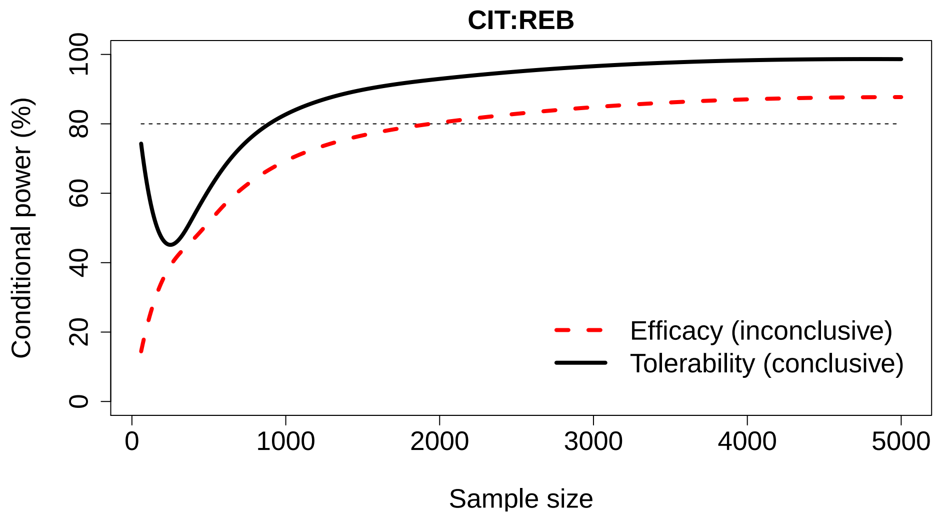

Figure 91:

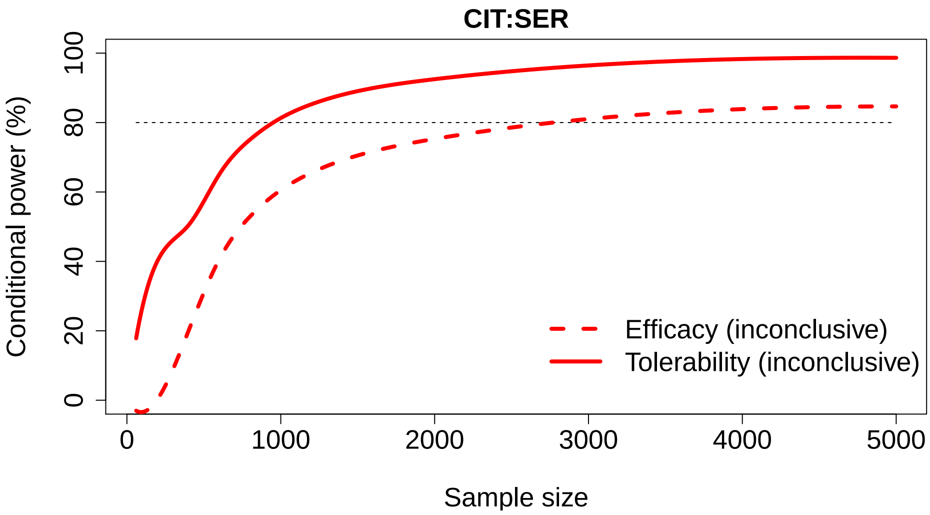

Figure 92:

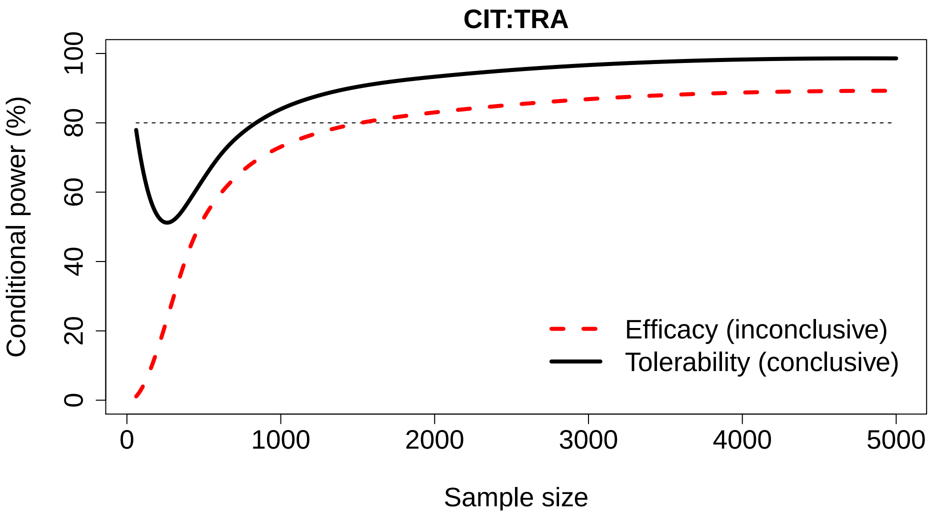

Figure 93:

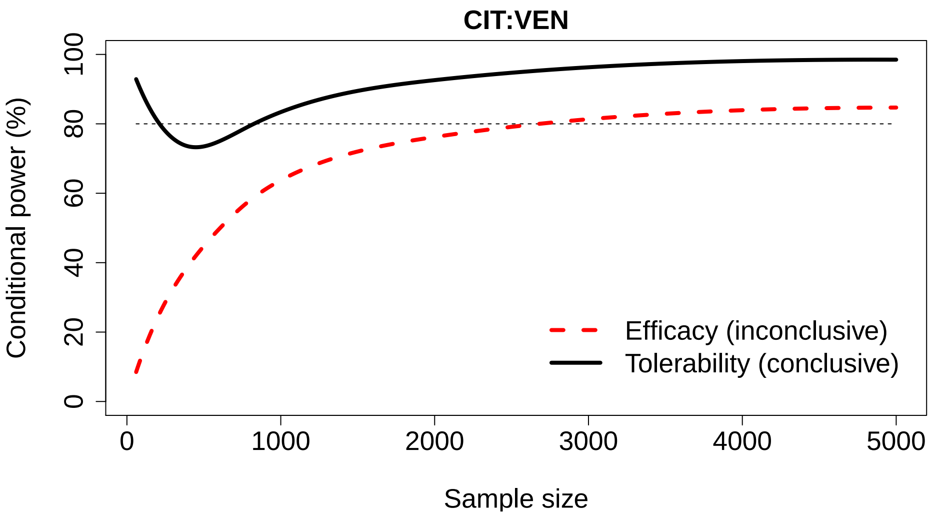

Figure 94:

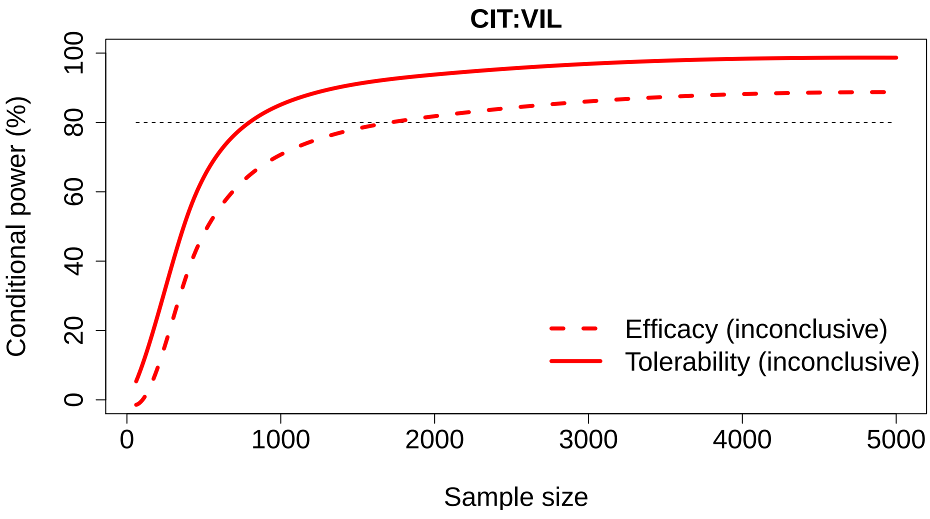

Figure 95:

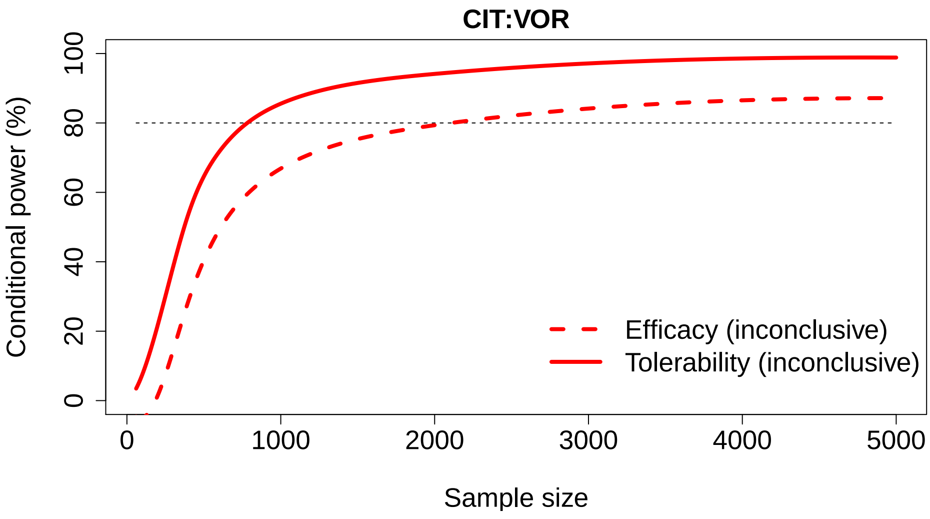

Figure 96:

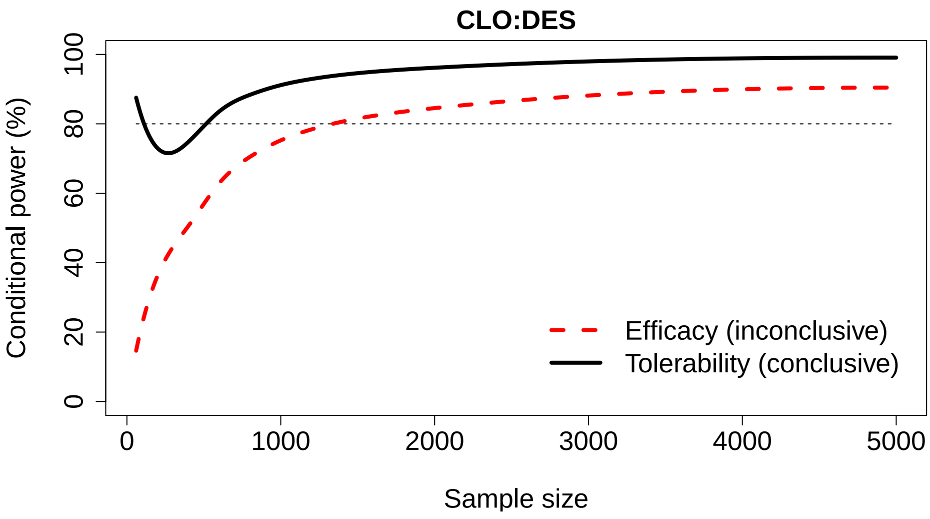

Figure 97:

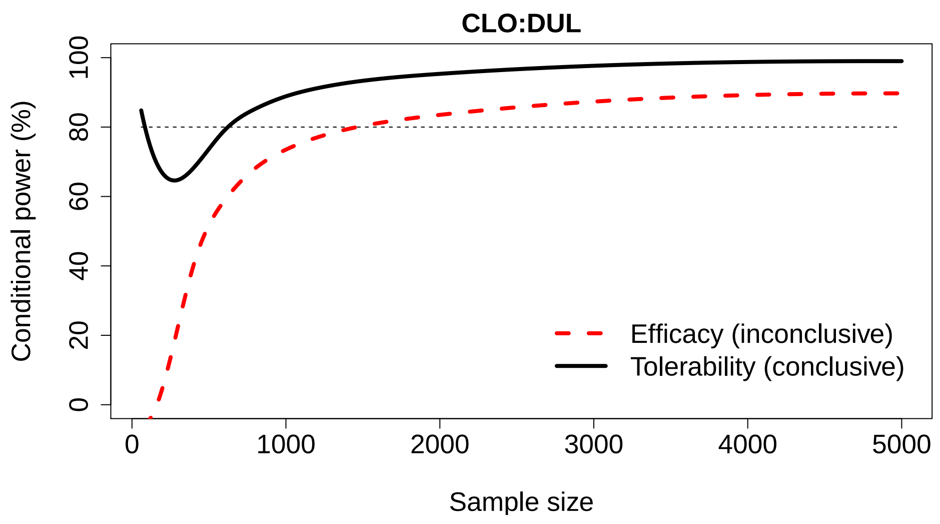

Figure 98:

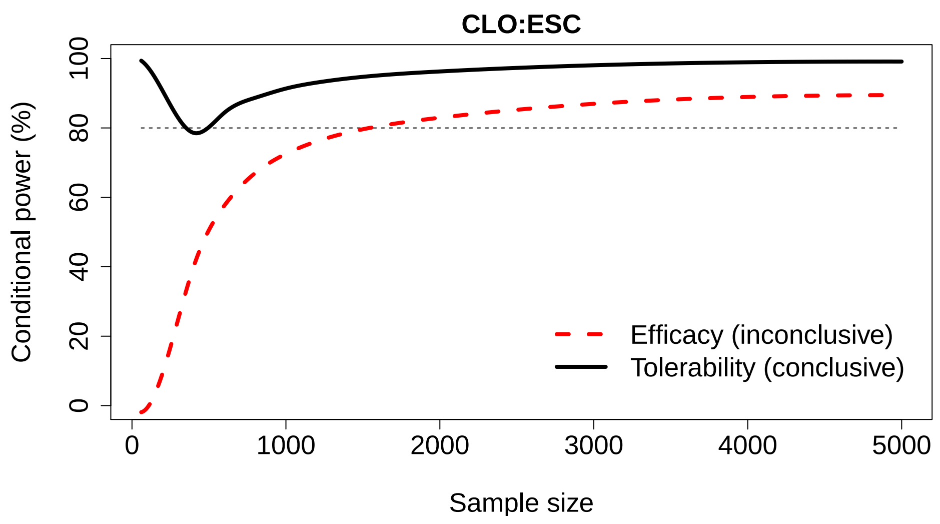

Figure 99:

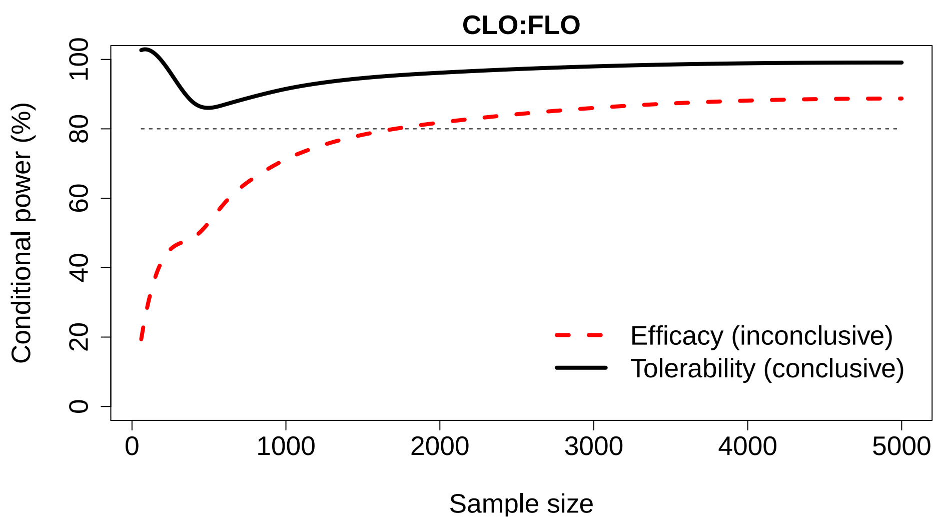

Figure 100:

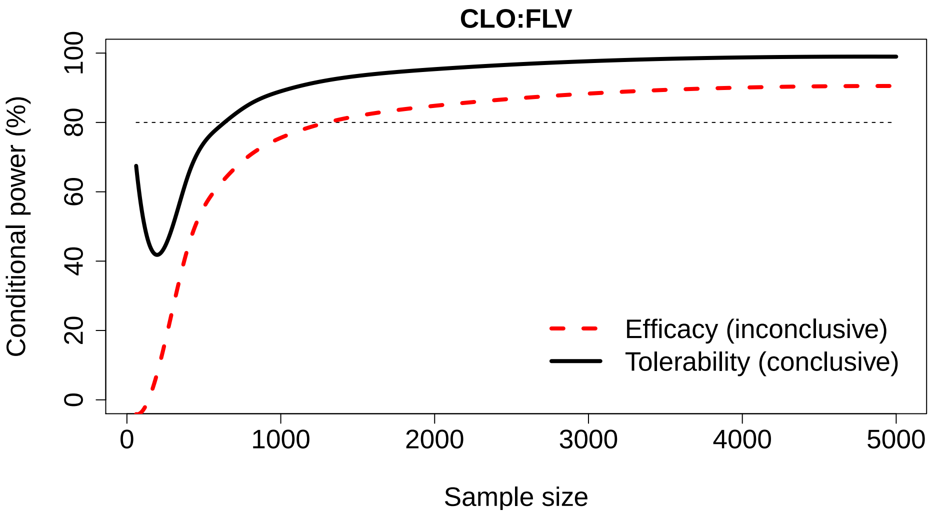

Figure 101:

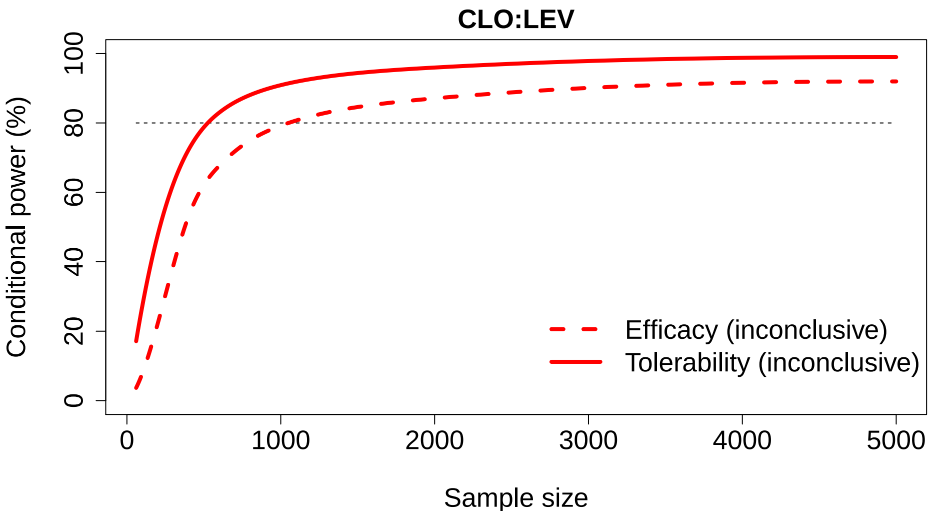

Figure 102:

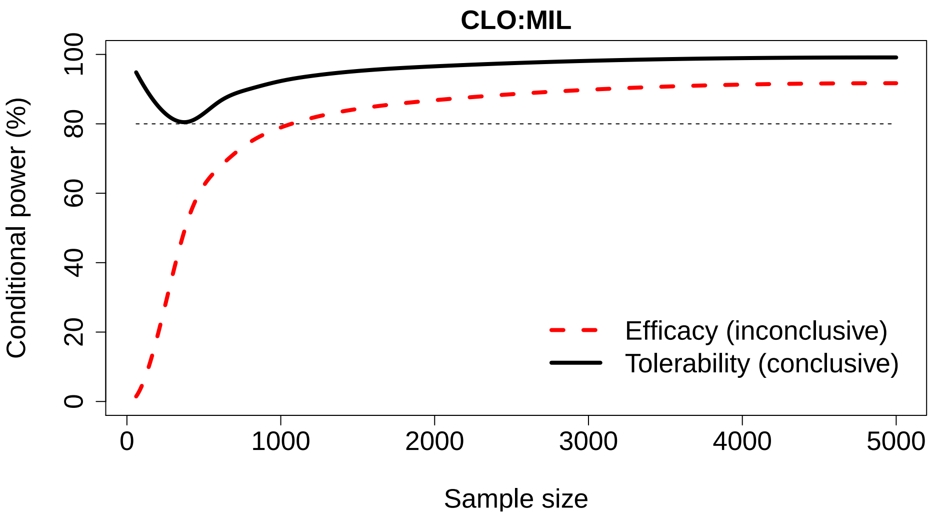

Figure 103:

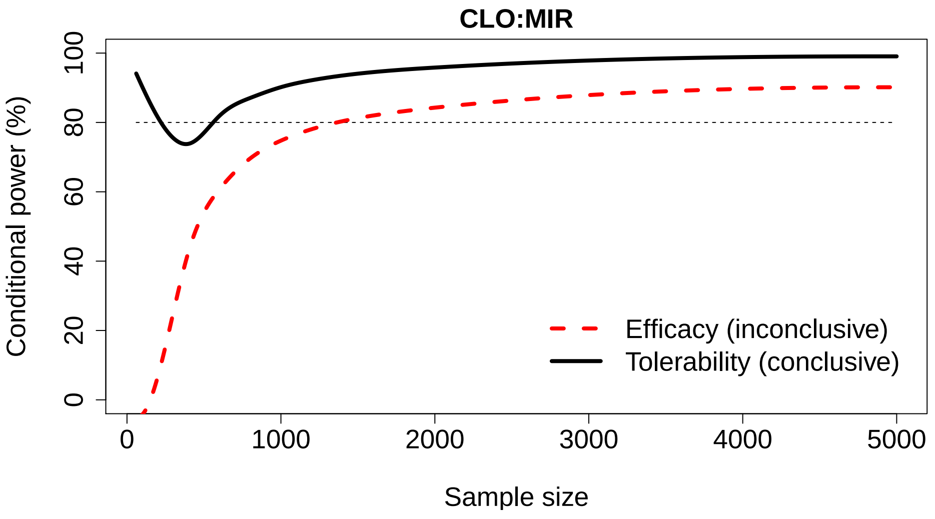

Figure 104:

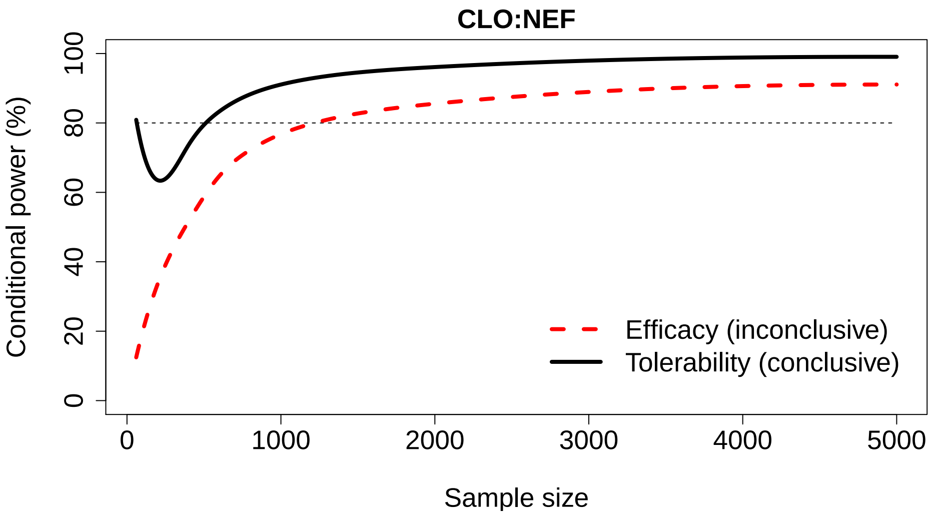

Figure 105:

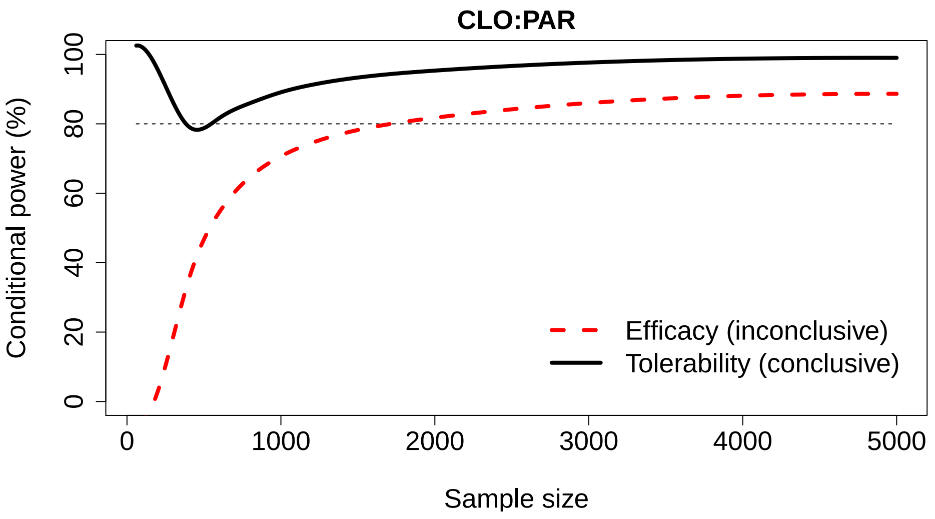

Figure 106:

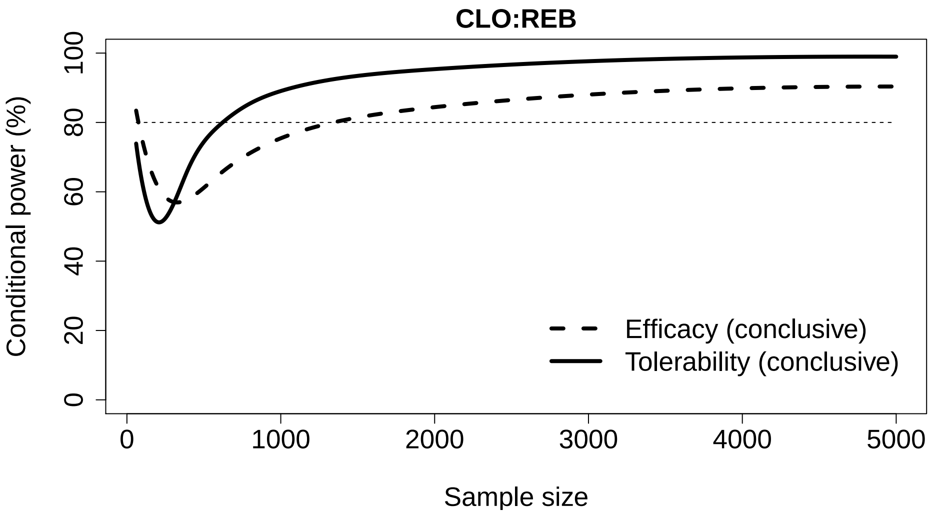

Figure 107:

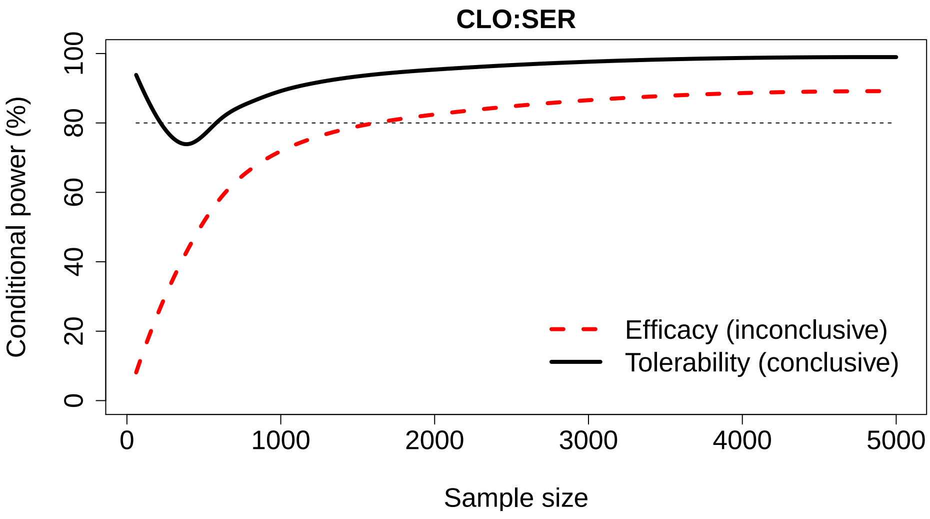

Figure 108:

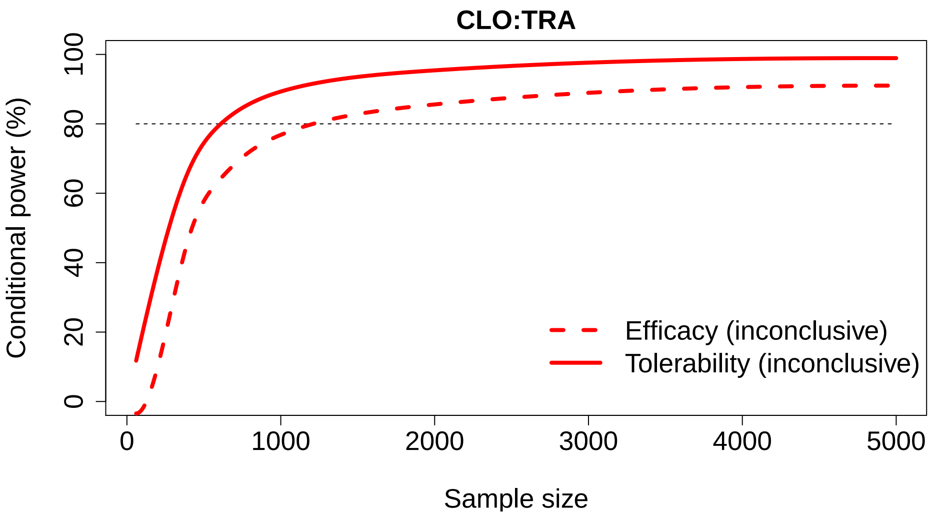

Figure 109:

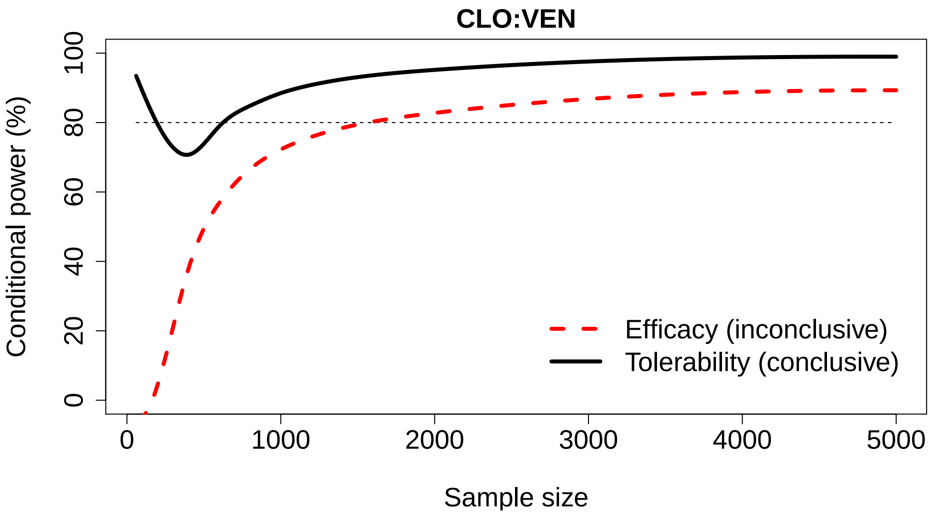

Figure 110:

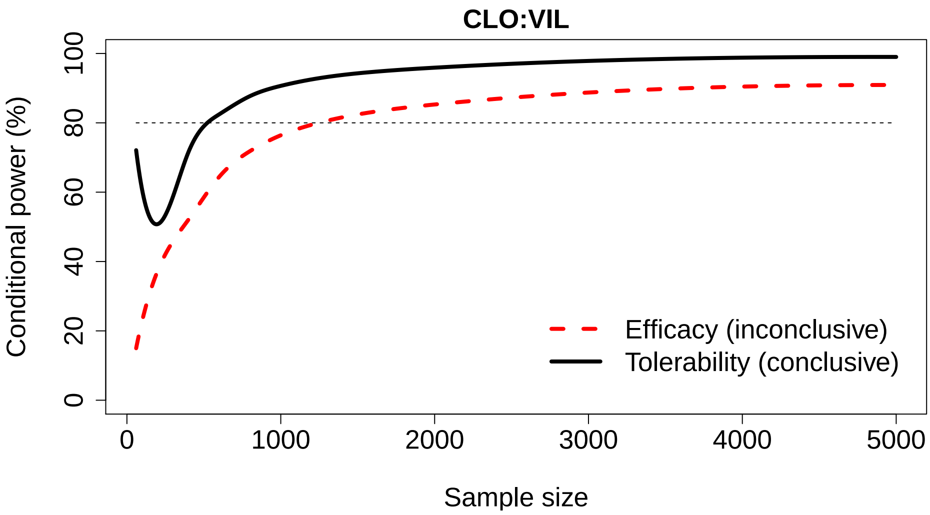

Figure 111:

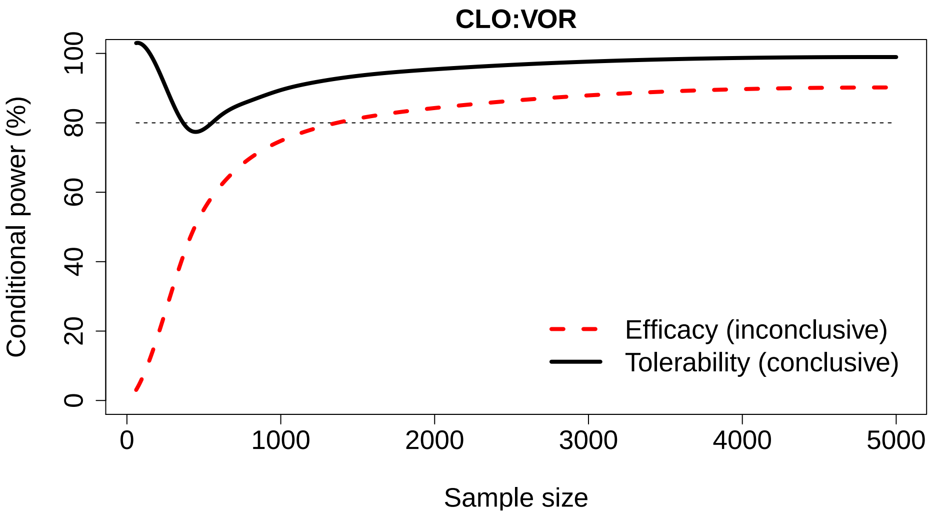

Figure 112:

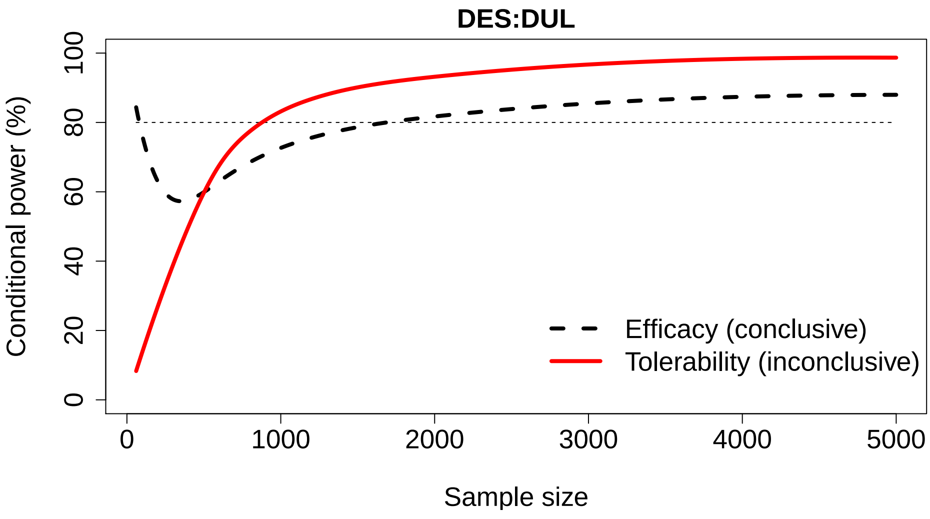

Figure 113:

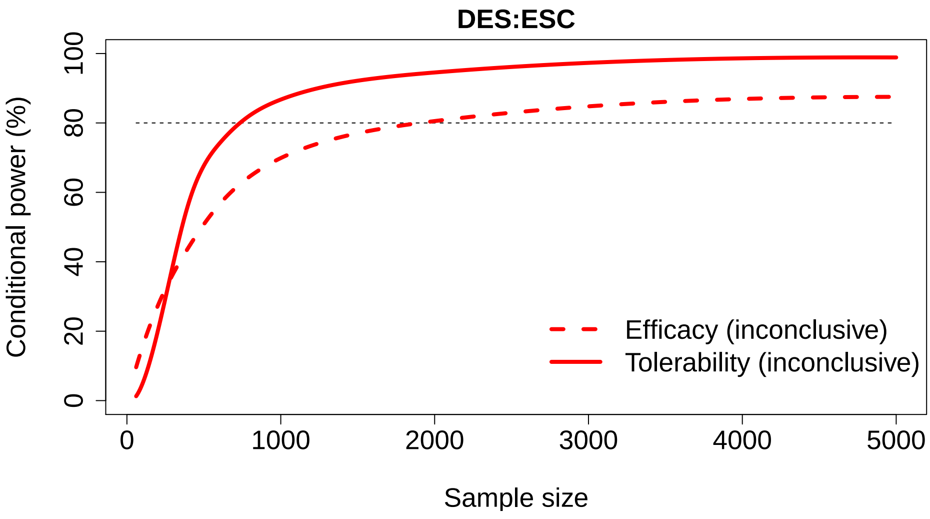

Figure 114:

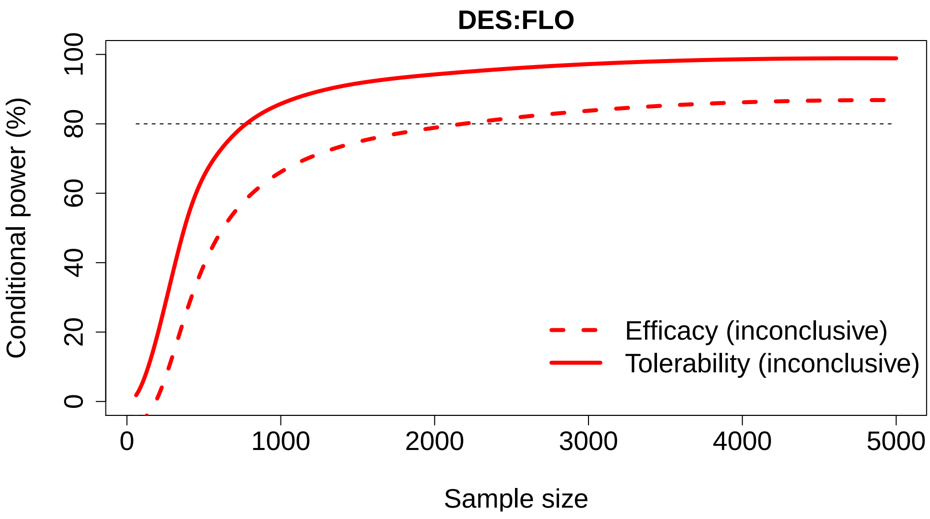

Figure 115:

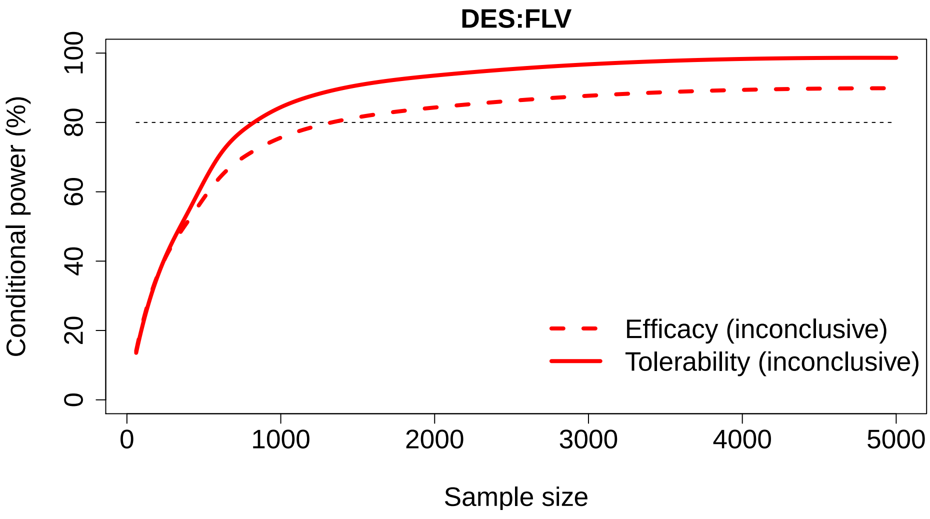

Figure 116:

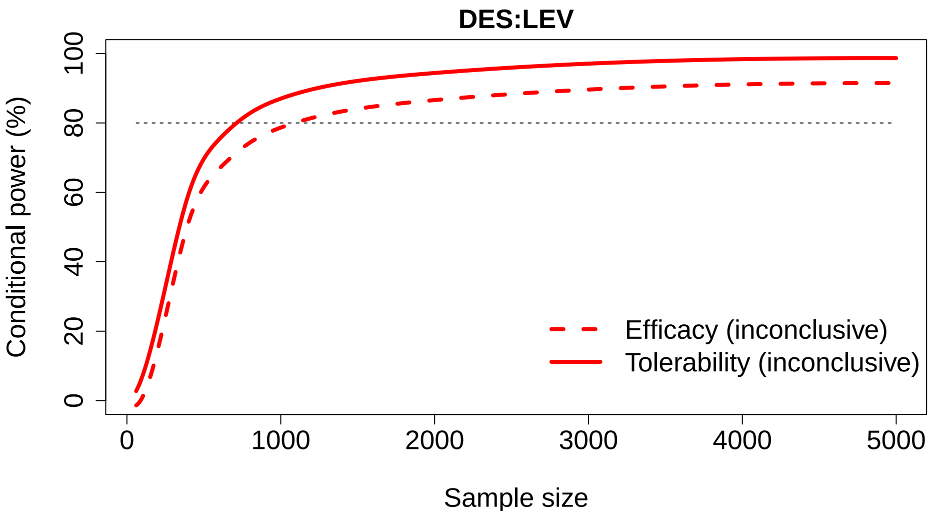

Figure 117:

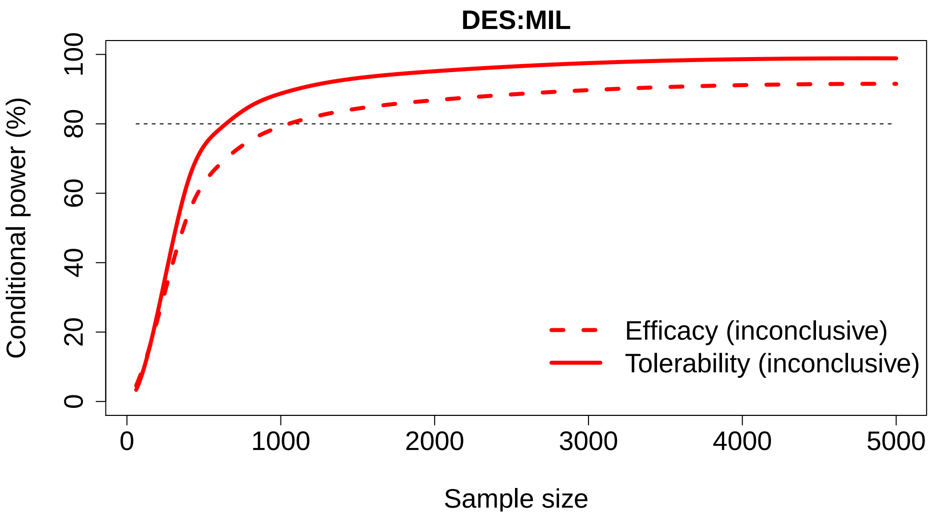

Figure 118:

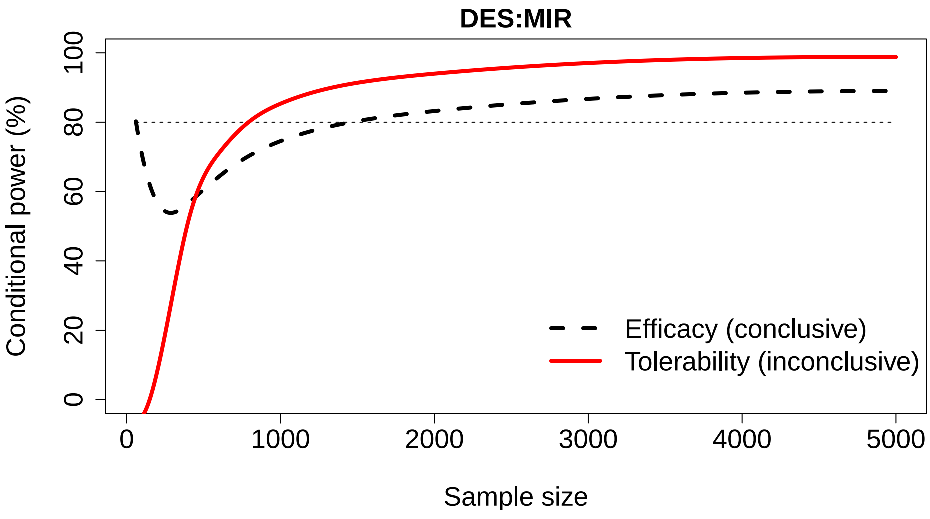

Figure 119:

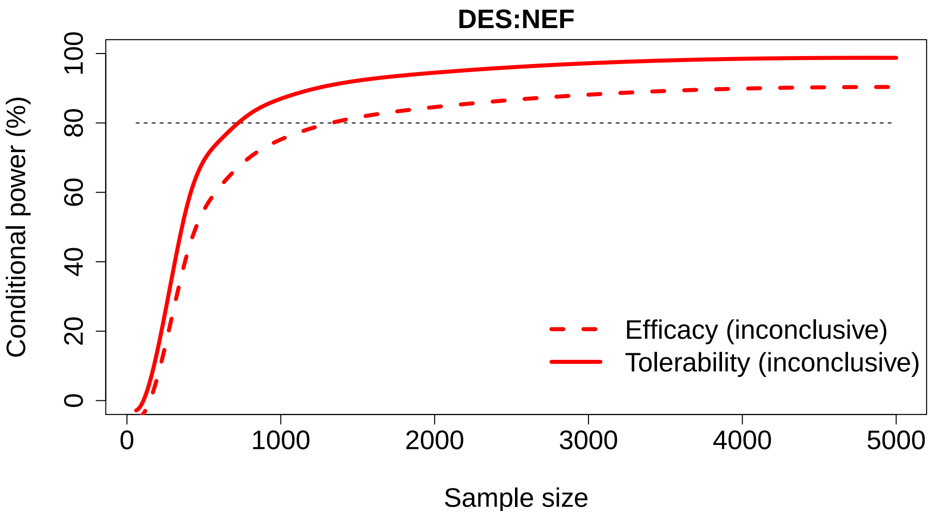

Figure 120:

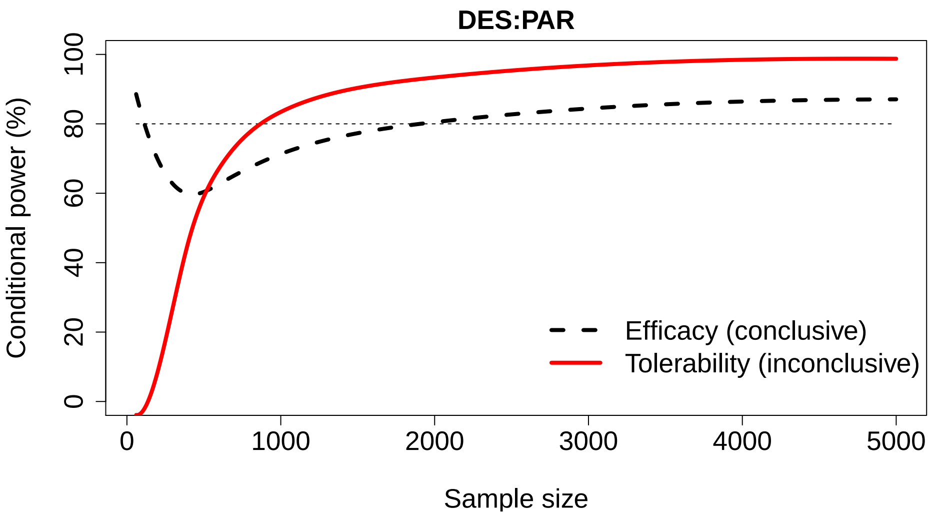

Figure 121:

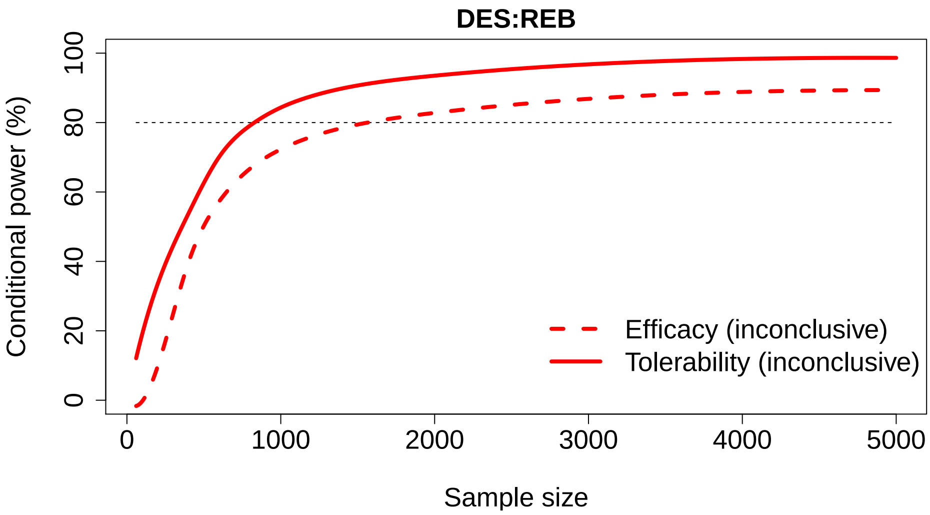

Figure 122:

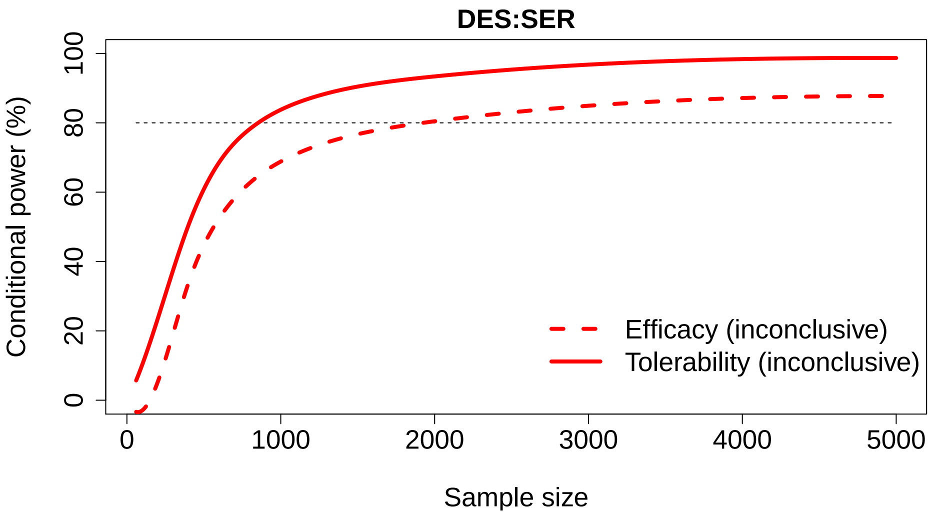

Figure 123:

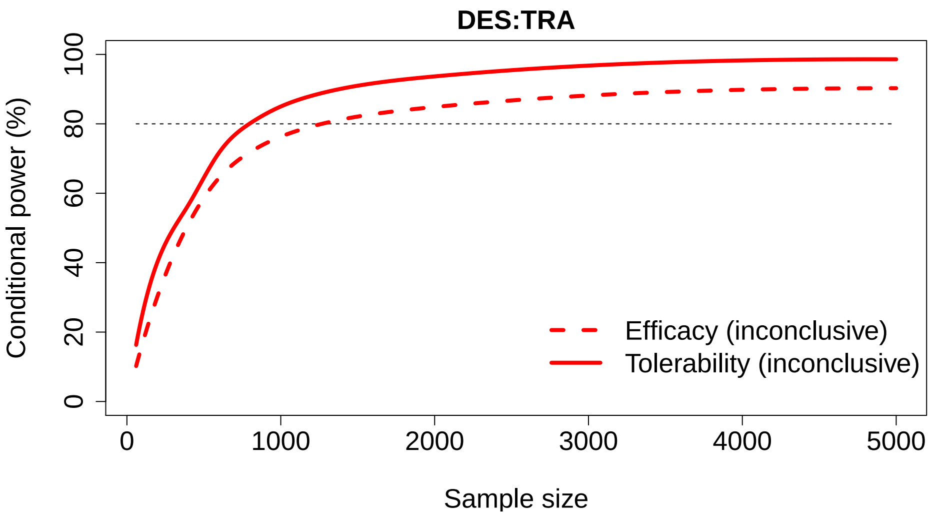

Figure 124:

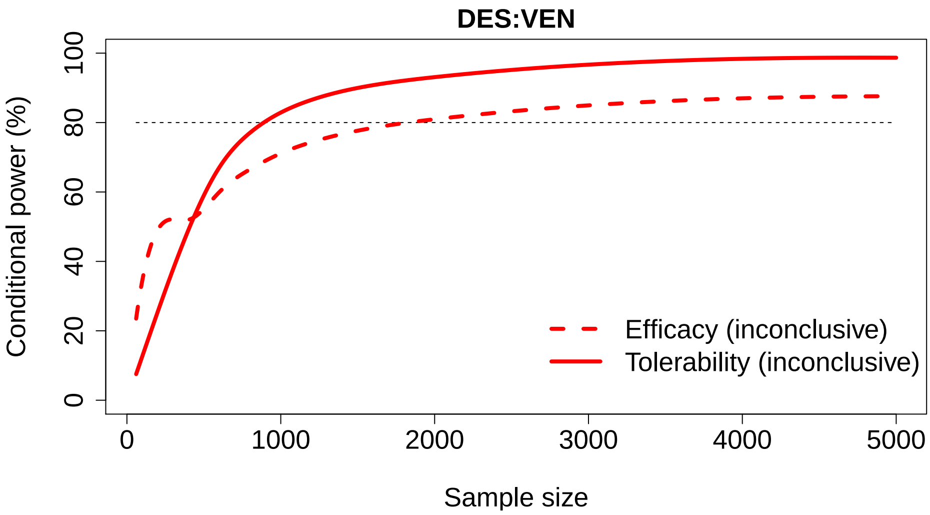

Figure 125:

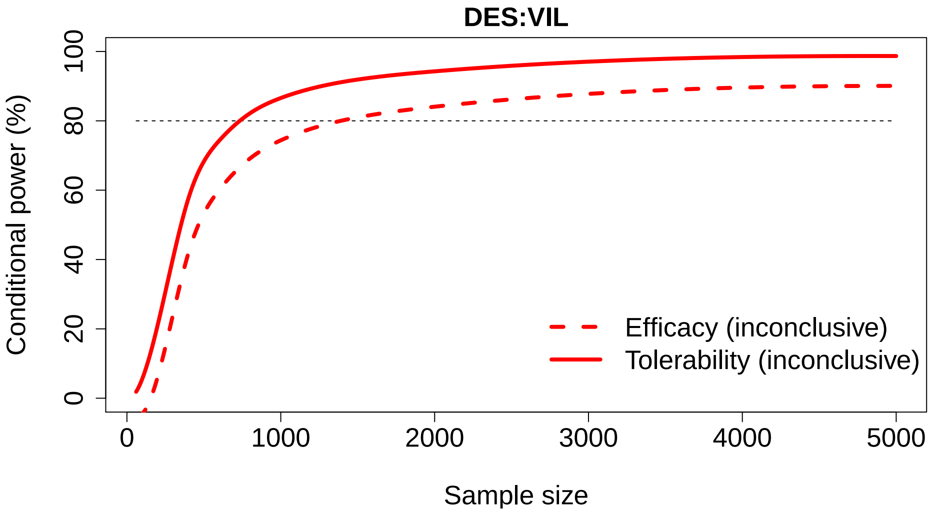

Figure 126:

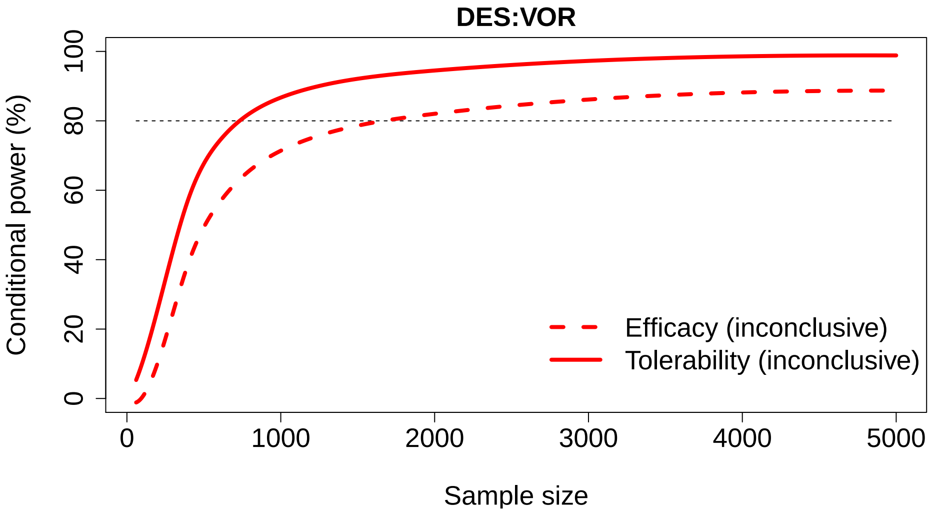

Figure 127:

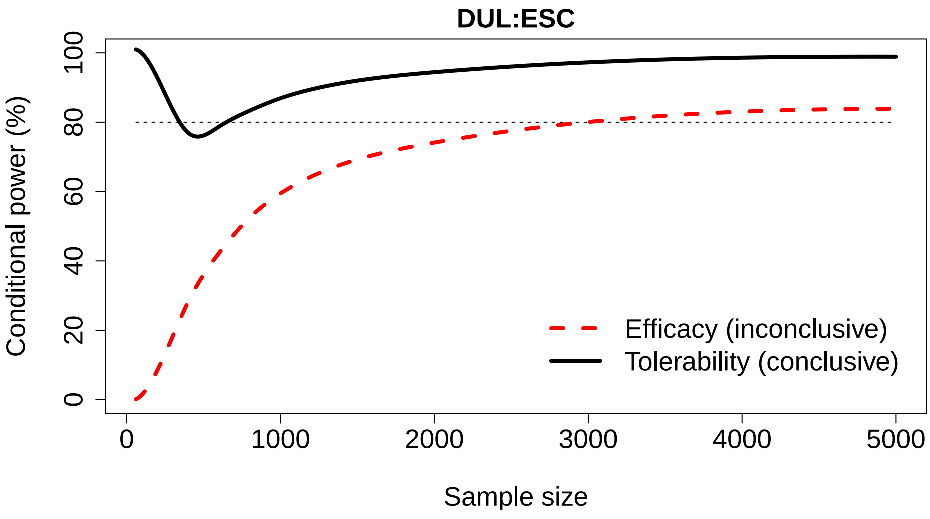

Figure 128:

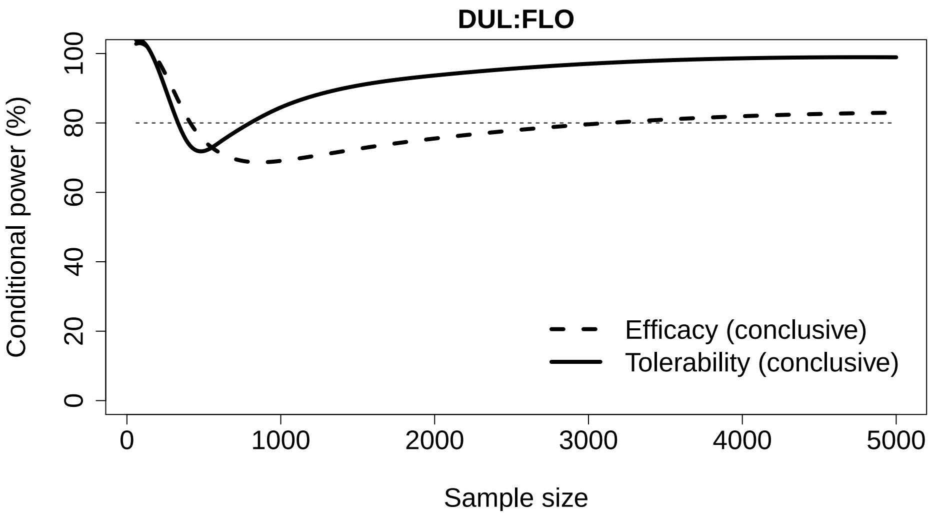

Figure 129:

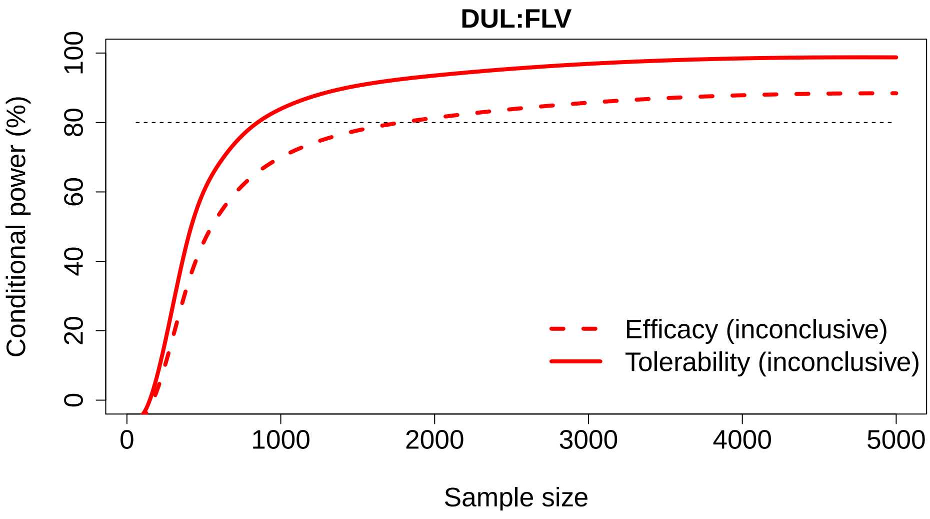

Figure 130:

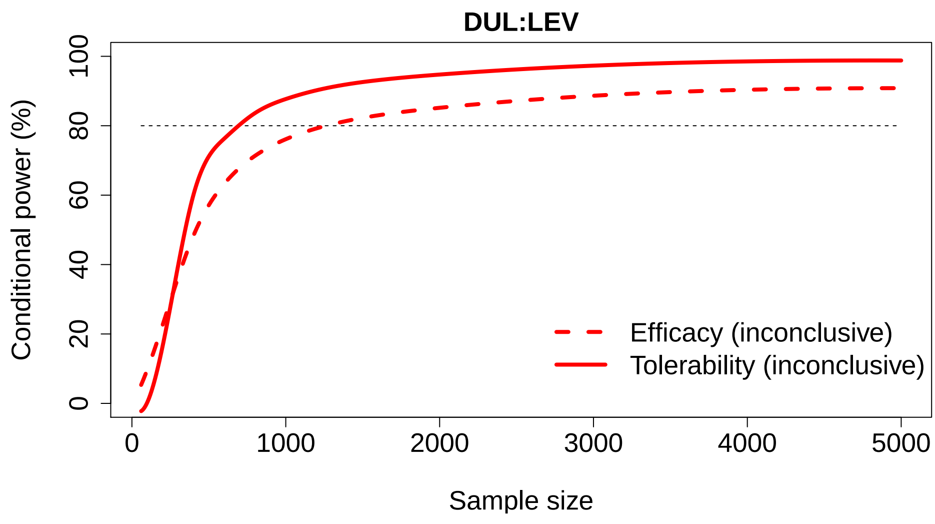

Figure 131:

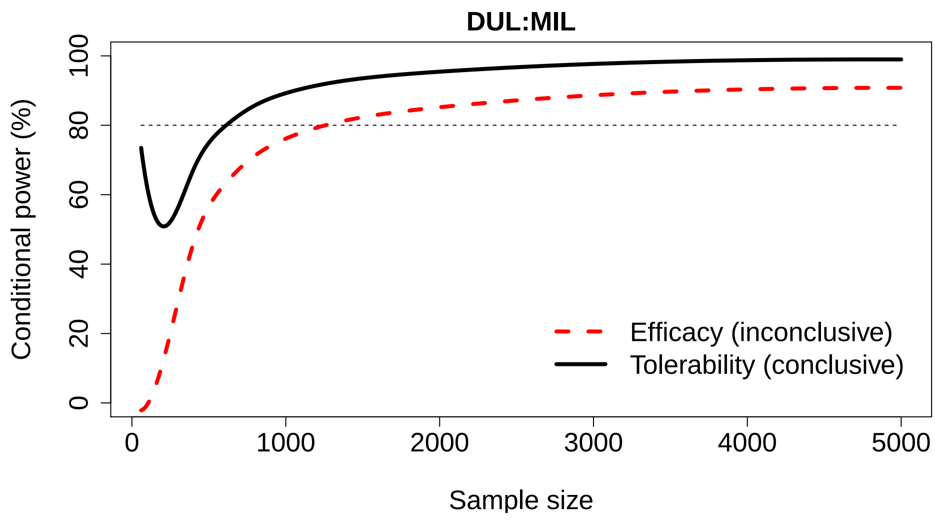

Figure 132:

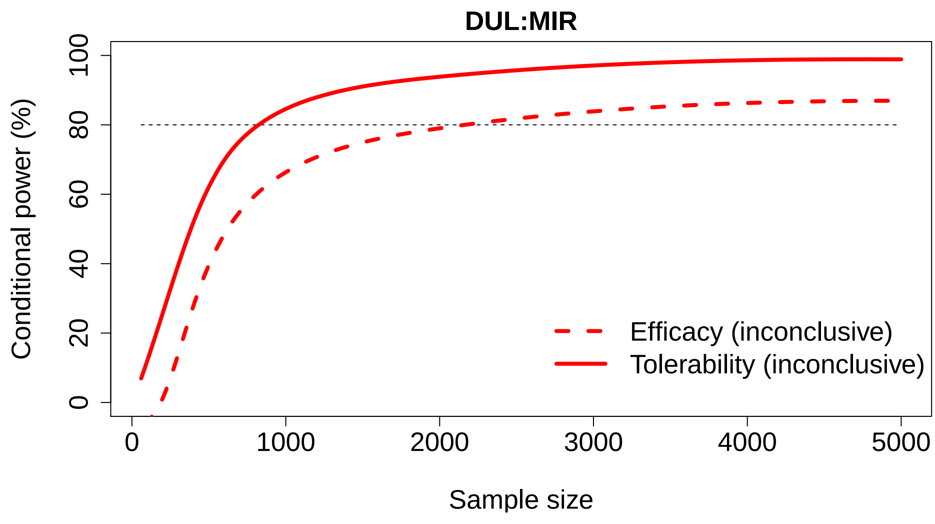

Figure 133:

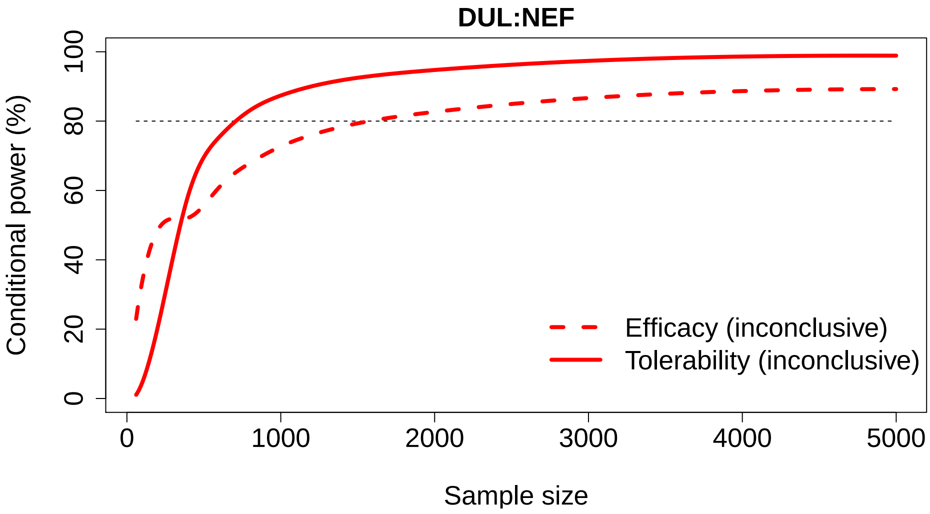

Figure 134:

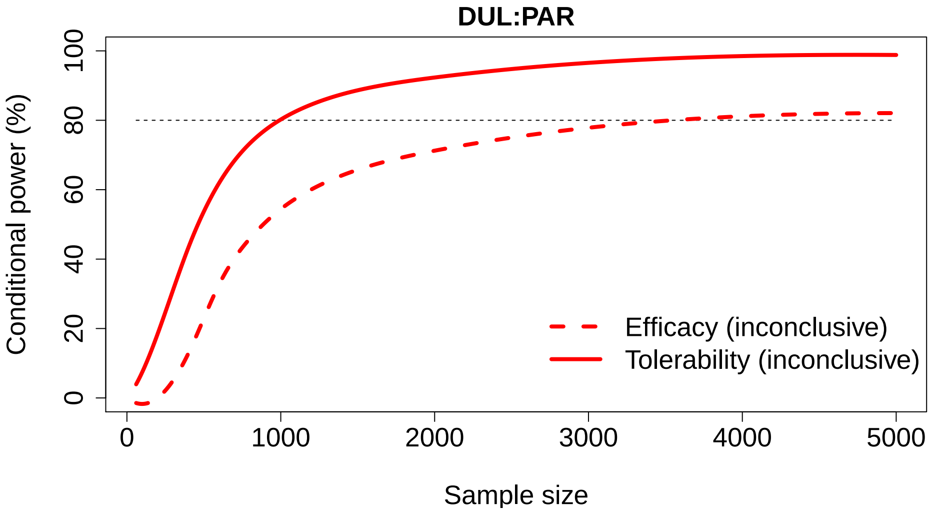

Figure 135:

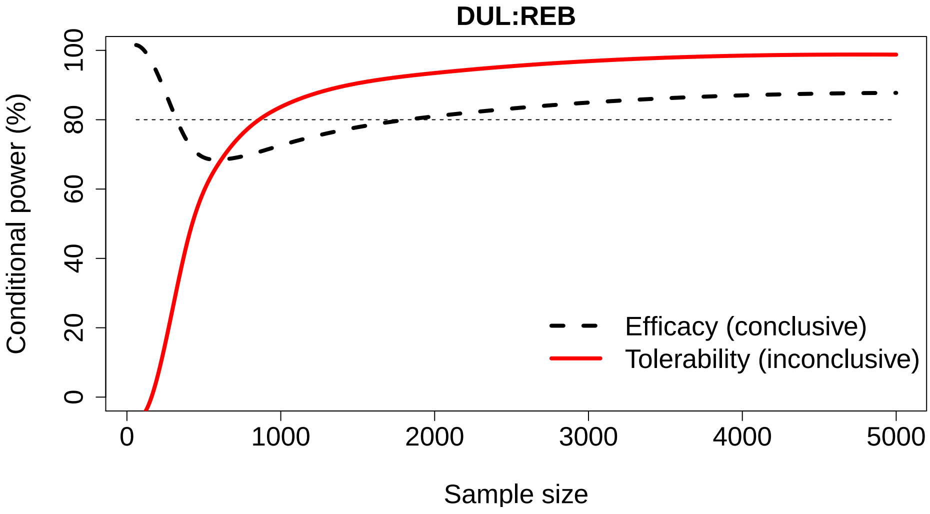

Figure 136:

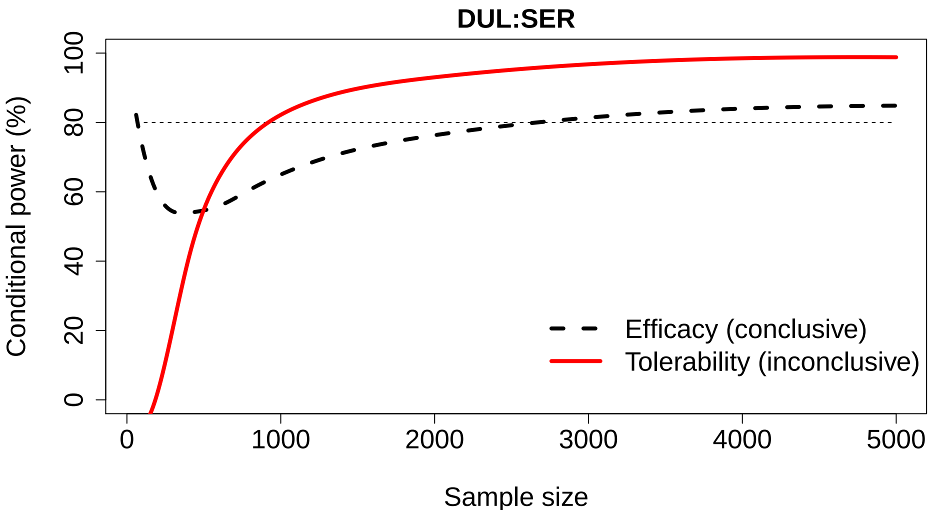

Figure 137:

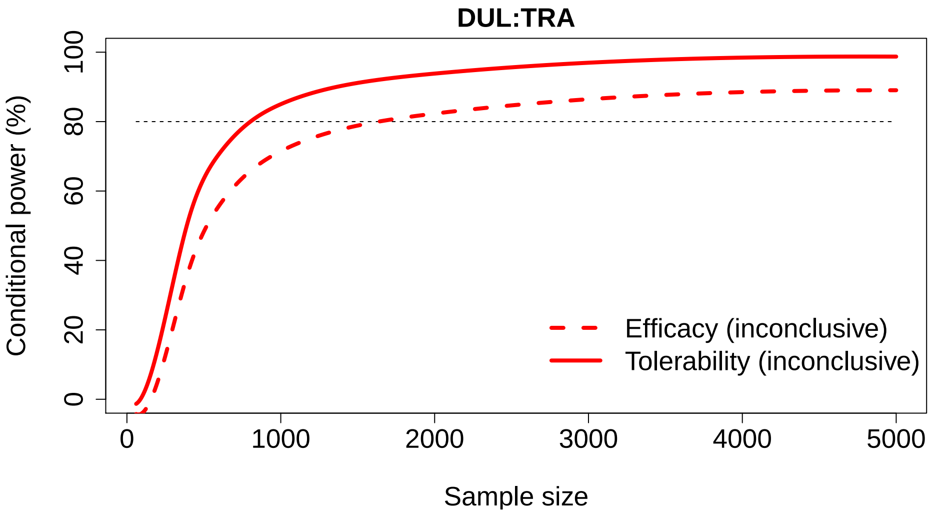

Figure 138:

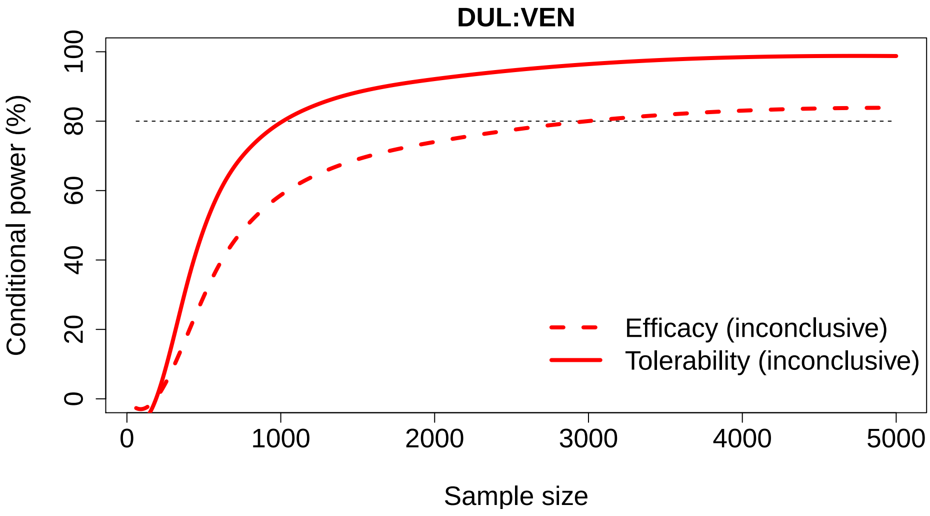

Figure 139:

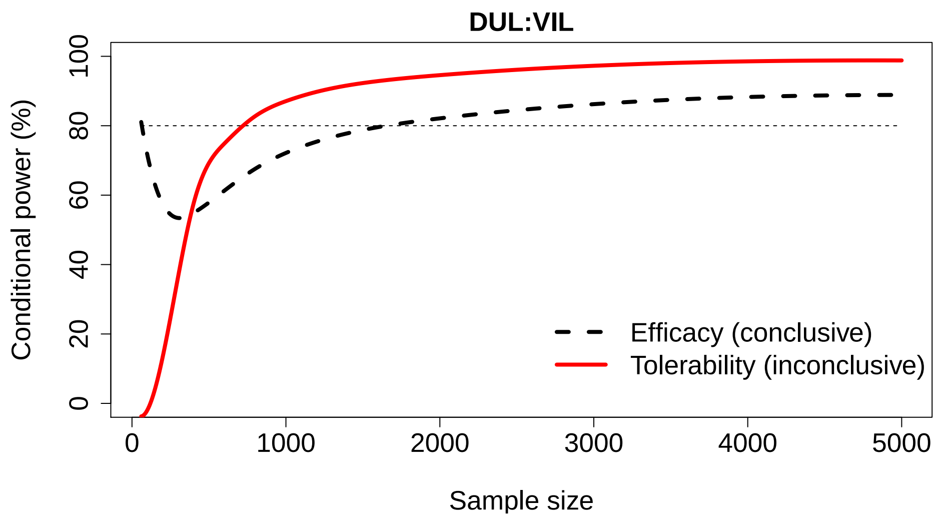

Figure 140:

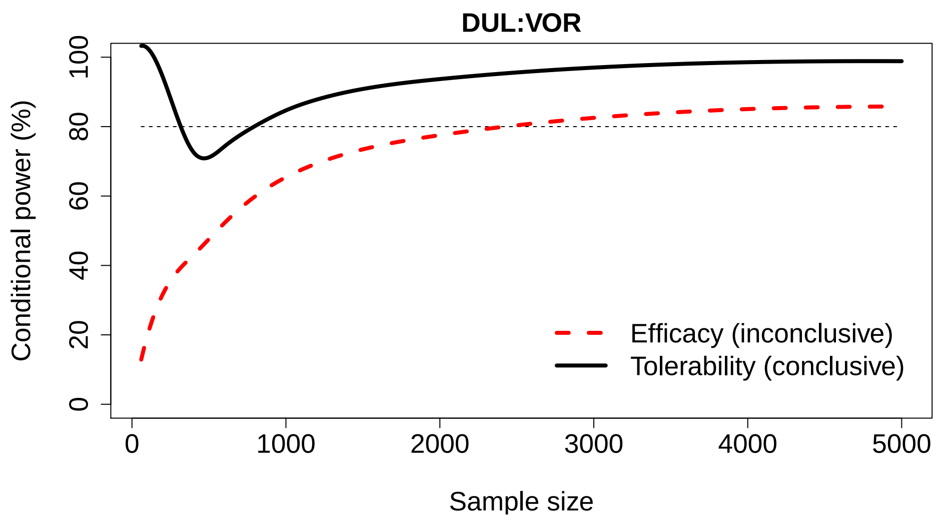

Figure 141:

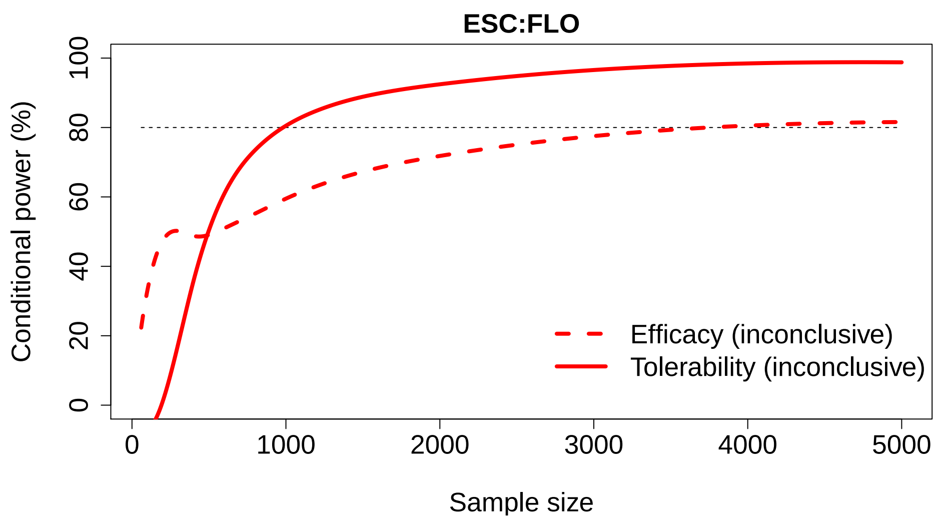

Figure 142:

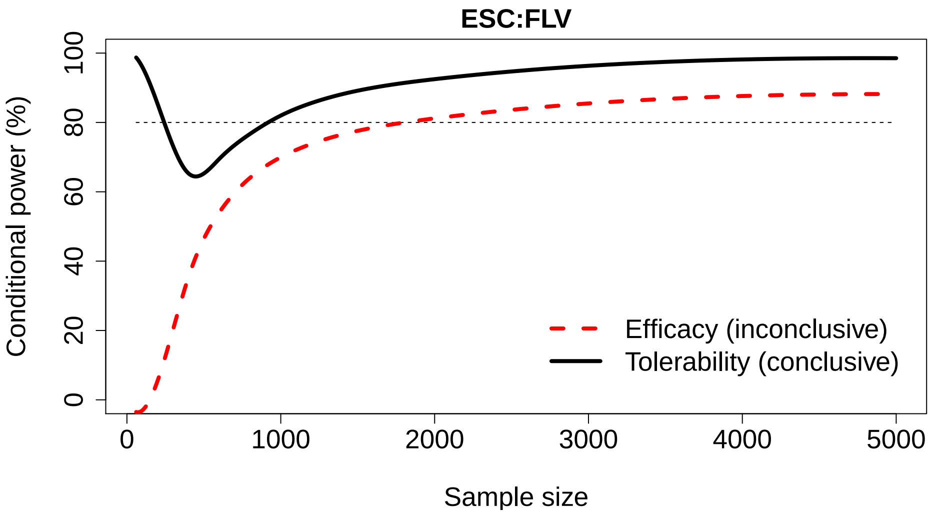

Figure 143:

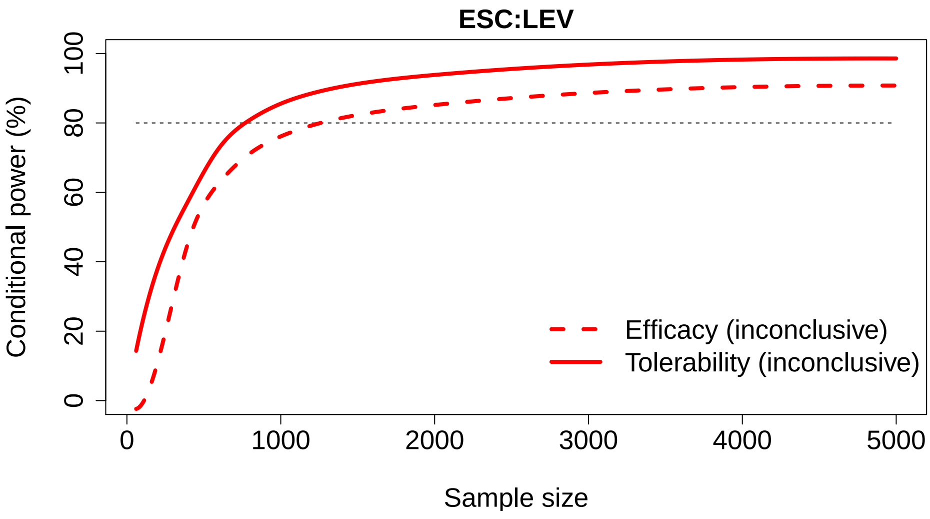

Figure 144:

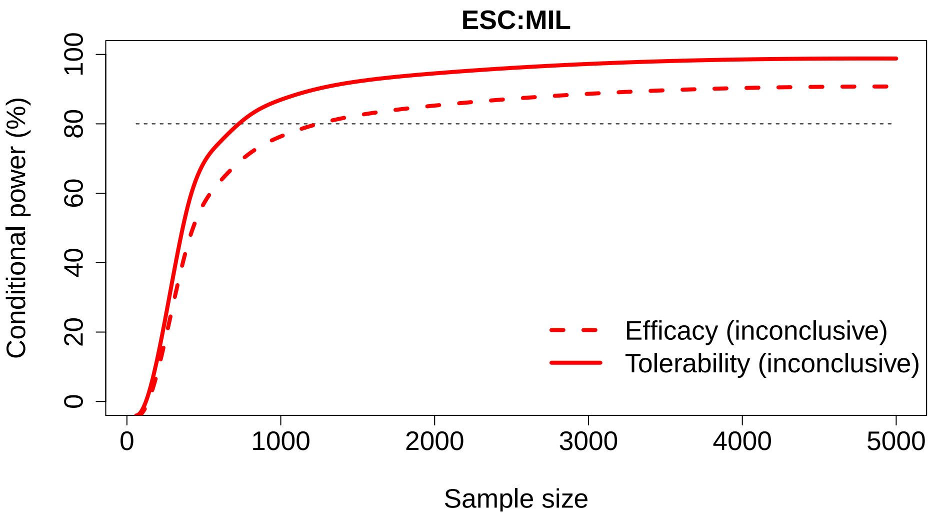

Figure 145:

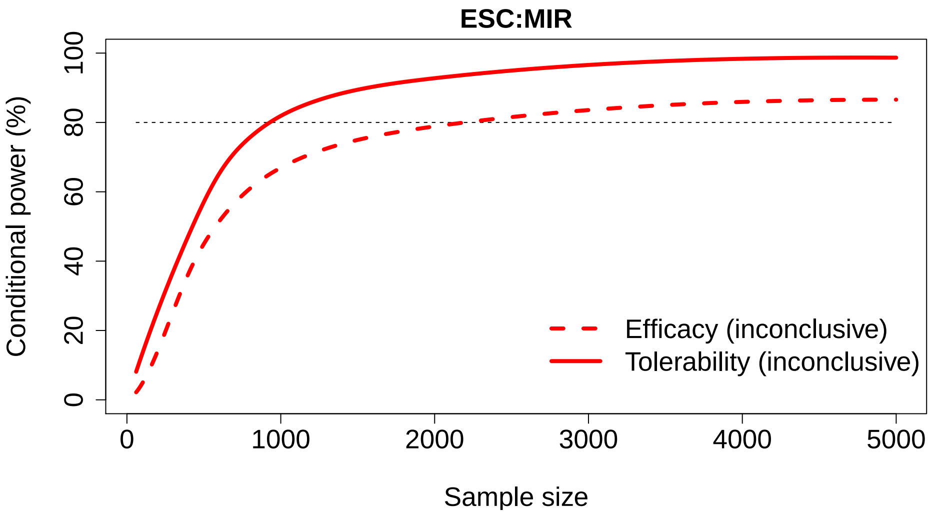

Figure 146:

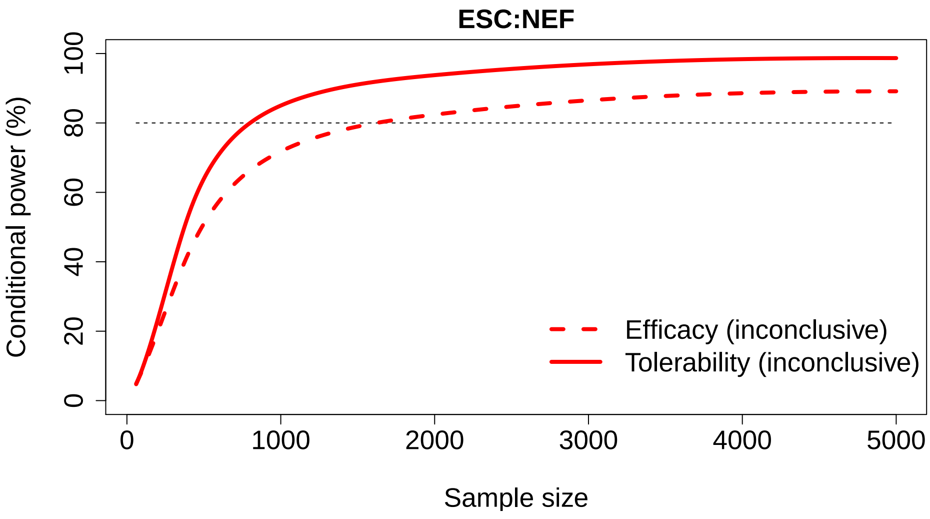

Figure 147:

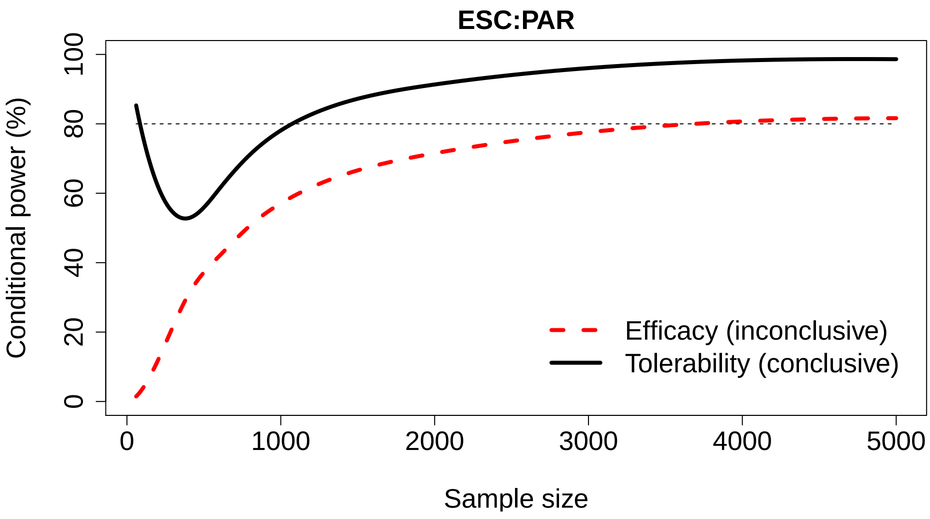

Figure 148:

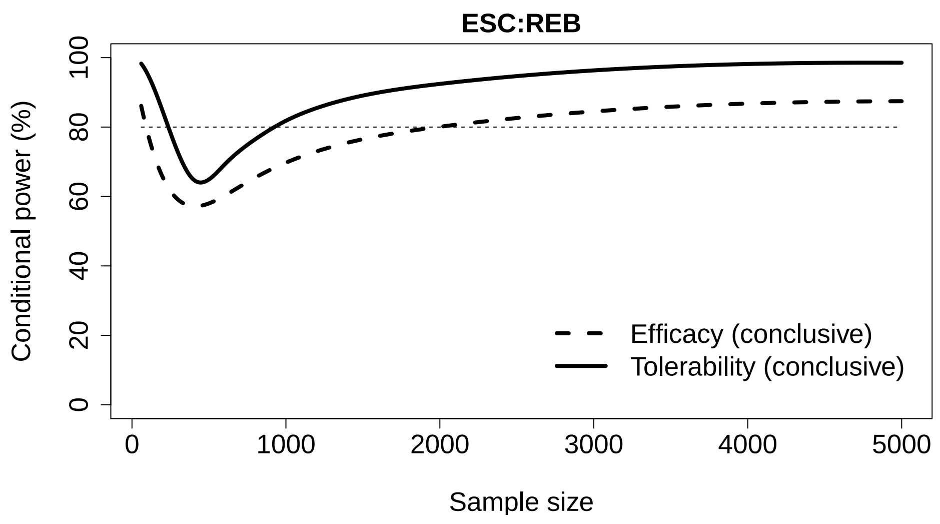

Figure 149:

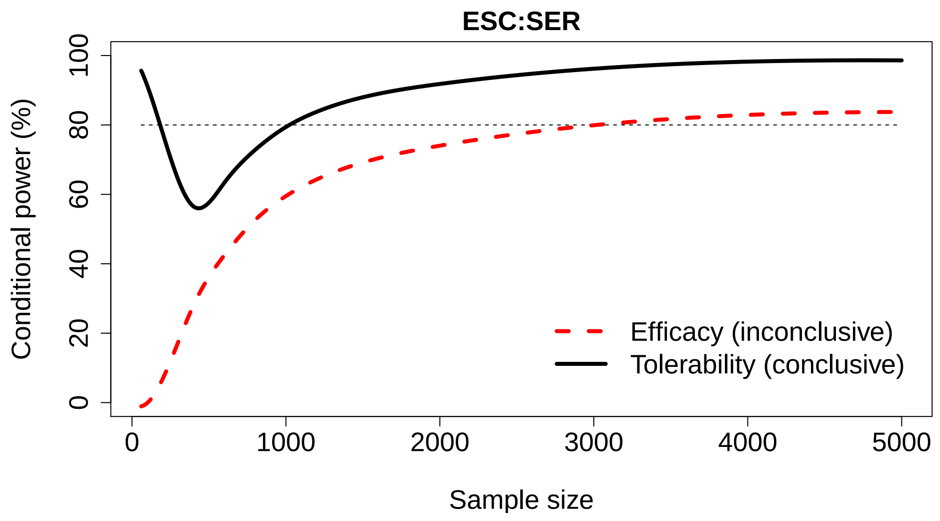

Figure 150:

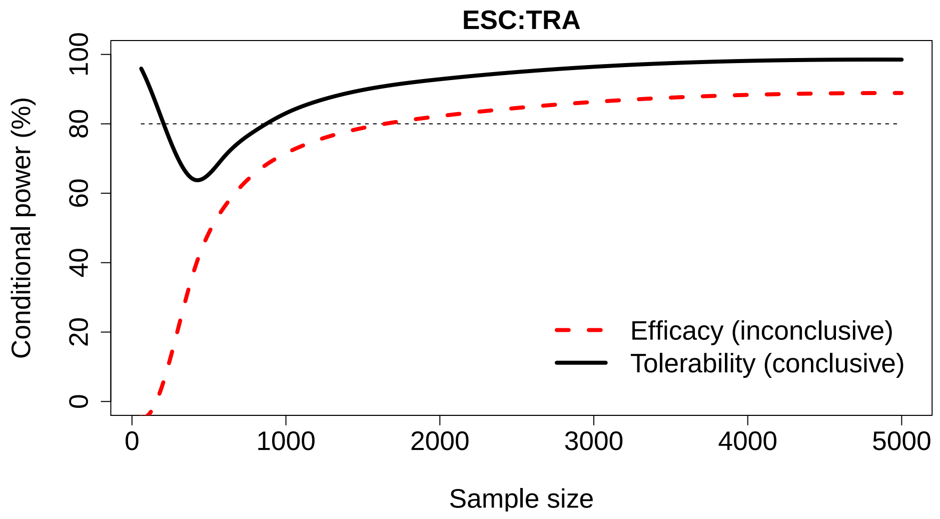

Figure 151:

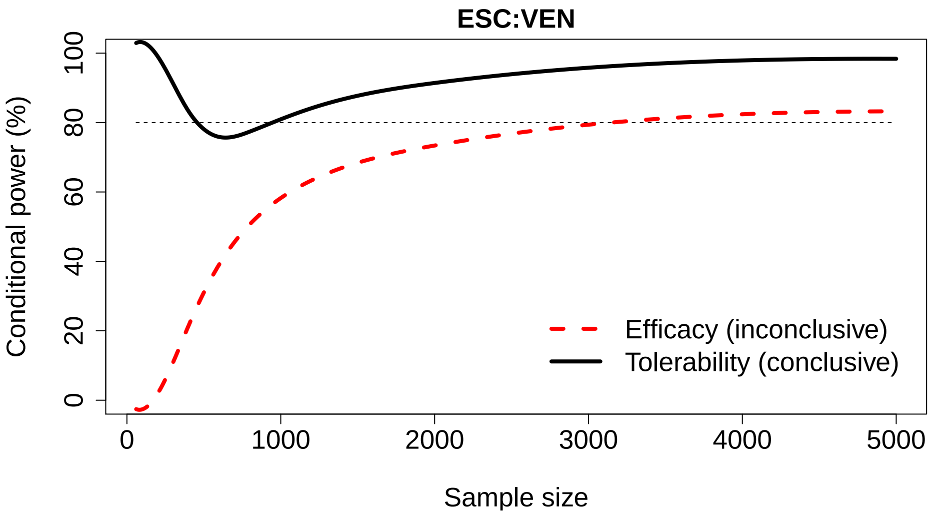

Figure 152:

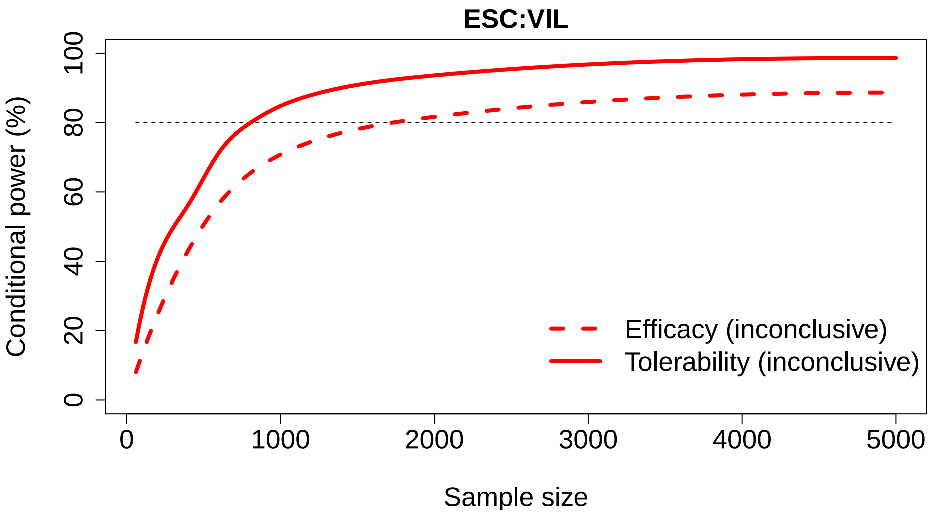

Figure 153:

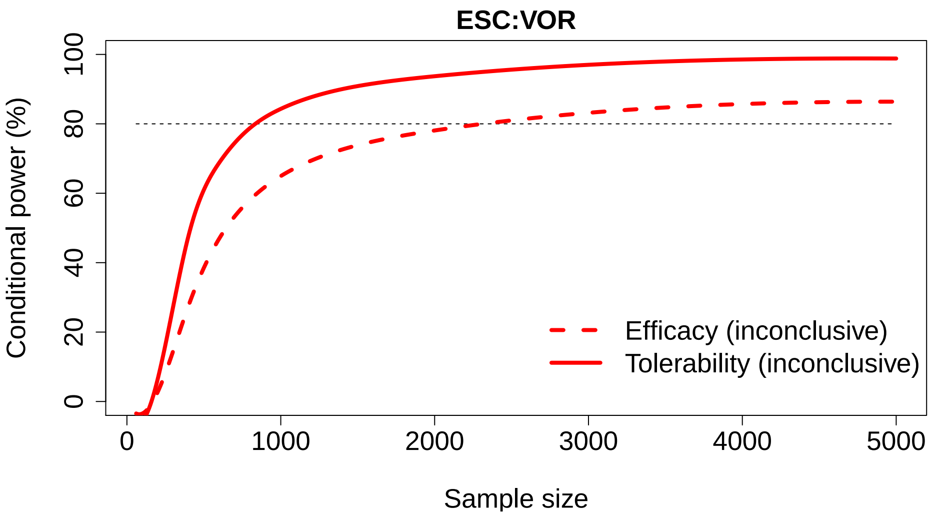

Figure 154:

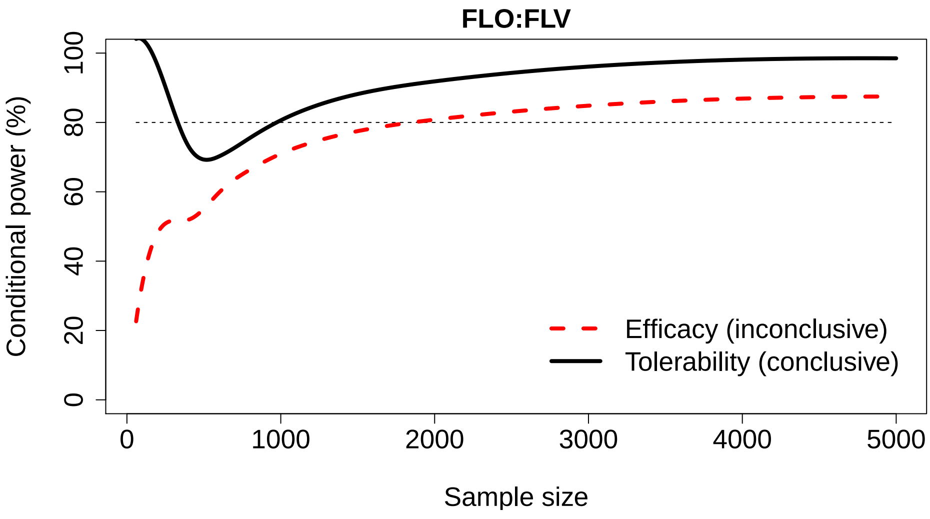

Figure 155:

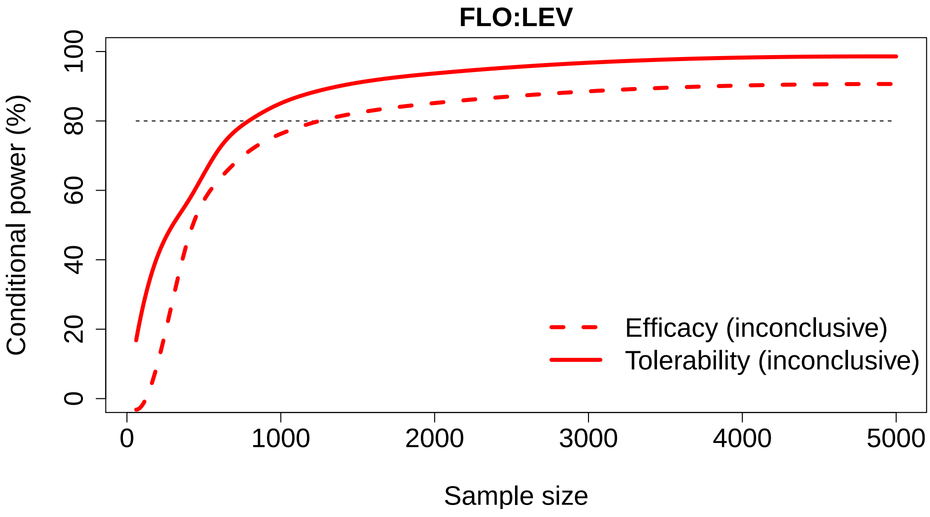

Figure 156:

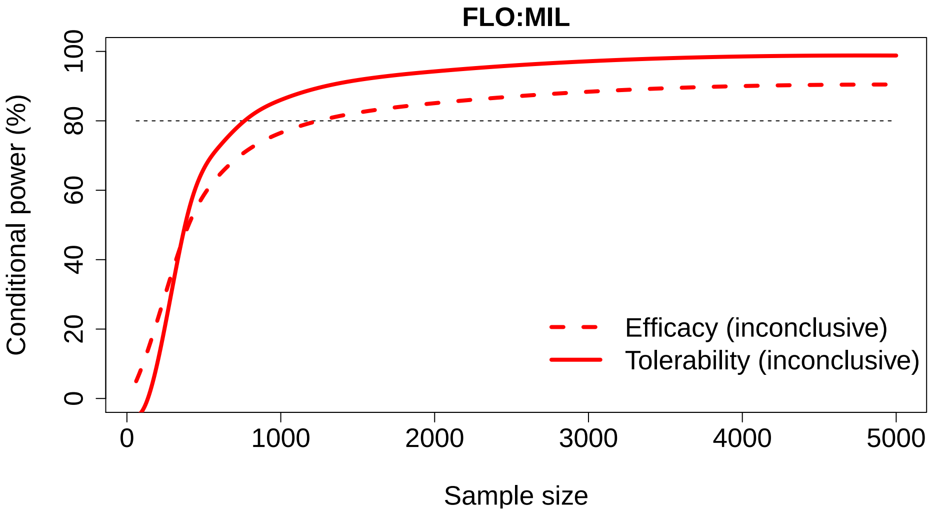

Figure 157:

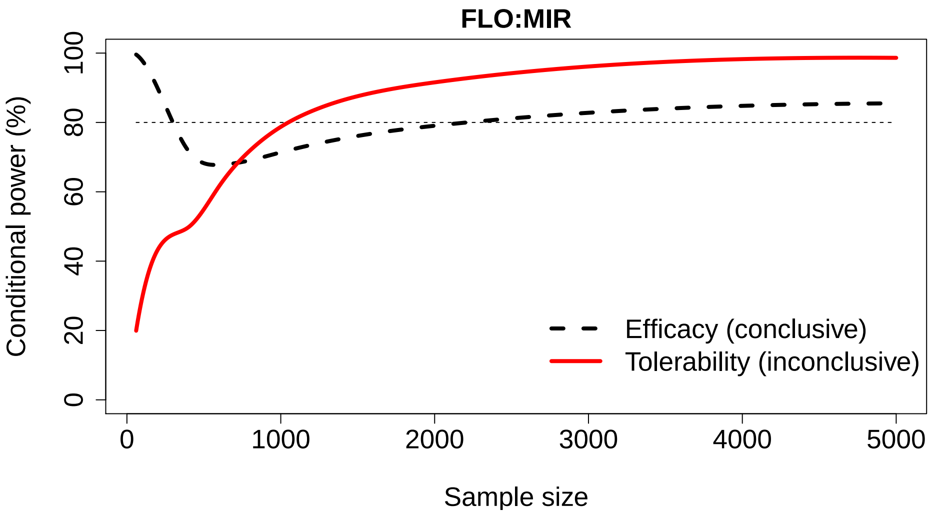

Figure 158:

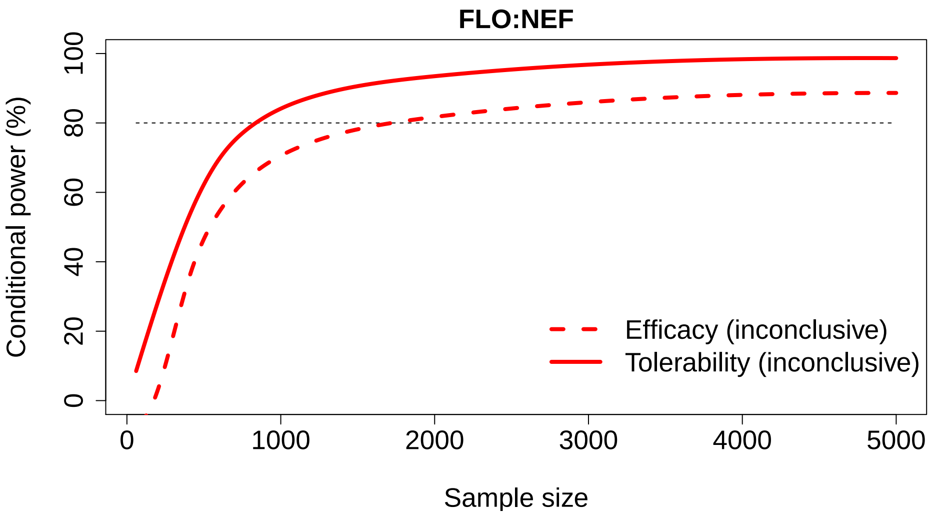

Figure 159:

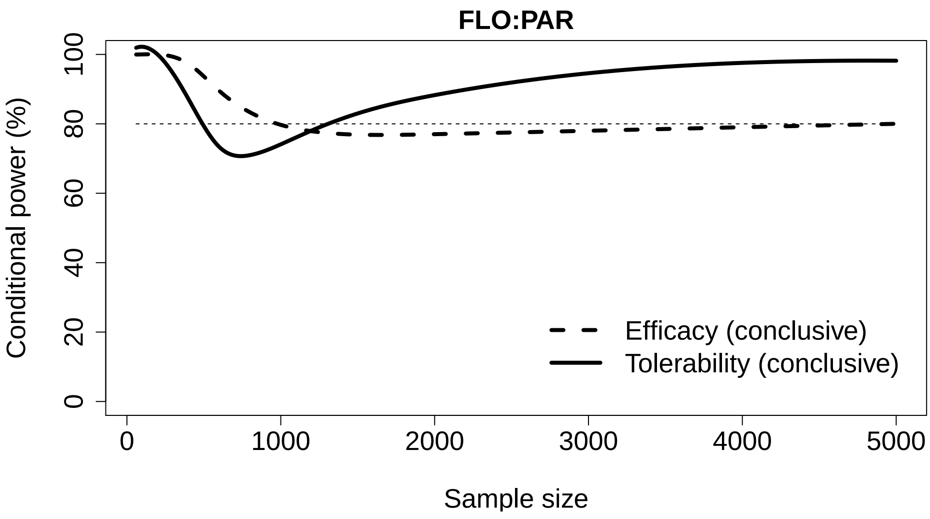

Figure 160:

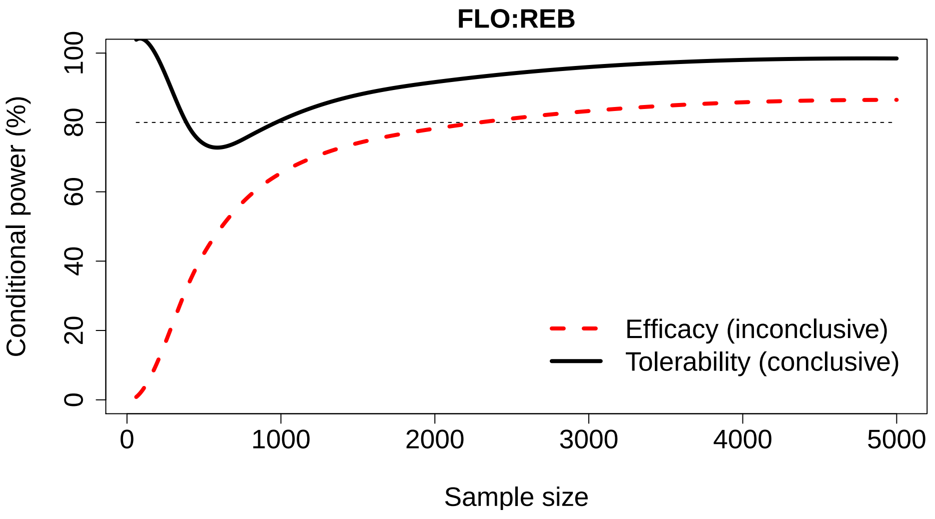

Figure 161:

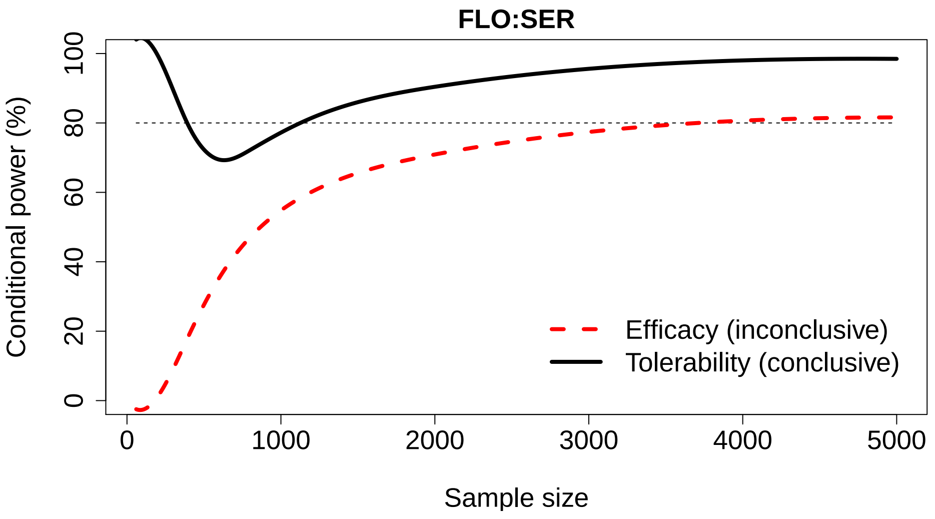

Figure 162:

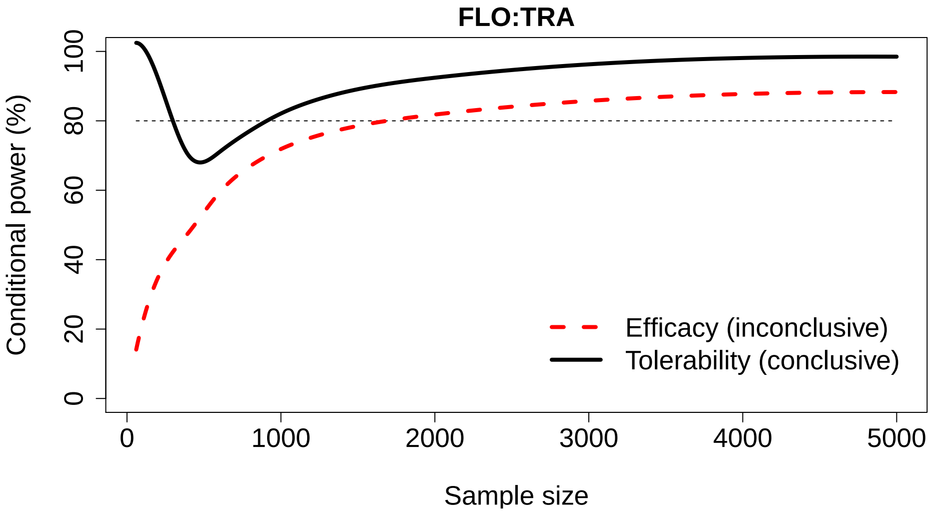

Figure 163:

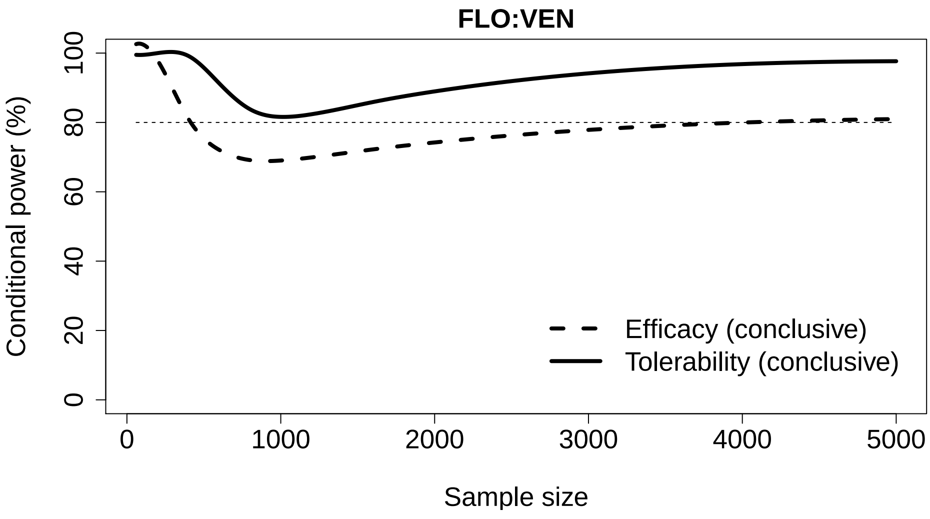

Figure 164:

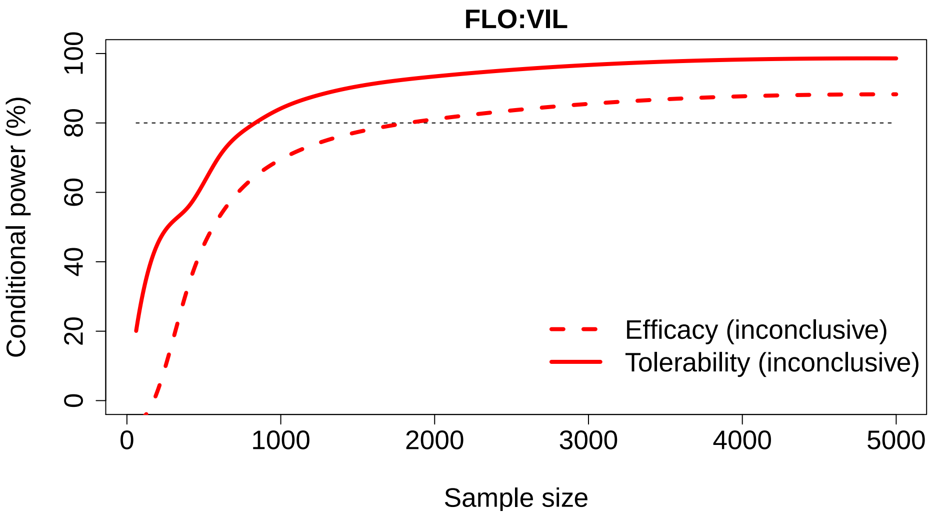

Figure 165:

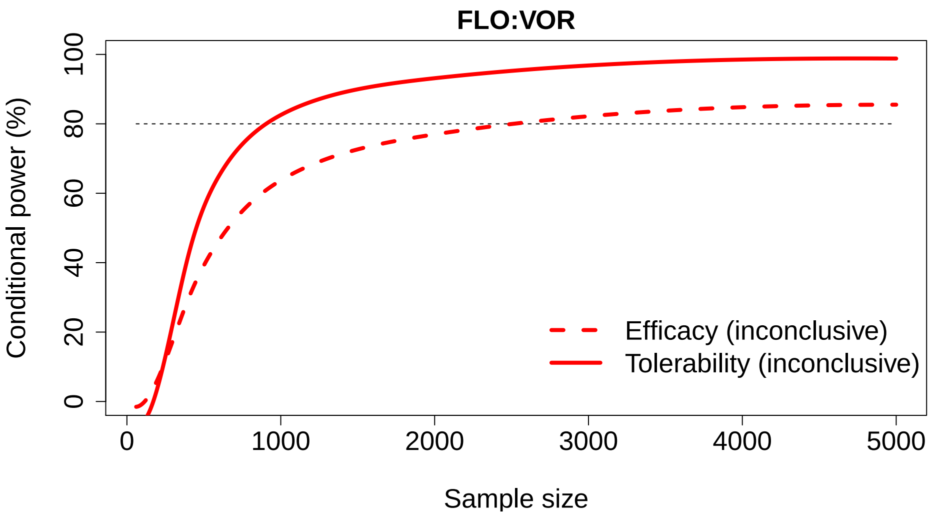

Figure 166:

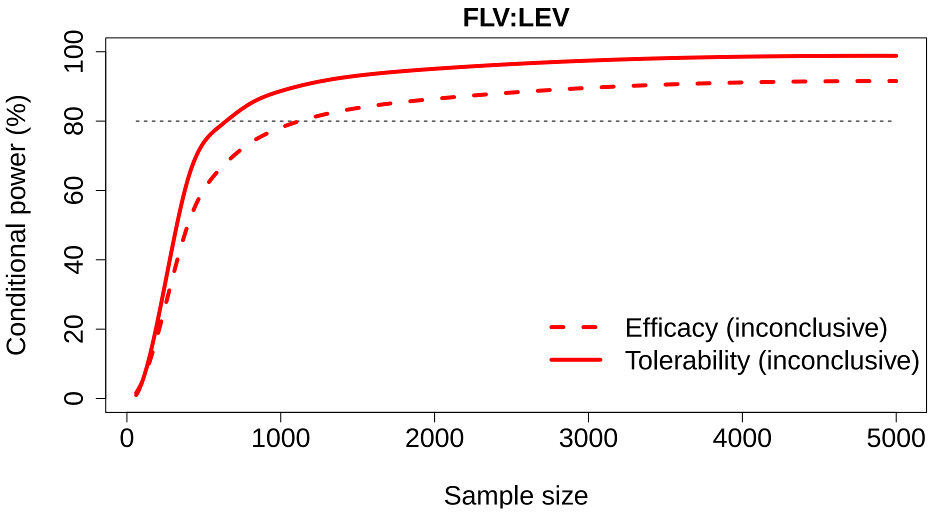

Figure 167:

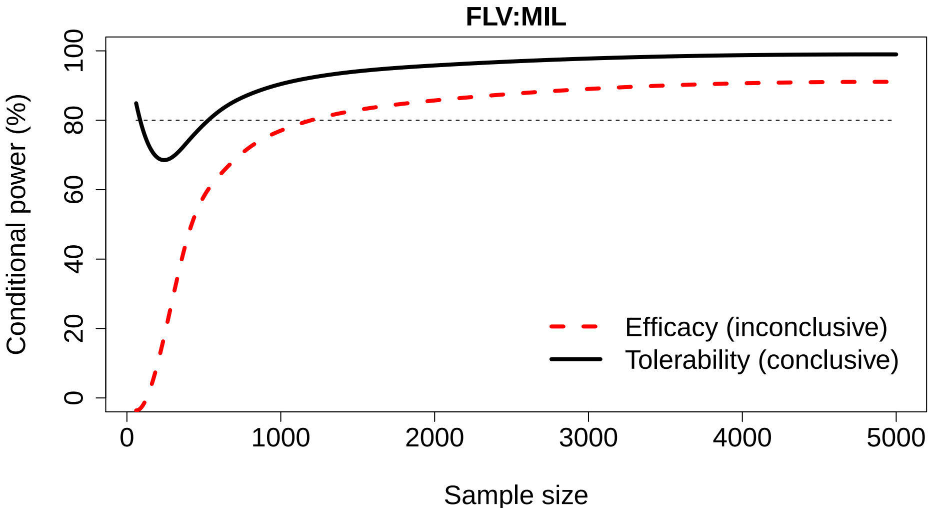

Figure 168:

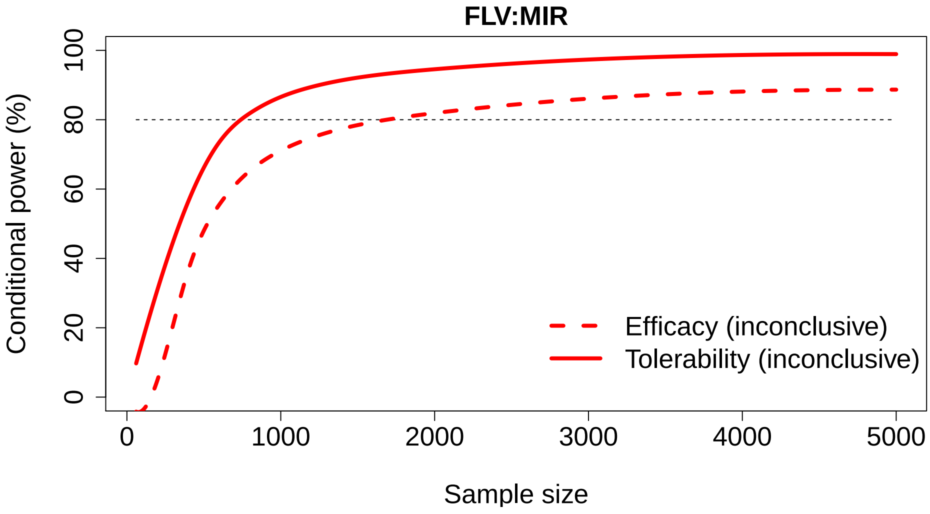

Figure 169:

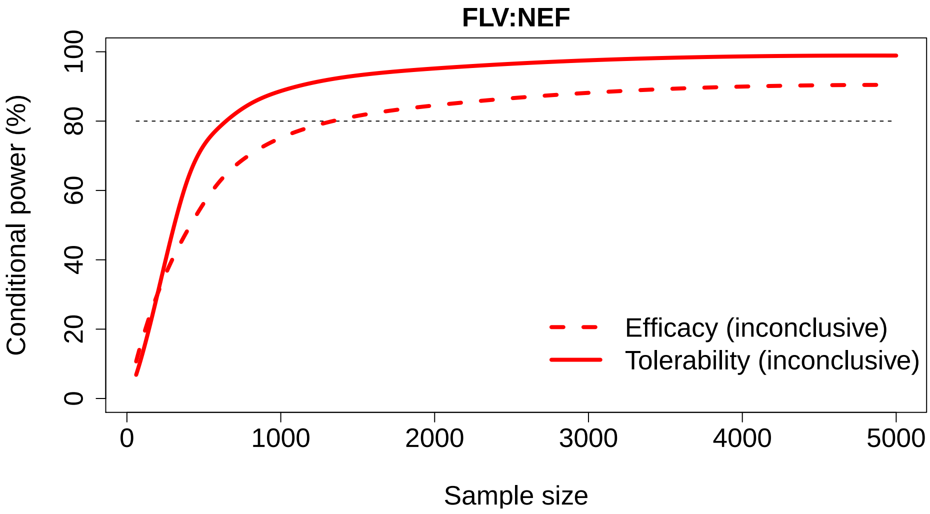

Figure 170:

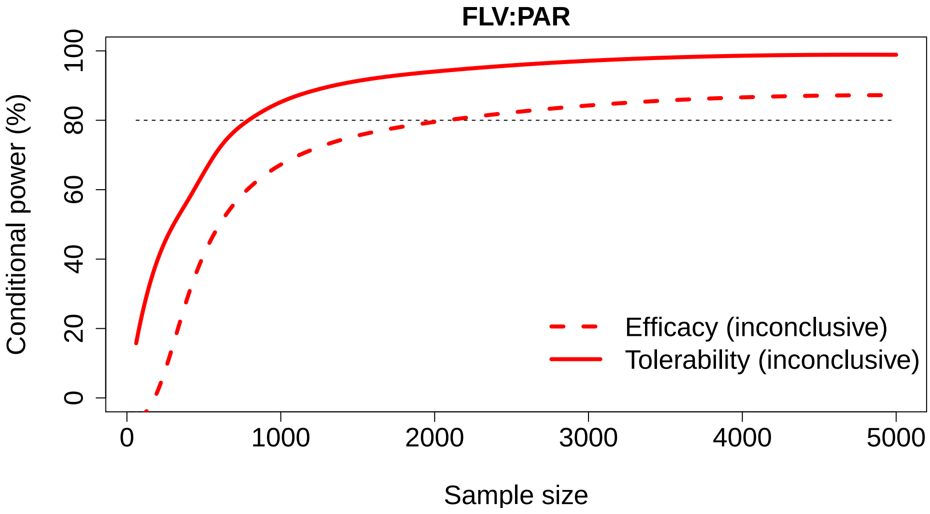

Figure 171:

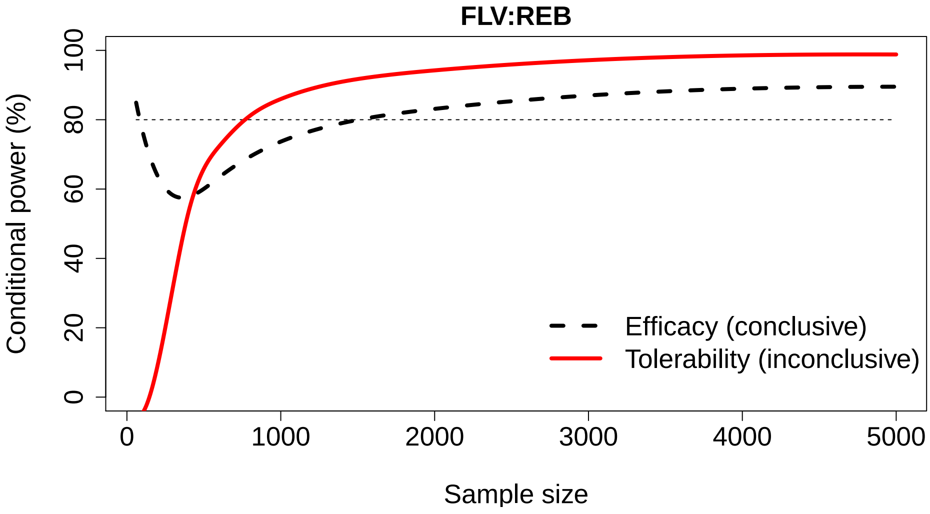

Figure 172:

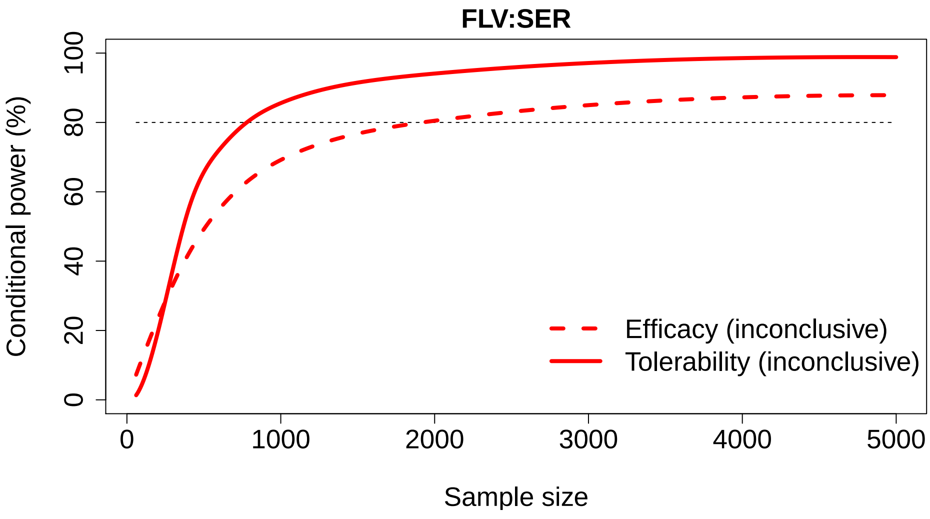

Figure 173:

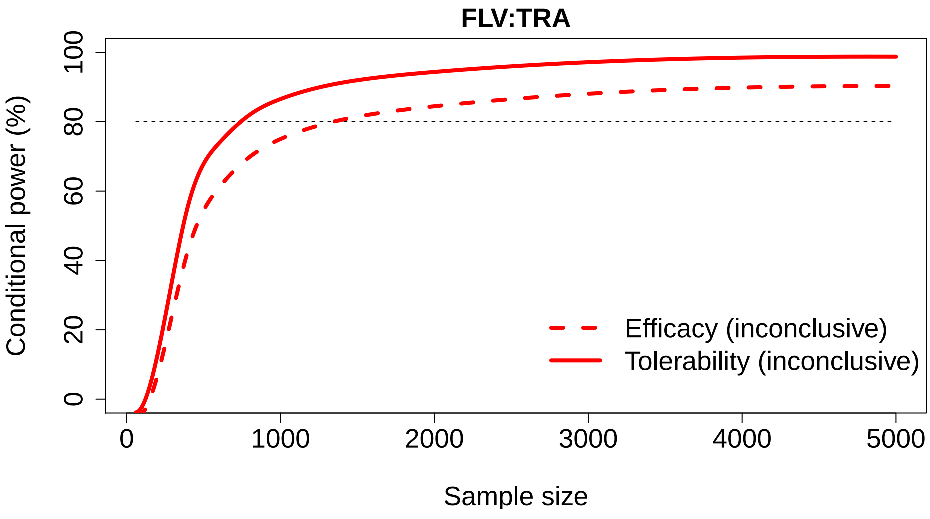

Figure 174:

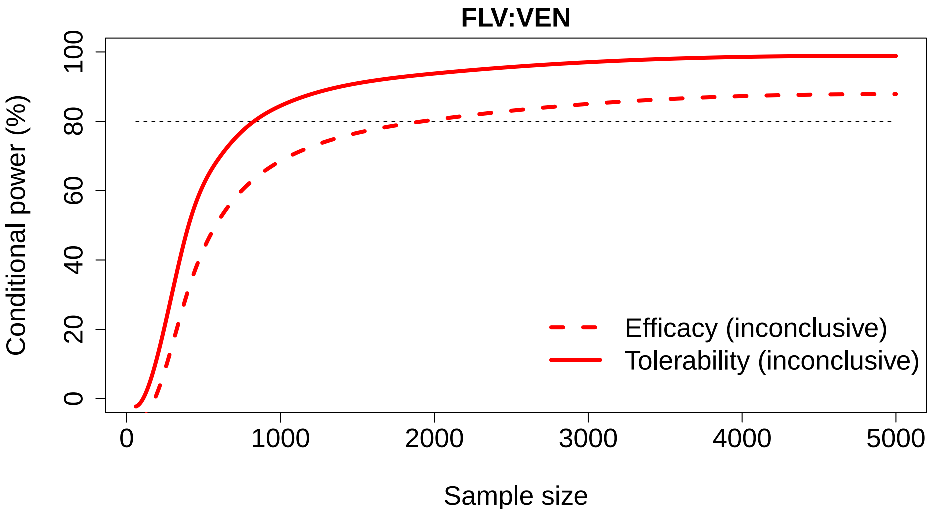

Figure 175:

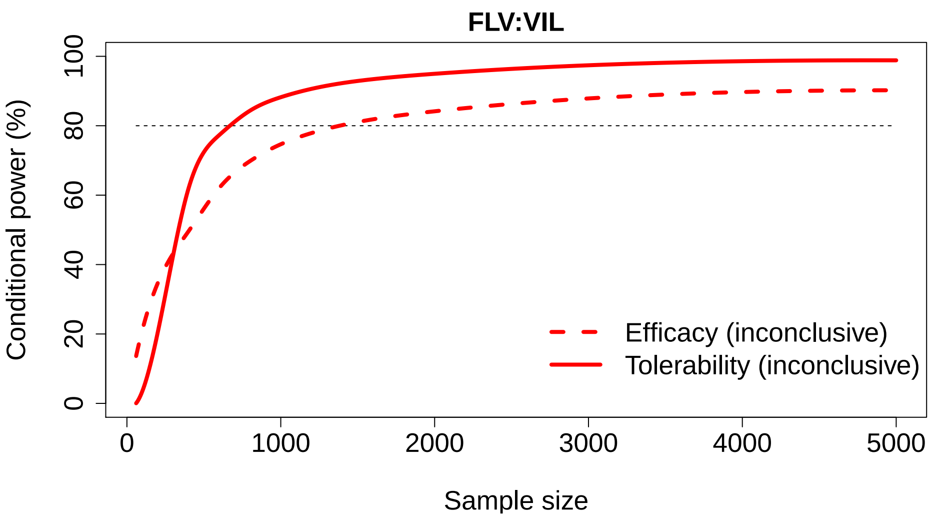

Figure 176:

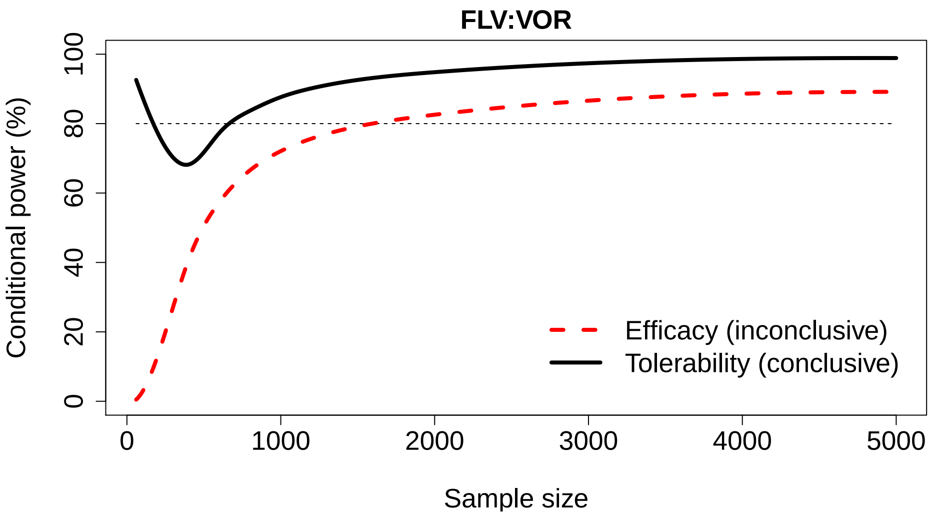

Figure 177:

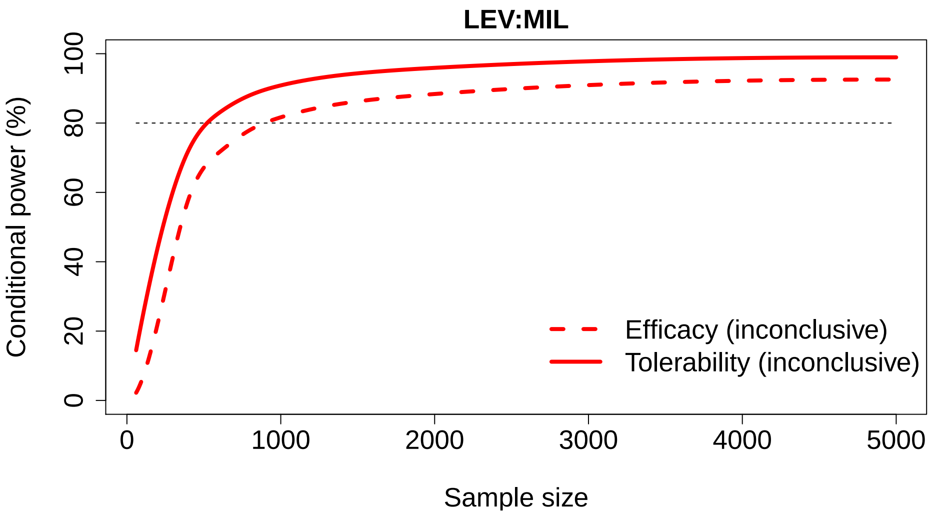

Figure 178:

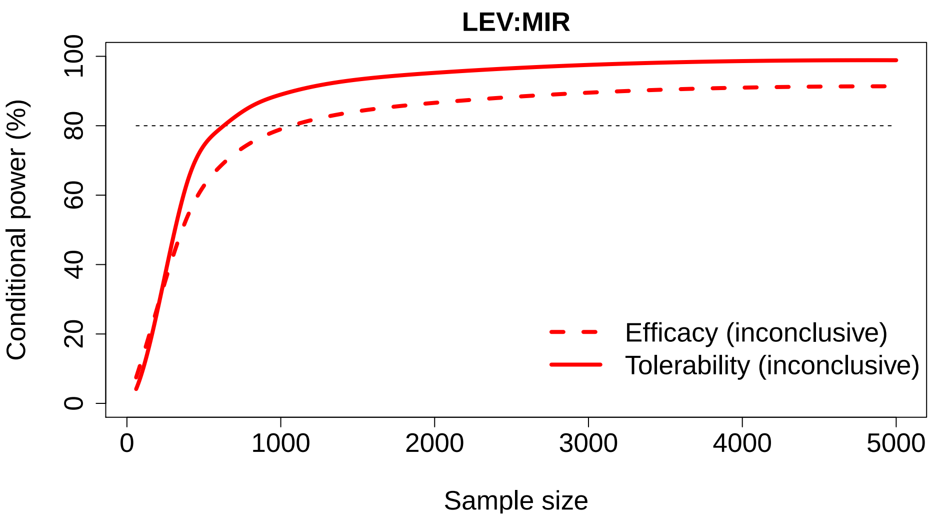

Figure 179:

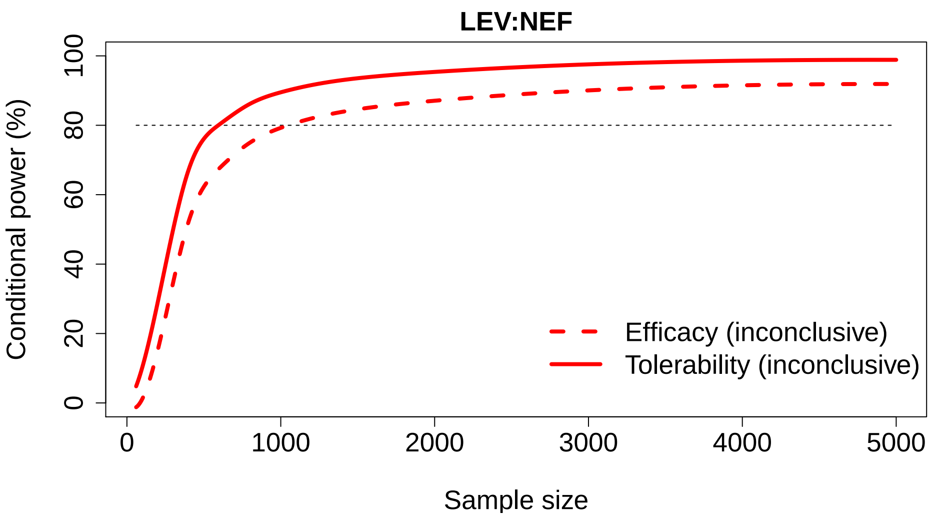

Figure 180:

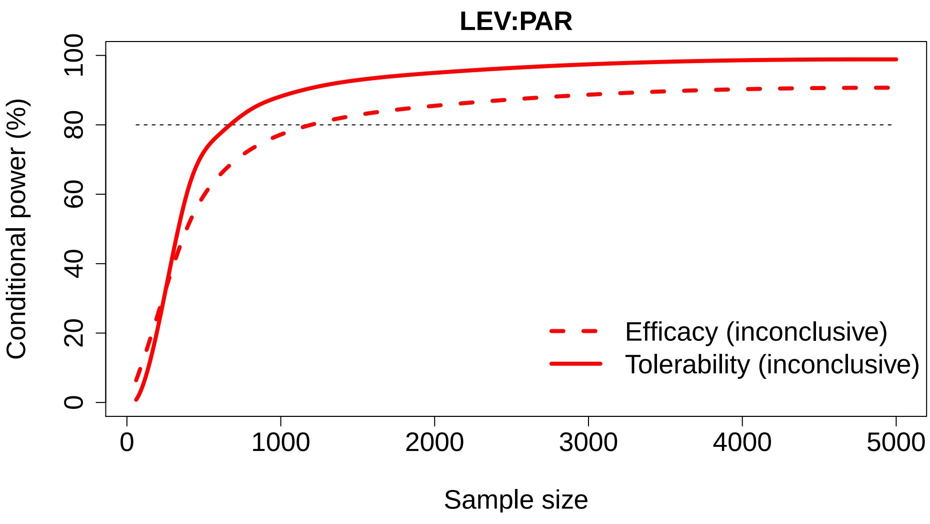

Figure 181:

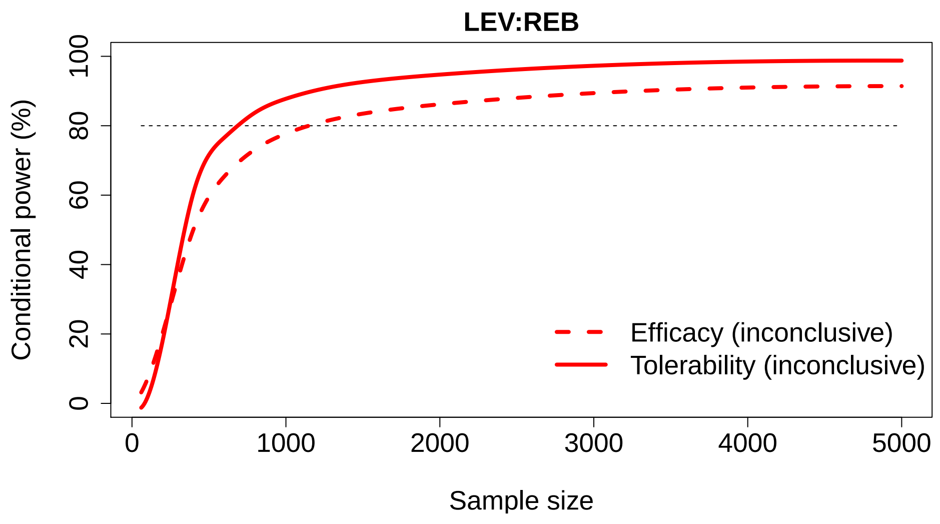

Figure 182:

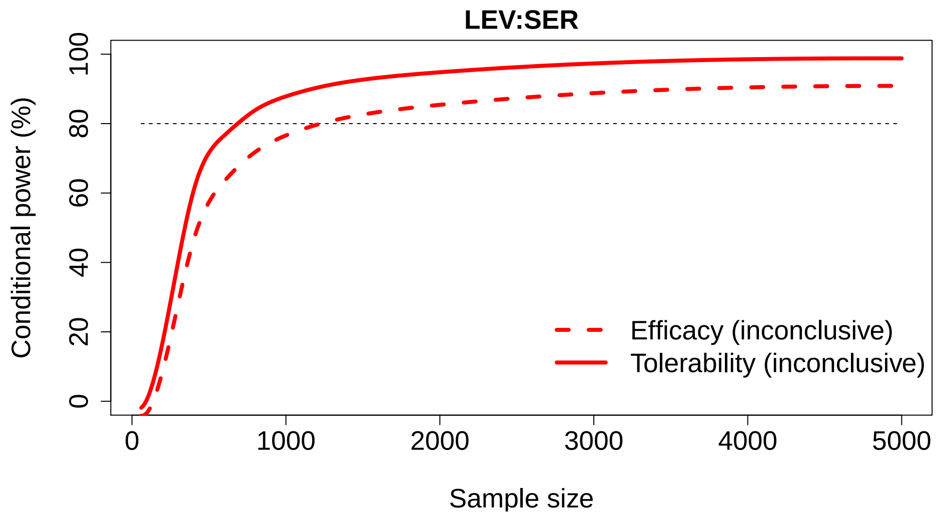

Figure 183:

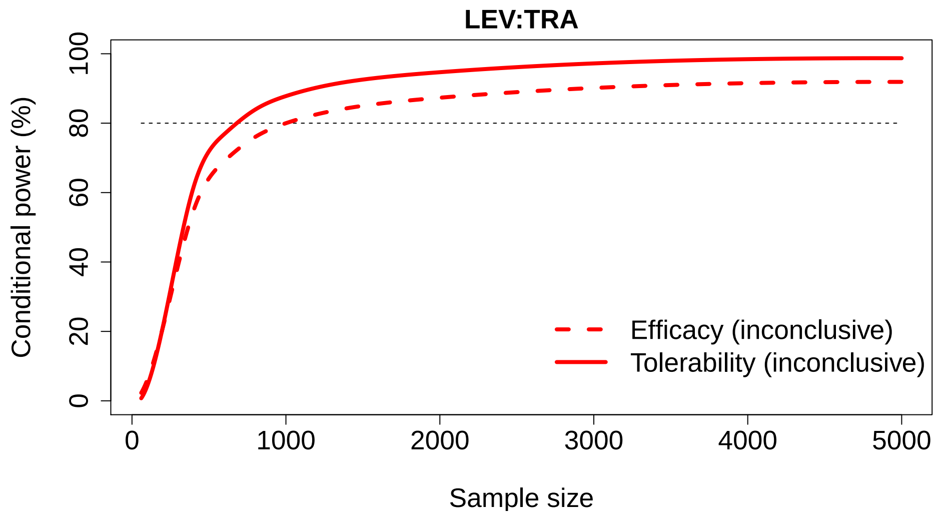

Figure 184:

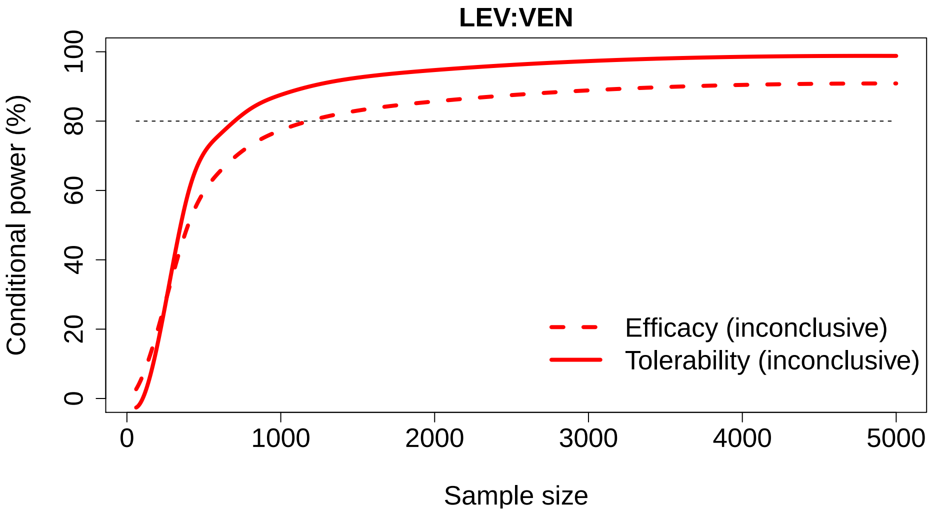

Figure 185:

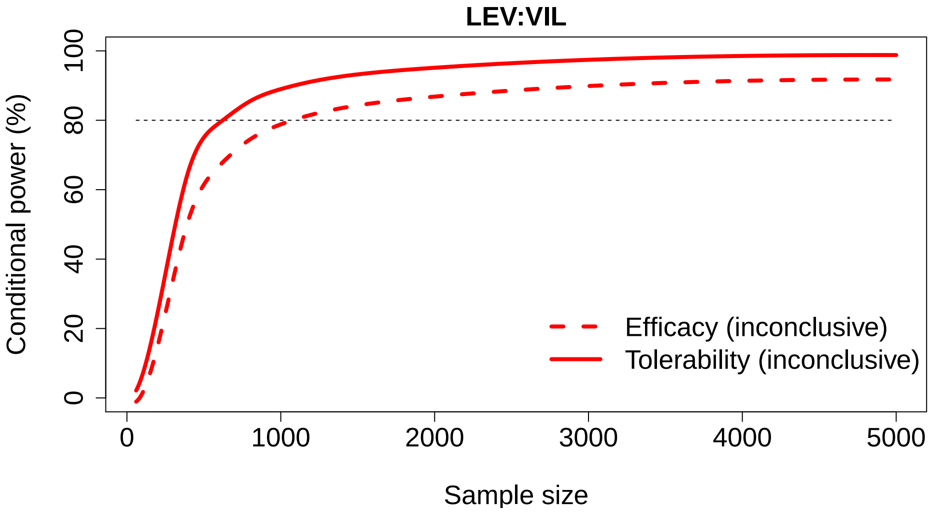

Figure 186:

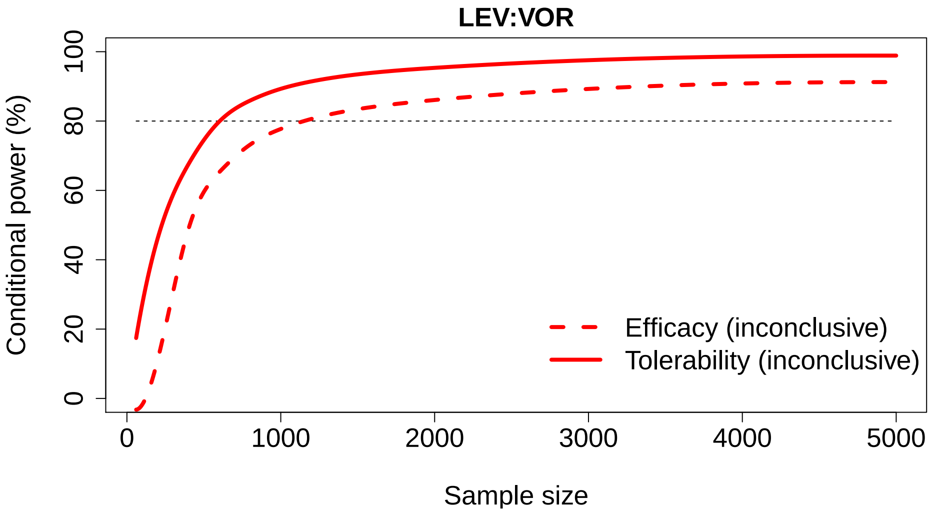

Figure 187:

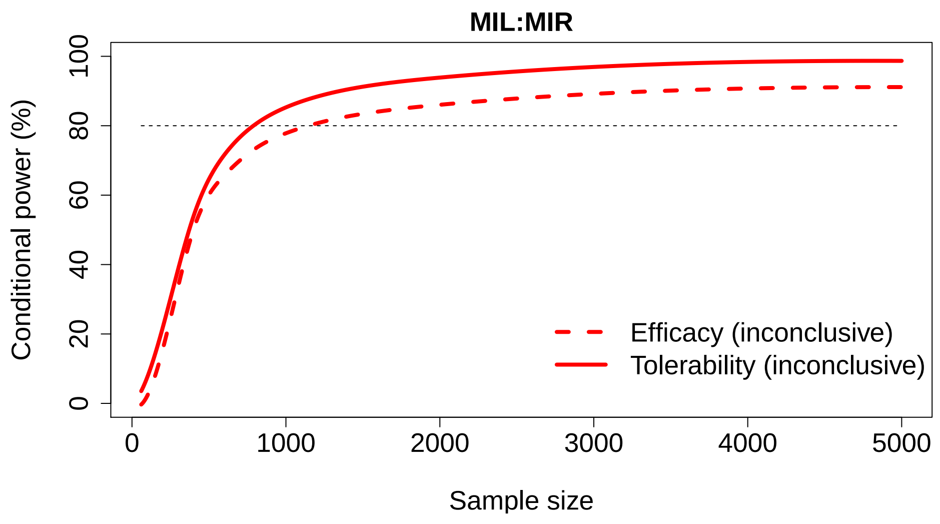

Figure 188:

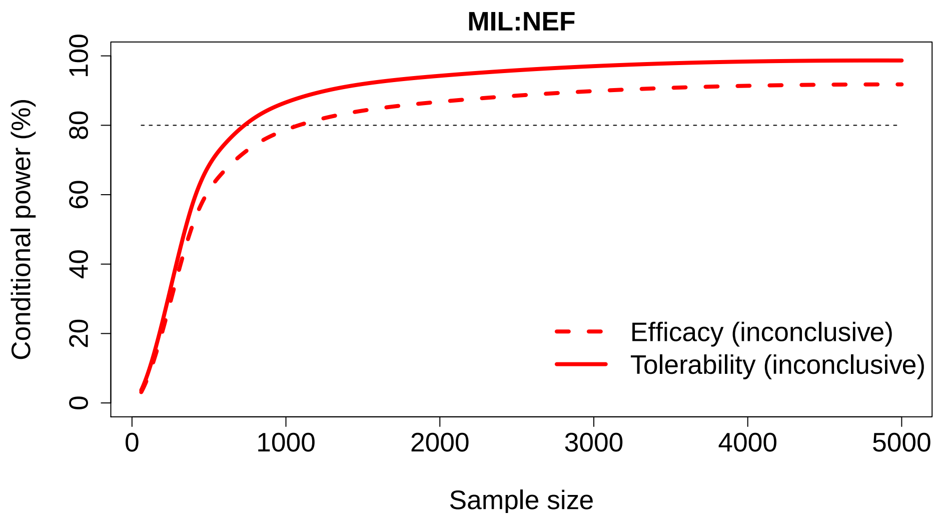

Figure 189:

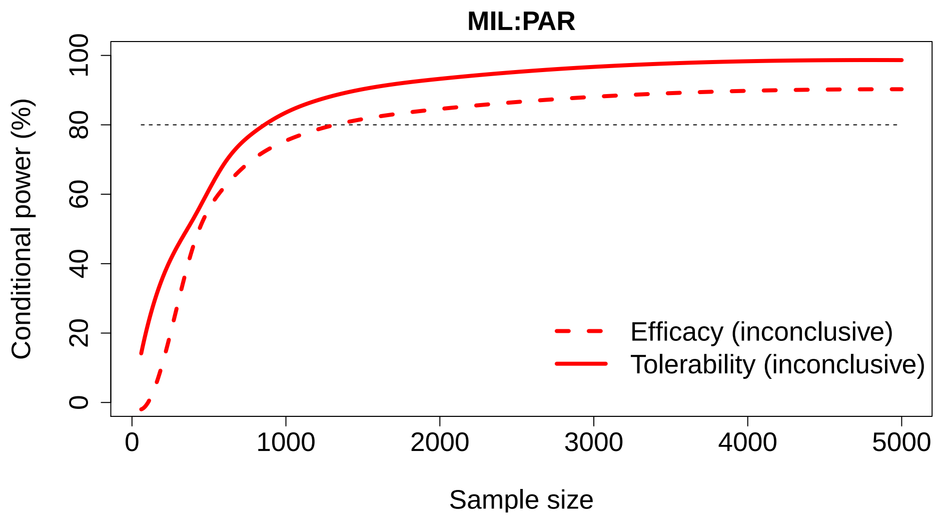

Figure 190:

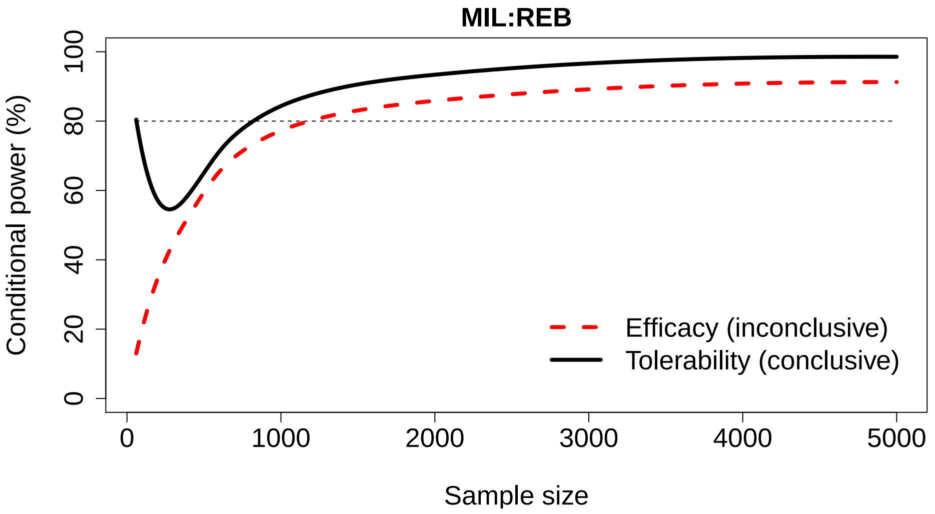

Figure 191:

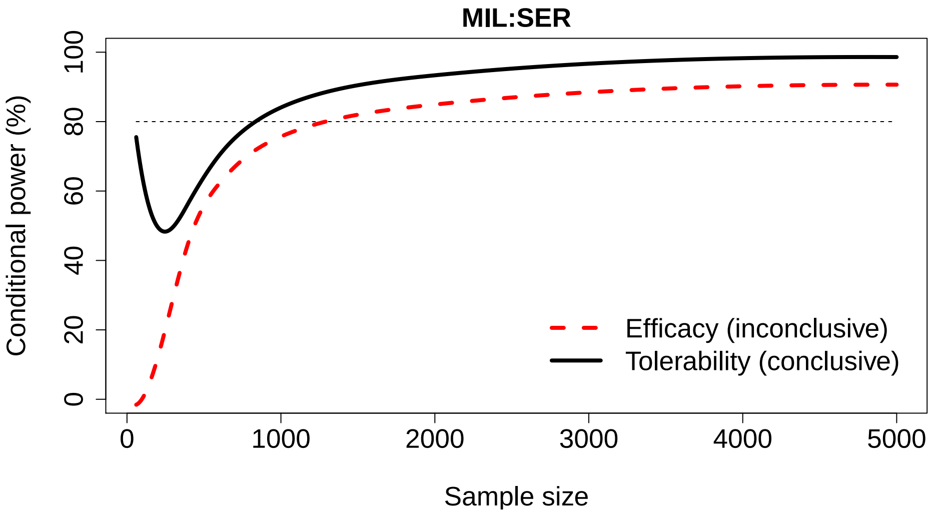

Figure 192:

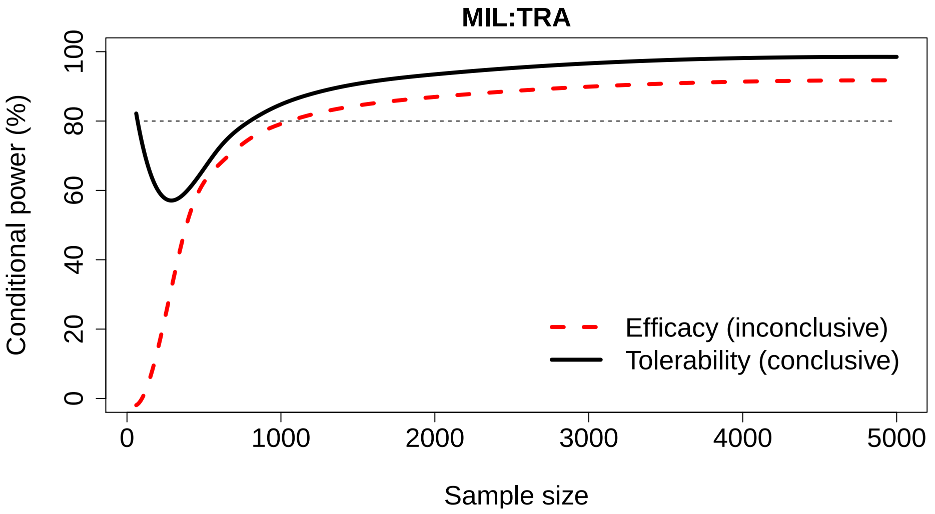

Figure 193:

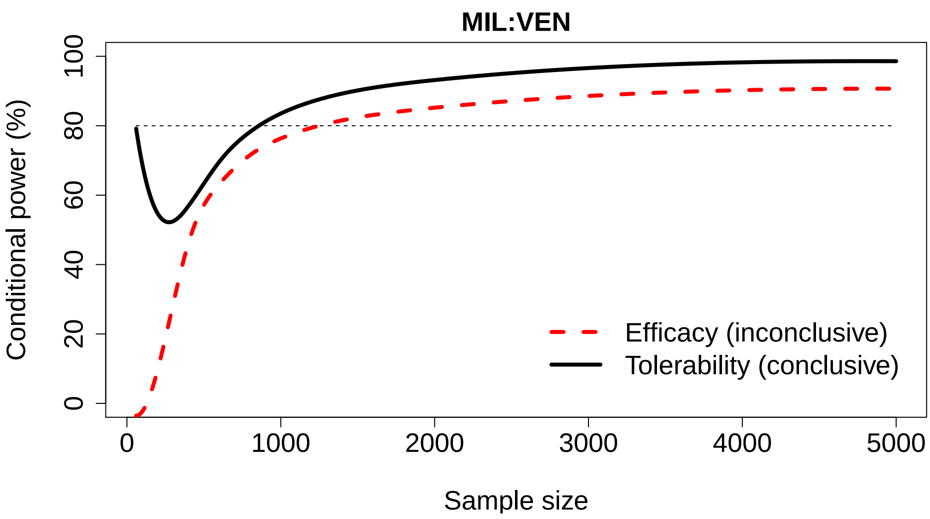

Figure 194:

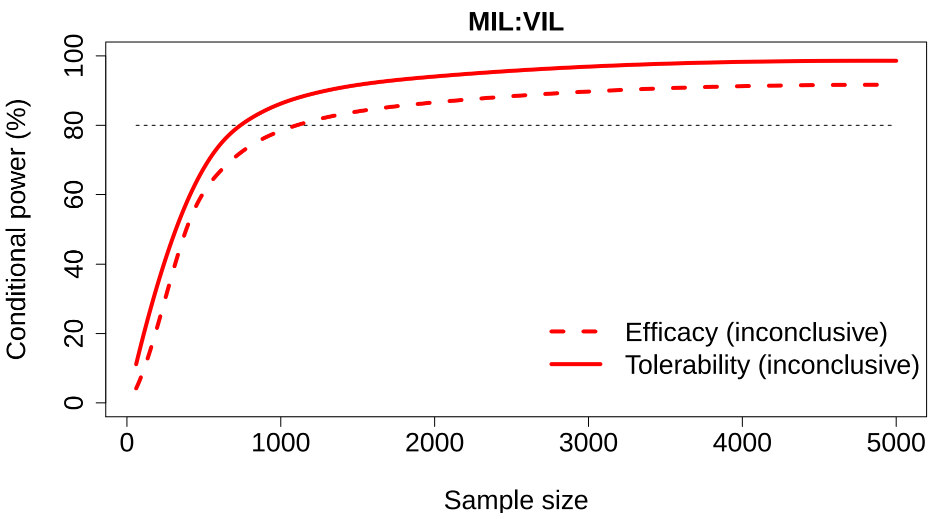

Figure 195:

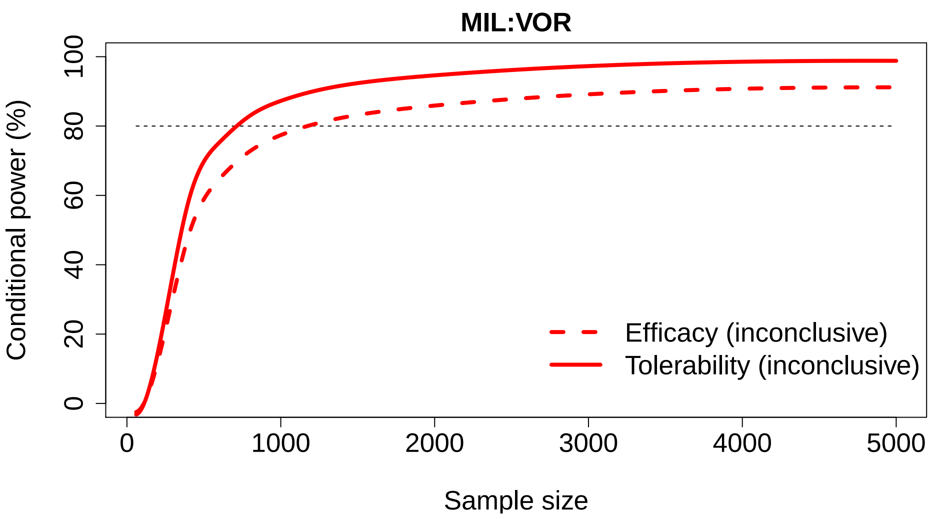

Figure 196:

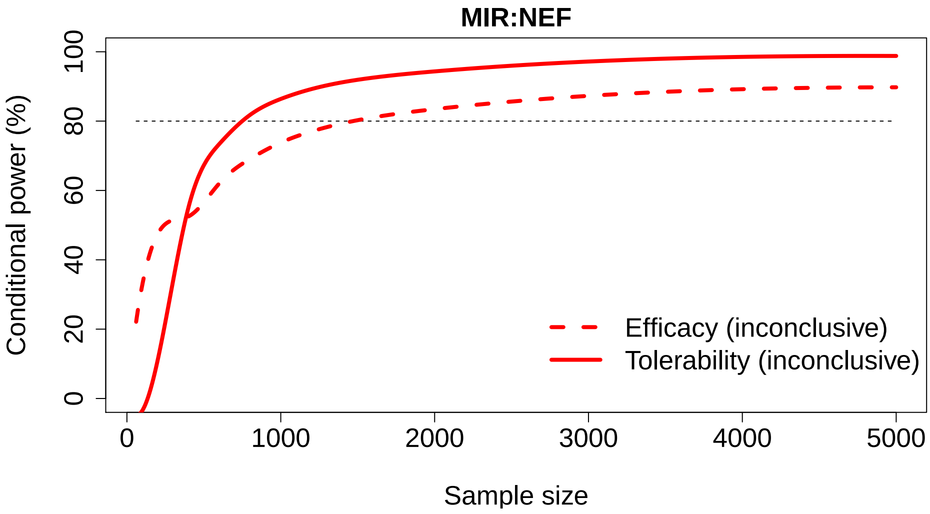

Figure 197:

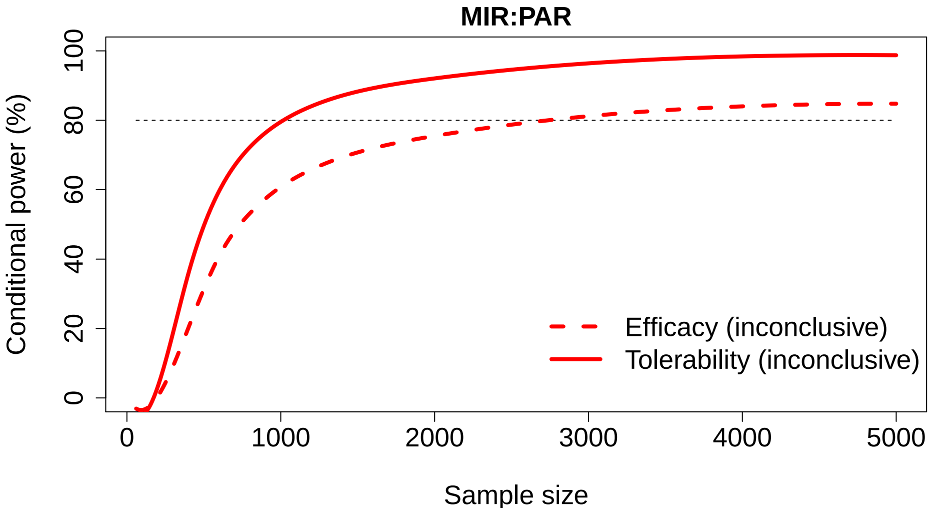

Figure 198:

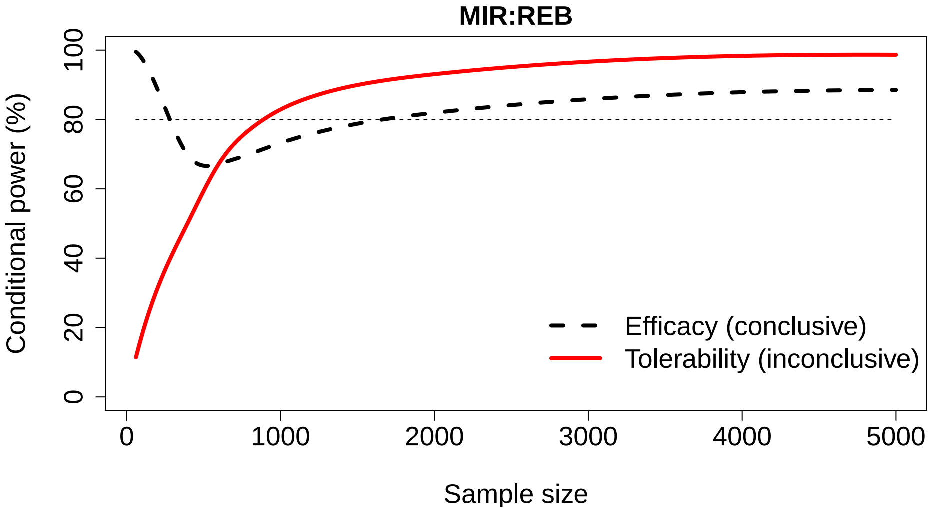

Figure 199:

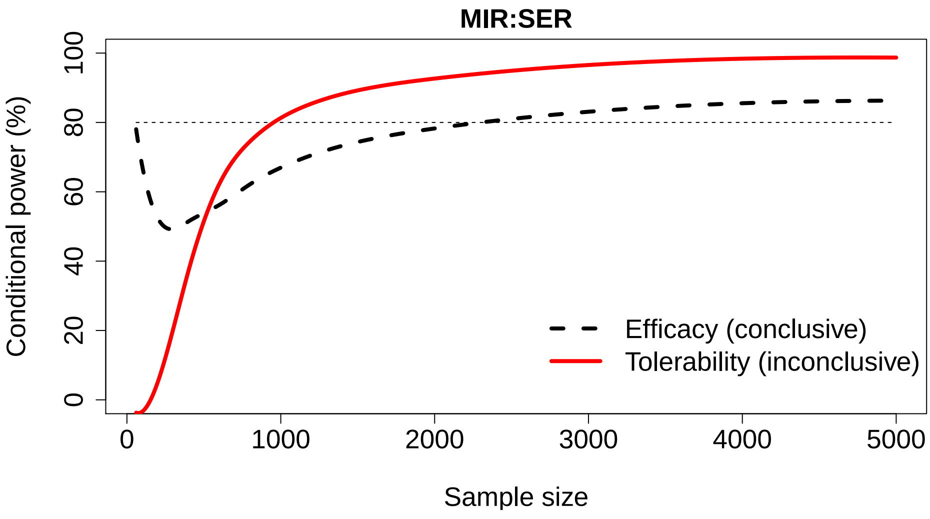

Figure 200:

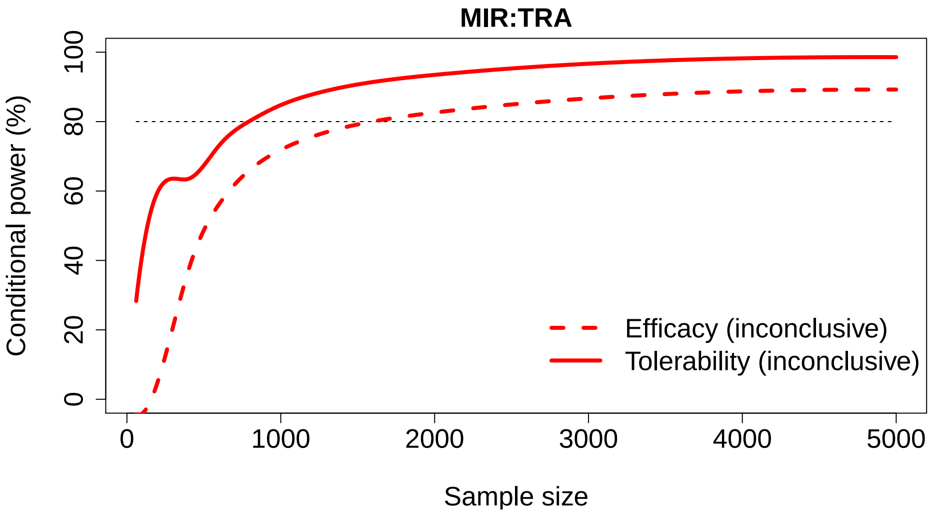

Figure 201:

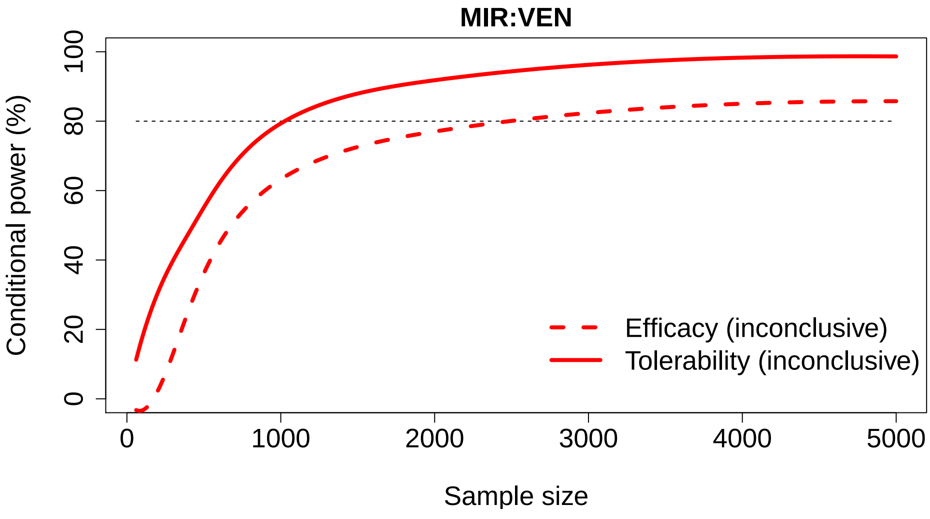

Figure 202:

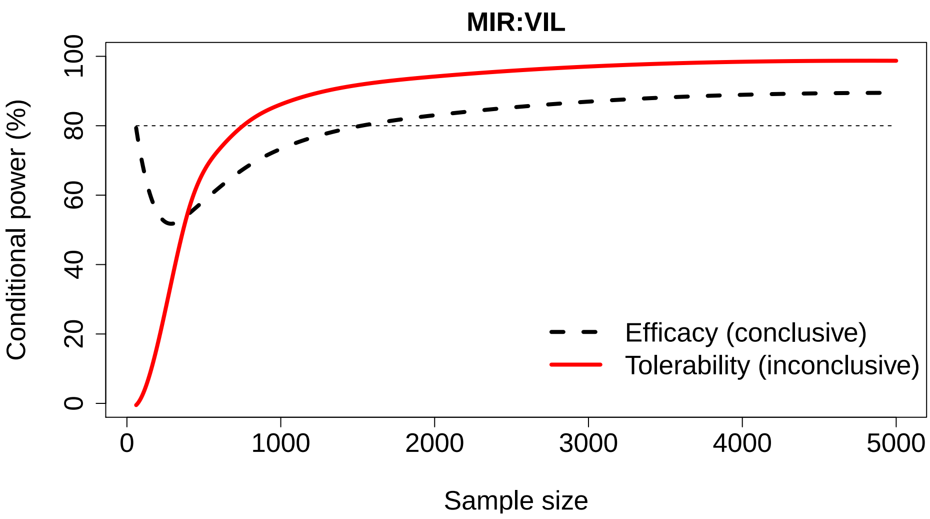

Figure 203:

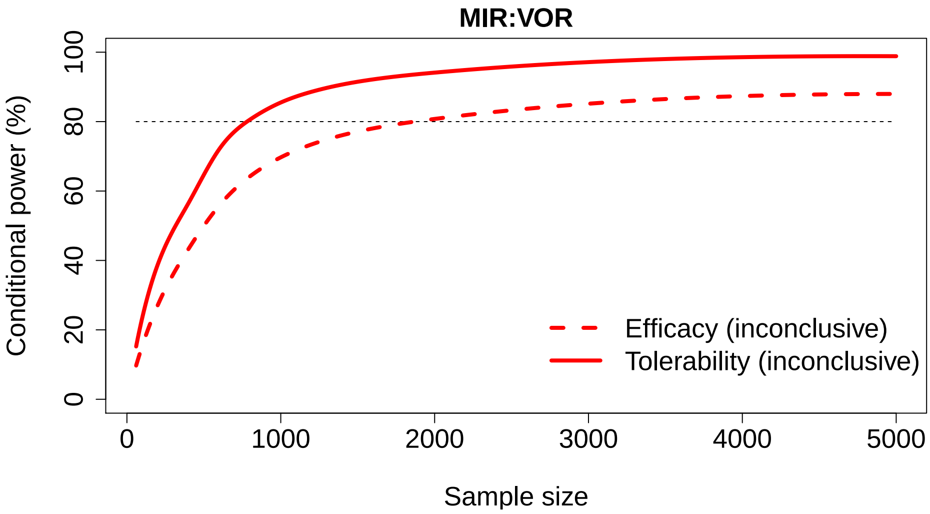

Figure 204:

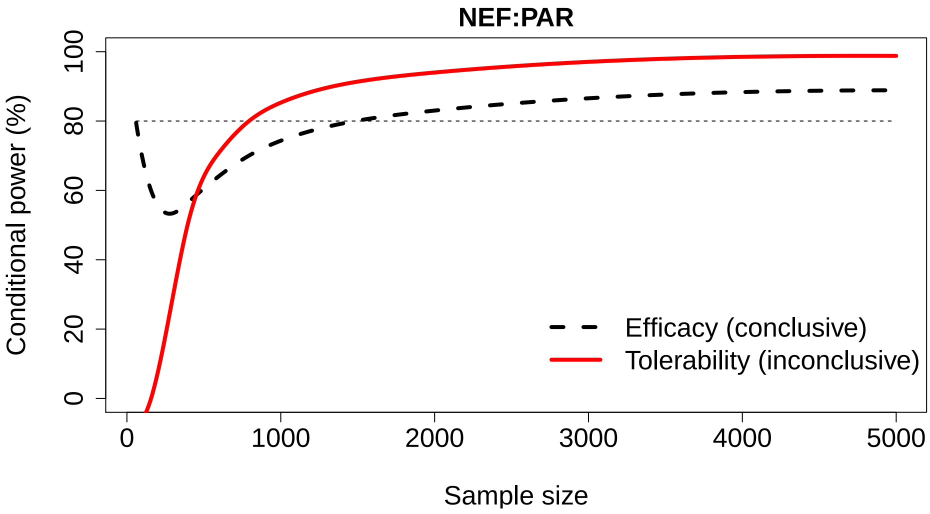

Figure 205:

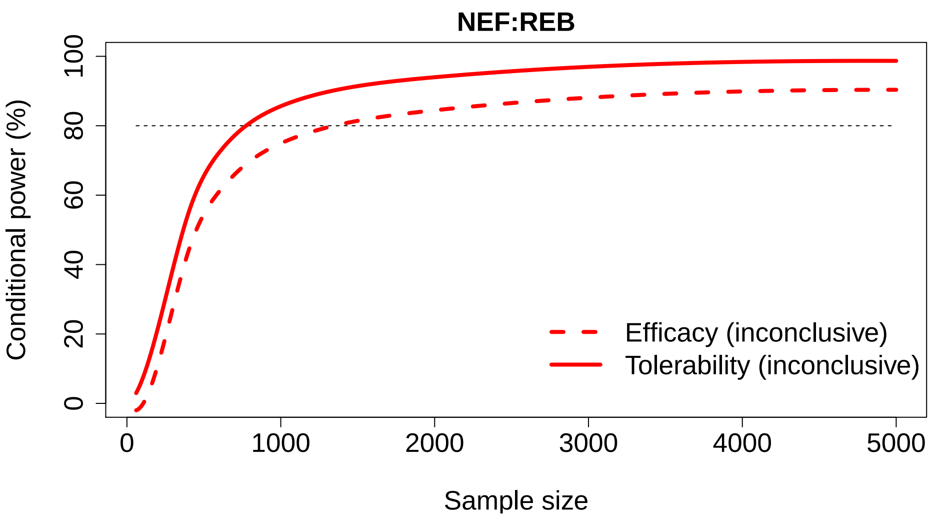

Figure 206:

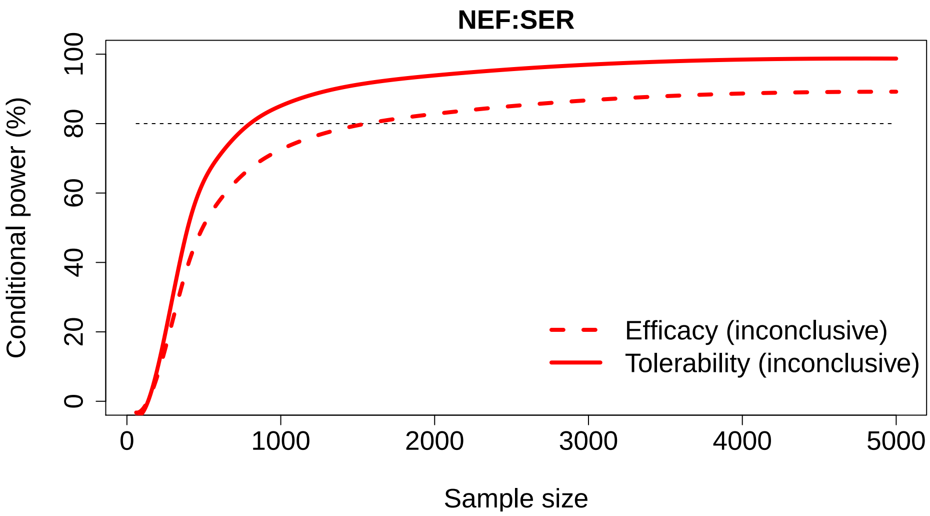

Figure 207:

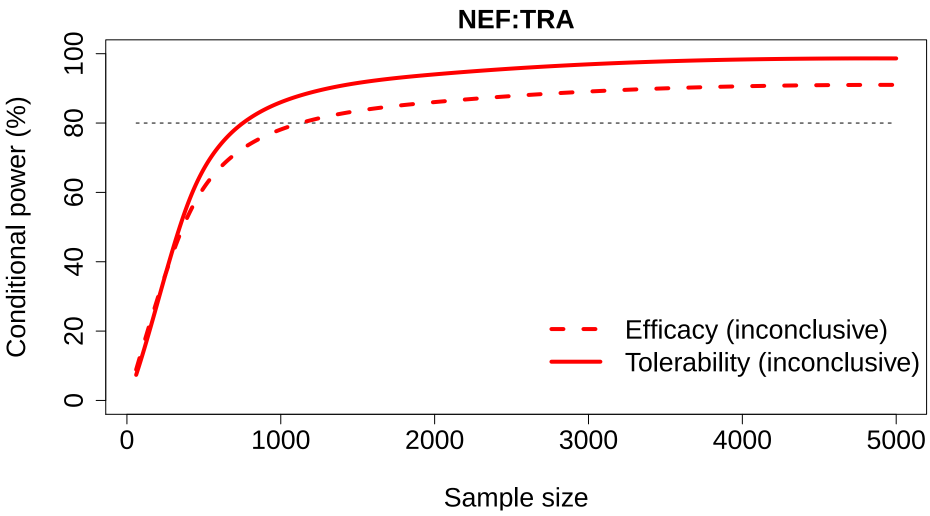

Figure 208:

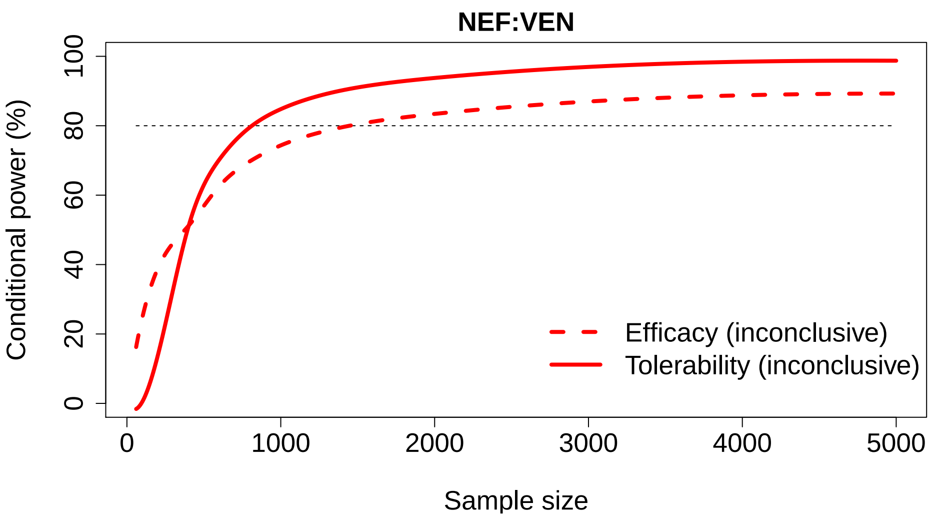

Figure 209:

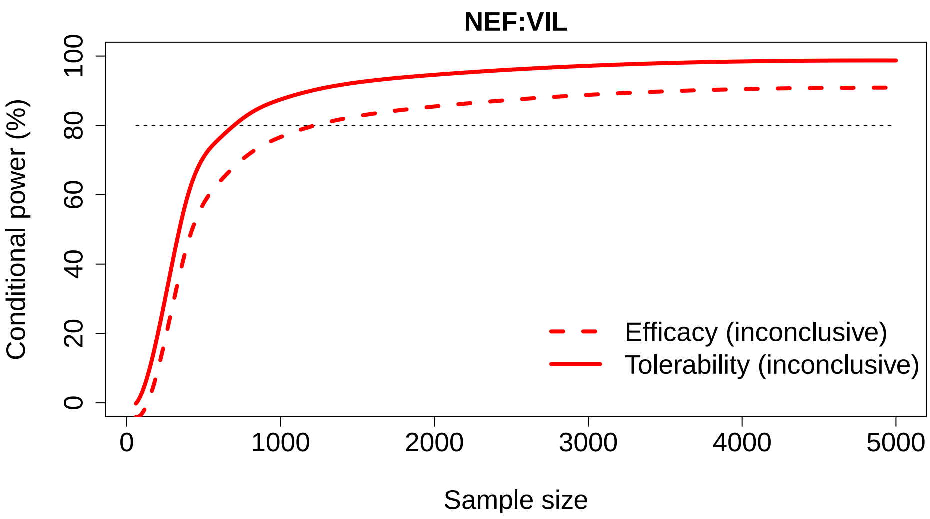

Figure 210:

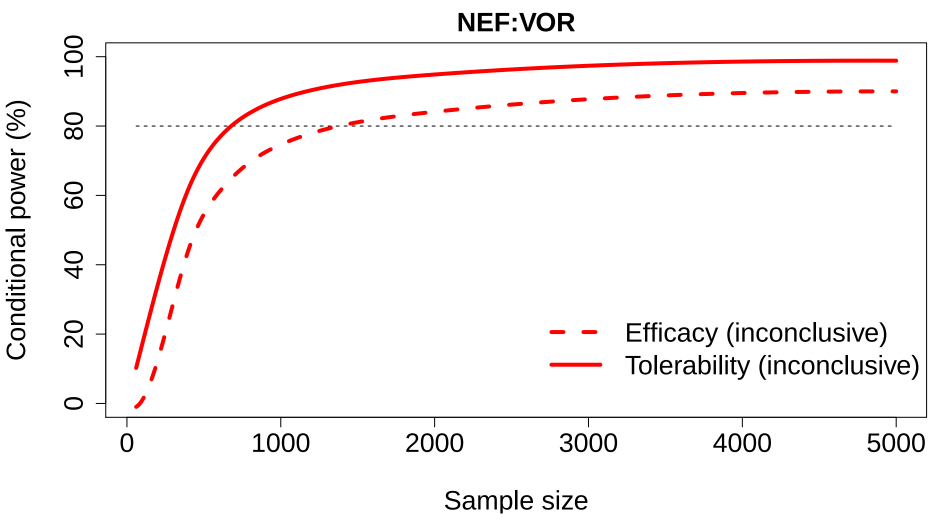

Figure 211:

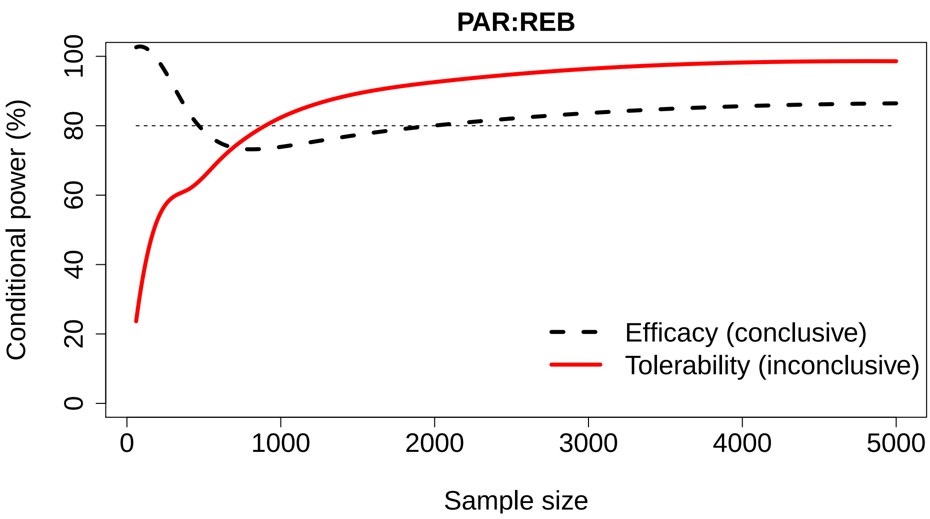

Figure 212:

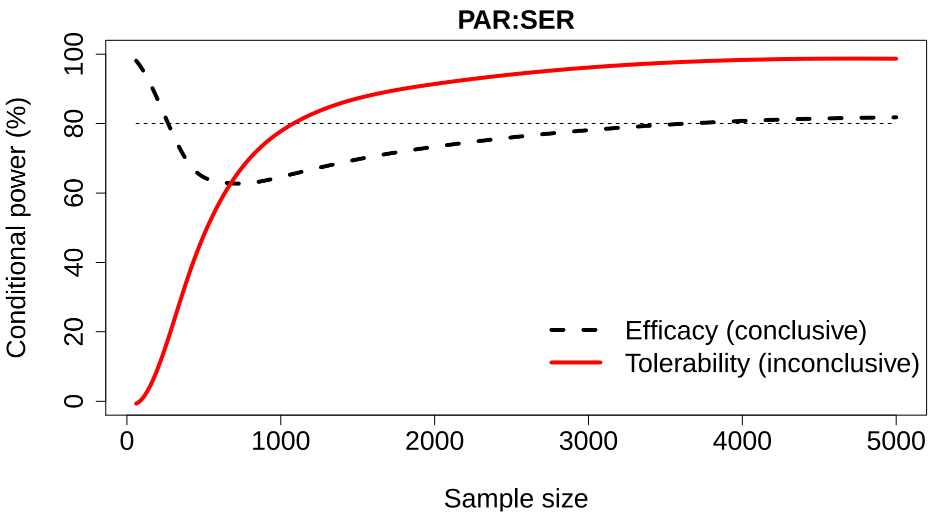

Figure 213:

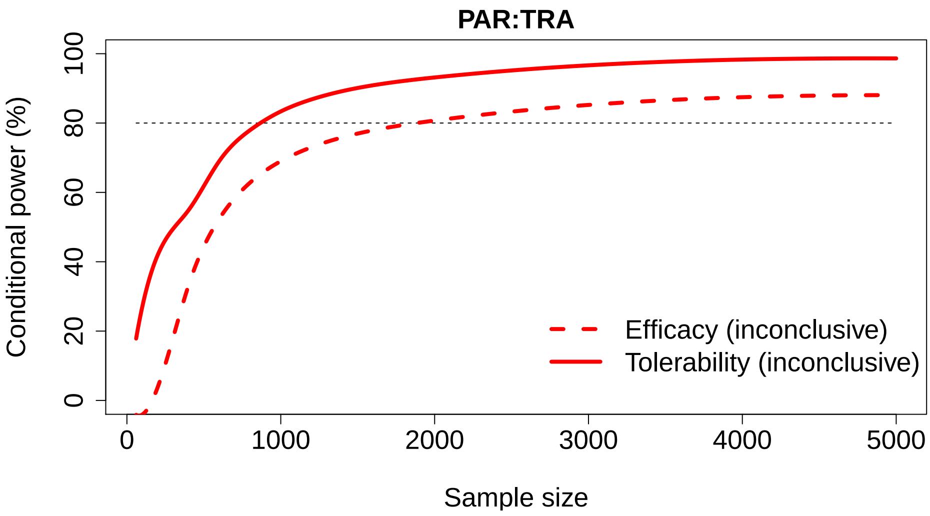

Figure 214:

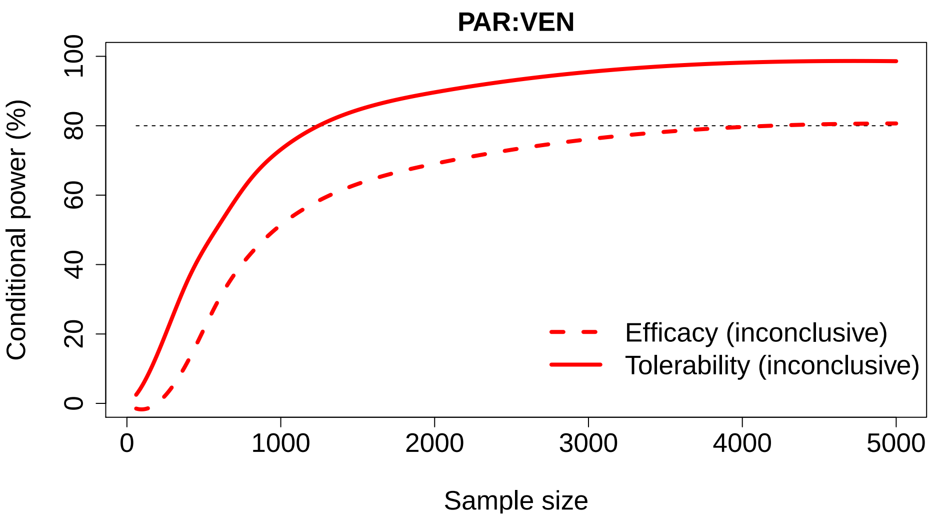

Figure 215:

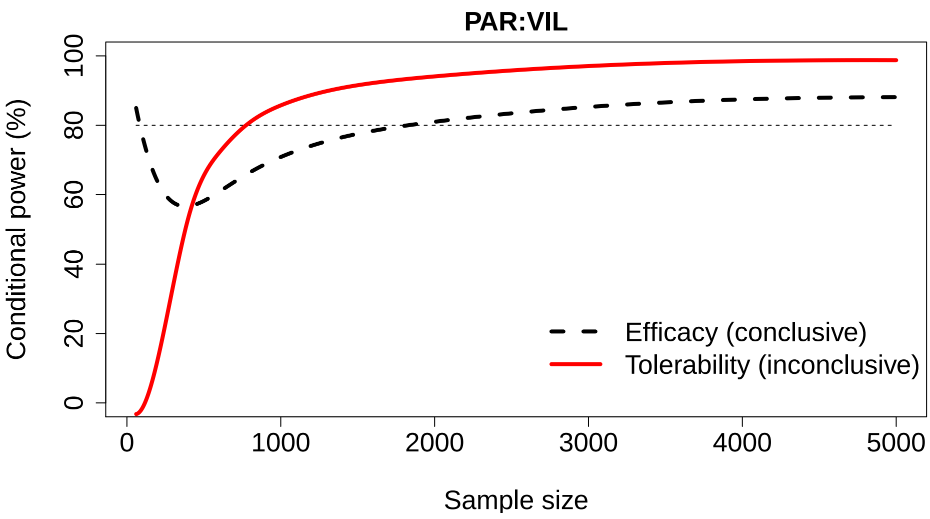

Figure 216:

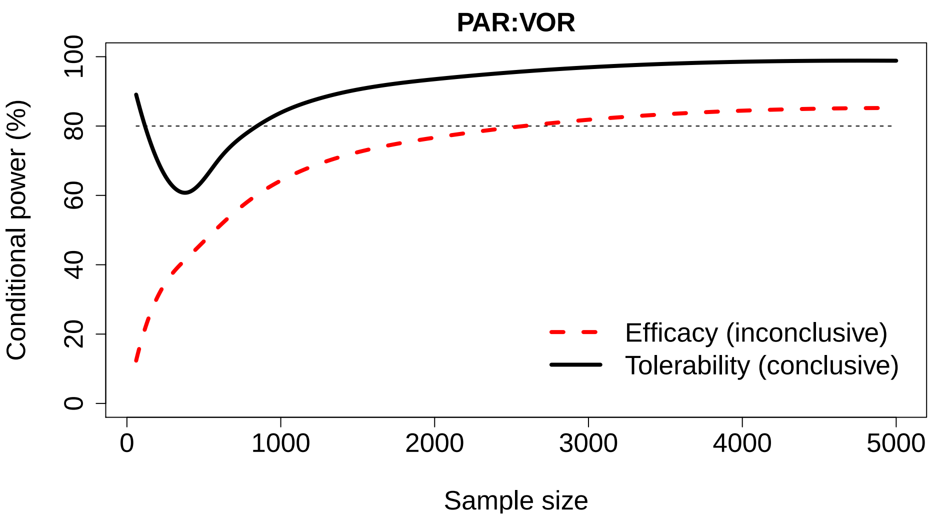

Figure 217:

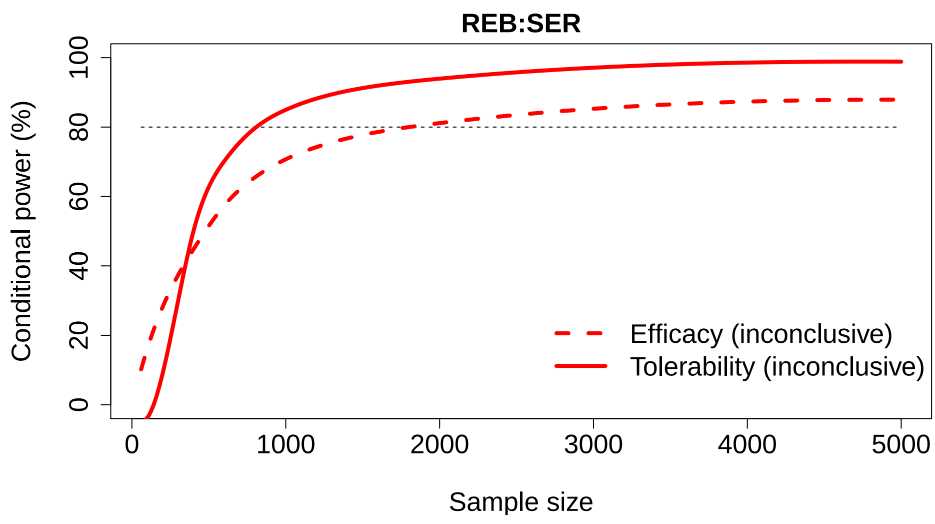

Figure 218:

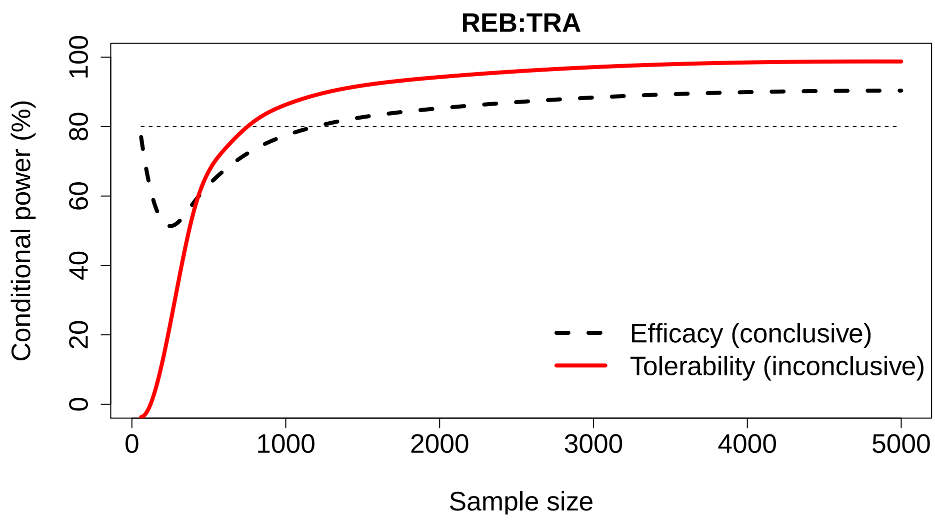

Figure 219:

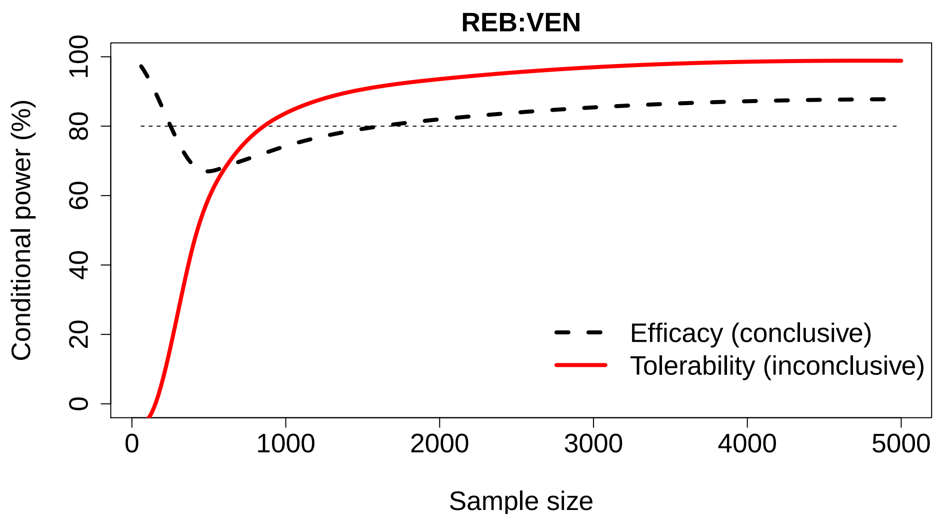

Figure 220:

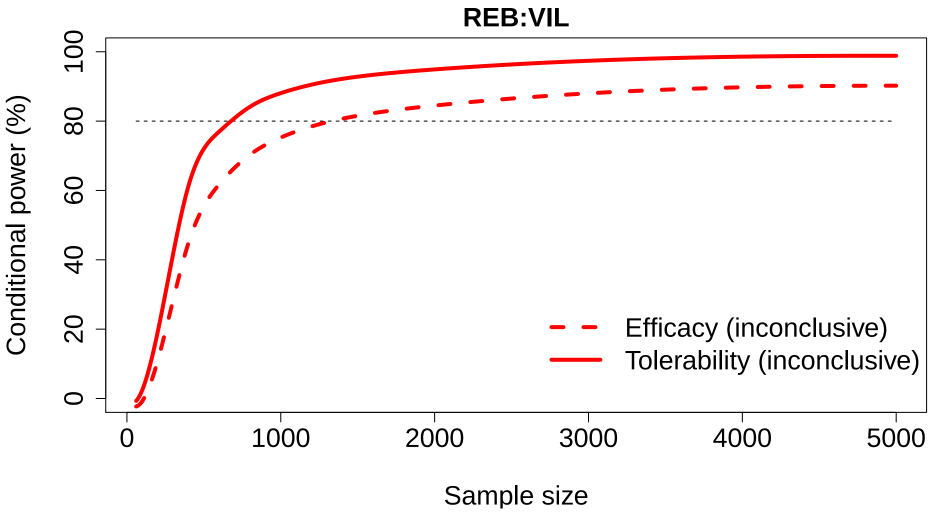

Figure 221:

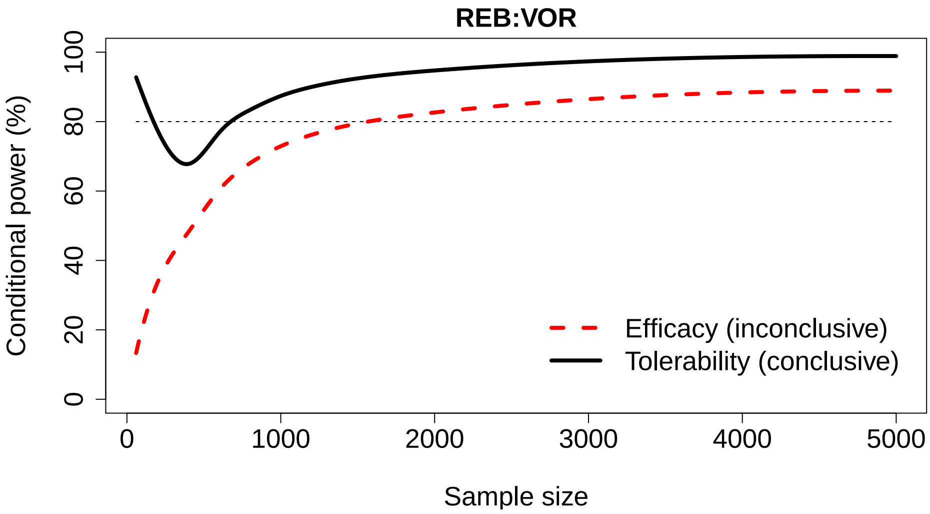

Figure 222:

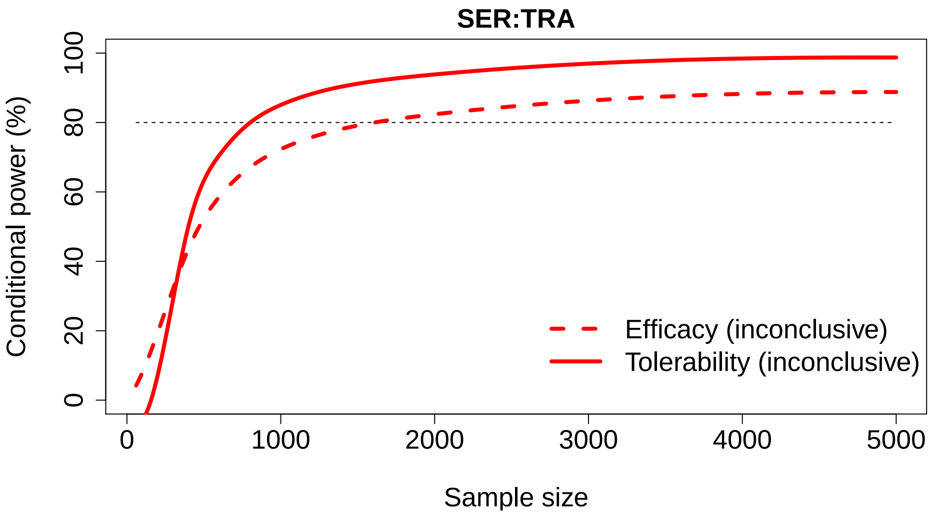

Figure 223:

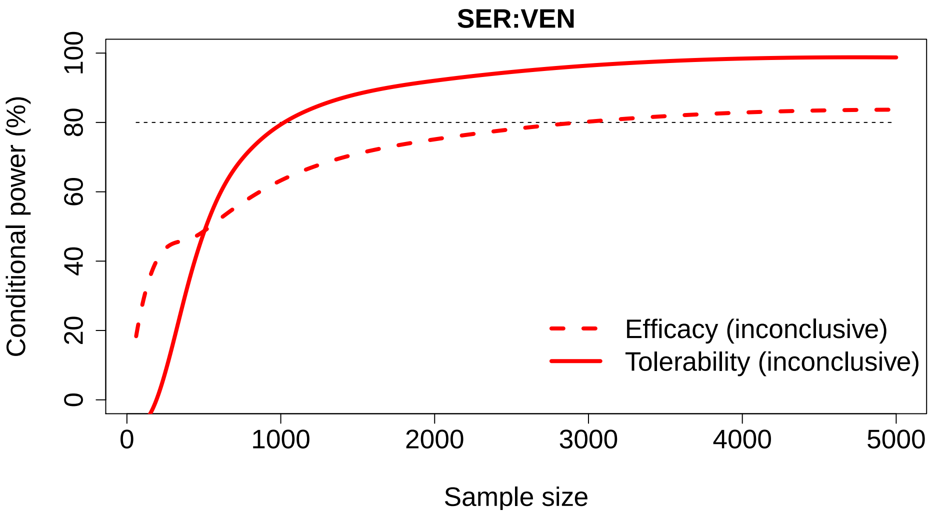

Figure 224:

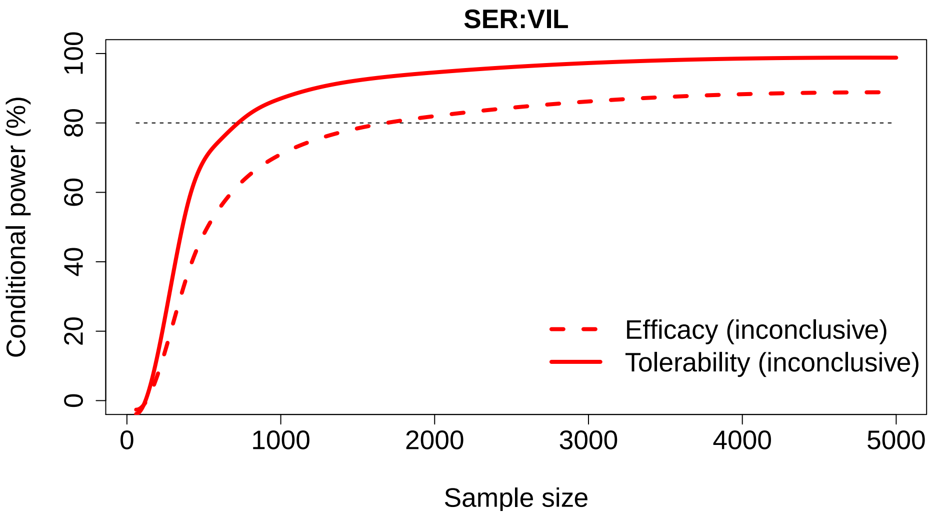

Figure 225:

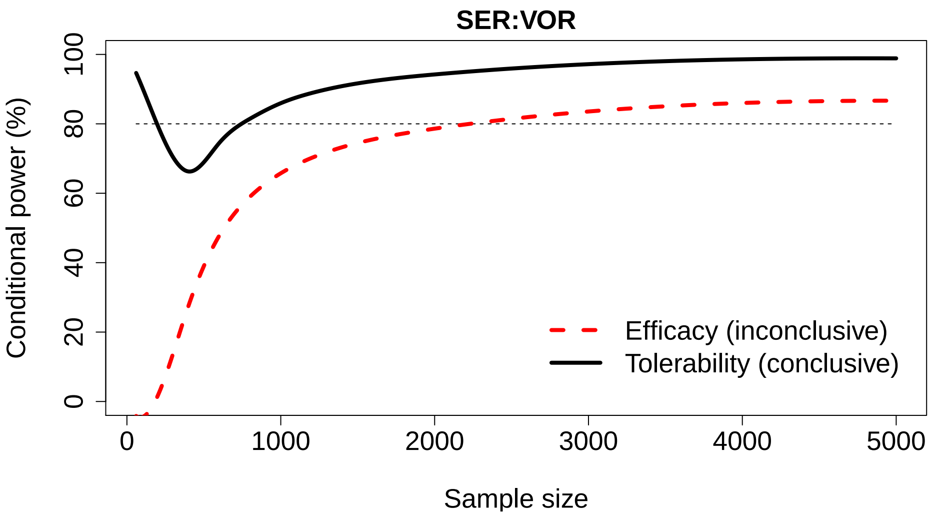

Figure 226:

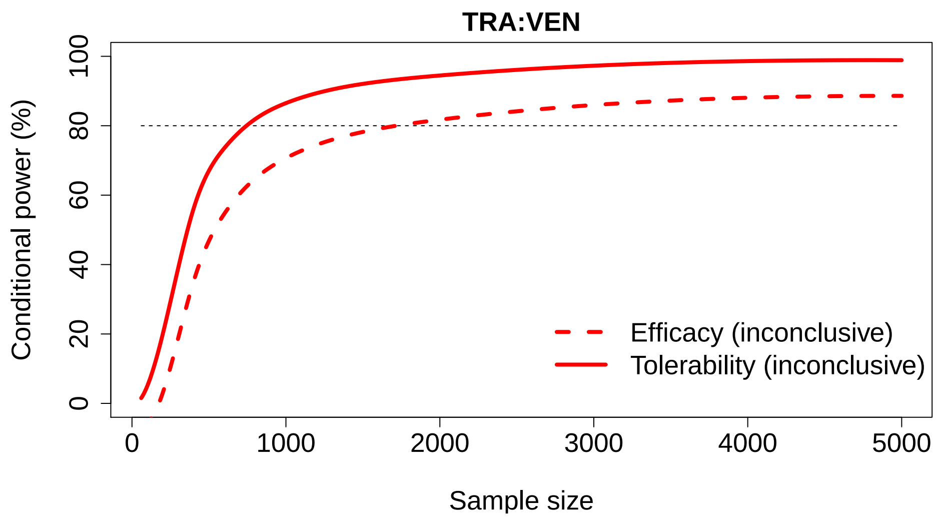

Figure 227:

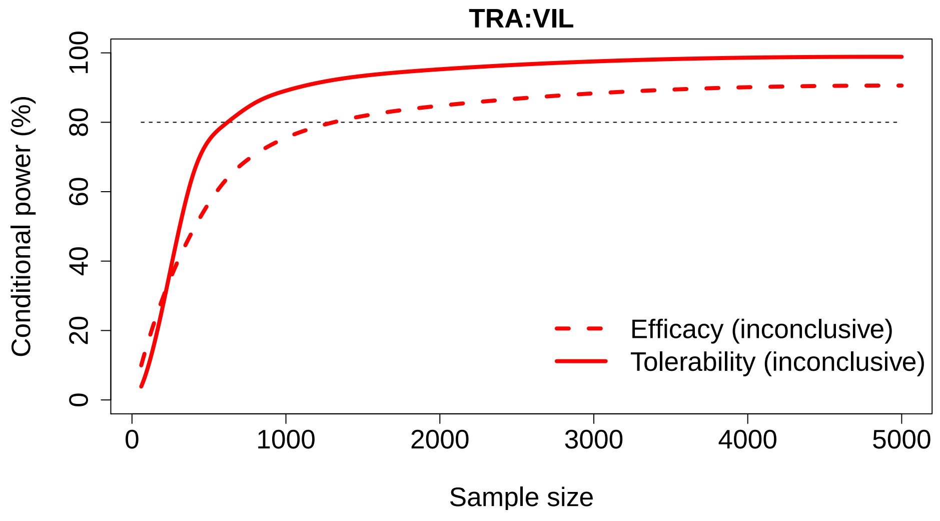

Figure 228:

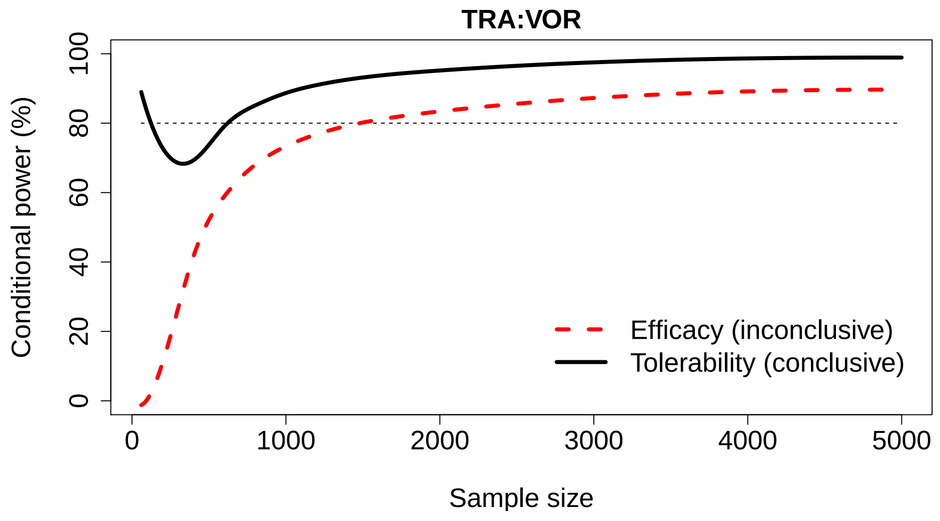

Figure 229:

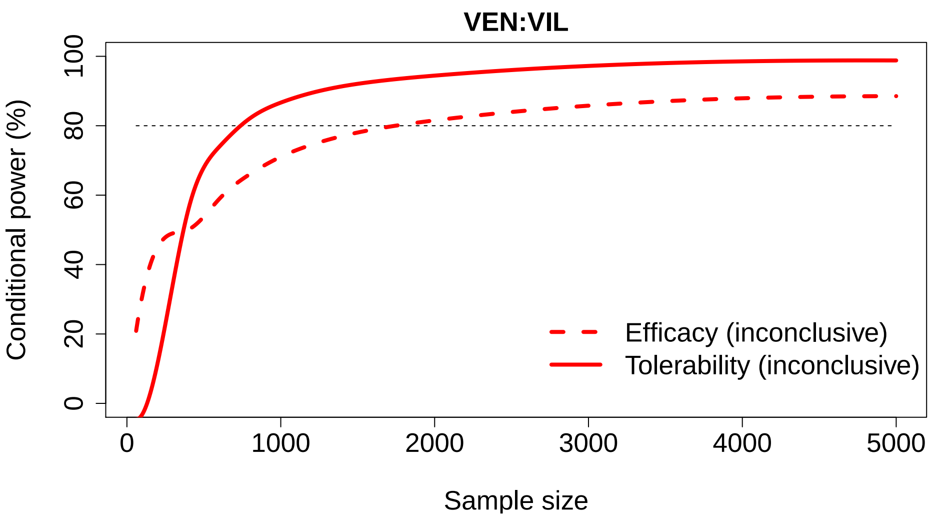

Figure 230:

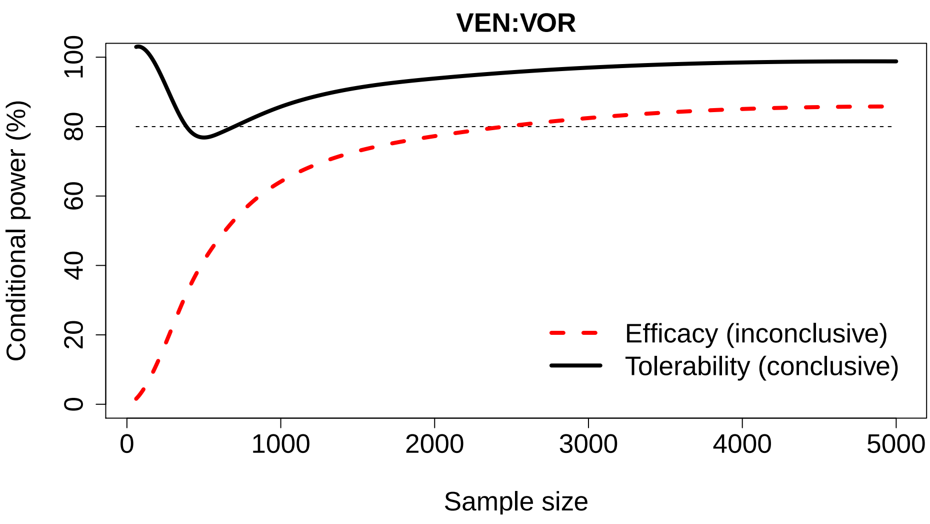

Figure 231:

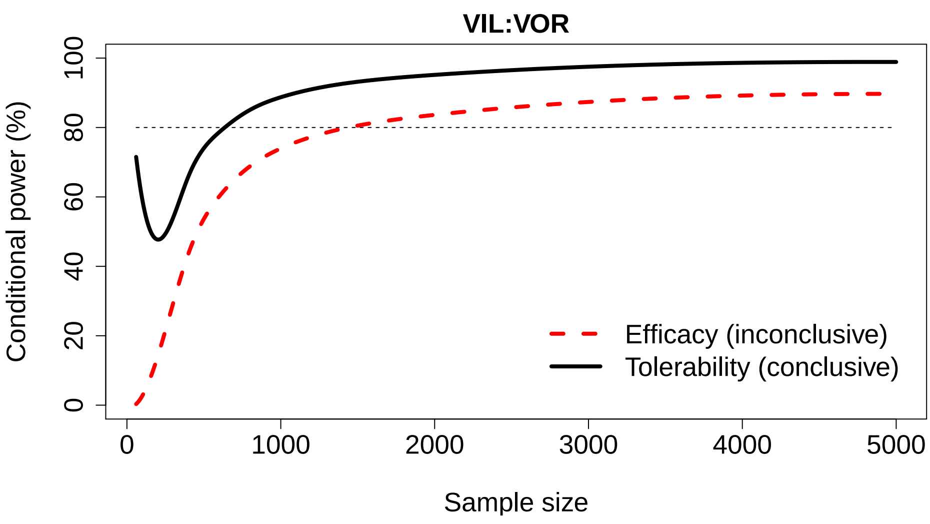

References

1. Nikolakopoulou A, Mavridis D, Salanti G. Using conditional power of network meta-analysis (NMA) to inform the design of future clinical trials [Journal Article]. Biom J. 2014;56(6):973–90.

2. Cipriani A, Furukawa TA, Salanti G, Chaimani A, Atkinson LZ, Ogawa Y, et al. Comparative efficacy and acceptability of 21 antidepressant drugs for the acute treatment of adults with major depressive disorder: a systematic review and network meta-analysis [Journal Article]. The Lancet. 2018;391(10128):1357–1366. Available from: http://dx.doi.org/10.1016/S0140-6736(17)32802-7.

3. Hamilton M. A rating scale for depression [Journal Article]. Journal of Neurology, Neurosurgery, Psychiatry. 1960;23(1):56–62.
